# Visualizing B–N Bond Breaking in Silicone Elastomer Networks through Turn-On Mechanochromic Luminescence

**DOI:** 10.1021/jacs.6c14340

**Published:** 2026-10-14

**Authors:** Beijia Chen, Fernando Vidal, Frieder Jäkle

**Affiliations:** Department of Chemistry, Rutgers University-Newark, 73 Warren Street, Newark, New Jersey 07102, United States

## Abstract

Mechanochromic luminescence (MCL) plays important roles in sensors, encryption, as impact indicators, and in real-time stress mapping. Boron–nitrogen Lewis pairs (LPs) are promising as mechanophores because both the interaction energy and the luminescence color can be readily fine-tuned. However, elastomers based on luminescent organoboron polymers have yet to be explored. Mechanofluorochromic elastomers were prepared by cross-linking pyridyl-substituted poly­(dimethylsiloxane) (PDMS) with luminescent polystyrene (PS)-based organoborane polymeric Lewis acids. Apart from enabling the full compatibilization of otherwise immiscible PS/PDMS matrices, supramolecular B–N bond formation quenched the blue emission of **PS1** via complexation of its pendant bithiophenyl-substituted arylborane chromophore units with pyridyl-functionalized PDMS. Upon stretching of the polymer film, a fluorescence turn-on, corresponding to uncomplexed boron chromophores, was observed by the naked eye under UV irradiation, indicating that the mechanical force was effectively translated to cleave the dynamic B–N bond. Aided by the low *T*_g_ of the PDMS segments, the “frustrated” mechanofluorochromic Lewis pairs (MFLPs) recombined instantly upon stress relief, rendering the material nonfluorescent again. Use of a yellow-luminescent donor-π-acceptor fluorophore that turns blue-emissive after pyridyl binding in **PS2** led to a ratiometric fluorescence response. Tensile tests and oscillatory rheology studies showed that network **F1** was relatively stronger than network **F2**, consistent with the relative Lewis acid strengths and ligand exchange kinetics derived from studies on molecular model systems. These results demonstrate the potential of tunable B–N Lewis pairs as versatile mechanofluorochromic cross-links in polymer networks.

## Graphical abstract

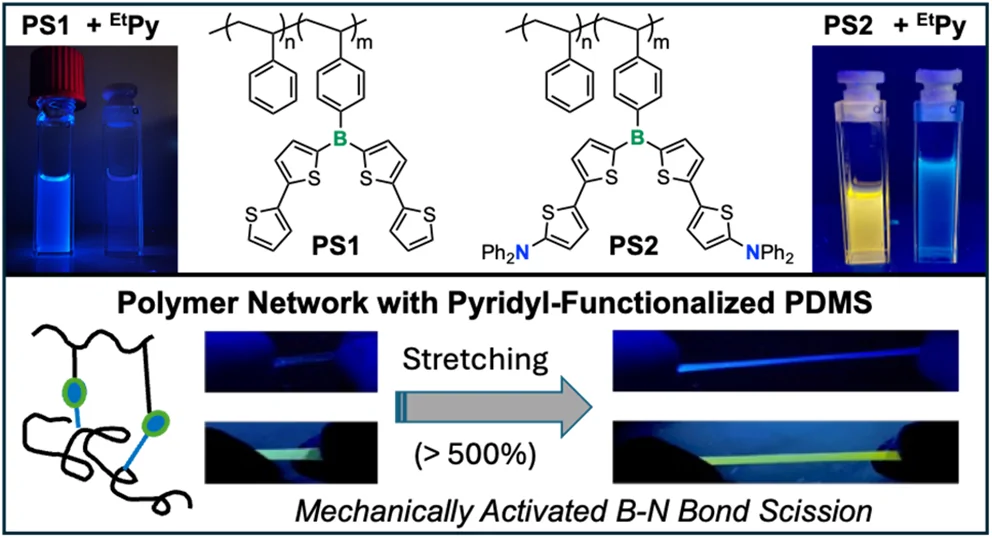

## Introduction

Stimuli-responsive polymers hold great potential for applications in indicators and sensors, smart displays, actuators, robotics, and the biomedical field.^1–7^ Of particular relevance to sensing and molecular detection are chromogenic and fluorogenic polymers that undergo reversible color changes upon exposure to a specific stimulus.^8–16^ Materials that exhibit mechanochromic luminescence (MCL), i.e., reversible changes in emission color in response to external mechanical forces such as grinding, shearing, stretching, or pressing, are promising for advanced sensors, encryption, anticounterfeiting, impact indicators, and real-time stress mapping.^17–28^ The choice of suitable mechanophores, which are molecular units that can quantify and locate force on the molecular scale, thus offering insights into macroscopic behavior of polymeric materials, is critically important.^28–31^ Mechanophores utilized in MCL materials frequently rely on reversible covalent bond formation (e. g., spyropyans,^32^ Diels–Alder adducts,^33^ cyclobutanes^34^), noncovalent interactions, and conformational changes prompted by application of force. Examples of mechanophores that generate MCL through processes other than covalent bond scission include systems based on excimer formation,^35^ rotaxanes that release interlocked fluorophores upon stretching,^36^ bent chromophores that exhibit optical changes upon planarization,^37^ and coordination compounds that incorporate emissive lanthanide ions^38^ (Figure 1a).

**Figure 1:**
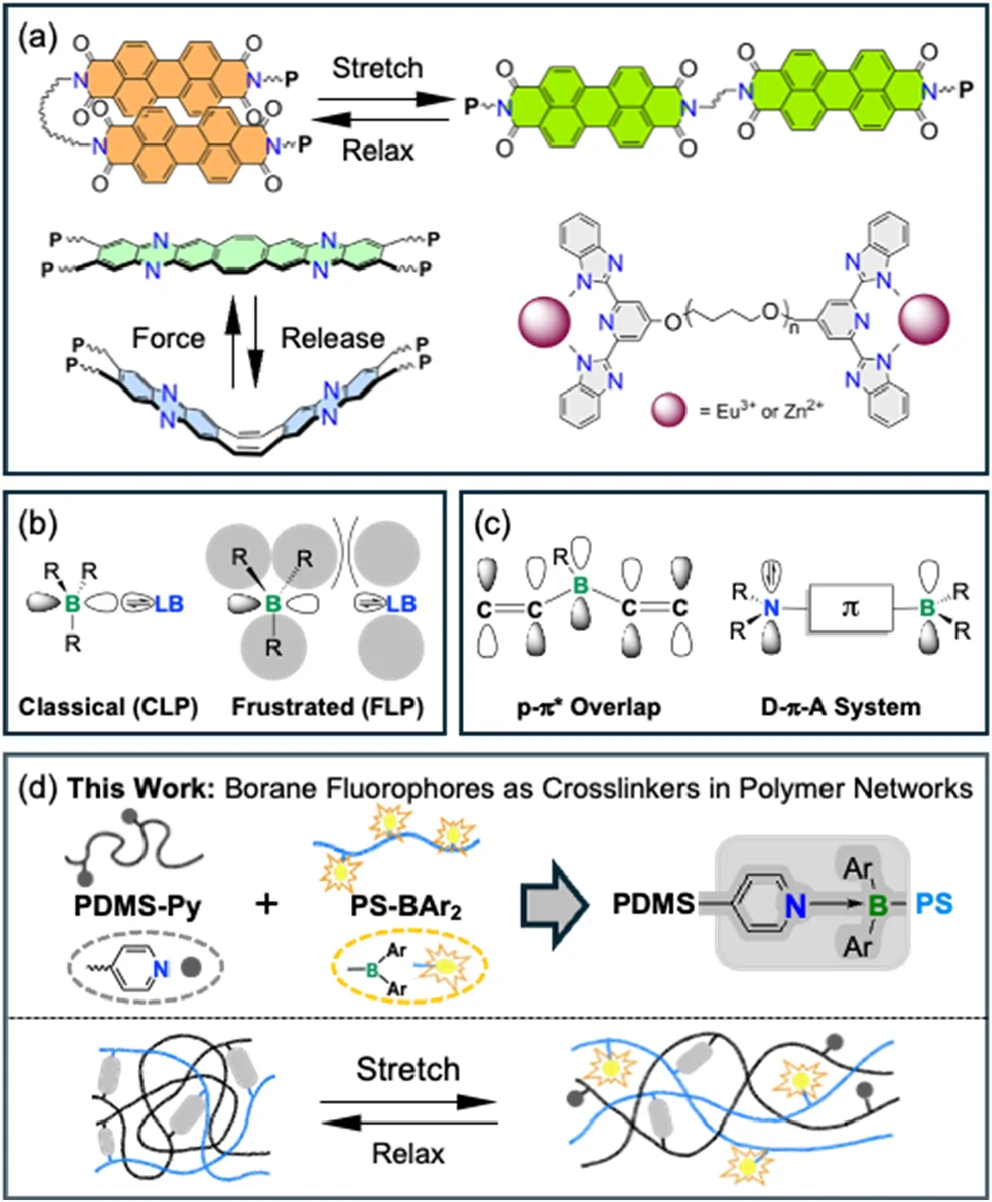
(a) Representative examples of dynamic covalent mechanofluorophores (P = polymer chain or network). (b) Classical Lewis pairs (CLPs) and ‘frustrated’ Lewis pairs (FLPs). (c) Tricoordinate borane fluorophores with p-π* extension of conjugation and donor-π-acceptor character, enabling intramolecular charge transfer (ICT). (d) Schematic illustrations of mechanically induced B–N bond cleavage in polymer networks resulting in reversible emission color changes.

Reversible bond formation is a key feature of typical stimuli-responsive materials and has been extensively utilized in the development of ‘smart’ soft materials.^39–42^ While numerous motifs capable of dynamic bond formation have been explored^43–49^ Lewis acid–base pairs (LPs) have recently emerged as a promising new approach because bond strength can be adjusted over a very wide range through molecular engineering via steric and electronic tuning, and even through photoswitching mechanisms.^50–65^ Classical Lewis pairs (CLPs) feature relatively strong bonds, whereas in the extreme case, steric hindrance in ‘frustrated’ Lewis pairs (FLPs) prevents direct interactions, so that the inherent reactivity of the Lewis acid (LA) and Lewis base (LB) can be exploited for small molecule activation and catalysis applications (Figure 1b).^66–71^ Another important property of tricoordinate organoboranes is their ability to form π-bonds with electron-donating substituents (e.g., X = NR_2_, OR, halides). In addition, overlap of the p-orbital on boron with empty π*-orbitals of conjugated organic substituents such as aromatic, olefinic, or alkynyl groups is frequently observed (Figure 1c).^72–77^ This p-π* overlap tends to lower the LUMO energies and therefore results in bathochromic shifts in absorption and emission.^78^ These shifts are especially pronounced in the case of donor-π-acceptor systems where intramolecular charge transfer (ICT) from a filled orbital on the electron-rich donor (D) to the empty orbital on the boron-centered acceptor (A) is prevalent.^79,80^

Polymers that are decorated with borane LAs have emerged in recent years and have been explored as recyclable catalysts, light-emitting, stimuli-responsive, and sensory materials.^55,71,81,82^ Importantly, when combined with LB-functionalized polymers, reversible cross-linking yields dynamic polymer networks.^50,54,83–87^ In 2018, we first demonstrated that the binding affinity of PS-bound tricoordinated dithienylborane as LA to poly­(dimethylsiloxane) (PDMS)-bound pyridine as LB is sufficient to overcome the unfavorable free energy of mixing, yielding clear, homogeneous polymer blends with self-healing properties.^84^ In subsequent studies, we combined different borane LAs (catecholboronate, di­(thiophen-2-yl)­borane) with amine LBs (pyridine, imidazole) to tune the adduct formation, achieving equilibrium constants and B–N dissociation rate constants spanning over 7 orders of magnitude.^54^ When introduced into PS–PDMS polymer blends, a 10,000-fold increase in the zero-shear viscosity could be realized from the weakest to the strongest LP interactions. The potential application of LPs to industrially relevant materials was exemplified by the fabrication of silicone elastomers with dynamic cross-links, which enabled facile melt reprocessing, unlike permanently cross-linked elastomers.^40,41^ The addition of nanoparticles as a filler and the use of rigid PS as a cross-linker yielded a doubly reinforced elastomer with tensile properties superior to those of the PDMS precursor and recoverable by melt reprocessing.

A particularly appealing aspect of boron-containing LPs is that multistimuli responsiveness can be achieved by leveraging differences in optical properties between bound and unbound states.^73,88–92^ This feature has been exploited in chemosensing, most notably the detection of fluoride and cyanide anions and of amines,^93–97^ but remains underexplored in the development of B–N LP-cross-linked elastomers. Building on the discoveries described above, we designed functional PS–PDMS networks that exhibit fluorescence turn-on under tension (Figure 1d). In **PS1**, the PS chains are rendered blue fluorescent by introducing bis­(bithiophenyl)­borane chromophores as side groups. As the dynamically cross-linked polymer network forms upon addition of pyridine-functionalized PDMS, the fluorescence is quenched by reversible supramolecular B–N bond formation. Diarylamine-substituted bithiophenes as pendent groups in **PS2** lead to yellow luminescence due to intramolecular charge transfer (ICT) from nitrogen to boron in the photoexcited state of the D-π-A chromophores. As a result, the polymer’s luminescence is visible to the naked eye in both the unbound (yellow) and bound (blue) states, but with different emission colors. We demonstrate here that mechanically induced cleavage of the dynamic B–N bonds upon stretching the bulk material restores the original fluorescence of some of the boron-containing chromophores, leading to an MCL response. Because of the polymer network’s elasticity, aided by the low glass transition temperature of the PDMS segments, the ‘frustrated’ mechanofluorochromic Lewis pairs (MFLPs) can recombine instantly upon stress relief, regenerating the optical properties of the bound state. These effects offer an opportunity to directly visualize B–N bond cleavage under mechanical force, such as stretching the specimens, and enable real-time stress mapping.

## Results and Discussion

### Synthesis and Characterization of Polymer Precursors and Model Systems

The triarylborane-functionalized polymers **PS1** and **PS2** were prepared from the styrene-based random copolymer P­(St-*co*-StSiMe_3_) as illustrated in Scheme 1, in analogy to previously developed methods for the synthesis of PS-based polymeric LAs.^54,95,98^ The molecular weight of the precursor polymer P­(St-*co*-StSiMe_3_) was determined by gel permeation chromatography (GPC) in THF relative to narrow polystyrene standards (*M*_n_ = 24,450 Da, *M*_w_ = 27,510 Da, *Đ* = *M*_w_/*M*_n_ = 1.13), and the ratio of incorporated monomers was assessed by ^1^H NMR analysis to be *n*/*m* = 8:1 (Figure S1, SI). Selective exchange of SiMe_3_ with BBr_2_ moieties was accomplished by treatment with BBr_3_ in DCM, and Br substituents on boron were subsequently exchanged with bithiophenyl groups using stannylated reagents. The triarylborane polymers were isolated as bright yellow (**PS1**) and dark orange (**PS2**) powdery solids in moderate to high yields after precipitating the one-pot reaction mixture from hexanes under an inert atmosphere. Molecular model compounds **M1** and **M2** were prepared using similar methods as described in the Supporting Information.

**Scheme 1:**
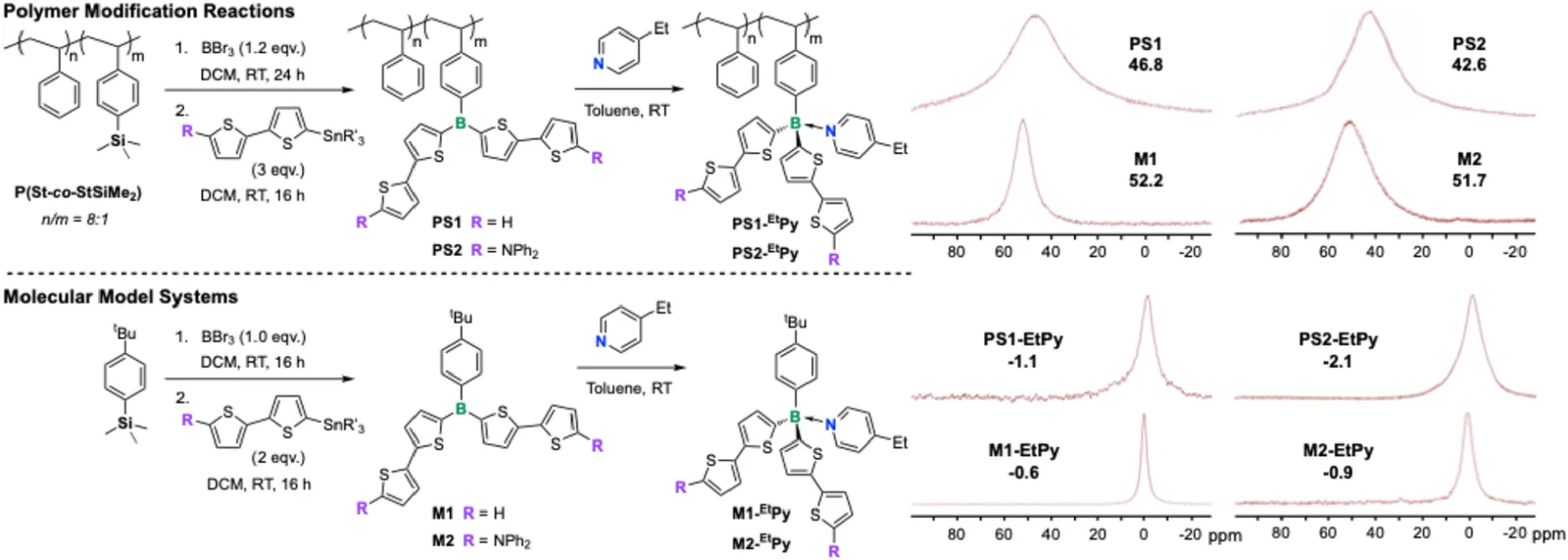
Synthesis of Arylborane Polymers **PS1** and **PS2** and Model Systems **M1** and **M2**, Complexation with 4-Ethylpyridine (^Et^Py), and ^11^B NMR Spectra of the Isolated Products with Chemical Shifts Given in ppm

Analysis of the isolated polymers confirmed quantitative conversion of -SiMe_3_ to -BAr_2_ groups, evidenced by the disappearance of the methyl signal at 0.30 ppm in the ^1^H NMR, and the emergence of broad new signals at 46.8 ppm (**PS1**) and 42.6 ppm (**PS2**) in the ^11^B NMR spectra (Scheme S1). The chemical shifts of the ^11^B signals are characteristic of moderately Lewis acidic tricoordinate boron centers.^99^ Integration of the ^1^H NMR signals confirmed that the average degree of functionalization remained at ca. 11%. To demonstrate the suitability for B–N LP formation, 4-ethylpyridine (^Et^Py) was reacted with the polymeric LAs to form polymers **PS1-** ^**Et**^ **Py** and **PS2-** ^**Et**^ **Py** (Scheme 1). The complexation was accompanied by rapid color changes from bright yellow to colorless (**PS1-** ^**Et**^ **Py**) and from orange to faint yellow (**PS2-** ^**Et**^ **Py**), respectively. The isolated polymer complexes showed much sharper, largely upfield-shifted ^11^B NMR signals at −1.1 ppm and −2.1 ppm, respectively, demonstrating complete conversion to tetracoordinate boron centers. The ^11^B NMR chemical shifts are in a similar range to those of the molecular model systems **M1** and **M2**, but are, as expected, significantly broadened and slightly upfield-shifted due to neighboring-group effects when incorporated into the polymers.^95^ With the boron sites complexed, **PS1-** ^**Et**^ **Py** and **PS2-** ^**Et**^ **Py** were rendered air-stable, enabling molecular weight determination by GPC in THF (Figure S1) and analysis of the thermal properties by DSC and TGA (Figures S2 and S3 and Table S1). The measured molecular weights were close to those predicted for the replacement of SiMe_3_ groups in P­(St-*co*-StSiMe_3_) with BAr_2_ groups (Table 1). A trend toward slightly lower molecular weights may have resulted from repeated redissolution, filtration, and precipitation during isolation, which could have removed some less-soluble, high-molecular-weight chains.

**Table 1:** Characteristics of Lewis Acid (LA) and Lewis Base (LB) Functionalized Polymers Employed in the Formation of PS/PDMS Network Materials

	FG^a^	*M*_n_ ^b^ (kDa)	*Đ* ^b^	*F* ^c^ (mol %)	*F*_av_ per chain^d^
**P(St-** ***co*** **-StSiMe**_**3**_)	SiMe_3_	24.4	1.13	0.11	24.3
**PS1-** ^**Et**^ **Py** ^e^	B(Th_2_)_2_	21.0	1.19	0.11	15.0
**PS2-** ^**Et**^ **Py** ^e^	B(Th_2_NPh_2_)_2_	23.0	1.25	0.11	13.4
**PDMS-Py** ^f^	4-Py	409	1.48	0.50	27.3

a Functional groups (FG) attached to polymer chain.

b Number-average molecular weight (*M*_n_) and dispersity (*Đ*) determined by GPC in THF against polystyrene (PS) standards.

c mol % of functional groups attached to polymer (*F*) from integration of ^1^H NMR spectra of the isolated polymers.

d Average number of functional groups (*F*_av_) calculated from *M*_n_ and *F* (mol %).

e Measured after the addition of 4-ethylpyridine to samples of **PS1** and **PS2**.

f See discussion on polymer networks formation.

Photophysical studies on the polymeric LAs **PS1** and **PS2**, the ^Et^Py-complexed polymers **PS1-** ^**Et**^ **Py** and **PS2-** ^**Et**^ **Py**, and the corresponding molecular model systems were performed in toluene (Figures 2 and S4–S14 and Tables S2 and S3). Upon excitation at the absorption maximum of 405 nm, **PS1** showed a blue emission with a maximum at 446 nm and a quantum yield of 9.4%, whereas excitation of **PS2** at its absorption maximum of 471 nm resulted in a yellow emission at 550 nm with a quantum yield of 17.5% (Table 2). The absorption and emission features are overall similar to those of the molecular systems **M1** and **M2**, except that the emission of **PS2** is broader than that of **M2**. This indicates that the photophysical properties are largely retained in the polymeric materials and that the chromophores act independently. The results also confirm that the complexation of boron with nitrogen LBs significantly alters molecular orbital energies and fluorescence properties. The spectra of the adducts revealed a large hypsochromic shift in both absorption and emission after complexation. Of note, the emission intensity of **PS1-** ^**Et**^ **Py** is almost fully quenched, whereas **PS2-** ^**Et**^ **Py** shows a blue emission at 476 nm with a reduced quantum yield of 3.5%, allowing for ratiometric detection of pyridine binding. Time-resolved fluorescence and quantum yield measurements revealed that the emission quenching is primarily due to a large reduction in the radiative rate constants, *k*_r_ (Table 2).

**Figure 2:**
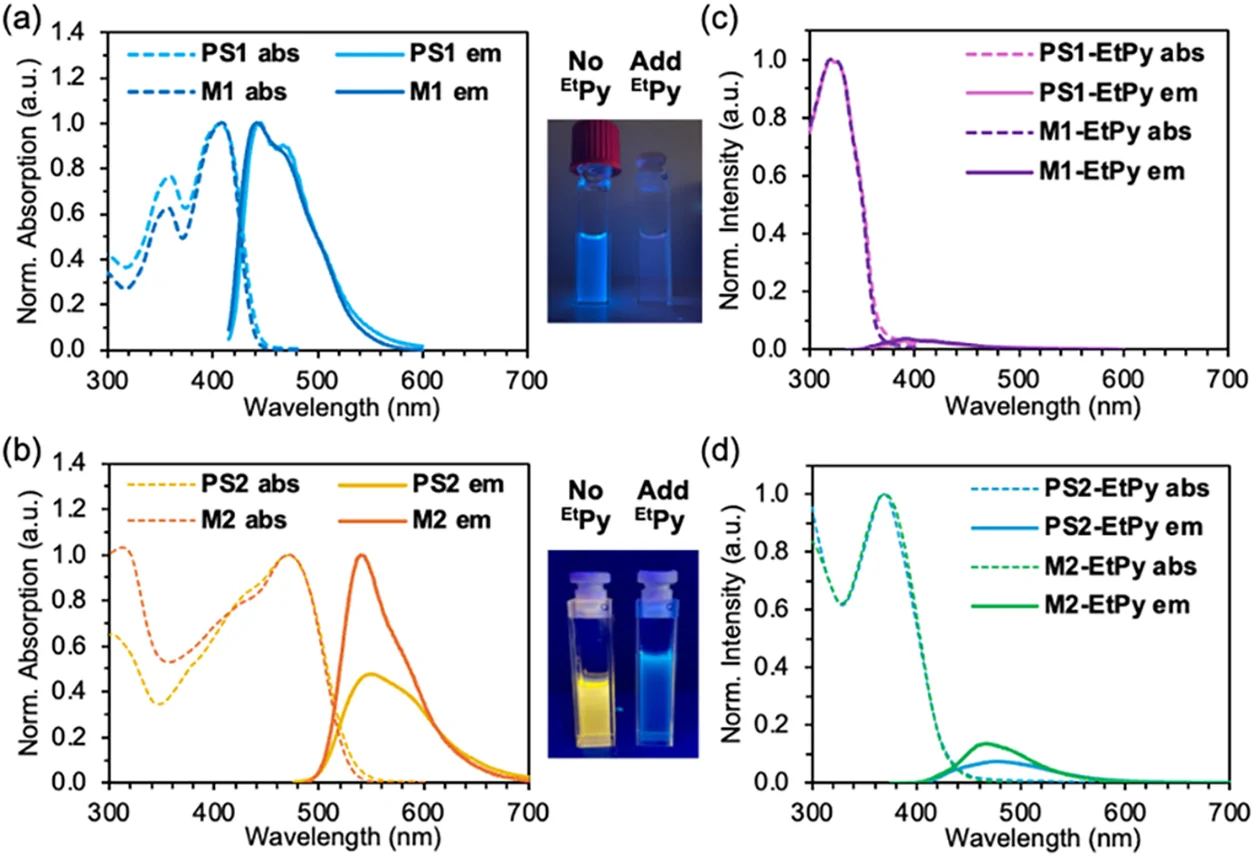
UV–vis absorption and emission spectra of (a) **PS1** and **M1**, (b) **PS2** and **M2**, (c) **PS1-** ^**Et**^ **Py** and **M1-** ^**Et**^ **Py**, and (d) **PS2-** ^**Et**^ **Py** and **M2-** ^**Et**^ **Py** (chromophore concentration ca. 3.0 × 10^–5^ M; a large excess of ^Et^Py was added for the complexes). The emission intensities for **PS1** and **PS2** are scaled relative to those of the free organoborane LAs **M1** and **M2**, considering the relative quantum yields and the area under the emission curve; similarly, for the complexes, the emission intensity is shown relatively to that of **M1** and **M2** respectively, considering the relative quantum yield, the area under the emission curve, and the absorbance at the excitation wavelength (for further details, see the SI, and for unscaled data, see Figure S4). Insets show photographs of solutions of **PS1** and **PS2** irradiated with a 365 nm UV lamp before and after the addition of an excess of ^Et^Py.

**Table 2:** Photophysical Data and Comparison with Computed Data for Model Compounds (CAM-B3LYP, 6-31G­(d))

	experimental data (toluene solution)							TD-DFT results (CAM-B3LYP)^a^			
	λ_abs_ (nm)	ε (10^4^ M^–1^cm^–1^)	λ_FI_ ^b^ (nm)	Φ_Fl_ ^c^ (%)	τ_av_ ^d^ (ns)	*k*_r_ ^e^ (10^7^ s^–1^)	*k*_nr_ ^e^ (10^7^ s^–1^)	λ_abs_ (nm)	*f*_abs_ ^f^	λ_Fl_ (nm)	*f*_Fl_ ^f^
**PS1**	405	n.d.	446	9.4	0.60	15.7	151	--	--	--	--
**M1**	409, 357	4.12, 2.55	442	9.5	0.42	22.6	215	366, 317	0.98, 0.43	401	1.05
**PS2**	471	n.d.	550	17.5	0.97	18.0	85.1	--	--	--	--
**M2**	472, 313	3.63	540	27.0	1.07	25.2	68.2	418, 368	1.51, 0.45	461	1.67
**PS1-** ^**Et**^ **Py**	321	n.d.	395	<0.5^g^	0.42	<1	>230	--	--	--	--
**M1-** ^**Et**^ **Py**	320	3.31	392	<0.5^g^	<0.25^g^	n.d.	n.d.	335, 306	0.00, 0.84	599, 336	0.00, 0.70
**PS2-** ^**Et**^ **Py**	368	n.d.	476	3.1	1.03	3.0	87.6	--	--	--	--
**M2-** ^**Et**^ **Py**	369	3.78	466	9.9	1.01	9.8	89.2	351, 347	0.69, 0.58	593, 390	0.00, 1.07

a Pyridine and benzene rings were used in the calculations instead of 4-ethylpyridine and 4-*tert*-butylphenyl.

b Excited at the longest wavelength absorption maximum.

c Fluorescence quantum yield determined using an integrating sphere.

d Average fluorescence lifetime.

e Radiative (*k*_r_) and nonradiative (*k*_nr_) decay rate constants calculated using the equations *k*_r_ = Φ/τ and *k*_nr_ = (1 – Φ)/τ.

f Computed oscillator strength.

g Lifetime/quantum yield too low to accurately determine.

To further assess the effect of pyridine complexation on orbital energy levels and transition energies, DFT and TD-DFT calculations were performed on the molecular models, **M1** and **M2**, as well as the complexed species **M1-Py** and **M2-Py** (Figures S15 and S16 and Tables S4–S9 and S17–S28). For simplification, pyridine and benzene rings were used in the calculations instead of 4-ethylpyridine and 4-*tert*-butylphenyl groups (Figure 3 and Table 2). The computational results correlate well with the photophysical data of the synthesized models and polymers. The electron-rich diphenylamino groups of **M2** raise the HOMO, resulting in a largely reduced HOMO–LUMO gap. Results of TD-DFT calculations at the CAM-B3LYP level were consistent with the red shifts in the absorption and emission maxima of **M2** relative to those of **M1**. High oscillator strengths are predicted for the lowest-energy absorption and emission of **M1**, and even more so of **M2**. However, consistent with the findings of Ren et al.,^57^ after complexation, the lowest-energy transition corresponds to ICT from bithiophene to pyridine-centered orbitals, which has a very low oscillator strength. More intense S_0_-S_3_ transitions are π–π* in nature, involving HOMO and LUMO+2 orbitals, both of which are localized on the bithiophene moieties.

**Figure 3:**
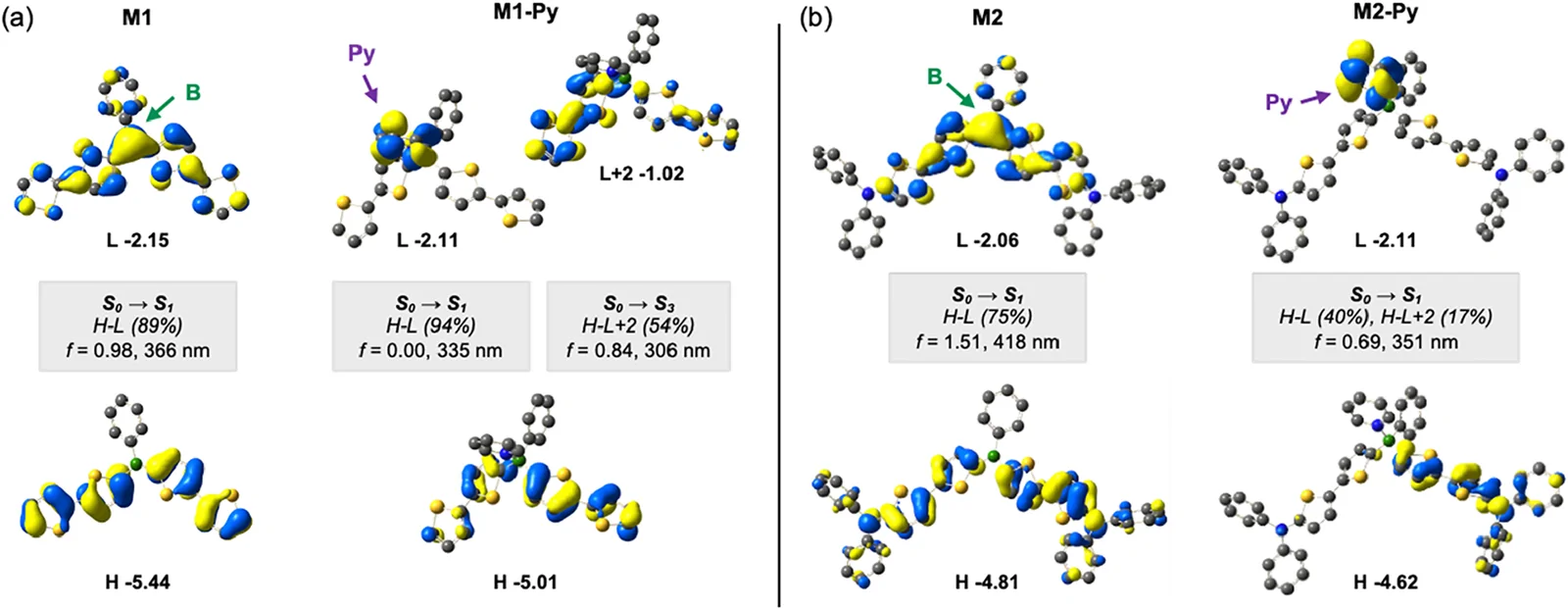
Molecular orbital plots, computed lowest energy optical transitions, and calculated HOMO–LUMO energy levels for (a) **M1**/**M1-Py** and (b) **M2**/**M2-Py**. DFT calculations were performed on model compounds with *tert*-butyl and ethyl groups replaced with H atoms at the B3LYP/6–31G­(d) level of theory, and the surface contours are displayed at an isovalue of 0.04. TD-DFT calculations were performed at the CAM-B3LYP/6–31G­(d) level of theory.

The thermodynamic binding affinity and the kinetics of bond formation and cleavage are critical to the performance of B–N LP-cross-linked polymer networks.^54^ To assess the thermodynamic propensity for adduct formation and the corresponding exchange kinetics, UV–vis titrations and variable-temperature (VT) NMR experiments were performed on molecular model compounds **M1** and **M2** in toluene (or *d*_*8*_-toluene) solutions. Toluene was chosen as a solvent because it is expected to closely mimic the microenvironment of the polymeric LPs in the film state. UV–vis titration of the model LAs by adding increments of 4-ethylpyridine revealed clear isosbestic points, and fitting of the absorption data using the HypSpec software (Figures S17–S20 and Table S10) yielded binding constants, *K*_eq_, of 5.0 × 10^6^ M^–1^ (**M1**) and 3.2 × 10^6^ M^–1^ (**M2**) (Figure 4a,b and Table 3) that are comparable to that of a borane with only a single thiophene ring as substituents (*K*_eq_ = 6 × 10^6^ M^–1^).^54^ The slightly lower binding constant for **M2**, indicative of reduced LA strength, is anticipated given the electron-donating effect of the diphenylamino substituents and is consistent with the relatively higher LUMO energy level inferred from computational studies. Nevertheless, the results suggest that both polymer-supported borane LAs bind pyridine LBs effectively, thereby enabling the construction of robust B–N LP-cross-linked polymer networks.

**Figure 4:**
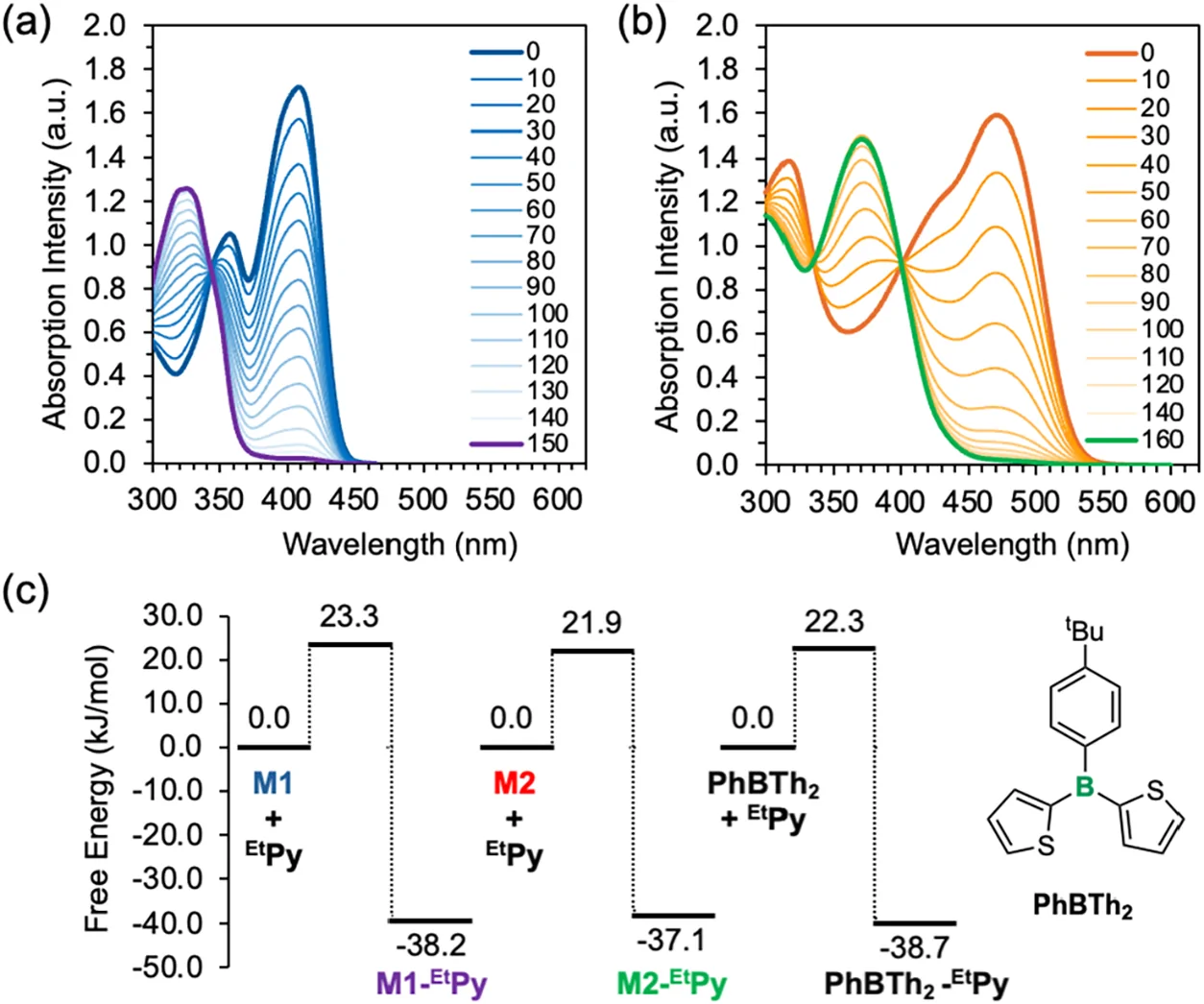
Titration of (a) **M1** and (b) **M2** in toluene (*c* = 4.6 × 10^–5^ M) with a solution of 4-ethylpyridine (^Et^Py) in toluene (cumulative amounts added are indicated in the figure in units of μL) monitored by UV–vis absorption spectroscopy. (c) Energy landscapes for the complexation of **M1** and **M2** with ^Et^Py, compared with the borane **PhBTh**_**2**_.^54^

**Table 3:** Equilibrium Constants and Activation Parameters for 4-Ethylpyridine Binding to Model Complexes

LA	LB	*K*_eq_ ^a^ (M^–1^)	Δ*G*_diss,298 K_^‡^ (kJ/mol)	*k*_diss,298 K_ ^b^ (s^–1^)
**M1**	^Et^Py	5.0 × 10^6^	61.5	103
**M2**	^Et^Py	3.2 × 10^6^	59.0	287

a From VT ^1^H NMR analysis in d^8^-toluene at [**M**] = 0.04 M.

b From UV–vis titration in toluene at [**M**] = 4.6 × 10^–5^ M and global data fitting at 108 (**M1**) and 141 (**M2**) different wavelengths.

To examine the dynamic exchange of the B–N LPs, VT-NMR spectra were acquired for the coordinated species (**M1-** ^**Et**^ **Py**, **M2-** ^**Et**^ **Py**) in the presence of an additional equivalent of free ^Et^Py ([LB]/[LA] = 2:1). Spectral data acquired over a temperature range from −20 °C to +50 °C revealed the expected exchange equilibrium between free and bound N donors, with two sets of signals merging into a single set as the temperature increased (Figures S22 and S23). Full line-shape analysis of the ^Et^Py signals, followed by Eyring-plot fitting, yielded the respective activation energies (Δ*G*_*a*_^≠^) and rate constants for B–N bond dissociation (*k*_diss_) at 298 K (Tables 3 and S11 and S12 and Figure S21–S23). The difference in the dissociation barrier between all reported thiophene-substituted arylboranes with ^**Et**^ **Py** was very small, ΔΔ*G*_diss_^‡^ = 2.5 kJ/mol,^54^ indicating equally feasible exchange dynamics (Figure 4c). However, it is worth noting that a somewhat higher rate constant was determined for **M2-** ^**Et**^ **Py** than for **M1-** ^**Et**^ **Py** and a related derivative with single thiophene groups on boron (**PhBTh**_**2**_, *k*_diss_ = 130 s^–1^),^54^ indicating that weaker binding of ^Et^Py also leads to more rapid exchange. The energy landscapes for the complexation of **M1** and **M2** with ^Et^Py are illustrated in Figure 4c and compared with those of **PhBTh**_**2**_.

Computational studies on **M1-** ^**Et**^ **Py** and **M2-** ^**Et**^ **Py** gave B–N bond lengths of ca. 1.69 Å and N–B–C_Ph_ bond angles (104.6–107.1°) that are similar for both complexes and within the typical range for B–N LPs.^59^ To further assess their suitability as mechanophores, we performed calculations in which the pyridyl LB and borane LA were gradually separated by stepwise increases in the distance between the anchoring atoms on the pyridyl and phenyl units (Figure S24). A total of 15 steps were performed to increase the distance by 1.5 Å. Total energy plots derived from structure optimization at each step revealed that at a 1.3 Å increase in distance, the overall energy dropped sharply, the geometry changed dramatically, and the B–N distance increased to 3.5 Å, indicating the breaking of the B–N bond. The maximum energy at an elongation of 1.2 Å was calculated to be very similar for both model systems, with *E*_max_ = 37 kJ/mol, corresponding to a force of *F*_max_ = 0.51 nN. The results are reasonably consistent with those derived experimentally from VT NMR spectroscopy (Table 3) and verify that deformations of the complex structure induced by targeted separation of anchor points can lead to sudden B–N bond cleavage under moderate force.

### Polymer Network Assembly and Characterization

For the assembly of polymer networks containing B–N Lewis pairs as cross-linking sites, PDMS was chosen as the matrix due to its low glass transition temperature (*T*_g_), enabling the formation of flexible elastomers that combine rapid reorganization with strain-induced toughening characteristics.^12,100–105^ When mixing pyridine-functionalized PDMS (**PDMS-Py**, *M*_n_ = 409 kDa, 0.5 mol %) with borane-functionalized **PS1** and **PS2** as multifunctional cross-linkers in toluene (1:1 molar ratio of boron to pyridine), instant gelation was observed (see Tables 1 and S13 for precursor polymer data). The organogels were annealed at 70 °C for 24 h to produce homogeneous, highly viscous solutions. Free-standing transparent elastic films (**F1** from **PS1**, **F2** from **PS2**) were obtained by pouring the solutions into PTFE molds and allowing solvent evaporation under ambient conditions in air. The films were further dried under vacuum and analyzed by TGA to confirm complete removal of toluene (Figure S25). The color of the **PS** solutions in toluene changed upon mixing with **PDMS-Py**, producing very light-greenish films of network **F1** and yellow films of network **F2** (Figure 5). These color changes are consistent with changes in the absorption seen upon complexation of **PS1** and **PS2** with 4-ethylpyridine (Figure S4), indicating B–N LP formation at the designated cross-linking sites.

**Figure 5:**
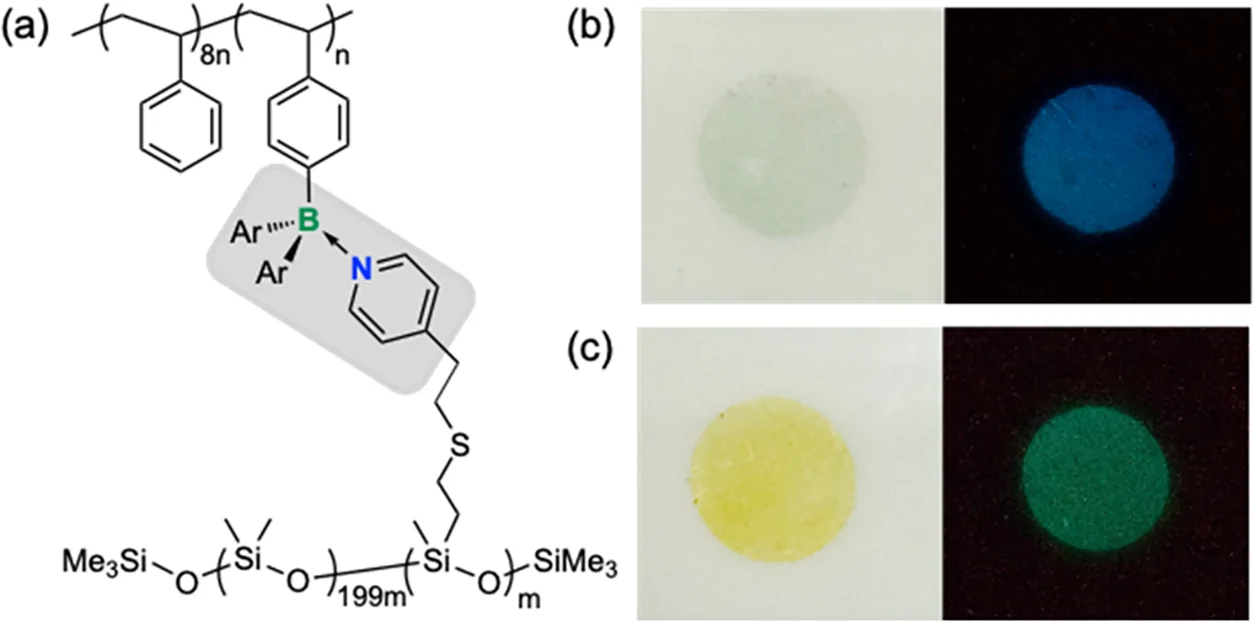
(a) Schematic illustration of network formation. Photographs of free-standing films of (b) **F1** (Ar = BTh) and (c) **F2** (Ar = BThNPh_2_) under ambient light (left) and irradiated with a UV lamp at 365 nm (right).

To assess the mechanical properties, rheological and tensile tests were performed on networks **F1** and **F2** (Figures 6 and S26–S29). Stress–strain curves showed strain hardening, a characteristic of cross-linked polymers (Figure 6a).^106^ When using similar chemical compositions, sample dimensions, and testing conditions, the Young’s modulus, *E*, of **F1** was very similar to that of **F2** at 220 kPa, consistent with soft supramolecular elastomeric materials having a similar degree of cross-linking (Figure S26d). However, the higher stress in **F1** than in **F2** beyond the initial linear region suggests that the dynamic bonds are weaker and the dynamic exchange in **F2** is more rapid. This indicates that the different exchange kinetics of the B–N LP cross-links, as derived from the VT NMR studies on the model systems, affects the mechanical strength of the bulk material. Stress-relaxation curves at 25 °C were recorded by holding the specimen at 300% strain over 10 min, and the corresponding stress values were recorded (Figure 6b). A slightly larger relative decrease in stress for **F1** (ca. 32% reduction) than for **F2** (ca. 22% reduction) was seen, most likely because the **F1** specimen’s initial stress was higher; however, the stress remained higher for **F1** than for **F2**. Creep tests were also performed at 25 °C by holding the specimen at 0.1 N and recording the strain values over 20 min (Figure S26c). They further confirmed that network **F2** was softer compared with **F1**, showing larger deformation over the same time scale. Importantly, both the stress-relaxation and creep experiments demonstrate that a certain level of chain flow is possible at room temperature due to the disentanglement of the PDMS chains enabled by the dynamicity of the LP cross-links.

**Figure 6:**
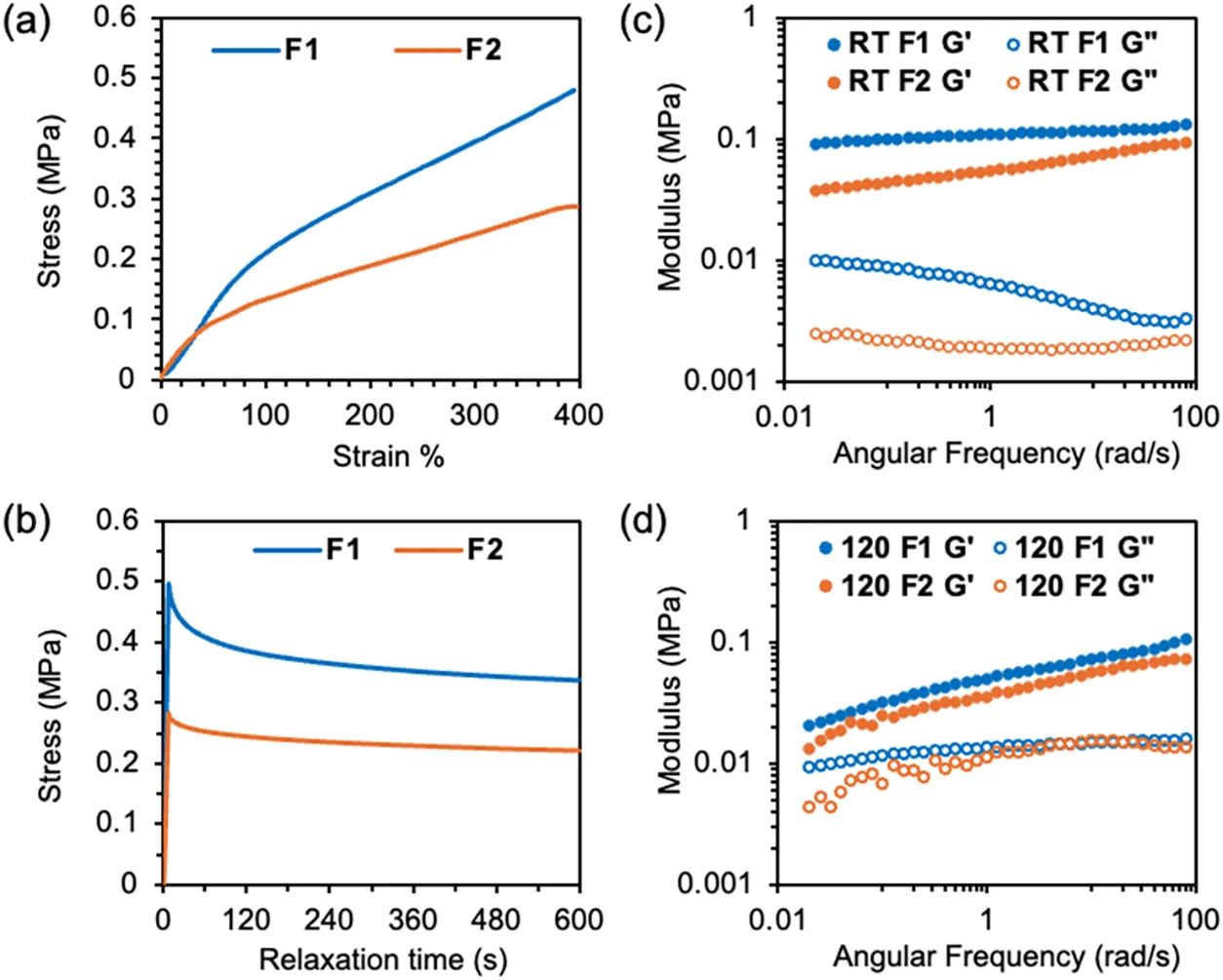
(a) Stress–strain curves to 400% strain for **F1** and **F2**, and (b) stress relaxation curves from tensile tests. (c, d) Frequency sweeps at room temperature (c) and 120 °C (d) showing changes in the storage (G’) and loss (G”) moduli for **F1** and **F2**.

Oscillatory frequency sweep curves showed that **F1** and **F2** behave as typical elastomers, with relatively larger storage moduli indicative of highly cross-linked materials that exhibit little change in modulus over the frequency range once the ‘rubbery plateau’ is reached (Figure 6c). Frequency sweeps were also recorded at elevated temperatures to construct master curves for **F1** and **F2** using time–temperature superposition (TTS) with a reference temperature of 65 °C (Figure S28). No crossover between the storage modulus *G*′ and the loss modulus *G*″ was observed for either network at temperatures up to 120 °C, but the storage modulus decreased steadily with increasing temperature (Figure 6d). The absence of a gel–sol transition in this temperature range demonstrates the presence of a transient polymer network based on supramolecular and dynamic B–N cross-links, underpinned by the thermal stability of the rapidly exchanging (free and bound) organoboranes. Over the same temperature increments, the reduction in G’ for **F2** was larger than for **F1**, again indicative of a more rapidly exchanging network for **F2**. We conclude that the more rapid cleavage of the B–N bonds, as inferred from the model systems, likely contributes to the lower modulus of the **F2** elastomer compared with **F1**.

### Mechanofluorochromic Properties

Given that the B–N dative bonds at the cross-linking sites are anticipated to be the weakest bonds in the network materials, mechanically induced deformation of the bulk material was expected to lead to preferential B–N bond scission.^107–109^ The possible scission of B–N bonds resulting in free LA and LB sites was demonstrated through computational studies (Figure S24). Furthermore, since the photophysical data described above show large differences in emission between free and complexed polymeric LA, we hypothesized that these LPs could serve as fluorogenic mechanophores, endowing the polymer networks with mechano-responsive fluorescent properties. Indeed, pronounced mechanofluorochromic effects were observed for both networks under irradiation with 365 nm UV light (Figure 7 and Figures S30–S33; see Mov_F1 and Mov_F2).

**Figure 7:**
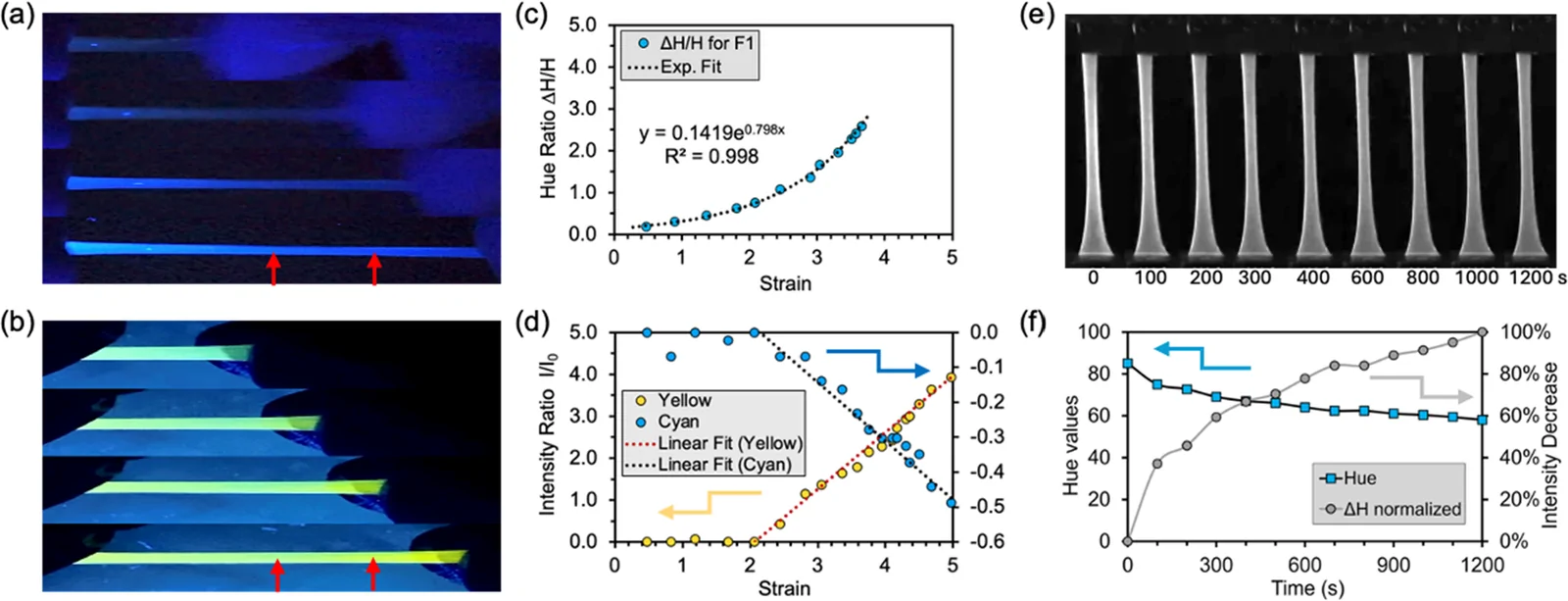
Mechanofluorochromism of **F1** and **F2** under tension. Frames from video recordings during the stretching of (a) **F1** and (b) **F2**. (c) Exponential fit of the increase in intensity of the blue emission of **F1** with strain and (d) changes in emission color composition of **F2** with strain, showing a linear fit of data above a strain threshold of ca. 200% (average of data at points indicated with red arrows in Figure 7a,b). (e) Frames from video recordings showing the emission decay of **F1** when held at 600% strain using a Black-White high-contrast Blue filter and (f) changes in Hue values during **F1** emission decay over a period of 1200 s.

For **F1**, the polymer network is only very weakly or nonemissive when LP formation is close to quantitative because of the very low oscillator strength for emission from the S_1_ state, according to TD-DFT calculations for **M1-** ^**Et**^ **Py**. However, upon B–N scission, the generated free LA sites are expected to exhibit strongly enhanced blue emission. Indeed, upon stretching the **F1** elastomer film, the emission intensity increased dramatically, resulting in a bright blue glow (Figure 7a). Upon releasing the force, the emission was quenched immediately, suggesting LP recombination and indicating good reversibility of the mechanofluorochromic process (see Mov_F1). Because of the low *T*_g_ of the PDMS segments, the majority component of the networks, rapid chain movement allows the ‘mechanically frustrated’ LPs to recombine instantly upon stress relief, rendering the material nonfluorescent again. For **F2**, the complexed polymer network is expected to be blue-emissive, with yellow emission anticipated from the free borane LA sites upon B–N bond scission. When recording the emission of **F2** while stretching the elastomer under UV irradiation, a color change from blue-green to yellow was observed (Figure 7b; see Mov_F2). We hypothesize that the initial blue-green emission of the specimen resulted from the presence of both complexed and a few uncomplexed borane moieties. The yellow component of the emission from the free LA sites in the polymer network might be enhanced by fluorescence resonance energy transfer (FRET) as the emission of the LP complex overlaps strongly with the absorption of the uncomplexed borane moiety (Figure S6). Nevertheless, a color change to yellow was clearly observed, especially at stress-concentrated sites, including both ends of the sample. Again, stress release rapidly restored the original blue-green emission of the elastomer film (see Mov_F2). The process was reversible for at least 10 cycles for polymer networks, provided the strain was well controlled, and sample breakage at large extensions was avoided.

To quantify the mechanically induced turn-on effect in **F1**, each video frame was converted to a blue-filtered image, allowing monitoring of changes in emission intensity. The strain ratio at each stage was calculated from pixel-counting sample elongation measurements. The changes in intensity levels along the polymer specimen were then analyzed at three different sites, averaged values plotted against strain, and fit to an exponential function (Figure 7c, see Figure S31 and Tables S14–S15 for individual data). The exponential emission enhancement can be rationalized by the fact that as tensile forces grow and the PDMS chain segments disentangle in the axial direction, more stress acts directly on the cross-links, thus leading to B–N bond breakage and restoring the luminescence of the free borane sites. For **F2**, the specimen was initially blue-green under no tensile load, gradually turning yellow as strain exceeded 200%. Image analysis of CMYK values at different positions on the specimens revealed that during stretching, the Magenta component remained nearly constant, while the Yellow component increased and the Cyan component decreased (Figure 7d, see Figure S33 and Table S16 for individual data). The absolute change in the values against strain at ratios beyond 200% followed approximately a linear fit. This again indicates that the segmental chain relaxation initially dissipates tensile load so that only a fraction of the B–N bonds is broken at small deformations. As the chains are extended further, the breaking of B–N bonds becomes inevitable, generating the yellow-emissive tricoordinate borane fluorophores. This strain threshold was more evident for the **F2** network, possibly because the B–N bonds are weaker, allowing the polymer segments greater mobility for energy dissipation.

Since we hypothesized that stress-induced B–N bond scission is a reversible process, stress relaxation in the soft PDMS matrix should allow for gradual recombination of the Lewis pairs even in the stretched state. To simplify the analysis process, **F1** was chosen for easier comparison of emission intensities. A video of a previously stretched sample of **F1** was recorded under UV irradiation, and frames were extracted at different time points during stress-relaxation (Figure 7e; see Mov_F1R). Analysis of the pixels (Figure 7f) showed that the decrease in absolute Hue values or emission intensity over time followed the same trend as the tensile stress-relaxation curve (see Figure 6b). These results further indicate a direct correlation between stress and emission intensity for **F1**, where a decrease in stress allows for reformation of the dative B–N bonds, changing the emission behavior back from that of the free borane LAs to the complexed Lewis pairs.

Finally, the possibility of disassembling the polymer networks by adding a competing Lewis base or Lewis acid was explored (Figures S34–S36). First, swelling tests of the obtained films were performed in toluene, THF, and DCM (Figure S34). In these moderately polar noncoordinating solvents, the polymer specimens fully swelled and maintained their shape after 2 days at ambient conditions, and viscous gels remained for over 1 week. The fact that gelation persisted even in solvents containing moisture and oxygen demonstrated the networks’ favorable stability. In contrast, when using pyridine, a coordinating but poor solvent for the polymers, little swelling occurred; the films became opaque and brittle after 1 week, indicating that a significant portion of the B–N bonds had been cleaved. Using a mixture of THF and pyridine resulted in swelling but did not fully dissolve the material, possibly due to generation of a few ‘permanent’ cross-links. Next, the addition of a competing strong Lewis acid was explored. Thin films of polymer networks **F1** and **F2** were taken in dry C_6_D_6_ and stored in valved NMR tubes for 1 week to ensure the polymer networks were fully swollen. An excess of BF_3_·OEt_2_ (two equivalents per B–N unit) was then added to each sample. The color and consistency of the swollen gels changed instantly, and after 5 days, the gels were fully disassembled (Figure 8). The disassembly process was also followed by ^11^B NMR spectroscopy, which showed that the BF_3_·OEt_2_ signal at 0.88 ppm gradually decreased, and a broad shoulder at 1.24 ppm appeared, which is assigned to the pyridine-substituted PDMS complexed by BF_3_ (Figure S36).

**Figure 8:**
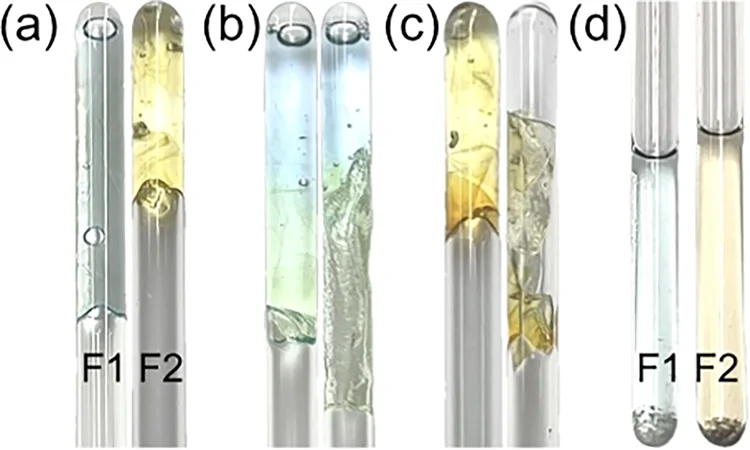
Photographs of (a) pristine swollen gels of **F1** and **F2** specimens in C_6_D_6_ after 5 days; (b) **F1** after addition of BF_3_·OEt_2_ for 5 min (left) and 20 min (right); (c) **F2** after addition of BF_3_·OEt_2_ for 5 min (left) and 20 min (right); (d) **F1** and **F2** specimens after 5 days.

## Conclusion

Mechanofluorochromic elastomers were prepared by cross-linking pyridyl-substituted PDMS with luminescent PS-based organoborane polymeric Lewis acids. Binding to pyridyl-functionalized PDMS quenched the blue fluorescence of **PS1** in the polymer network **F1** and led to a change of the yellow fluorescence of **PS2** to blue-green in **F2.** Excitingly, when the polymer films are stretched, a fluorescence turn-on can be readily observed by the naked eye under UV irradiation. We hypothesize that a significant fraction of the dynamic B–N bonds is cleaved when stretching the films, thus restoring the fluorescent ‘free’ boranes in **F1** (blue) and **F2** (yellow). Aided by the low *T*_g_ of the PDMS segments, the generated MFLPs recombine instantly upon stress relief, rendering the material nonfluorescent again. Photophysical studies on the polymers, corresponding molecular model systems, and their pyridine complexes, complemented by computations, provided in-depth insights into the excited-state properties of these materials. Tensile tests and oscillatory rheology studies consistently showed that network **F1** is relatively stronger than **F2**, in good agreement with results from UV–vis titrations and VT NMR studies on molecular model systems.

Overall, the findings reveal B–N Lewis pairs as highly tunable motifs for developing new materials with desirable mechanochromic luminescence, with potential utility across diverse fields, from real-time stress mapping to optical sensors and encryption technologies.

## Supplementary Material









## Notes

Experimental details and characterization data (PDF)
Mechanofluorochromic behavior (MOV_F1) (MOV)
Mechanofluorochromic behavior (MOV_F1R) (MOV)
Mechanofluorochromic behavior (MOV_F2) (MOV)

## †

POLYMAT and Department of Polymers and Advanced Materials, Physics, Chemistry and Technology, Faculty of Chemistry, University of the Basque Country UPV/EHU, Paseo Manuel de Lardizábal, 3, 20018 Donostia-San Sebastián, Spain
