# Supplementary material for: Visualizing B–N Bond Breaking in Silicone Elastomer Networks through Turn-On Mechanochromic Luminescence
Source: J Am Chem Soc. 2026 Oct 14;148(42):45892–903. doi: 10.1021/jacs.6c14340 (PMC13721150; doi:10.1021/jacs.6c14340)
Supplement: Supplementary file 1 [file ja6c14340_si_001.pdf]

Supporting Information

for

Visualizing B-N Bond Breaking in Silicone Elastomer Networks Through Turn-on Mechanochromic Luminescence

Beijia Chen, Fernando Vidal, and Frieder Jäkle*

Department of Chemistry, Rutgers University-Newark,
73 Warren Street, Newark, NJ 07102, USA

* Corresponding Author: Frieder Jäkle (email: fjaekle@rutgers.edu)

1. Materials and General Methods

Synthetic Procedures: Reactions involving air- and moisture-sensitive compounds were carried out in a glove box or using standard Schlenk techniques under positive pressure of pre-purified nitrogen gas. Toluene and hexanes were dried using a solvent purification system consisting of an alumina and a copper column and stored over Na-K alloy in a glovebox. Dichloromethane was passed through an alumina column, distilled from CaH_2, and degassed by the freeze-pump-thaw method; C_6D_6 and toluene-D_8 were degassed and stored over NaK; $CDCl_3$ was distilled from CaH_2 and stored over 4Å molecular sieves; 4-ethylpyridine was distilled from CaH_2. The precursors [2,2'-bithiophen]-5-yltributylstannane (**1a**), and *N*,*N*-diphenyl-4'-(trimethylstannyl)-[2,2'-bithiophen]-5-amine (**1b**), and (4-(*tert*-butyl)phenyl)-trimethylsilane (**2**) were synthesized according to previously published procedures.[1] All other reactants, reagents, and solvents were purchased from commercial sources and used without further purification.

NMR Spectroscopy: 500 MHz ^{1}H, 160.4 MHz ^{11}B, and 125.8 MHz $^{13}C[^{1}H]$ NMR data were recorded at ambient temperature on a 500 MHz Bruker AVANCE spectrometer. 160.4 MHz ^{11}B NMR spectra were acquired with boron-free quartz NMR tubes using a boron-free 5mm PH SEX 500S1 11B-H/F-D probe. ^{1}H and $^{13}C[^{1}H]$ NMR spectra were referenced internally to solvent signals ($CDCl_3$: 7.26 ppm for ^{1}H NMR, 77.16 ppm for ^{13}C NMR; C_6D_6: 7.16 ppm for ^{1}H, 128.06 ppm for ^{13}C NMR; toluene-D_8: 7.01 ppm for ^{1}H NMR. ^{11}B NMR signals were referenced externally to $BF_3{\cdot}OEt_2$. Chemical shift multiplicities are reported as singlet (s), doublet (d), multiplet (m), and broad (br), and the abbreviations Py (pyridine), Ph (phenyl), ^{t}Bu (*tert*-butyl), Et (ethyl), BTh (2,2'-bithiophene), and PS (polystyrene) are used for signal assignments.

High-Resolution Mass Spectroscopy: High-resolution mass spectrometry (HRMS) data of solutions of the boranes in a mixture of toluene and acetonitrile, with added Bu_4NCN, were obtained on an Orbitrap Exploris 240 instrument in negative ion mode using electrospray ionization (ESI).

Photophysical Studies: UV-vis absorption data were acquired on a Varian Cary 5000 UV-Vis/NIR spectrophotometer. The fluorescence spectra were recorded using a Horiba Fluorolog-3 spectrofluorometer with a FluoroHub R-928 detector. As specified, a 350 nm, 390 nm, or 450 nm nanoLED was used for fluorescence lifetime measurements. The same instrument equipped with a pre-calibrated Quanta-φ integrating sphere was used for absolute fluorescence quantum yield measurements. For weakly emitting borane Lewis acid-base complexes, a 4-curve method was applied using Thorlabs neutral-density filters.

Polymer Characterization. GPC analyses were performed in THF (1 mL/min) using a Waters Alliance e2695 system equipped with a 717plus autosampler, a 2414 refractive index detector, and a 2998 photodiode array (PDA) detector. For separation, the samples were passed through a series of styragel columns maintained in a column heater at 30 °C (Polymer Laboratories, PLgel 5 μm guard, PLgel 5 μm MIXED-C, and PLgel 5 μm MIXED-D columns). The columns were calibrated against 10 narrow polystyrene standards (Agilent Technologies) in the molecular weight range of 580 to 371,100 Da. Instrument operation and data analysis were performed using Empower software.

Rheology Measurements. Oscillatory shear measurements were performed on a Discovery HR-2 rheometer (TA Instruments) using stainless-steel 8 mm parallel plates. Axial force was maintained at approximately 2.0 N while increasing the geometry gap to 90 μm to ensure full contact between the elastic polymer film and the plates. Oscillatory frequency sweeps (1% strain, frequency at 100-0.01 rad/s) were performed at 25 °C after 10 min of equilibration to

remove the thermomechanical history of the bulk material. Dynamic temperature ramp tests were run at a ramp rate of 1 °C/min from the loading temperature of 0 °C to 160 °C (cooling cycle from 160 °C to 0 °C) with a strain of 1% and a frequency of 1.0 rad/s. Samples were equilibrated at 0 °C for 10 minutes before any heating-cooling cycles. Samples were held either at 0 °C or 160 °C for 5 minutes between each heating/cooling scan. Data analysis was performed using the TRIOS Software (TA Instruments).

Time-Temperature Superposition (TTS). Samples were loaded onto a Discovery HR-2 rheometer (TA Instruments) and analyzed by oscillatory shear measurements using stainless steel 8 mm parallel plates, as described above. Consecutive oscillatory frequency sweeps (100-0.01 rad/s at 1% strain) were performed at 5 °C intervals with 5 min equilibration time between runs. Samples were first tested at the lowest temperature (55 °C), and the temperature was then incrementally increased to 120 °C during each experiment. The data were combined into the corresponding master curves using the TRIOS Software (TA Instruments), applying the Williams-Landel-Ferry (WLF) equation to generate Time-Temperature Superposition plots with a reference temperature of 65 °C.

Thermogravimetric Analysis (TGA) was performed on a Discovery TGA (TA Instruments). Samples were placed on Platinum HT pans and heated at 10 °C/min to 1000 °C under N_2 flow (60 mL/min).

Differential Scanning Calorimetry (DSC) was performed on a Discovery DSC (TA Instruments). Samples were loaded at 25 °C and heated to 130 °C (first heating scan to remove thermal history), then cooled down to 0 °C (first cooling scan), heated to 130 °C, and cooled to 0 °C again (second heating-cooling cycle) at a ramp rate of 10 °C/min.

Mechanical Testing. Rectangular pieces (approximately 5.0 mm × 3.0 mm × 0.2 mm) of the polymer network films were mounted on a DMA Q800 (TA Instruments) equipped with a film-tension clamp. Tests were performed after calibrating at 25 °C for 10 minutes. Stress-strain curves were recorded using a force ramp and a strain ramp. Adjusted true stress and strain were calculated with an estimated Poisson's ratio of 0.45. Force ramps were performed at 1 N/min until the specimen had reached the maximum length of the apparatus (~28 mm). Strain ramps were performed at 60% increments per minute until the specimen had reached the maximum length of the apparatus. Stress-relaxation curves were recorded by holding the specimen at 300% strain over 10 minutes, and the corresponding stress values were recorded. Creep tests were performed by holding the specimen at 0.1 N and recording the strain values over 20 minutes.

Density Functional Theory (DFT). DFT calculations were performed with the Gaussian16 software package. The geometry optimizations and frequency calculations were performed using the B3LYP functional with the 6-31G(d) basis set. Time-dependent DFT (TD-DFT) calculations were performed using the cam-B3LYP functional with the 6-31G(d) basis set for all atoms. B-N bond breakage was simulated with a scan optimization of atom distances with a restricted 6-31G(d) basis set. Redundant coordinates were set on atoms in para-position to B or N on the benzene and pyridine ring, respectively. 15 steps of 0.1 Å increments between the anchor atoms were taken to deform the molecule, and the geometry was optimized at each step.

2. Synthesis of Molecular Model Compounds

Synthesis of Bis([2,2'-bithiophen]-5-yl)(4-(*tert*-butyl)phenyl)borane (M1)

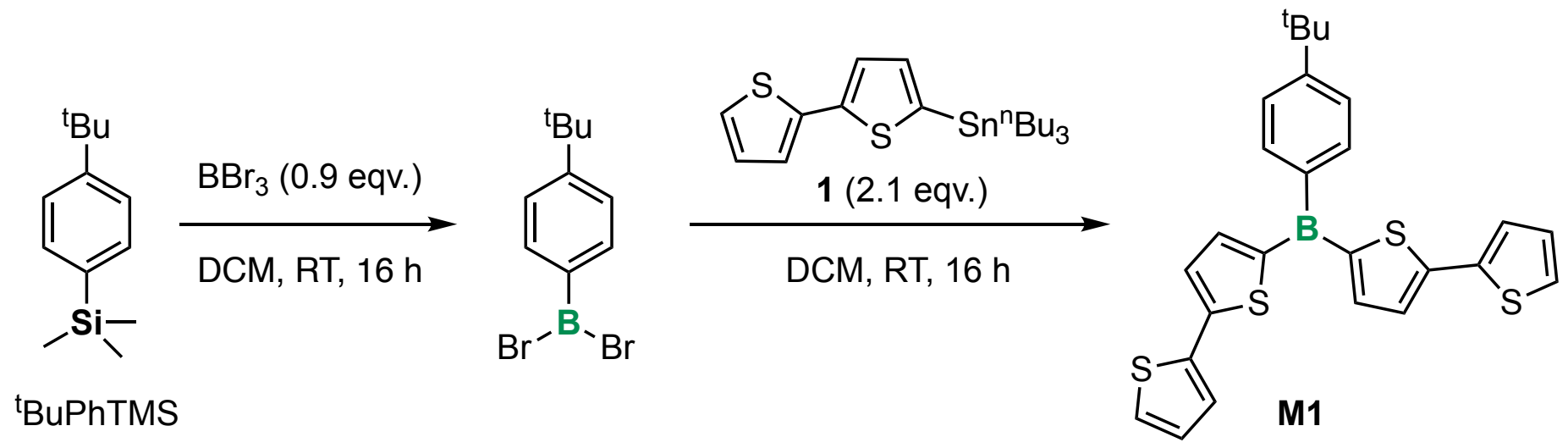

Inside a N_2-filled glovebox, (4-(*tert*-butyl)phenyl)trimethylsilane (1.032 g, 5.00 mmol) was dissolved in 3.0 mL of anhydrous CH_2Cl_2 in a screw-cap pressure tube. BBr_3 (1.127 g, 4.50 mmol) in 2.0 mL of anhydrous CH_2Cl_2 was slowly added dropwise at room temperature, and the resulting light brown solution was allowed to stir for 16 h. At this point, analysis by ^{1}H and ^{11}B NMR in C_6D_6 of the crude reaction mixture showed full consumption of BBr_3 (loss of sharp signal at 39 ppm in the ^{11}B NMR spectrum). A solution of **1** (4.782 g, 10.5 mmol) in 3.0 mL of CH_2Cl_2 was added dropwise to the reaction mixture. The bright yellow solution was stirred at room temperature for 16 hours. The reaction mixture was precipitated into 40 mL of hexanes and stored inside a glovebox freezer at -20 °C. The solids were collected on a fine-fritted funnel, washed with cold hexanes (3 × 10 mL), and dried under high vacuum to give **M1** as a yellow powder. Yield 1.40 g (66%).

^{1}H NMR (C_6D_6, 25 °C, 500 MHz): δ 7.93 (d, J = 7.9 Hz, 2H, ^{t}BuPh), 7.74 (d, J = 3.7 Hz, 2H, BTh), 7.46 (d, J = 8.1 Hz, 2H, ^{t}BuPh), 7.23 (d, J = 3.7 Hz, 2H, BTh), 7.07 (d, J = 3.6 Hz, 2H, BTh), 6.72 (d, J = 5.1 Hz, 2H, BTh), 6.62 (m, 2H, BTh), 1.28 (s, 9H, ^{t}Bu).

^{13}C NMR (C_6D_6, 25 °C, 125.8 MHz): δ 153.6, 149.0, 144.3 (br, B-C), 143.4 (BTh), 140.5 (br, B-C), 137.2, 136.7 (^{t}BuPh), 127.9 (BTh, overlapped), 125.6 (BTh), 125.5 (BTh), 125.0 (BTh), 124.5 (^{t}BuPh), 35.0 (^{t}Bu), 31.4 (^{t}Bu).

^{11}B NMR (C_6D_6, 25 °C, 160.4 MHz): δ 52.2 ($w_{1/2}$ = 1800 Hz).

HRMS of CN^- ion complex (ESI, negative ion mode): m/z = 500.0805 ([**M1**+CN]$^-$, calculated for $C_{27}H_{23}BNS_4$ 500.0812).

Synthesis of 4-Ethylpyridine Complex of M1 (M1-^{Et}Py)

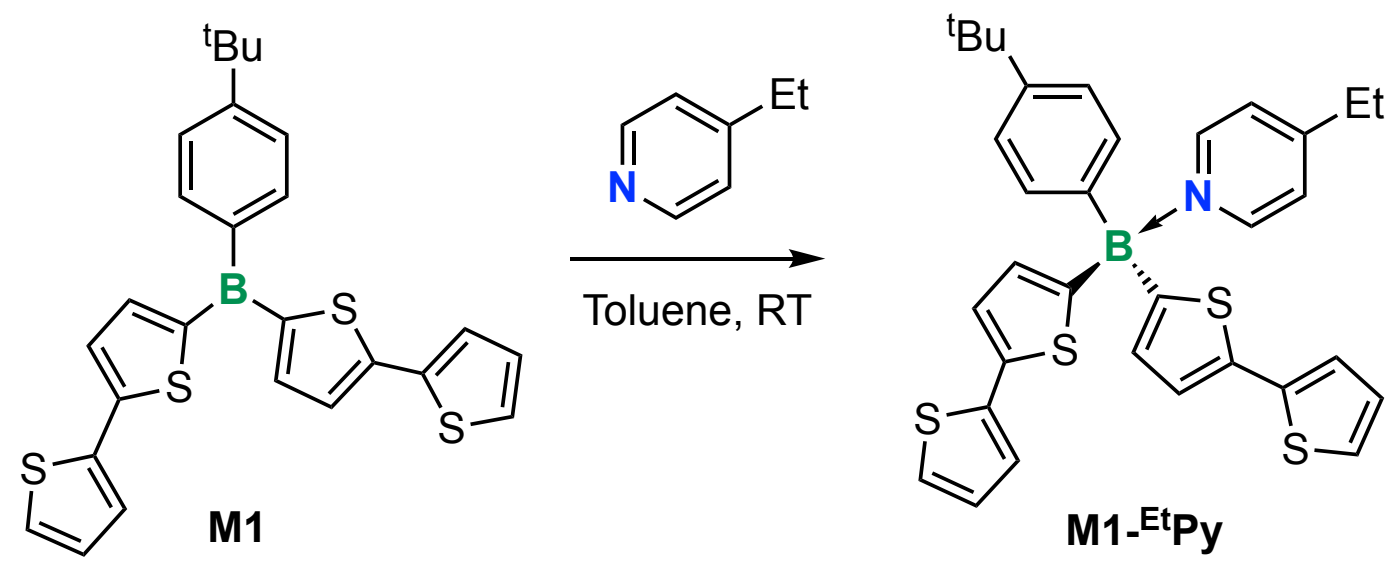

Inside a N_2-filled glovebox, **M1** (95.2 mg, 0.20 mmol) was dissolved in 2.0 mL of anhydrous toluene. Then, 4-ethylpyridine (25.7 mg, 0.24 mmol, 1.2 eq) was added neat via syringe at room temperature. The reaction mixture was precipitated into cold anhydrous hexanes, and a white suspension formed immediately. The mixture was kept at -20 °C for 24 hours to facilitate further crystallization. The solids were collected on a fritted funnel, washed with cold hexanes (3 × 5 mL), and dried under high vacuum to give **M1-^{Et}Py** as a white powder. Yield 82.1 mg (71%).

^{1}H NMR (C_6D_6, 25 °C, 500 MHz): δ 8.32 (d, J = 5.5 Hz, 2H, Py), 7.55 (d, J = 8.0 Hz, 2H, ^{t}BuPh), 7.46 (d, J = 7.5 Hz, 2H, ^{t}BuPh), 7.35 (d, J = 3.5 Hz, 2H, BTh), 7.23 (d, J = 3.5 Hz, 2H, BTh), 7.05 (d, J = 4.0 Hz, 2H, BTh), 6.69 (d, J = 5.0 Hz, 2H, BTh), 6.65 (m, 2H, BTh), 6.13 (br, 2H, Py), 1.81 (br, 2H, Et), 1.35 (s, 9H, ^{t}Bu), 0.60 (br, 3H, Et).

^{13}C NMR (C_6D_6, 25 °C, 125.8 MHz): δ 158.5, 155.5 (br, B-C), 148.8, 148.3 (br, B-C), 146.9 (o-Py), 139.6, 139.3, 133.9 (^{t}BuPh), 133.6 (BTh), 127.9 (overlapped, BTh), 125.0 (BTh), 124.7 (^{t}BuPh), 124.1 (m-Py), 123.6 (BTh), 123.3 (BTh), 34.5 (^{t}Bu), 31.7 (^{t}Bu), 27.8 (Et), 12.8 (Et).

^{11}B NMR (C_6D_6, 25 °C, 160.4 MHz): δ 0.6 ($w_{1/2}$ = 400 Hz).

Synthesis of Bis(5'-(*N,N*-diphenylamino)-[2,2'-bithiophen]-5-yl)(4-(*tert*-butyl)phenyl)borane (M2)

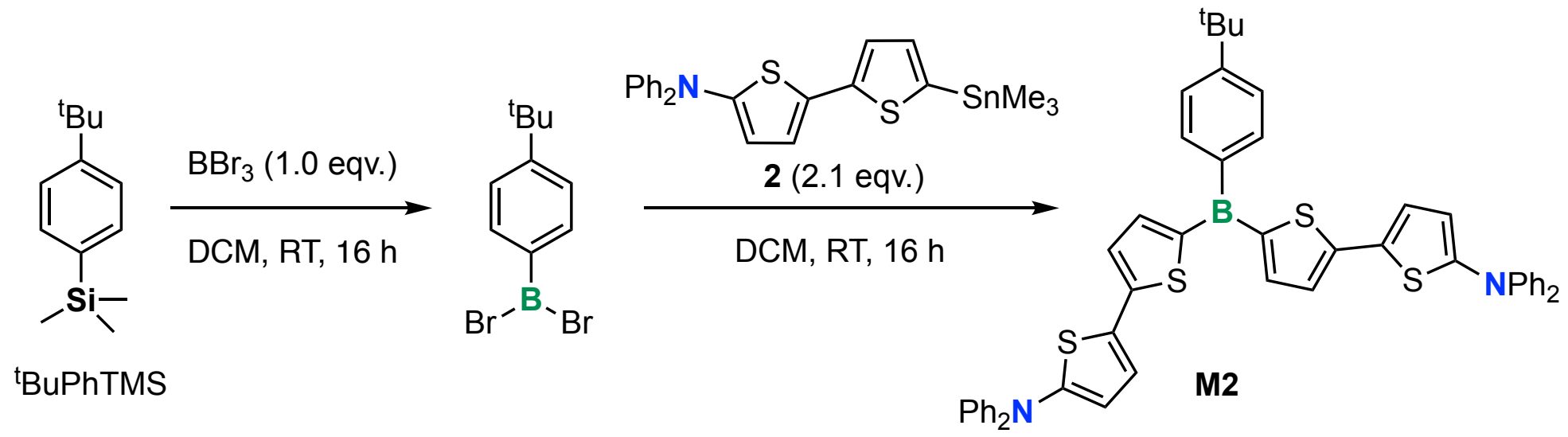

Inside a N_2-filled glovebox, (4-(*tert*-butyl)phenyl)trimethylsilane (213.0 mg, 1.03 mmol) was dissolved in 5.0 mL of anhydrous CH_2Cl_2. BBr_3 (250.0 mg, 1.00 mmol) was slowly added dropwise by syringe at room temperature, and the resulting light-brown solution was stirred for 16 hours. At this point, analysis by ^{1}H and ^{11}B NMR in C_6D_6 of the crude reaction mixture showed clean, full conversion to the dibromo(4-(*tert*-butyl)phenyl)borane intermediate (a broad signal at 56.6 ppm in the ^{11}B NMR spectrum). The solvent was evaporated under high vacuum, and the residue was redissolved in fresh CH_2Cl_2 and concentrated again to ensure complete removal of Me_3SiBr and any excess of BBr_3. The brown residue was then dissolved again in 2 mL of anhydrous CH_2Cl_2, and **2** (1.0728 g, 2.17 mol) in 5.0 mL of CH_2Cl_2 was added to the solution. The dark orange solution was stirred at room temperature overnight. The reaction mixture was then concentrated to about 2 mL and precipitated into 30 mL of cold hexanes (ca. -20 ºC). The suspension was placed inside a glovebox freezer at -20 °C. A precipitate formed, which was collected on a fritted funnel, washed with cold hexanes (3 x 2 mL, -20 °C). For further purification, the solid was taken up in hot hexanes and recrystallized at -30 °C. The solids were collected and then dried under high vacuum to give **M2** as a dark orange powder. Yield: 10 mg (ca. 1%); the low yield is due to difficulties in the purification of the compound.

^{1}H NMR (C_6D_6, 25 °C, 500 MHz): δ 7.90 (d, J = 7.5 Hz, 2H, ^{t}BuPh), 7.72 (d, J = 3.5 Hz, 2H, BTh), 7.45 (d, J = 8.0 Hz, 2H, ^{t}BuPh), 7.13-7.11 (m, 10H, BTh & o-NPh_2), 7.03 (t, J = 7.5 Hz, 8H, m-NPh_2), 6.88 (d, J = 4.0 Hz, 2H, BTh), 6.85 (t, J = 7.3 Hz, 4H, p-NPh_2), 6.72 (d, J = 4.0 Hz, 2H, BTh), 1.28 (s, 9H, ^{t}Bu).

^{13}C NMR (C_6D_6, 25 °C, 125.8 MHz): δ 153.6, 152.9, 150.0, 148.1, 144.1 (br, B-C), 143.9 (BTh), 141.2 (br, B-C), 136.9, 130.7, 129.7 (m-NPh_2), 125.1 (BTh), 124.8 (^{t}BuPh), 124.4 (BTh), 123.8 (p-NPh_2), 123.6 (o-NPh_2), 120.7 (BTh), 34.9 (^{t}Bu), 31.4 (^{t}BuPh).

^{11}B NMR (C_6D_6, 25 °C, 160.4 MHz): δ 51.7 ($w_{1/2}$ = 4000 Hz).

HRMS of CN^- ion complex (ESI, negative ion mode): m/z = 834.2275 ($[M\mathbf{2}+CN]^-$, calculated for $C_{51}H_{41}BN_3S_4$ 834.2282).

Synthesis of 4-Ethylpyridine Complex of M2 (M2-^{Et}Py)

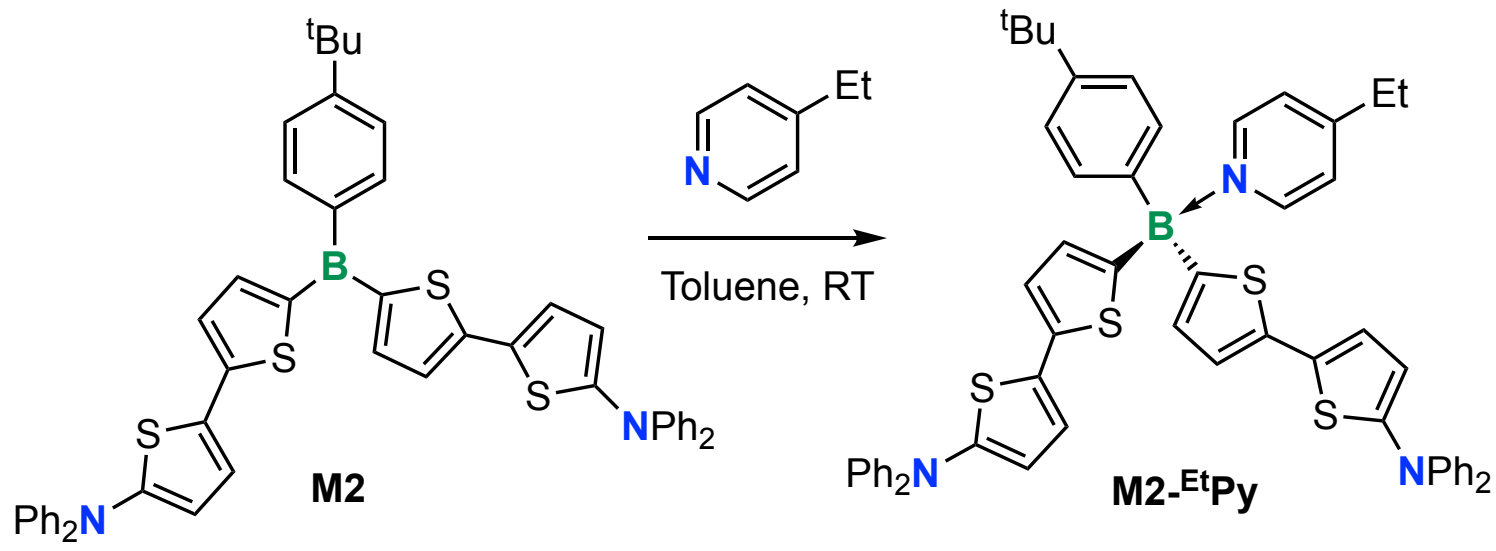

Inside a N_2-filled glovebox, to a solution of a crude sample of **M2** (274.2 mg, 0.339 mmol) in 2.0 mL toluene was added 4-ethylpyridine (43.8 mg, 0.409 mmol, 1.2 eqvs.) via syringe. A light-yellow suspension formed immediately. The suspension was kept at -20 °C for 24 hours. A precipitate formed, which was collected on a fritted funnel, washed with hexanes (3 × 5.0 mL), and dried under high vacuum to give **M2-^{Et}Py** as a yellow powder. Yield 253.6 mg, 82% from **M2**.

^{1}H NMR (C_6D_6, 25 °C, 500 MHz): δ 8.31 (d, J = 6.0 Hz, 2H, Py), 7.54 (d, J = 8.0 Hz, 2H, ^{t}BuPh), 7.46 (d, J = 8.0 Hz, 2H, ^{t}BuPh), 7.23 (d, J = 3.0 Hz, 2H, BTh), 7.18 (d, J = 3.0 Hz, 2H, BTh), 7.14 (d, J = 8.0 Hz, 8H, o-NPh_2), 7.01 (t, J = 7.8 Hz, 8H, m-NPh_2), 6.85 (d, J = 4.5 Hz, 2H, BTh), 6.82 (t, J = 7.5 Hz, 4H, p-Ph), 6.38 (d, J = 3.5 Hz, 2H, BTh), 6.15 (br d, J = 5.0 Hz, 2H, Py), 1.81 (q, J = 7.5 Hz, 2H, Et), 1.34 (s, 9H, ^{t}Bu), 0.59 (t, J = 7.5 Hz, 3H, Et).

^{13}C NMR (C_6D_6, 25 °C, 125.8 MHz): δ 155.2 (br, B-C), 149.8 (i-BTh), 148.7 (i-Ph/Py), 148.4 (i-NPh_2), 146.9 (br, o-Py), 140.1 (i-BTh), 134.1 (i-BTh), 133.9 (^{t}BuPh), 133.6 (BTh), 129.5 (m-NPh_2), 124.7 (^{t}BuPh), 124.3 (BTh), 124.1 (br, m-Py), 123.1 (p-NPh_2), 123.0 (o-NPh_2), 122.4 (BTh), 121.8 (BTh), 34.5 (^{t}Bu), 31.7 (^{t}Bu), 27.8 (Py-Et), 12.9 (Py-Et), B-C(Ph) not observed.

^{11}B NMR (C_6D_6, 25 °C, 160.4 MHz): δ 0.9 ($w_{1/2}$ = 650 Hz).

3. Synthesis and Characterization of Linear Polystyrene Copolymers

3.1 Polymer Functionalization

The synthesis of the silylated polystyrene copolymer precursor (**P(St-*co*-StSiMe$_3$)** was previously reported.[2] GPC *vs* PS standards (THF, 1 mL min^{-1}): M_n = 24,449 Da, M_w = 27,510 Da, $Đ = M_w/M_n$ = 1.13, DP_n = 218. Ratio of incorporated monomers of n/m = 8:1 based on ^{1}H NMR analysis and n = 194, m = 24.3 based on GPC analysis (Figure S1).

Synthesis of P(St-*co*-StB(BTh)$_2$) (PS1)

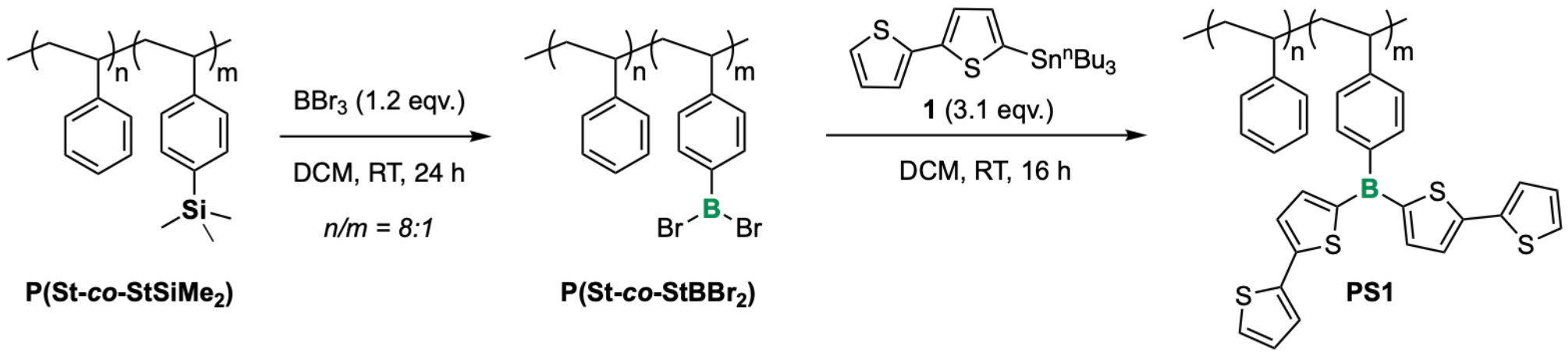

Inside a N_2 glovebox, a solution of BBr_3 (150.2 mg, 0.600 mmol, 1.2 eqvs.) in 2.0 mL of anhydrous CH_2Cl_2 was added dropwise at room temperature to a solution of **P(St-*co*-StSiMe$_3$)** (497.5 mg, 0.493 mmol of $SiMe_3$ groups) in 4.0 mL anhydrous CH_2Cl_2. The mixture was then stirred at room temperature for 24 hours. After confirming quantitative replacement of silyl

groups by ^{1}H NMR analysis (disappearance of $SiMe_3$ signal), a solution of Bu_3SnBTh (**1**, 683 mg, 1.52 mmol, 3.1 eqvs.) in 2.0 mL anhydrous CH_2Cl_2 was added dropwise to the mixture. The reaction mixture was further stirred at room temperature overnight. The polymer **PS1** was isolated by repeated precipitation from toluene into hexanes and obtained as a yellow powder. Yield 455.2 mg, 72% from **P(St-*co*-StSiMe$_3$)**.

^{1}H NMR (C_6D_6, 25 °C, 500 MHz): δ 7.76 (br, 2H, ^{B}Ph), 7.5-6.4 (m, Ph & ^{B}Ph & BTh), 2.5-1.4 (CH_2-CH backbone).

^{13}C NMR (C_6D_6, 25 °C, 125.8 MHz): δ 149.3, 147-145 (overlapped, PS), 144.7, 143.9, 141.1 (br, B-C), 137.7, 137.6, 137.2, 129.3, 126.2, 126.0, 125.7, 125.5, 47.5-42.0 (backbone C-H), 41.1 (backbone CH_2).

^{11}B NMR (C_6D_6, 25 °C, 160.4 MHz): δ 46.8 ($w_{1/2}$ = 4000 Hz).

GPC analysis was performed on the 4-ethylpyridine complex, which shows better stability under ambient conditions than the free Lewis acid-functionalized polymer, *vs* PS standards (THF, 1 mL min^{-1}): M_n = 21,009, M_w = 24,981, $Đ = M_w/M_n$ = 1.19, DP_n = 139. Ratio of incorporated monomers of n/m = 8:1 based on ^{1}H NMR analysis; n = 120, m = 15.0 based on GPC analysis, assuming that 4-ethylpyridine is bound to boron according to GPC-PDA data (Figures S1). The slight decrease in molecular weight relative to the silylated precursor polymer may be due to the loss of less-soluble higher-molecular-weight material during reprecipitation.

Complexation of PS1 with 4-Ethylpyridine (PS1-^{Et}Py)

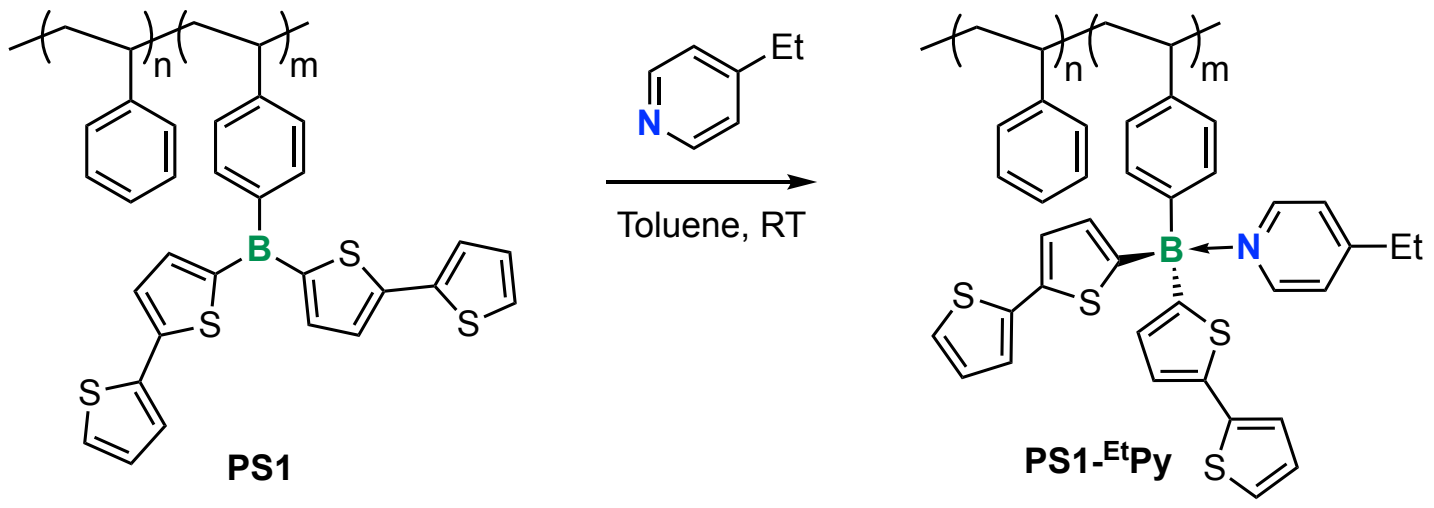

Inside a N_2 glovebox, to a solution of **PS1** (103.1 mg, 0.0807 mmol of boron functional groups) in 1.0 mL of anhydrous toluene were added 10.0 µL of 4-ethylpyridine (9.4 mg, 0.088 mmol) by syringe. The polymer solution was precipitated into 5.0 mL of anhydrous hexanes and the solids collected by centrifugation. The solids were washed with fresh hexanes and then dried under high vacuum to give **PS1-^{Et}Py** as a white powder. Yield 89.7 mg, 79%. ^{1}H NMR integration confirmed the repeat unit ratio of approximately n/m = 8:1.

^{1}H NMR (C_6D_6, 25 °C, 500 MHz): δ 8.33 (br, 2H, Py), 7.5-6.5 (m, St & ^{B}Ph & BTh), 6.26 (br, 2H, Py), 2.5-1.4 (m, 30H, CH_2-CH backbone & Py-CH_2), 0.66 (br, 3H, CH_3).

^{11}B NMR (C_6D_6, 25 °C, 160.4 MHz): δ -1.1 ($w_{1/2}$ = 900 Hz).

Synthesis of P(St-*co*-StB(BThNPh$_2$)$_2$) (PS2)

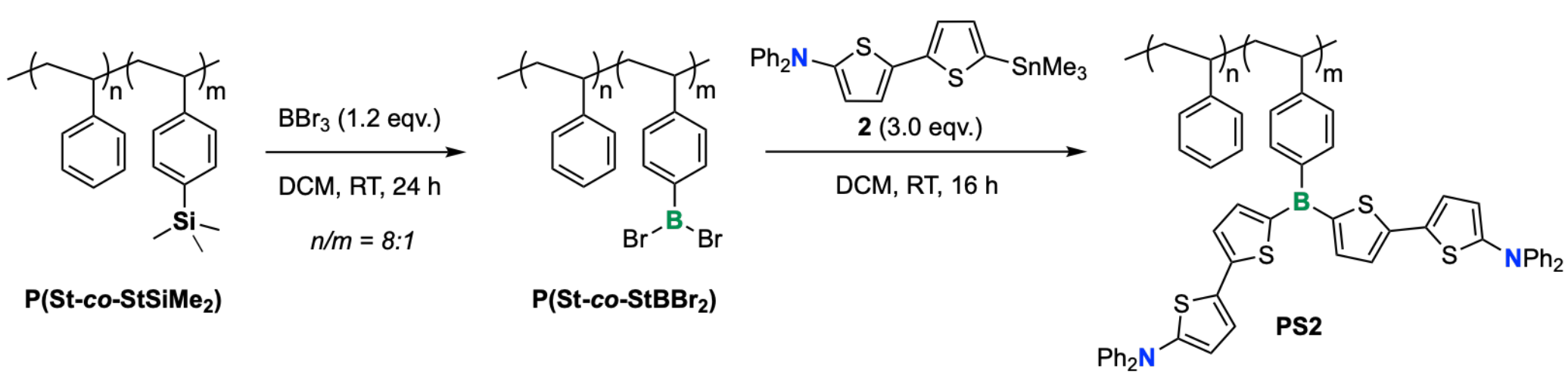

Inside a N_2 glovebox, a solution of BBr_3 (150.5 mg, 0.600 mmol, 1.2 eqvs.) in 2.0 mL of anhydrous CH_2Cl_2 was added dropwise at room temperature to a solution of **P(St-*co*-StSiMe$_3$)** (502.3 mg, 0.498 mmol of $SiMe_3$ groups) in 4.0 mL anhydrous CH_2Cl_2. The mixture was then stirred at room temperature for 24 hours. After confirming quantitative replacement of silyl groups by ^1H NMR analysis, a solution of $Me_3SnBThNPh_2$ (**2**, 742.3 mg, 1.50 mmol, 3.0 eqvs.) in 2.0 mL anhydrous CH_2Cl_2 was added dropwise to the mixture. The reaction mixture was further stirred at room temperature overnight. The polymer **PS2** was isolated by repeated precipitation from toluene into hexanes and obtained as a dark orange powder. Yield 336.5 mg, 42% from **P(St-*co*-StSiMe$_3$)**.

^1H NMR (C_6D_6, 25 °C, 500 MHz): δ 7.72 (br, 4H, ^{B}Ph & BTh), 7.5-6.4 (m, St & NPh_2 & ^{B}Ph & BTh), 6.36 (br, 2H, BTh), 2.5-1.4 (m, 29H, CH_2-CH backbone).

^{13}C NMR (C_6D_6, 25 °C, 125.8 MHz): δ 148.1, 147-145 (PS), 138.5, 129.7 (m-NPh_2), 129.6, 123.9 (p-NPh_2), 123.6 (o-NPh_2), 123.5 (BTh), 123.1, 120.9 (BTh), 47.5-42.0 (backbone C-H observed in HSQC), 41.1 (backbone CH_2).

^{11}B NMR (C_6D_6, 25 °C, 160.4 MHz): δ 42.6 ($w_{1/2}$ = 3200 Hz).

GPC analysis was performed on the 4-ethylpyridine complex, which shows better stability under ambient conditions than the free Lewis acid-functionalized polymer, *vs* PS standards (THF, 1 mL min^{-1}): M_n = 23,028 Da, M_w = 28,812 Da, $Đ = M_w/M_n$ = 1.25, DP_n = 123. Ratio of incorporated monomers of n/m = 8:1 based on ^1H NMR analysis; n = 107, m = 13.4 based on GPC analysis, assuming that 4-ethylpyridine is bound to boron according to GPC-PDA data (Figure S1). The slight decrease in molecular weight relative to the silylated precursor polymer may be due to the loss of less-soluble, higher-molecular-weight material during reprecipitation.

Complexation of PS2 with 4-Ethylpyridine (PS2-^{Et}Py)

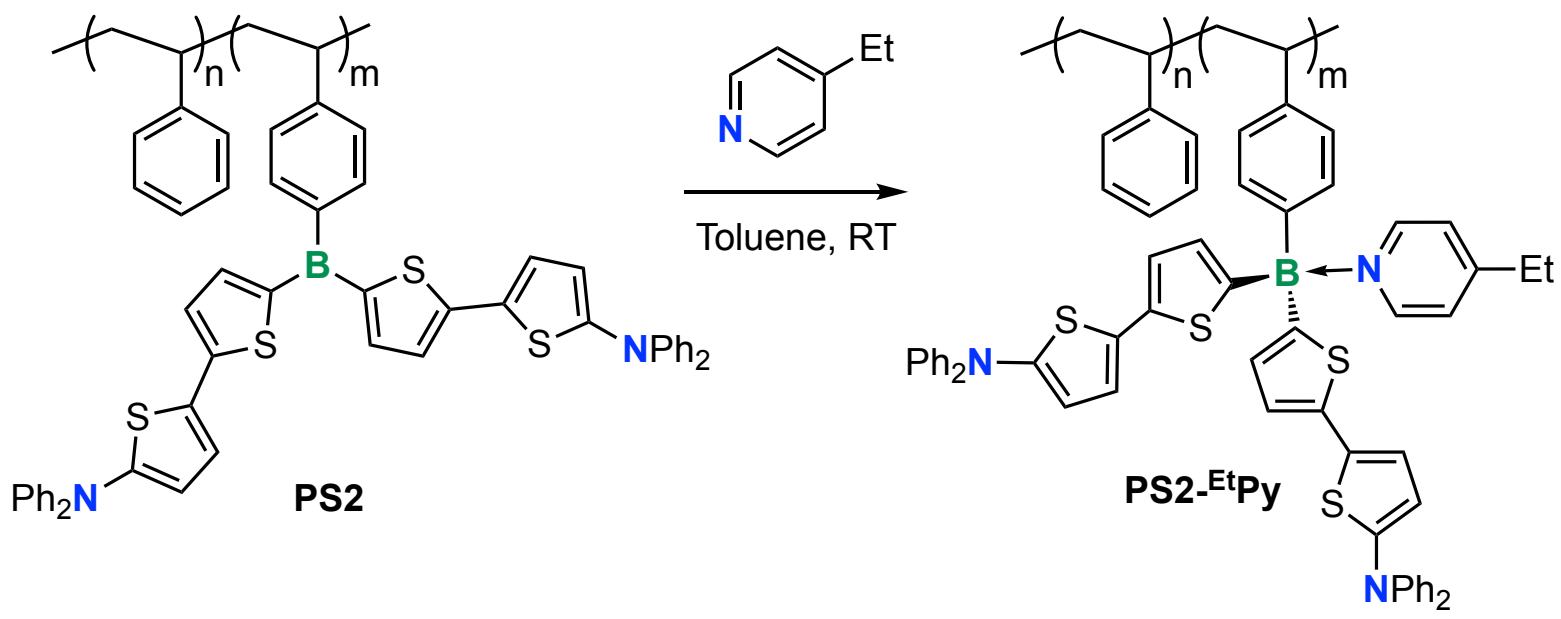

Inside a N_2 glovebox, to a solution of **PS2** (82.5 mg, 0.0511 mmol of boron functional groups) in 1.0 mL of anhydrous toluene, 8.0 µL of 4-ethylpyridine (7.5 mg, 0.070 mmol) were added by syringe. The polymer solution was precipitated into 5.0 mL of anhydrous hexanes and the solids collected by centrifugation. The solids were washed with fresh hexanes and dried under high vacuum to give **PS2-^{Et}Py** as a pale-yellow powder. Yield 68.9 mg, 78%. ^1H NMR integration confirmed the repeat unit ratio of approximately n/m = 8:1.

^1H NMR (C_6D_6, 25 °C, 500 MHz): δ 8.30 (br, 2H, Py), 7.5-6.6 (m, St & NPh_2 & ^{B}Ph & BTh), 6.5-6.2 (br, 4H, BTh & Py), 2.5-1.4 (m, 28H, CH_2-CH backbone and Py-CH_2), 0.66 (br, 3H, CH_3).

^{11}B NMR (C_6D_6, 25 °C, 160.4 MHz): δ 2.3 ($w_{1/2}$ = 1600 Hz).

3.2 Polymer Characterization

Molecular weight determination of linear copolymer Lewis pair complexes

GPC-RI analyses were performed on the silylated precursor polymer and both borane polymers in THF; an excess of 4-ethylpyridine (giving rise to a large solvent peak at a retention time of ca. 21 minutes) was added to the borane polymer solutions to ensure complexation and enhance polymer stability during analysis. The absorption spectra of the polymer complexes were verified by photodiode array (PDA) detection, showing absorption features consistent with pyridine-complexed polymers (slight shifts relative to the reported UV-vis data for the isolated polymers are attributed to the different solvent (THF with ca. 5% added 4-ethylpyridine *versus* toluene with an excess of ethylpyridine).

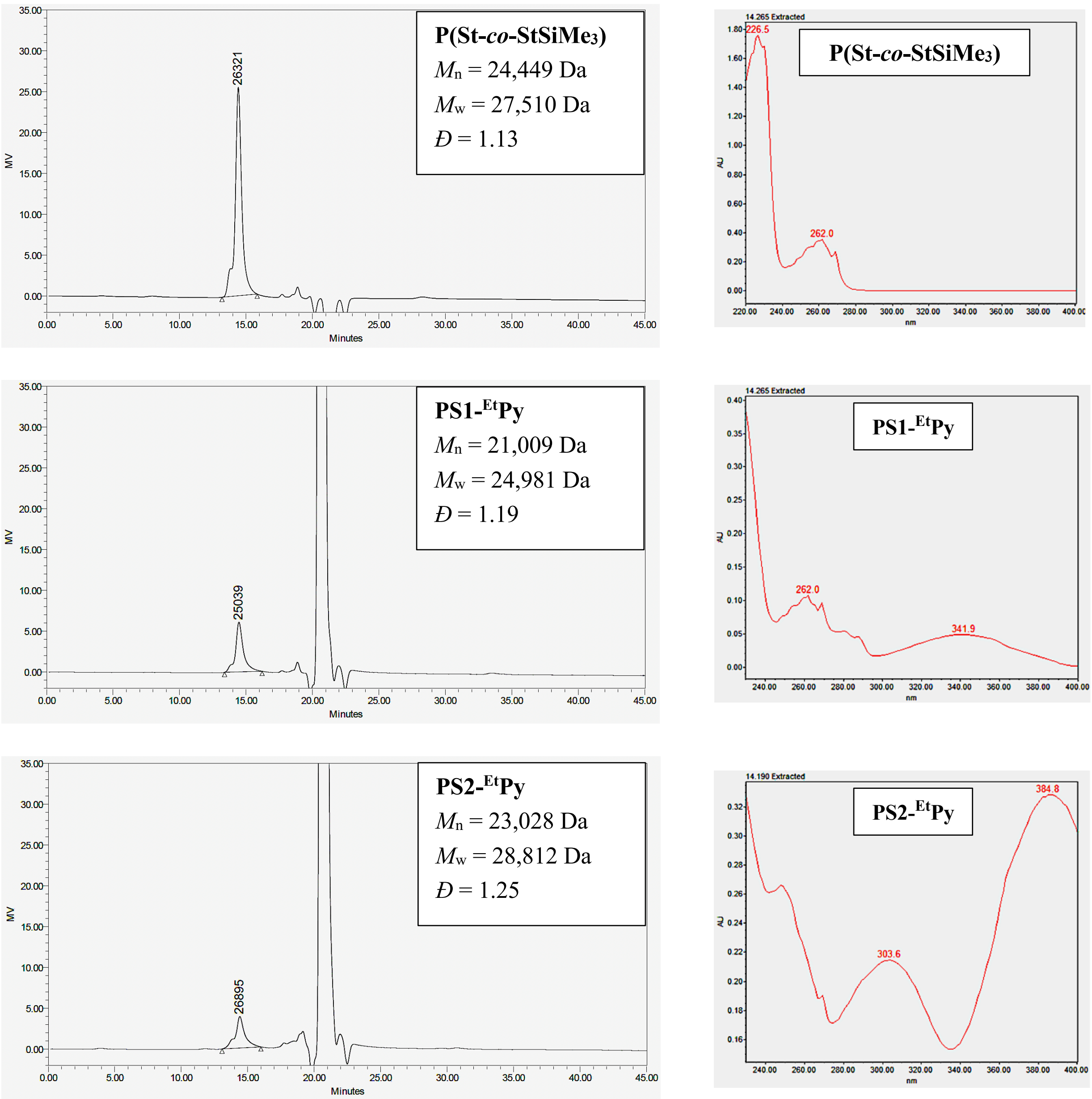

Figure S1. GPC-RI traces (THF with added 4-ethylpyridine, 1 mL min^{-1}) and UV-vis spectra from PDA detection for (top) PSTMS, (middle) **PS1-^{Et}Py**, and (bottom) **PS2-^{Et}Py**.

DSC and TGA analyses of linear copolymer Lewis pair complexes

The glass transition temperatures and thermal stability of the pyridine-complexed copolymers were assessed.

Thermogravimetric analyses (TGA) were performed by heating samples of the 4-ethylpyridine-complexed copolymers from 30 °C to 700 °C at a heating rate of 10 °C min^{-1} (Figure S2). Decomposition temperatures (T_d) are reported as the onset of decomposition in wt% vs. temperature plots. The onset of Lewis base loss was also determined, again as the onset of weight loss in the wt% vs. temperature plots.

**Polymer**	**PS1-^{Et}Py**	**PS2-^{Et}Py**
Wt% ^{Et}Py	7.7	6.2
Wt% Ar	11.8	19.3
Wt% Ar_2B	24.4	39.3
**Wt% Ar_2B-^{Et}Py**	**32.2**	**45.5**

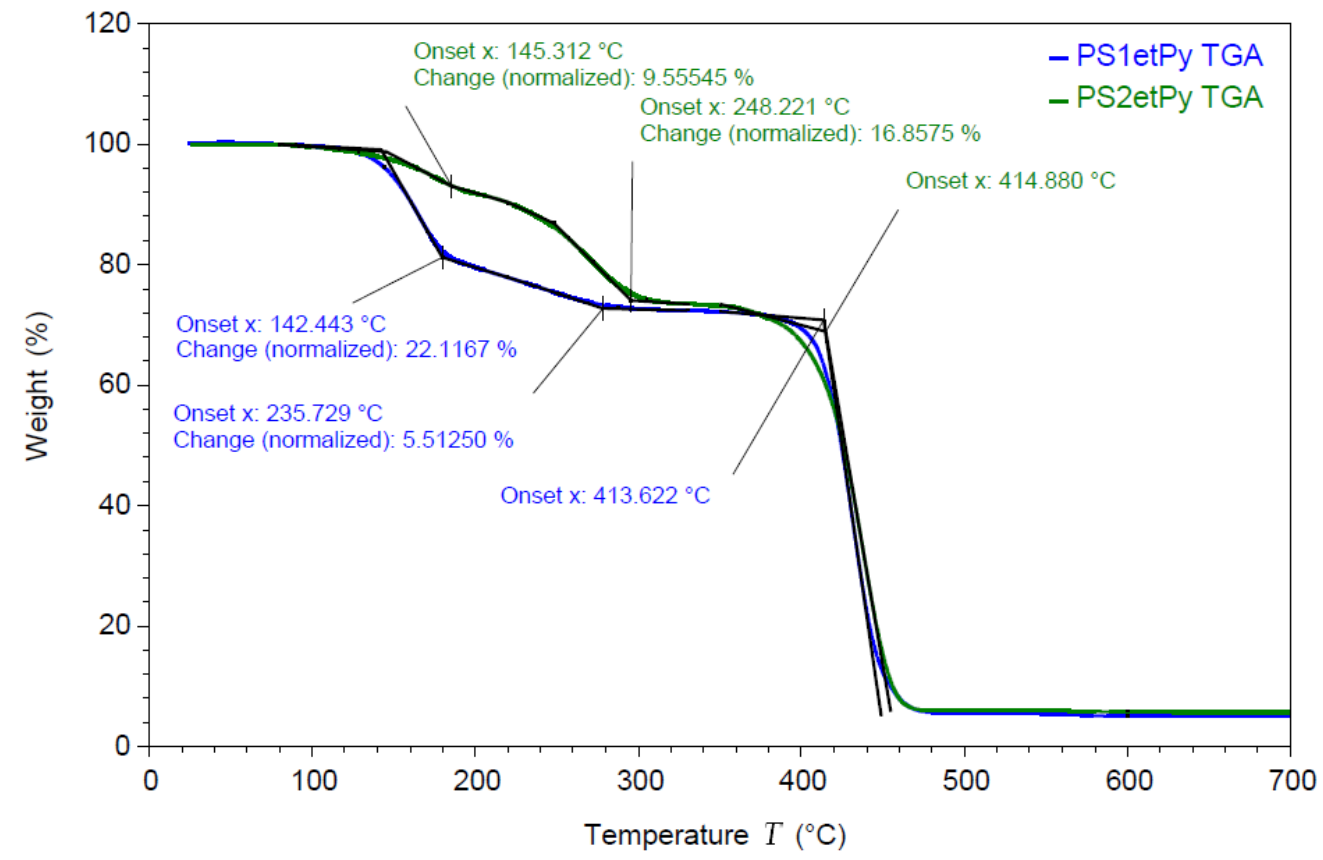

Figure S2. TGA traces for **PS1-^{Et}Py** and **PS2-^{Et}Py**, and calculated weight% of borane side groups and 4-ethylpyridine

Glass transition temperatures (T_g) were measured by differential scanning calorimetry (Figure S3); the mid-point of the transition during the second heating cycle to 130 °C at 10 °C min^{-1} is reported, showing a T_g close to that of unfunctionalized polystyrene.

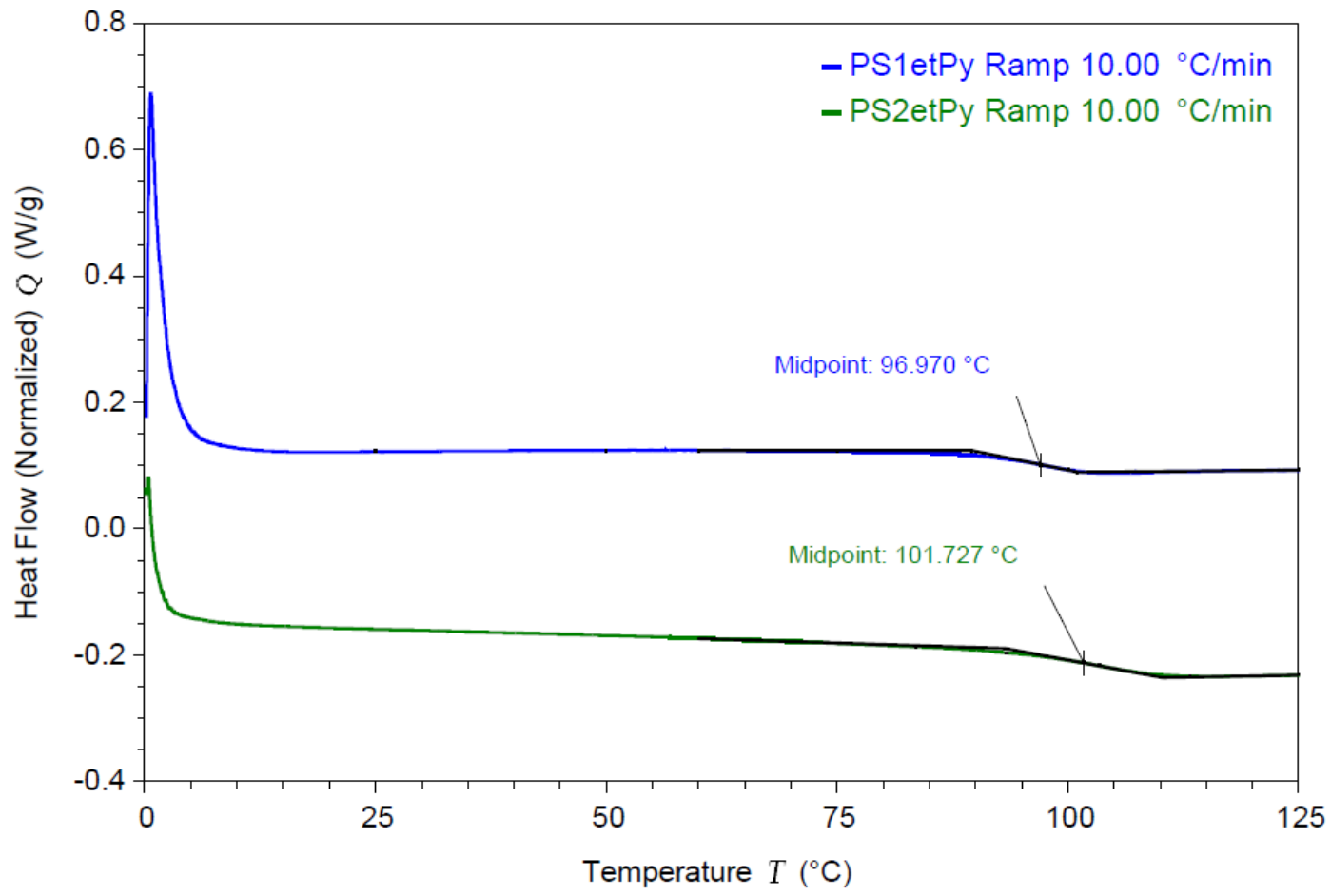

Figure S3. DSC curves for **PS1-^{Et}Py** and **PS2-^{Et}Py** during the second heating ramp.

Table S1. Results from differential scanning calorimetry (DSC) and thermogravimetric analyses (TGA)

Polymer	**PS1-^{Et}Py**	**PS2-^{Et}Py**
$T_{g,\ midpoint}$ (°C)	97.0	101.7
T_d (°C)	142.4 / 413.6	145.3 / 414.9

4. Photophysical Studies

4.1 Absorption and Emission Spectra

UV-Vis and fluorescence spectra of the molecular model compounds, copolymers, and their isolated pyridine complexes were recorded in toluene (chromophore concentration 3.0×10^{-5} M). For all complexes, spectra were recorded after adding a ca. 1000-fold excess of ^{Et}Py to the solution to avoid emission from the more strongly emissive uncomplexed species.

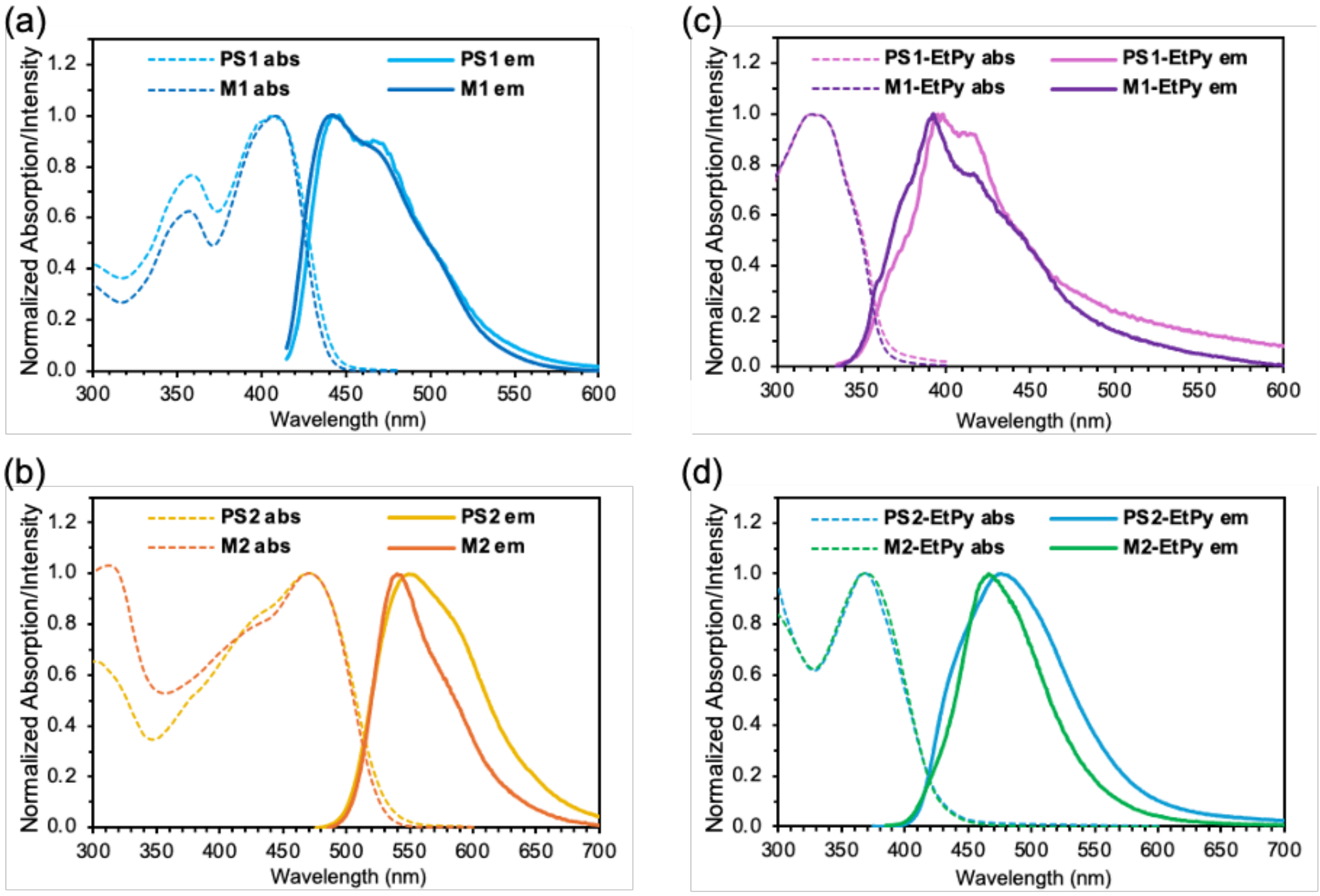

Figure S4. Comparison of normalized absorption (abs) and emission (em) spectra of the free (**PS1**, **PS2**) and complexed (**PS1-^{Et}Py**, **PS2-^{Et}Py**) copolymers with those of the free (**M1**, **M2**) and complexed (**M1-^{Et}Py**, **M2-^{Et}Py**) molecular model compounds; excitation at longest wavelength λ_{max}.

PS1 | **PS2**

no ^{Et}Py + ^{Et}Py | no ^{Et}Py + ^{Et}Py

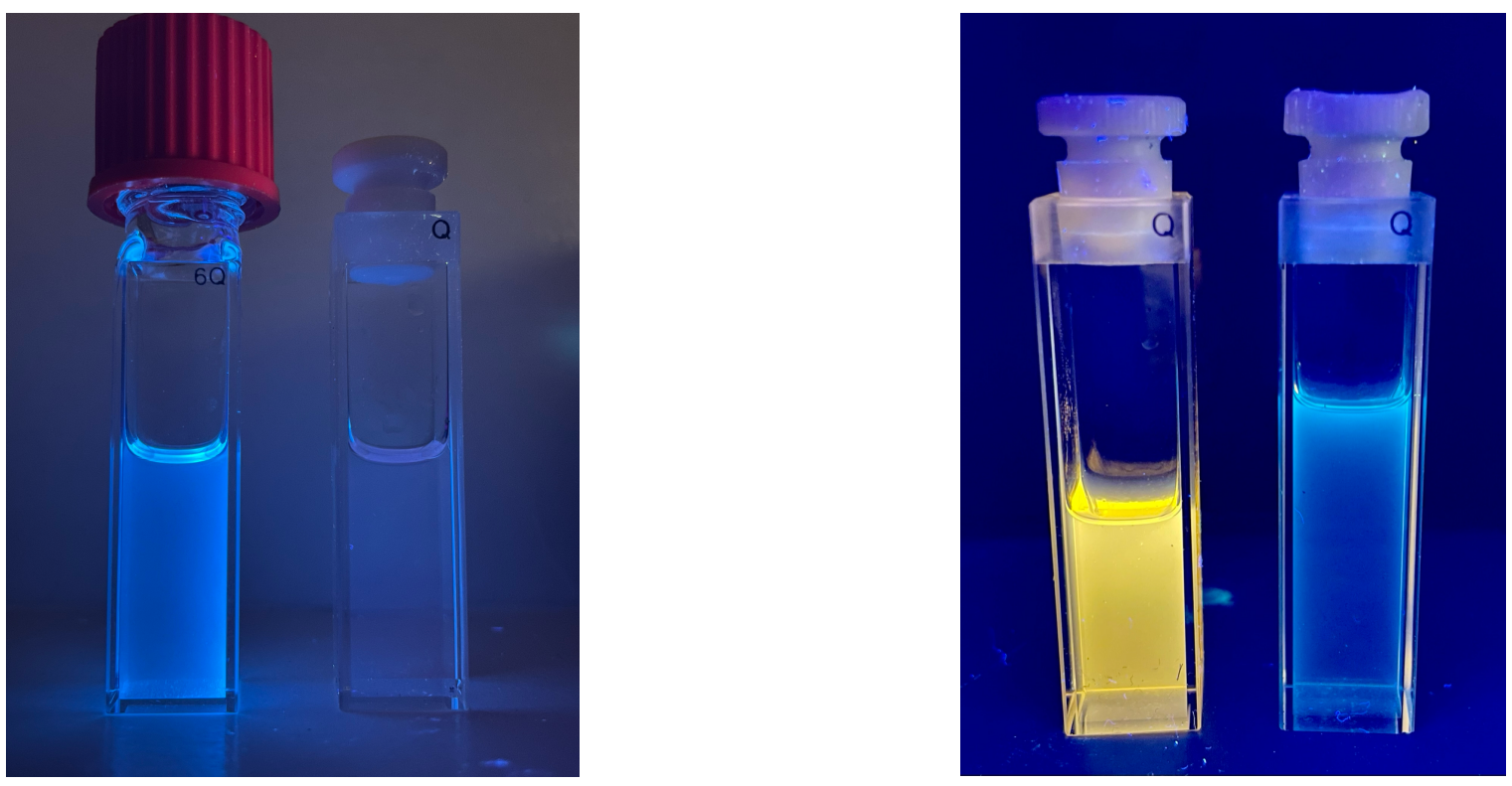

Figure S5. Photographs of solutions of the free (left) and complexed (right) copolymers **PS1** and **PS2** in toluene irradiated with a handheld UV lamp at 365 nm.

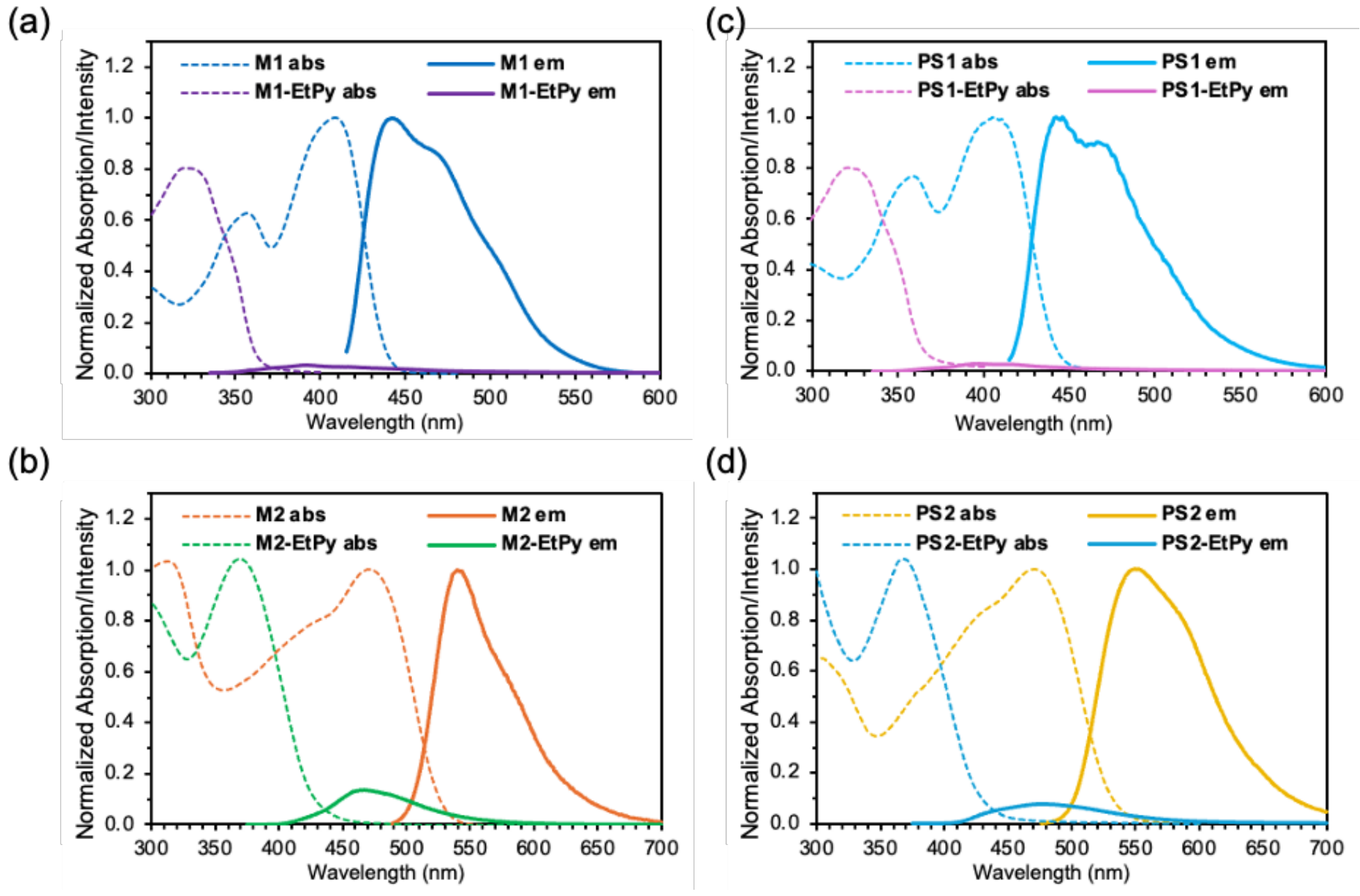

Figure S6. Comparison of absorption (abs) and emission (em) spectra of (a) **M1** and **M1-^{Et}Py**, (b) **M2** and **M2-^{Et}Py**, (c) **PS1** and **PS1-^{Et}Py**, and (d) **PS2** and **PS2-^{Et}Py** in toluene (excitation at longest wavelength λ_{max}), illustrating the effect of complexation on the photophysical properties. The absorption intensities of the free Lewis acids were normalized, and the absorption intensities of the complexes were then scaled to those of the free molecular Lewis acids based on the relative absorptivities (from titration data, see Figure S20) of the free acids and complexes. The emission intensities of the complexes were scaled relative to those of the free molecular organoborane Lewis acids (**M1** and **M2**), accounting also for differences in absorbance at the excitation wavelength.

For scaling of the data presented in **Figure S6** and **Figure 2** of the manuscript, the emission intensities for **PS1** and **PS2** were scaled relative to those of the free organoborane LAs **M1** and **M2**, considering the relative quantum yields and the area under the emission curve; similarly, for the complexes, the emission intensity is shown relatively to that of **M1** and **M2** respectively, considering the relative quantum yield, the area under the emission curve, and the absorbance at the excitation wavelength.

Formula for scaling of absorption data of **M-EtPy** and **PS-EtPy**:

$A_{\mathbf{M\text{-}EtPy}} = A_{\mathbf{M}} \times (\varepsilon_{\mathbf{M\text{-}EtPy}}/\varepsilon_{\mathbf{M}})$ and

$A_{\mathbf{PS\text{-}EtPy}} = A_{\mathbf{PS}} \times (\varepsilon_{\mathbf{M\text{-}EtPy}}/\varepsilon_{\mathbf{M}})$,

assuming that the absorption of the polymer repeat unit is identical to that of the molecular model compound

Formula for scaling of emission data of **M2-EtPy** and **PS2-EtPy**:

$I_{\mathbf{M\text{-}EtPy}} = I_{\mathbf{M}} \times (\Phi_{\mathbf{M\text{-}EtPy}}/\Phi_{\mathbf{M}}) \times (A(\lambda_{exc,\mathbf{M}})/A(\lambda_{exc,\mathbf{M\text{-}EtPy}}))$ and

$I_{\mathbf{PS\text{-}EtPy}} = I_{\mathbf{PS}} \times (\Phi_{\mathbf{PS\text{-}EtPy}}/\Phi_{\mathbf{PS}}) \times (A(\lambda_{exc,\mathbf{M}})/A(\lambda_{exc,\mathbf{M\text{-}EtPy}}))$,

where $A(\lambda_{exc,\mathbf{M}})/A(\lambda_{exc,\mathbf{M\text{-}EtPy}})$ is the relative ratio of the absorbance for the free acid and the complex at the respective excitation wavelength. The intensity was further corrected for differences in the integrated emission area.

Note that the quantum yield for **M1-EtPy** and **PS1-EtPy** was too low to determine accurately; hence, data acquired after the addition of EtPy were used directly in Figures 2 and S6.

4.2 Fluorescence Lifetime and Quantum Yield Measurements

Fluorescence lifetime measurements were performed using 4096 channels over 224.7 ns, corresponding to 0.05486 ns per channel.

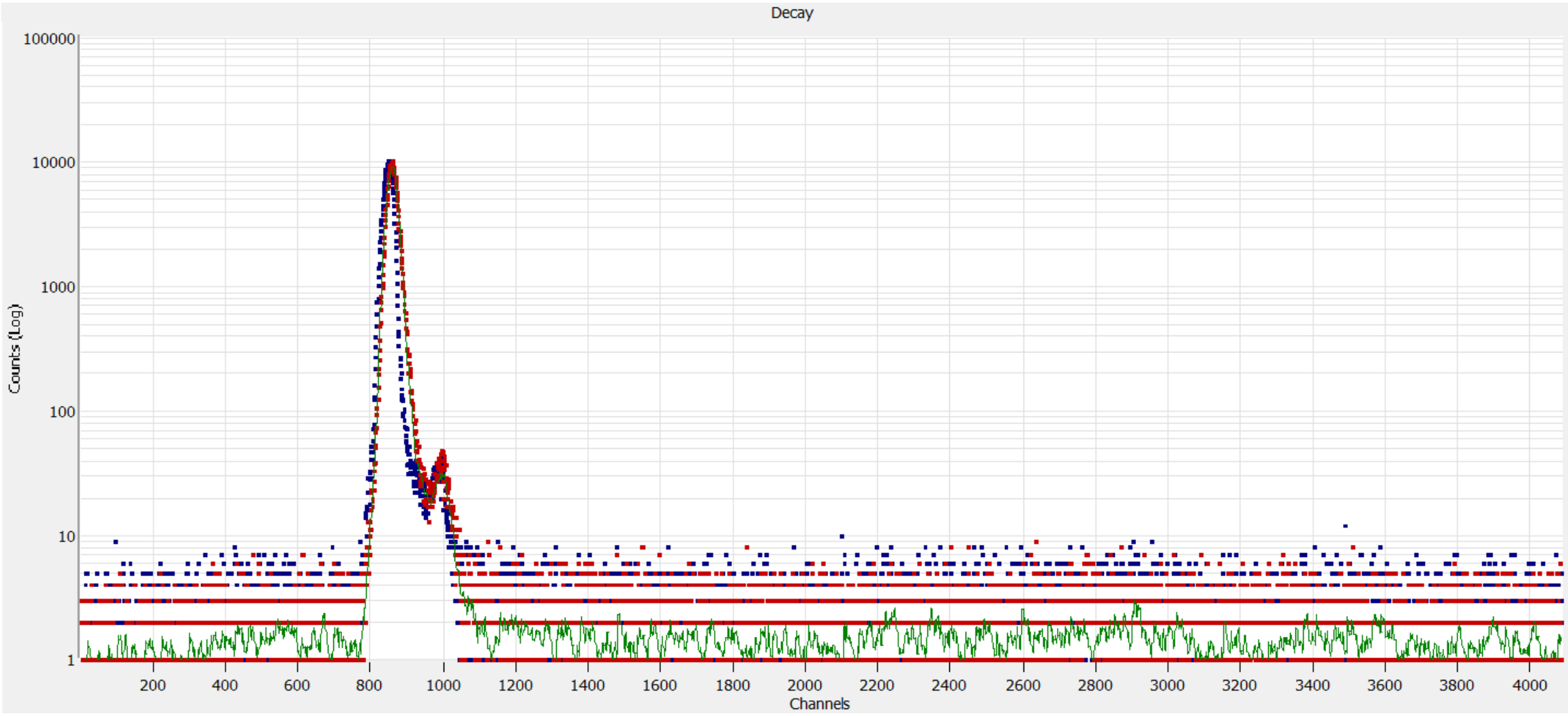

Figure S7. Fluorescence decay of **M1** (blue: prompt, red: fluorescence decay data, green: single-exponential fit); excited at 390 nm, detected at 440 nm. Single exponential fit with τ = 0.42 ns, χ^2 = 1.75.

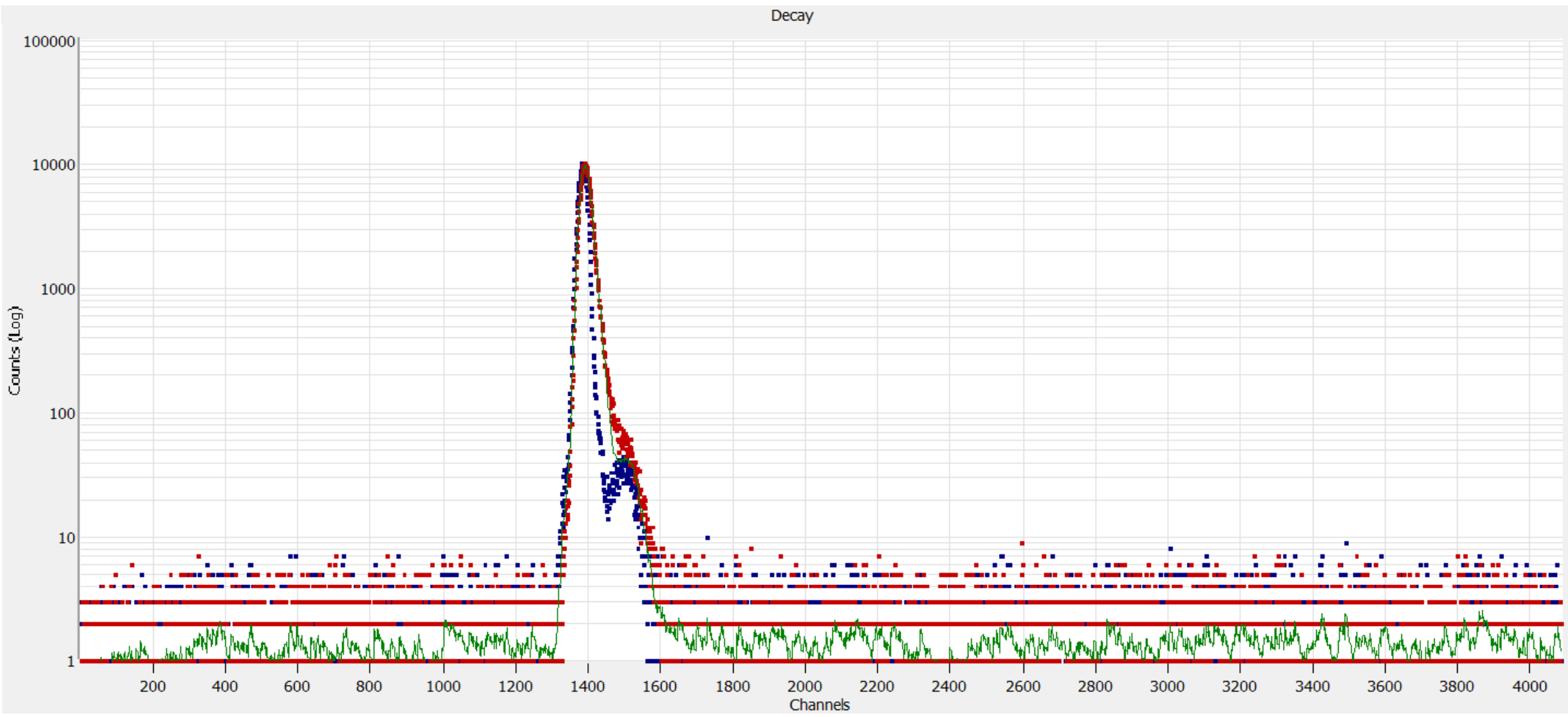

Figure S8. Fluorescence decay of **PS1** (blue: prompt, red: fluorescence decay data, green: single-exponential fit); excited at 390 nm, detected at 440 nm. Single exponential fit with τ = 0.60 ns, χ^2 = 1.23.

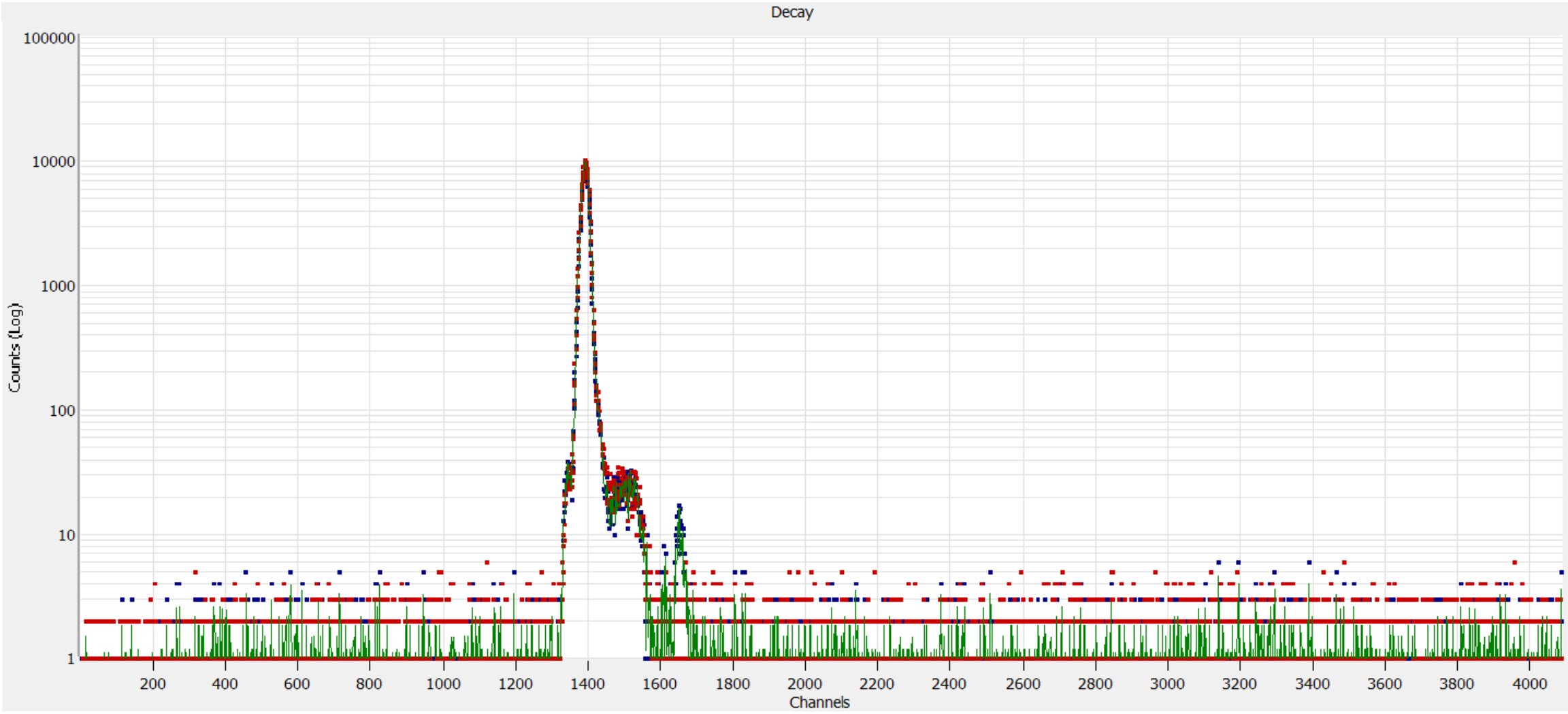

Figure S9. Fluorescence decay of **M1-^{Et}Py** (blue: prompt. green: fluorescence decay data, green: double-exponential fit); excited at 350 nm, detected at 390 nm. Single-exponential fit with τ_1 = 0.19 ns, χ^2 = 1.32. The very rapid emission decay of complex of **M1** did not allow reliable lifetime data to be determined.

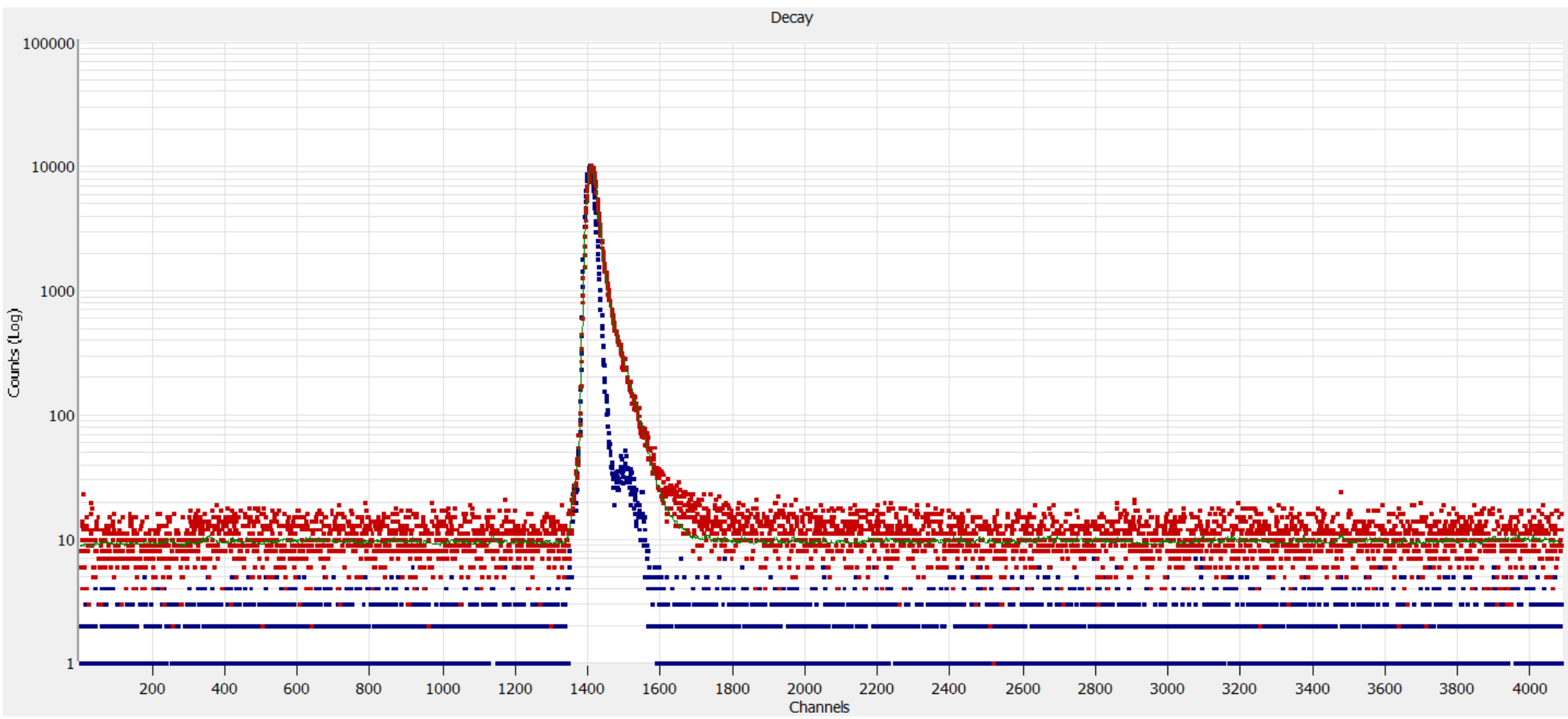

Figure S10. Fluorescence decay of **PS1-^{Et}Py** (blue: prompt, red: fluorescence decay data, green: double-exponential fit); excited at 300 nm, detected at 390 nm. Double exponential fit with τ_1 = 0.32 ns (71.3% relative amplitude) and τ_2 = 1.85 ns (28.7% relative amplitude), χ^2 = 1.27.

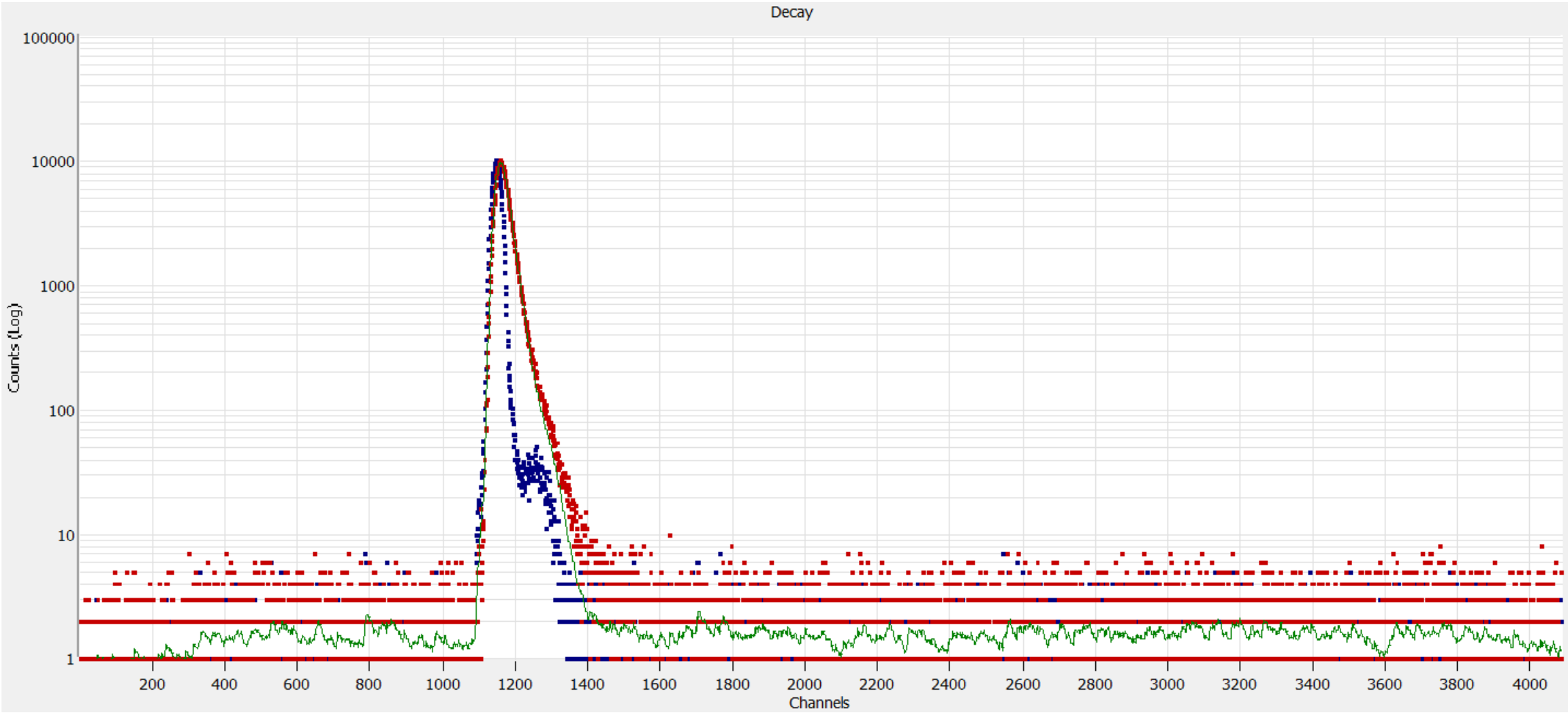

Figure S11. Fluorescence decay of **M2** (blue: prompt, red: fluorescence decay data, green: single-exponential fit); excited at 450 nm, detected at 550 nm. Single-exponential fit with τ = 1.07 ns, $\chi^2 = 1.19$.

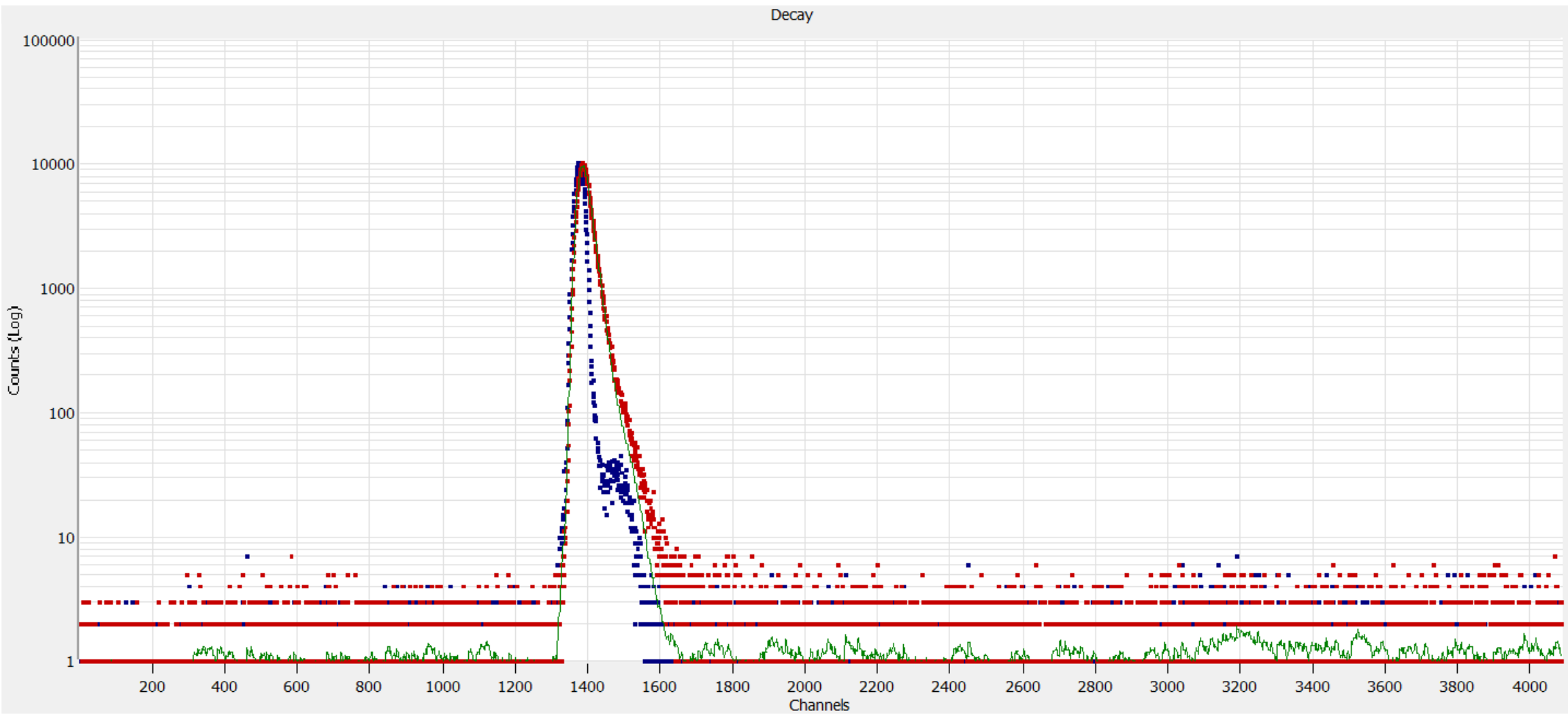

Figure S12. Fluorescence decay of **PS2** (blue: prompt, red: fluorescence decay data, green: single-exponential fit); excited at 450 nm, detected at 540 nm. Single-exponential fit with τ = 0.97 ns, $\chi^2 = 1.23$.

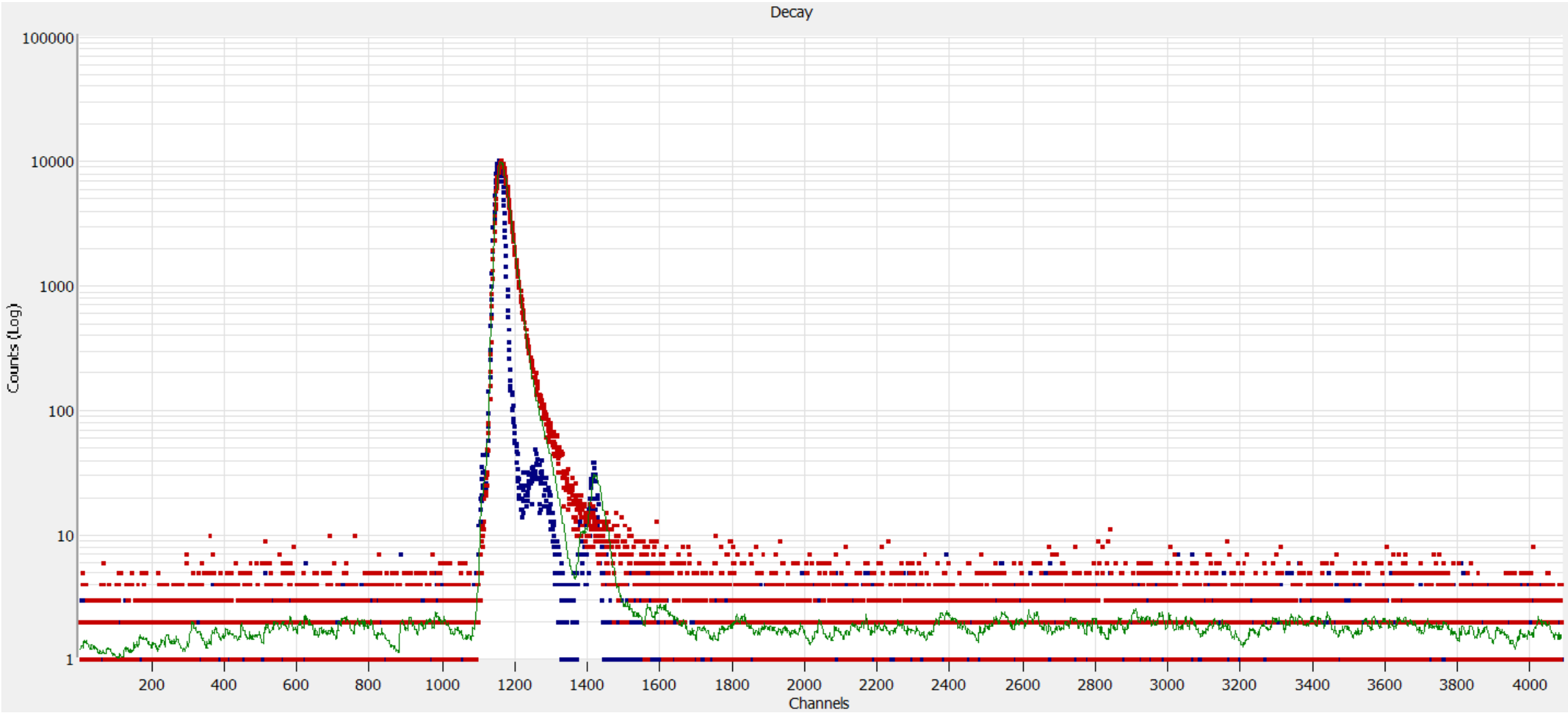

Figure S13. Fluorescence decay of **M2-^{Et}Py** (blue: prompt, red: fluorescence decay data, green: single-exponential fit); excited at 350 nm, detected at 420 nm. Single-exponential fit with τ = 1.01 ns, χ^2 = 1.68.

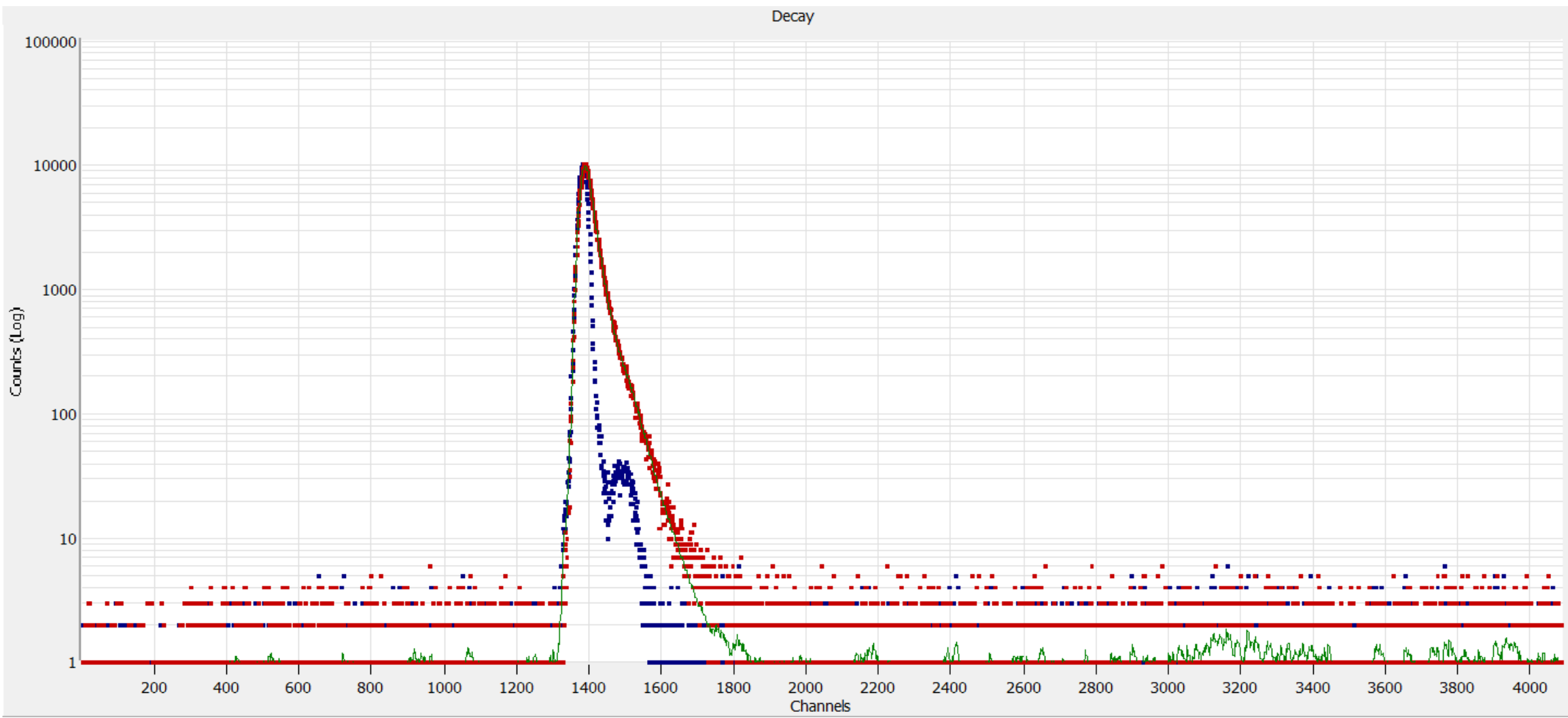

Figure S14. Fluorescence decay of **PS2-^{Et}Py** (blue: prompt, red: fluorescence decay data, green: double-exponential fit); excited at 390 nm, detected at 460 nm. Double exponential fit with τ_1 = 0.89 ns (77.7 % relative amplitude) and τ_2 = 2.48 ns (22.3 % relative amplitude), χ^2 = 0.84.

Table S2. Results of fluorescence lifetime and quantum yield measurements in toluene

Molecule/ Polymer	λ_{ex} / λ_{obs} (nm)	τ_{av} [a] (ns)	χ^2	Φ_{Fl} [b] (%)	k_r [c] (10^7 s^{-1})	k_{nr} [c] (10^7 s^{-1})
**M1**	390 / 440	0.42	1.75	9.5	22.6	215
**PS1**	350 / 390	0.60	1.23	9.4	15.7	151
**M2**	450 / 550	1.07	1.19	27.0	25.2	68.2
**PS2**	450 / 540	0.97	1.24	17.5	18.0	85.1
**M1-^{Et}Py**	350 / 390	<0.25 [d]	n.d.	<0.5 [d]	n.d.	n.d.
**PS1-^{Et}Py**	300 / 390	0.42	1.27	<0.5 [d]	<1	>230
**M2-^{Et}Py**	350 / 420	1.01	1.68	9.9	9.8	89.2
**PS2-^{Et}Py**	390 / 460	1.03	0.84	3.1	3.0	87.6

[a] Average fluorescence lifetime. [b] Fluorescence quantum yield determined using an integrating sphere. [c] Radiative (k_r) and non-radiative (k_{nr}) decay rate constants calculated using the equations $k_r = \Phi / \tau$, $k_{nr} = (1 - \Phi) / \tau$. [d] Lifetime/quantum yield below instrument capabilities.

Table S3. Comparison of photophysical data in toluene solution with computed data (CAM-B3LYP, 6-31G(d))

	Experimental Data (Toluene Solution)					TD-DFT Data (CAM-B3LYP) [e]			
Compound	λ_{abs} (nm)	ε (10^4 $M^{-1}cm^{-1}$)	λ_{Fl} [a] (nm)	Φ_{Fl} [b] (%)	τ_{av} [c] (ns) (χ^2)	λ_{abs} (nm)	f_{abs}	λ_{Fl} (nm)	f_{Fl}
**M1**	409<br>357	4.12<br>2.55	442	9.5	0.42<br>(1.75)	366<br>317	0.98<br>0.43	401	1.05
**PS1**	405	n.d.	446	9.4	0.60<br>(1.23)	--	--	--	--
**M2**	472<br>313	3.63	540	27.0	1.07<br>(1.20)	418<br>368	1.51<br>0.45	461	1.67
**PS2**	471	n.d.	550	17.5	0.97	--	--	--	--
**M1-^{Et}Py**	320	3.31	392	<0.5 [d]	<0.25 [d]	335<br>306<br>292	0.00<br>0.84<br>0.41	599<br>348<br>336	0.00<br>0.01<br>0.70
**PS1-^{Et}Py**	321	n.d.	395	<0.5 [d]	0.42<br>(1.27)	--	--	--	--
**M2-^{Et}Py**	369	3.78	466	9.9	1.01<br>(1.68)	351<br>347<br>336	0.69<br>0.58<br>0.44	593<br>404<br>390	0.00<br>0.01<br>1.07
**PS2-^{Et}Py**	368	n.d.	476	3.1	1.03	--	--	--	--

[a] Excited at the longest wavelength absorption maximum. [b] Fluorescence quantum yield determined using an integrating sphere. [c] Average fluorescence lifetime. [d] Lifetime/quantum yield too low to accurately determine. [e] Pyridine and benzene rings were used in the calculations instead of 4-ethylpyridine and 4-*tert*-butylphenyl.

5. Computational Studies

5.1 Computed Orbital Energy Levels and Orbital Plots

The orbital energy levels and transition energies were computed for the model molecules and their corresponding complexes with pyridine (rather than 4-ethylpyridine). The results correlated well with the photophysical data of the molecules. For both the tricoordinate boranes and tetracoordinate borane-pyridine complexes, the orientation for thiophene units was chosen such that neighboring thiophene S atoms are pointing in opposite directions. These conformers were lowest in energy for the tricoordinate boranes based on force-field calculations.

	M1	M1-Py	M2	M2-Py
**Structure**	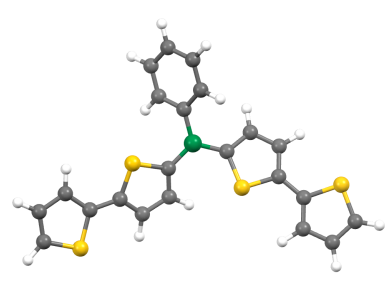	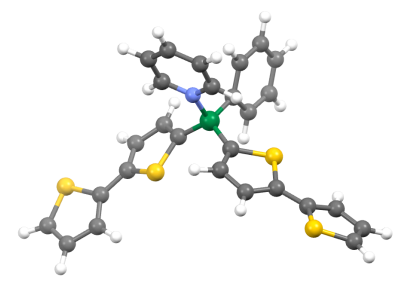	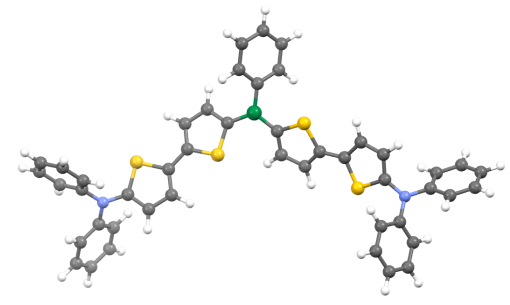	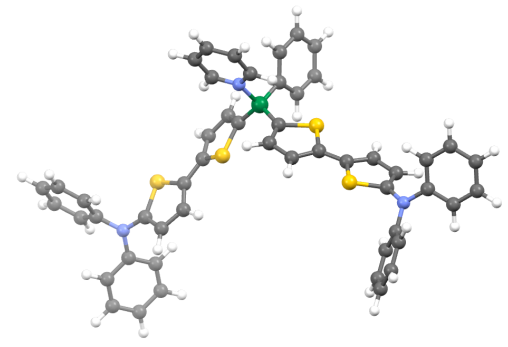
**LUMO+2**		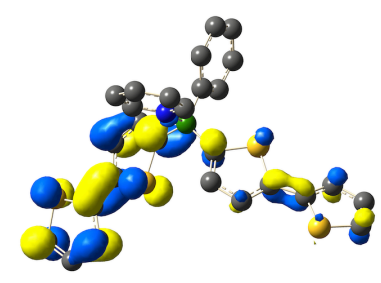	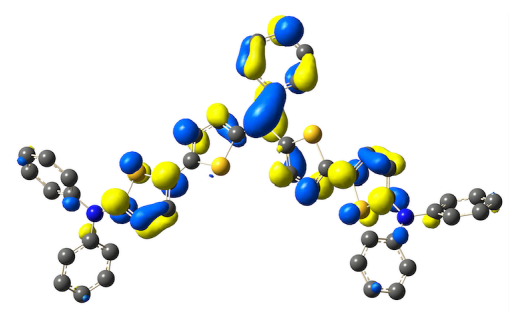	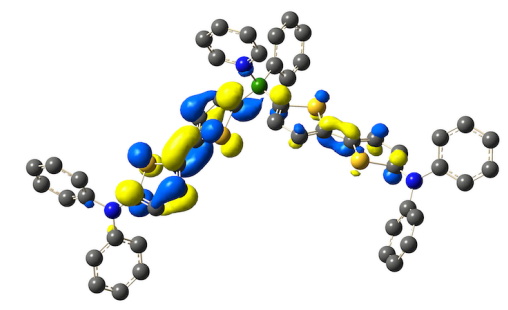
**LUMO+1**	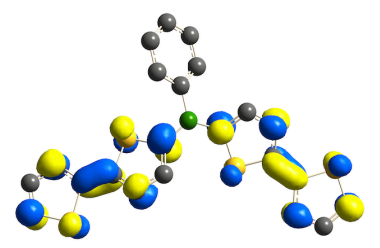	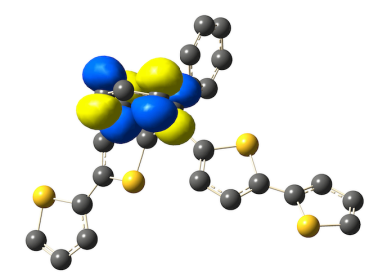	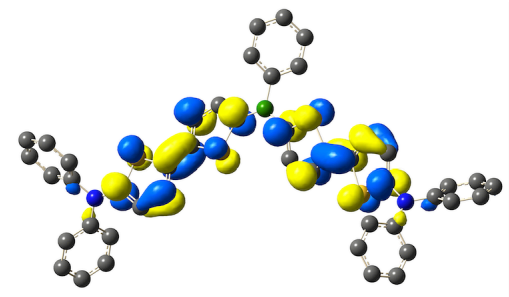	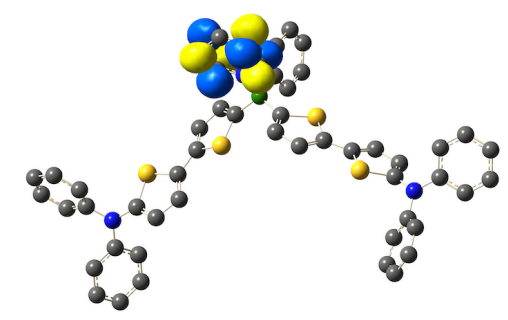
**LUMO**	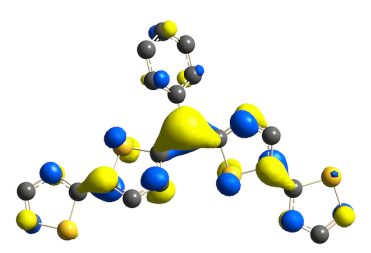	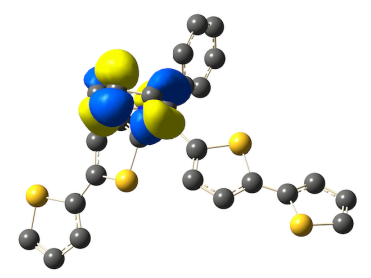	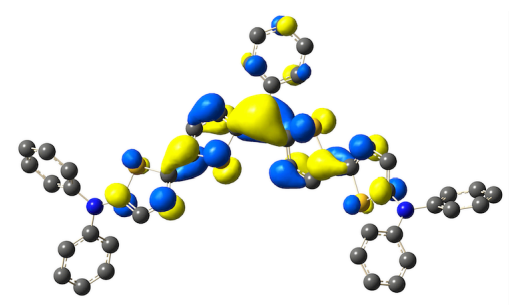	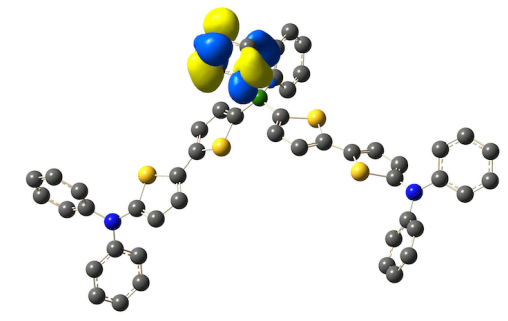
**HOMO**	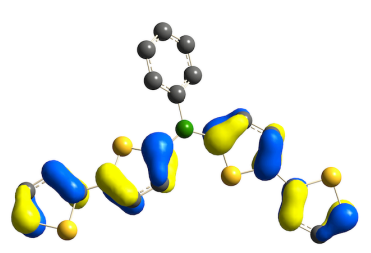	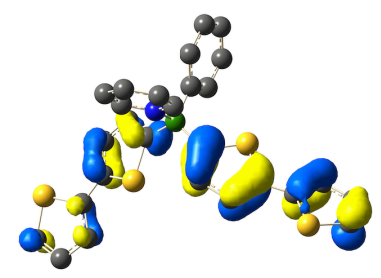	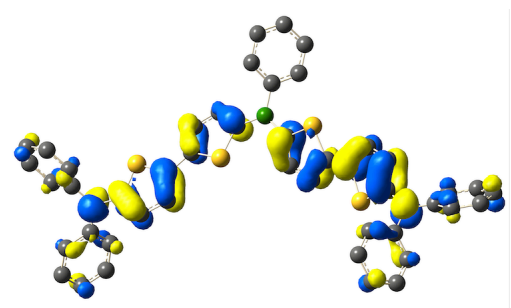	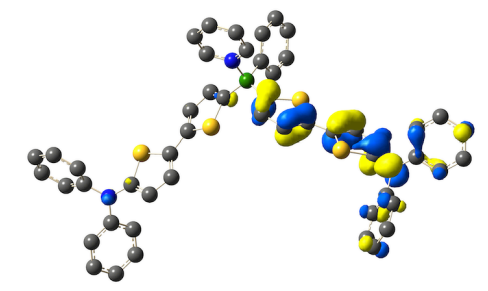
**HOMO-1**	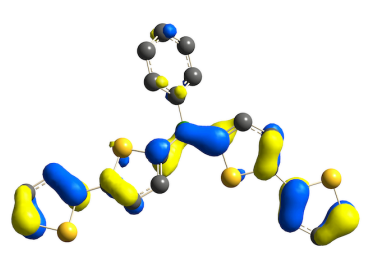	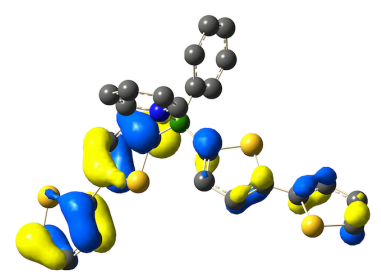	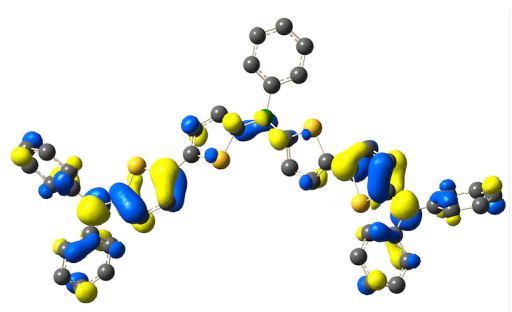	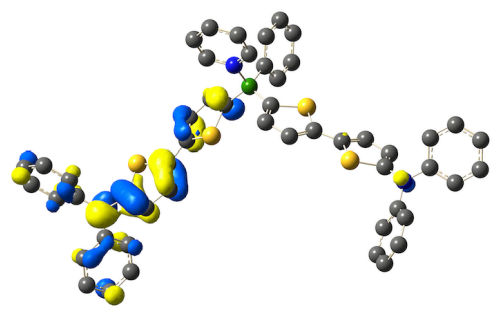
**HOMO-2**		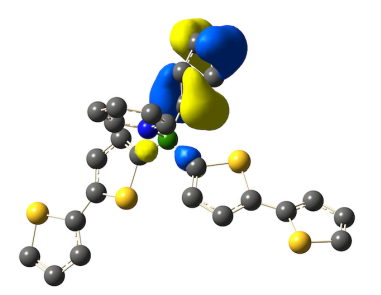	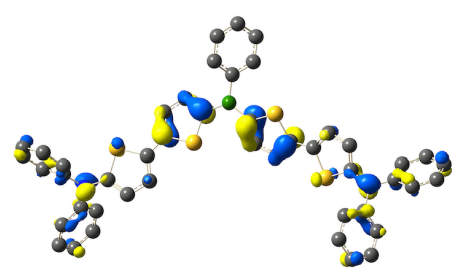	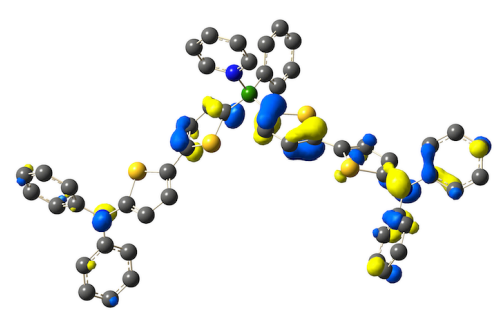

Figure S15. Frontier molecular orbital diagrams (isovalue of 0.04 e/Å^3) for **M1**, **M1-Py**, **M2**, and **M2-Py**. Calculations were performed at the B3LYP/6-31G(d) level.

Table S4. Ground state orbital energy levels from DFT computations (B3LYP, 6-31G(d)).

Energy (eV)	HOMO-2 (eV)	HOMO-1 (eV)	HOMO (eV)	LUMO (eV)	LUMO+1 (eV)	LUMO+2 (eV)	ΔE_g[a] (eV)
**M1**	-6.637	-5.806	-5.440	-2.154	-1.291	-0.660	**3.287**
**M1-Py**	-6.134	-5.224	-5.012	-2.114	-1.389	-1.016	**2.897**
**M2**	-5.858	-4.990	-4.813	-2.056	-1.275	-0.656	**2.757**
**M2-Py**	-5.514	-4.745	-4.625	-2.112	-1.389	-1.111	**2.513**

[a] HOMO-LUMO gap

Table S5. Comparison of computed orbital energy levels (RB3LYP/6-31G(d)), computed band gaps, and experimentally estimated band gaps

Compound	LUMO (eV)	HOMO (eV)	$E_{g, DFT}$ (eV) [a]	E_{TDDFT} (eV) [b]	$E_{g, UV}$ (eV) [c]
**M1**	-2.154	-5.440	3.287	3.39	3.03
**M1-Py / M1-^{Et}Py**	-2.114	-5.012	2.897	3.71	3.81
**M2**	-2.056	-4.813	2.757	2.97	2.63
**M2-Py / M2-^{Et}Py**	-2.112	-4.625	2.513	3.53	3.36

[a] HOMO-LUMO gap. [b] S_0-S_1 transition. [c] Estimated from longest wavelength absorption maxima.

5.2 TD-DFT Computations

Table S6. Computed absorption transitions to excited states for **M1** and **M1-Py** from TD-DFT calculations (B3LYP/6-31G(d) // rcam-B3LYP/6-31G(d))

Free Lewis Acid M1; E(TD-HF/TD-DFT) = -2464.30970840 Hartree					
Excited states	Energy (eV)	Wavelength (nm)	Oscillator strength, *f*	Assignment	% Contrib'n [a]
**S1**	**3.390**	**365.8**	**0.982**	H-1 → L+1	6.69
				**H → L**	**89.40**
**S2**	**3.098**	**317.3**	**0.425**	**H-1 → L**	**80.04**
				H → L+1	13.31
**Pyridine Complex M1-Py; E(TD-HF/TD-DFT) = -2712.46142182 Hartree**					
Excited states	Energy (eV)	Wavelength (nm)	Oscillator strength, *f*	Assignment	% Contrib'n [a]
**S1**	3.705	334.6	0.004	H-8 → L	3.68
				**H → L**	**94.15**
**S2**	3.932	315.3	0.010	H-9 → L	4.26
				**H-1 → L**	**91.59**
**S3**	**4.046**	**306.4**	**0.842**	H-1→ L+2	8.80
				H-1 → L+3	22.07
				H-1 → L+1	2.54
				**H → L+2**	**54.13**
				H → L+3	8.62
**S4**	4.240	292.4	0.409	**H-1 → L+2**	**45.41**
				H → L+1	5.84
				**H → L+3**	**42.97**

[a] % contribution = 2 c^2 * 100%

Table S7. Computed absorption transitions to excited states for **M2** and **M2-Py** from TD-DFT calculations (B3LYP/6-31G(d) // rcam-B3LYP/6-31G(d)).

Excited states	Energy (eV)	Wavelength (nm)	Oscillator strength, *f*	Assignment	% Contrib'n [a]
**Free Lewis Acid M2; E(TD-HF/TD-DFT) = -3498.63290409 Hartree**					
**S1**	2.969	417.6	1.512	H-2 → L	6.69
				H-1 → L+1	12.74
				**H → L**	**75.43**
**S2**	3.365	368.4	0.454	H-3 → L	4.32
				**H-1 → L**	**64.38**
				H-1 → L+2	3.22
				H-2 → L+1	23.87
**Pyridine Complex M2-Py; E(TD-HF/TD-DFT) = -3746.77527355 Hartree**					
**Excited states**	**Energy (eV)**	**Wavelength (nm)**	**Oscillator strength, *f***	**Assignment**	**% Contrib'n [a]**
**S1**	3.528	351.4	0.687	H-2 → L	7.16
				H-1 → L+2	5.81
				H-1 → L+3	10.19
				**H → L**	**39.57**
				H → L+1	2.80
				H → L+2	17.41
				H → L+3	11.43
**S2**	3.568	347.5	0.583	H-2 → L	8.18
				H-1 → L+2	7.85
				H-1 → L+3	9.43
				**H → L**	**40.77**
				H → L+1	4.20
				H → L+2	15.61
				H → L+3	8.00
**S3**	3.692	335.9	0.439	H-3 → L	2.17
				H-1 → L	11.19
				**H-1 → L+2**	**40.38**
				H-1 → L+3	2.64
				H → L+1	2.46
				**H → L+3**	**31.90**

[a] % contribution = 2 c^2 * 100%

Table S8. Computed emission for **M1** and **M1-Py** from TD-DFT calculations (B3LYP/6-31G(d) // rcam-B3LYP/6-31G(d))

Free Lewis Acid M1; E(TD-HF/TD-DFT) = -2464.31544514 Hartree					
**Excited states**	**Energy (eV)**	**Wavelength (nm)**	**Oscillator strength, *f***	**Assignment**	**% Contrib'n [a]**
**S1**	**3.092**	**401.0**	**1.047**	H-1 → L+1	5.90
				**H → L**	**91.07**

Pyridine Complex M1-Py; E(TD-HF/TD-DFT) = -2712.46142182 Hartree					
**Excited states**	**Energy (eV)**	**Wavelength (nm)**	**Oscillator strength, *f***	**Assignment**	**% Contrib'n [a]**
**S1**	2.071	598.8	0.0006	**H → L**	**96.96**
**S2**	2.946	420.9	0.008	H-9 → L	3.81
				**H-1 → L**	**93.73**
**S3**	**3.418**	**362.7**	**0.702**	H-1 → L+3	7.32
				H → L+1	6.04
				**H → L+2**	**83.76**

[a] % contribution = 2 c^2 * 100%

Table S9. Computed emission for **M2** and **M2-Py** from TD-DFT calculations (B3LYP/6-31G(d) // rcam-B3LYP/6-31G(d)).

Free Lewis Acid M2; E(TD-HF/TD-DFT) = -3498.63290409 Hartree					
**Excited states**	**Energy (eV)**	**Wavelength (nm)**	**Oscillator strength, *f***	**Assignment**	**% Contrib'n [a]**
**S1**	**2.688**	**461.2**	**1.669**	H-2 → L+2	3.05
				H-1 → L+1	11.50
				**H → L**	**81.76**

Pyridine Complex M2-Py; E(TD-HF/TD-DFT) = -3746.77527355 Hartree					
**Excited states**	**Energy (eV)**	**Wavelength (nm)**	**Oscillator strength, *f***	**Assignment**	**% Contrib'n [a]**
**S1**	2.092	592.6	0.0003	H-3 → L	4.37
				**H → L**	**92.75**
**S2**	3.071	403.7	0.005	H-3 → L	3.30
				H-4 → L+2	17.18
				**H-5 → L+3**	**76.23**
**S3**	**3.183**	**389.5**	**1.068**	H → L+1	10.67
				H → L+2	20.46
				**H → L+3**	**62.16**

[a] % contribution = 2 c^2 * 100%

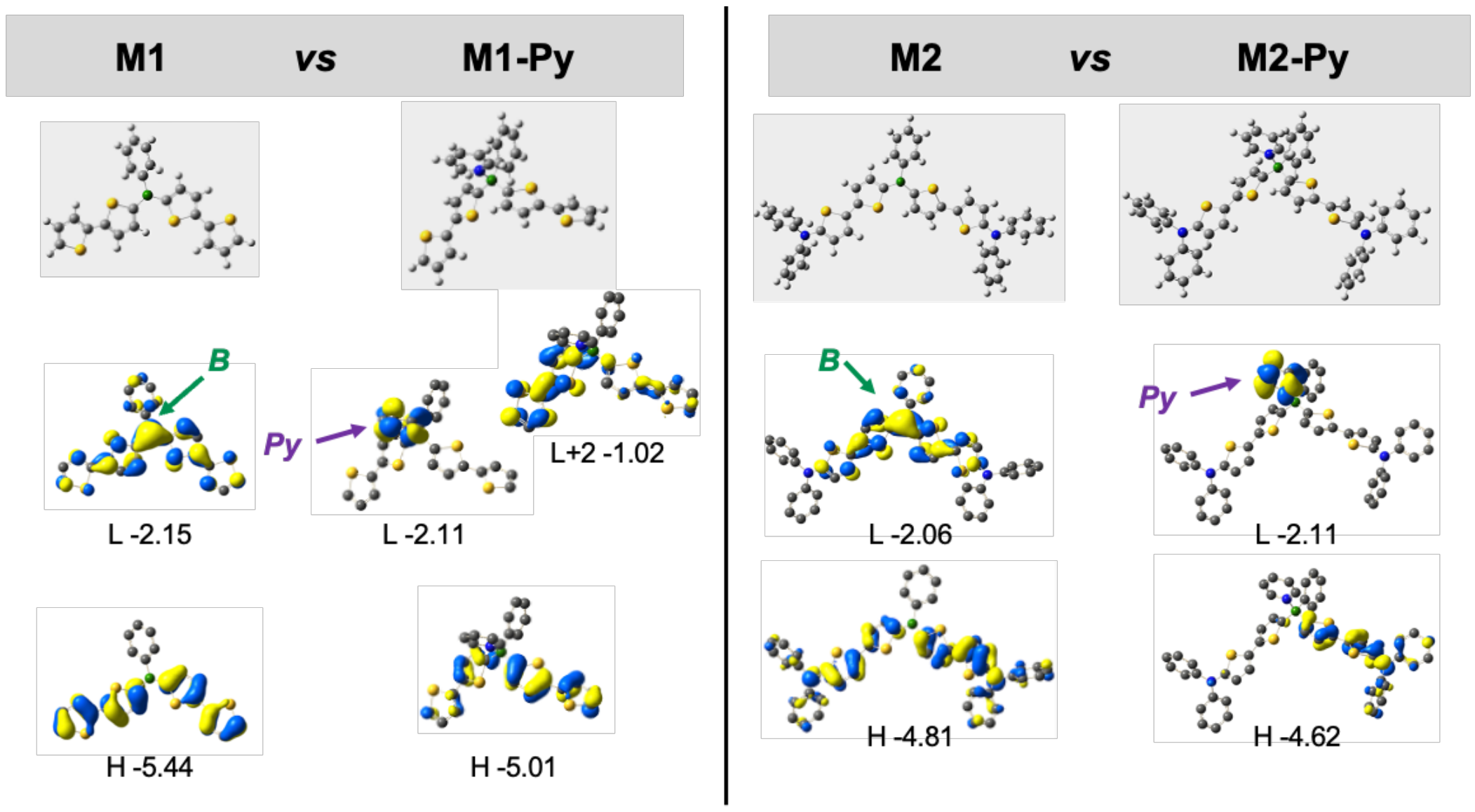

TD-DFT CAM-B3LYP 6-31g(d):

**Compound**	**M1**	**M1-Py**	**M2**	**M2-Py**
**Transition Energy / eV (nm)**	3.39 (**366**)	4.05 (**306**) [a]	2.97 (**418**)	3.53 (**351**)
**Oscillator strength**	0.98	0.84	1.51	0.69
**Orbital Contributions**	H -> L (89%)	H -> L+2 (54%)	H -> L (75%)	H -> L (40%)

[a] Transition to S_3; the excitation of **M1-Py** to S_1 at 3.71 eV (335 nm) corresponds to a H->L transition but has a low oscillator strength of 0.004.

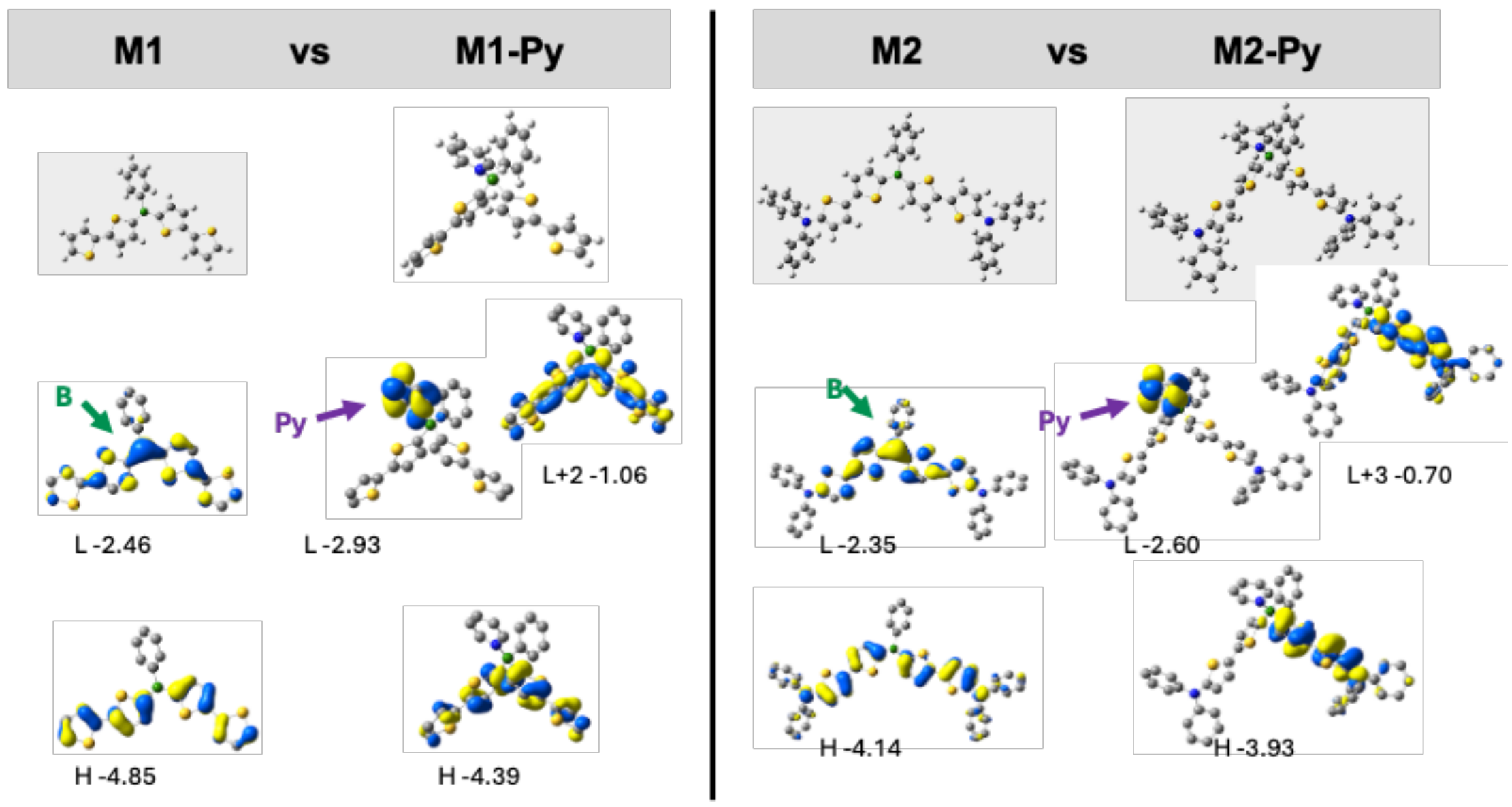

TD-DFT CAM-B3LYP 6-31g(d):

**Compound**	**M1**	**M1-Py**	**M2**	**M2-Py**
**Transition Energy / eV (nm)**	3.09 (**401**)	3.42 (**363**) [a]	2.69 (**461**)	3.18 (**390**) [b]
**Oscillator strength**	1.05	0.70	1.67	1.07
**Orbital Contributions**	H -> L (91%)	H -> L+2 (84%)	H -> L (82%)	H -> L+3 (62%)

[a] Transition from S_3; the emission of **M1-Py** from S_1 at 2.07 eV (599 nm) corresponds to a H->L transition but has a low oscillator strength of 0.0006.
[b] Transition from S_3; the emission of **M2-Py** from S_1 at 2.09 eV (593 nm) corresponds to a H->L transition but has a low oscillator strength of 0.0003.

Figure S16. Comparisons of orbital distributions (isovalue of 0.04 e/Å^3) and computed energies (eV) for frontier orbitals involved in the lowest energy absorption and emission of **M1**, **M1-Py**, **M1**, and **M2-Py**. Structures were optimized at the S_0 (top, absorption) and S_1 state (bottom, emission), respectively, using the B3LYP/6-31G(d) functional. Results from TD-DFT calculations at the CAM-B3LYP/6-31G(d) level are provided in tabular form.

6. Analysis of Lewis Pair Formation

Bulk mechanical tests of the polymer networks suggested that the binding strength of B-N Lewis pairs in **F1** (bithienyl borane groups as Lewis acidic sites) was higher than in **F2** (diphenylamino-bithienyl borane as Lewis acidic sites). Studies on small-molecule model systems were carried out to rationalize and quantify the differences.

6.1 UV-Vis Titration Experiments and Determination of Binding Constants

To determine the binding affinities of the B-N Lewis pairs, UV-Vis titration experiments were performed. Solutions of small molecule borane LA and ^{Et}**Py** Lewis base were prepared from stock solutions in anhydrous toluene inside a N_2-filled glovebox. The absorption baseline was collected with a blank solution of toluene in a screw-capped Quartz UV-vis cuvette (1 cm path length). The same cuvette was then rinsed three times with the analyte solution (LA) and filled with 2.0 mL of the organoborane solution. The exact volume of the analyte was determined by weight of the solution added to the cuvette. The corresponding titrant, ^{Et}**Py**, was added to the cuvette inside a glovebox using a 10-microliter syringe to avoid exposure to air. UV-Vis absorption data were recorded on a Varian Cary 5000 UV-Vis/NIR spectrophotometer.

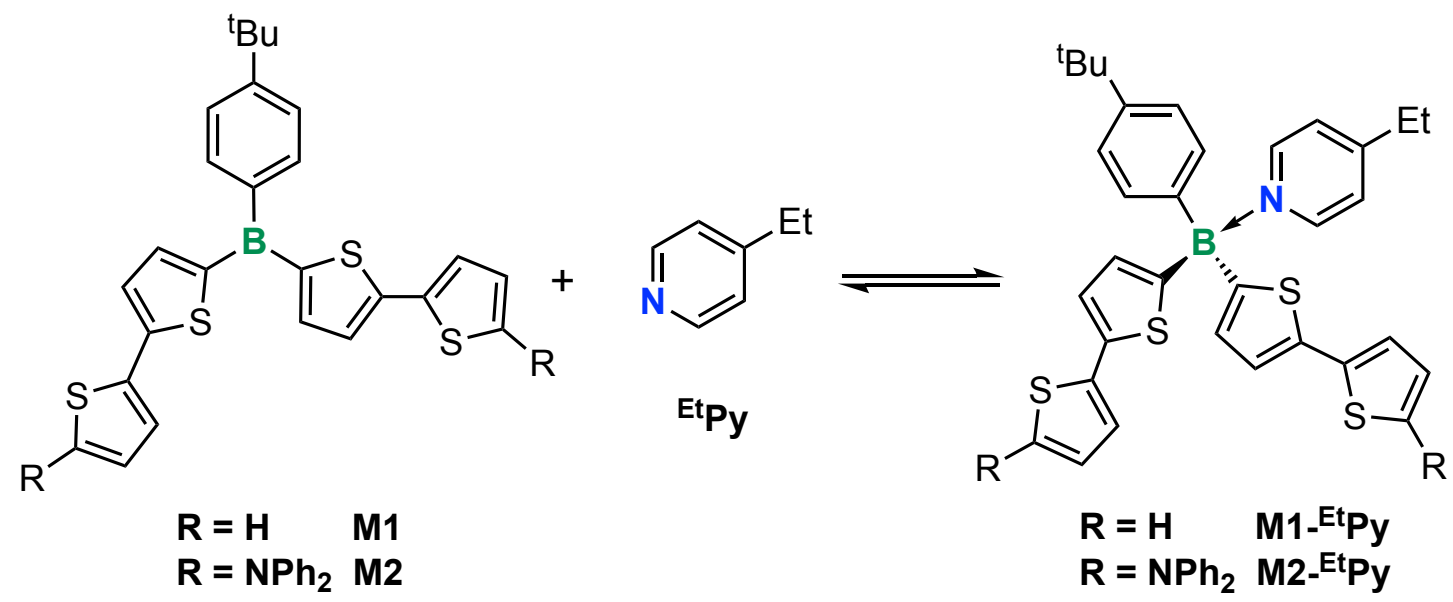

For **M1**, 2.25 mL of a 4.60×10^{-5} M solution in anhydrous toluene was loaded into a cuvette and titrated with incremental amounts of a solution of ^{Et}**Py** in toluene (7.34×10^{-4} M), up to a total of 150 µL of added base. After each addition, the cuvette was removed from the glovebox, placed in the spectrometer cell, and three consecutive spectra were recorded at each titration point; the resulting data were averaged.

For **M2**, 2.20 mL of a 4.65×10^{-5} M solution in anhydrous toluene was loaded into a cuvette and titrated with incremental amounts of a solution of ^{Et}**Py** in toluene (1.28×10^{-3} M), up to a total of 150 µL of added base. After each addition, the cuvette was removed from the glovebox, placed in the spectrometer cell, and three consecutive spectra were recorded at each titration point; the resulting data were averaged.

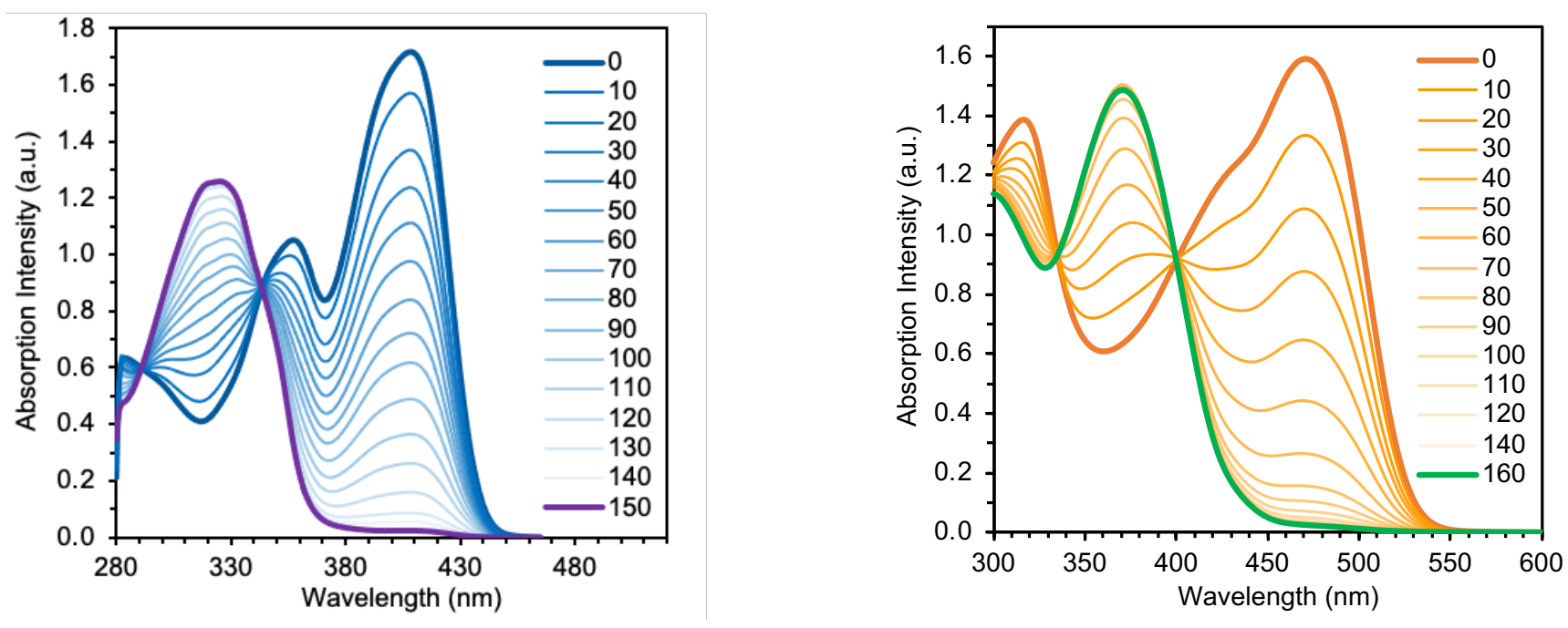

Figure S17. Titration of **M1** (left) and **M2** (right) in toluene with a solution of 4-ethylpyridine in toluene, monitored by UV-Vis absorption spectroscopy; cumulative amounts of 4-ethylpyridine solution added are indicated in units of µL.

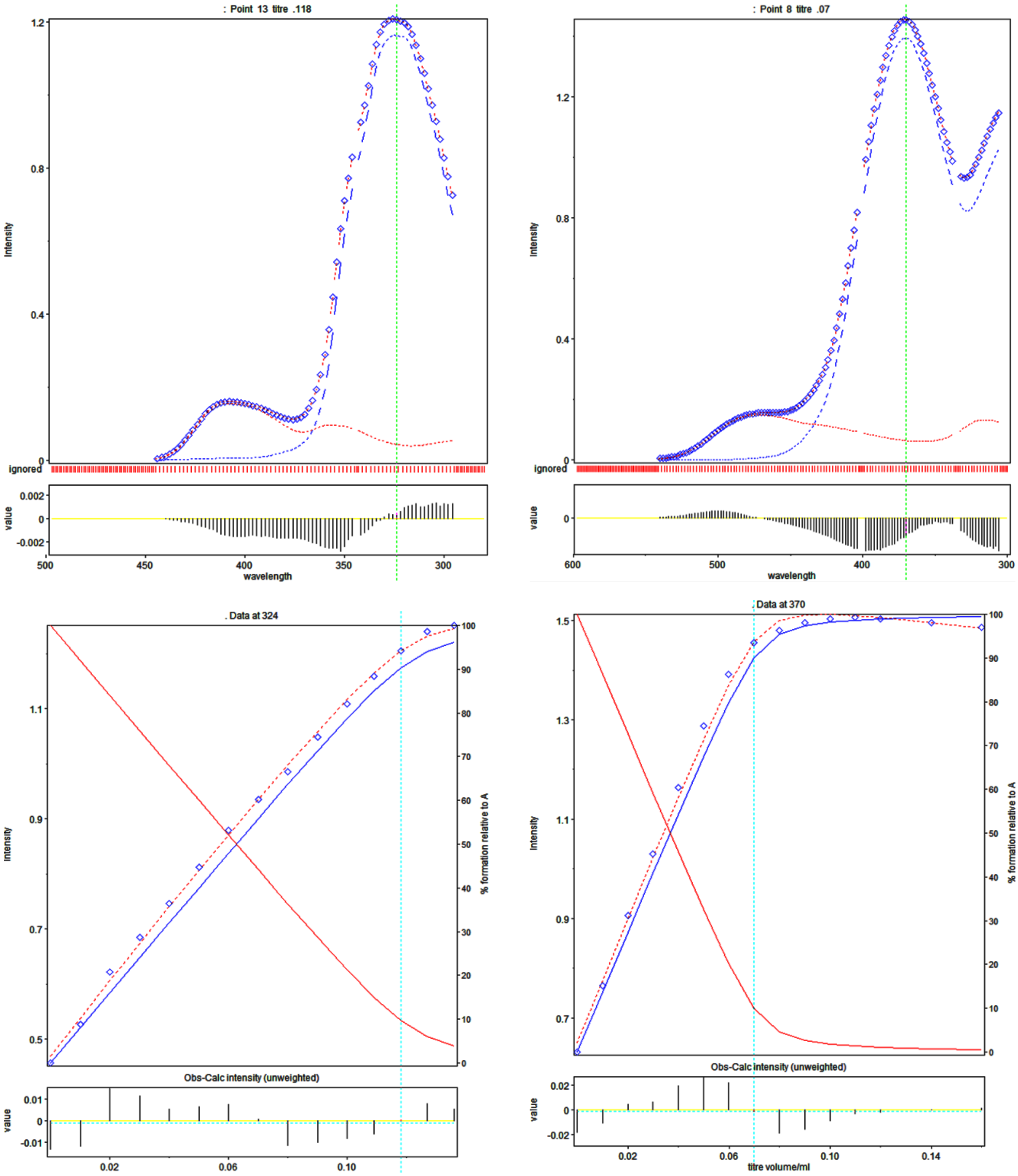

Figure S18. Fit of absorption titration data (Hypspec^{TM} software) for the titration of **M1** with 4-ethylpyridine in toluene ([**M1**] = 4.60 × 10^{-5} M, [^{Et}**Py**] = 7.43 × 10^{-4} M) with a binding constant of lgK = 6.68 (derived from a global fit at 108 different wavelengths over the range of λ = 497 to 280 nm). Selected binding isotherms are illustrated at λ_{abs} = 324 and 406 nm.

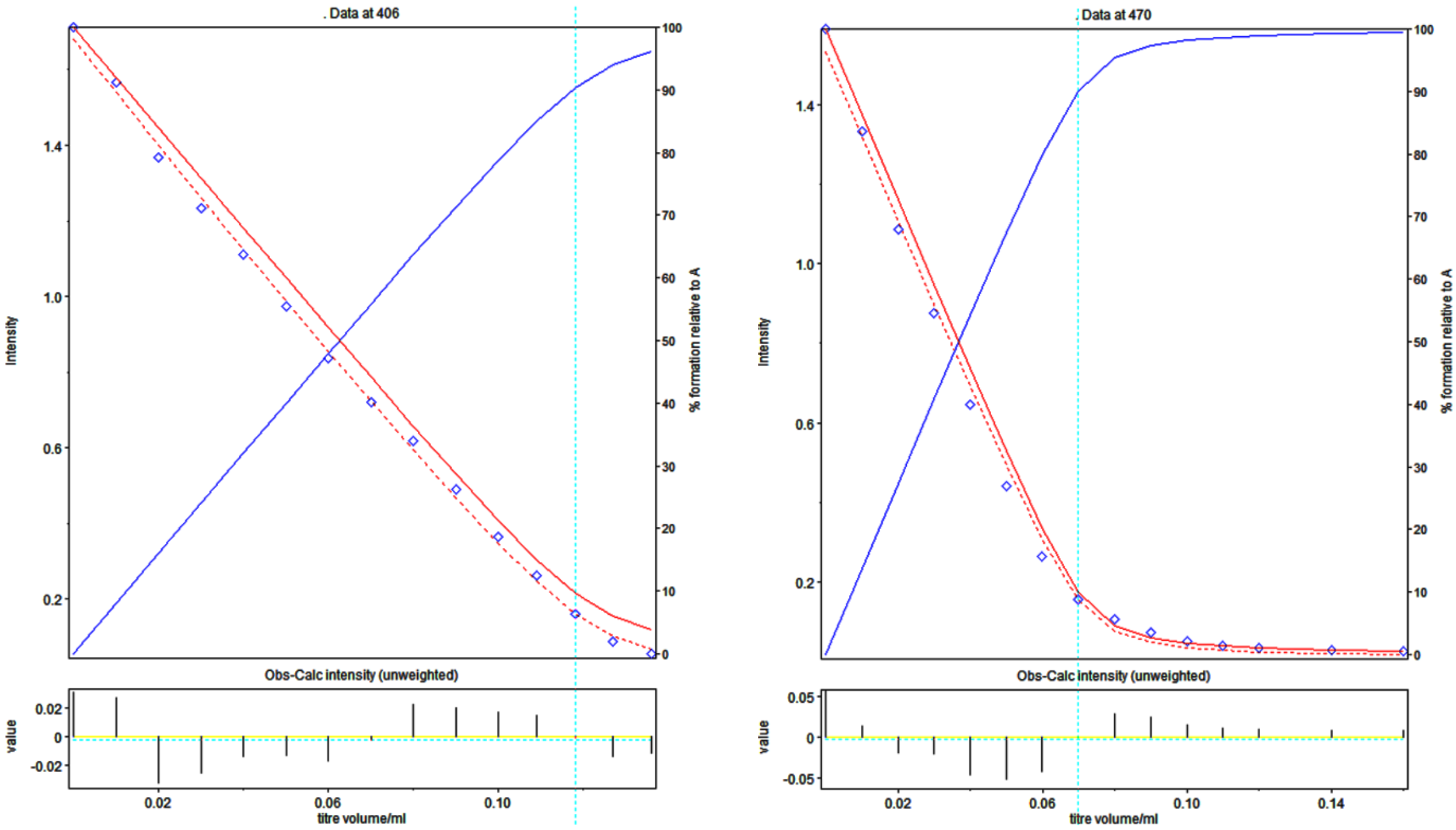

Figure S19. Fit of absorption titration data (Hypspec™ software) for the titration of **M2** with 4-ethylpyridine in toluene ([**M2**] = 4.65 × 10^{-5} M, [^{Et}**Py**] = 1.28 × 10^{-3} M) with a binding constant of lgK = 6.54 (derived from a global fit at 141 different wavelengths over the range of λ = 597 to 306 nm). Selected binding isotherms are illustrated at λ_{abs} = 370 and 470 nm.

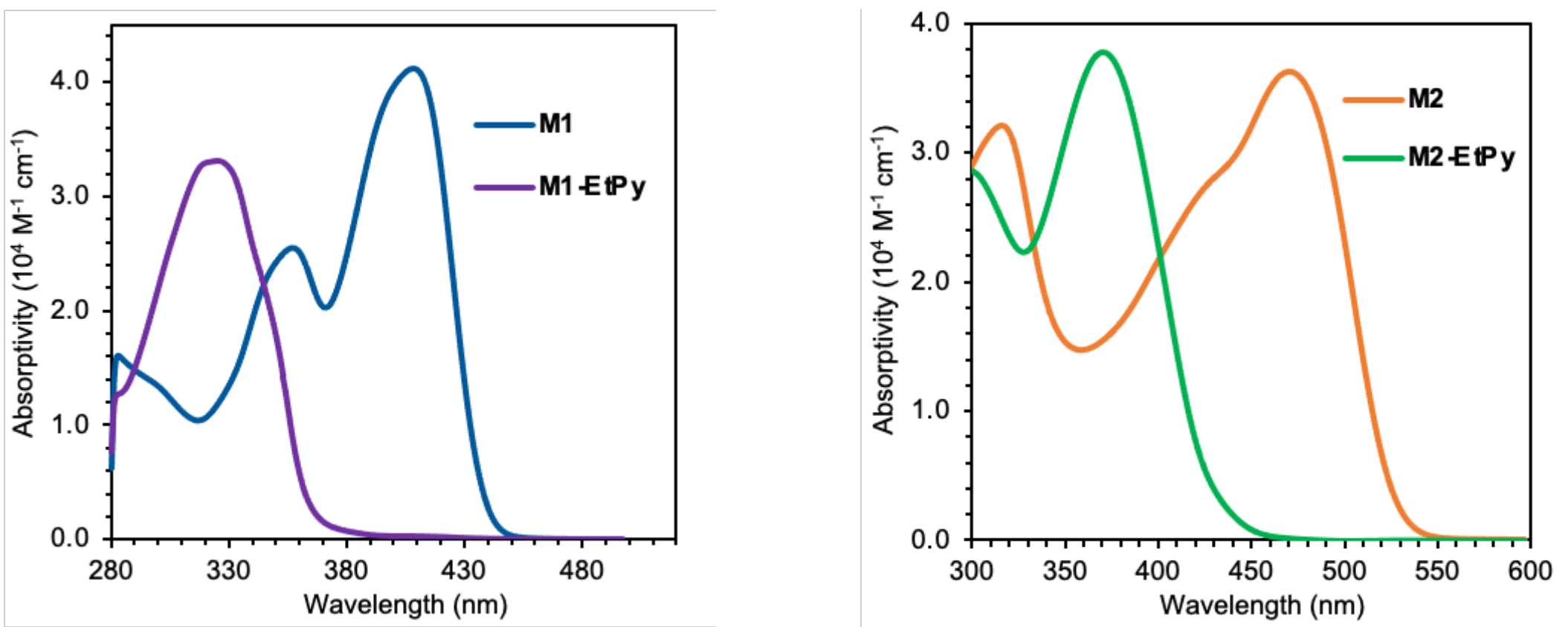

Figure S20. Calculated absorption spectra based on titration data fitting using the Hyperquad™ software of (left) **M1** (blue) and **M1-EtPy** (violet) and (right) **M2** (orange) and **M2-EtPy** (green) in toluene.

Table S10. Summary of binding equilibria (K_{eq}) and free energies (ΔG^o_{298K}) for Lewis pair complex formation determined by global data fitting using HypSpec™ software; computational data for reaction energies (ΔE_{DFT}) and enthalpies (ΔH_{DFT}) derived from DFT computations (B3LPY/6-31g(d)) are provided for comparison.

Compd	Lewis Base	K_{eq} (M^{-1})	lg K_{eq} (M^{-1})	ΔG^o_{298K} (kJ/mol)	ΔE_{DFT} (kJ/mol)	ΔH_{DFT} (kJ/mol)
**M1**	4-ethylpyridine	5.01 x 10^6	6.7	-38.2	-20.1	-19.8
**M2**	4-ethylpyridine	3.16 x 10^6	6.5	-37.1	-17.4	-17.2

6.2 VT-NMR Experiments and Eyring Plots

NMR samples of **M1-^{Et}Py** and **M2-^{Et}Py** with 1 additional equivalent of 4-ethylpyridine (**^{Et}Py**) were prepared in deuterated toluene at ca. 0.04 M concentration. The samples were initially analyzed on a 500 MHz Bruker spectrometer (499.9 MHz ^{1}H NMR) at 25 °C for adequate tuning, locking, and shimming. Samples were then brought to the desired temperatures, in the range of 0 °C to 50 °C for **M1-^{Et}Py** and 0 °C to 40 °C for **M2-^{Et}Py**, and further shimming was performed every 20 °C increments. Data consisted of 8 scans at each temperature. The resulting spectra containing the selected broadened LB peaks were analyzed by D-NMR line-shape analysis in Topspin software, using a model of the corresponding spin system. Concentrations of the species in equilibrium, coupling constants, and spectral linewidths were refined at -20 °C (253.15 K) in the slow exchange limit regime. These parameters were subsequently kept constant for the rest of the refinements. To obtain the rate constants, both the aromatic spin system in **^{Et}Py** (treated as a simple AB set of two doublets) and the ethyl groups were considered, and both were fit simultaneously. Thus, one set of averaged rate constants was obtained for each sample, and the resulting Eyring plot was fitted to calculate the activation energy at room temperature.

Table S11. Rate constants at corresponding temperatures.

T (K)	k_{M1} (s^{-1})	k_{M2} (s^{-1})
273.15	4.68	/
278.15	9.06	33.55
283.15	17.27	55.78
288.15	34.59	96.21
293.15	58.25	161.28
298.15	/	264.95
303.15	189.70	482.10
308.15	372.53	864.33
313.15	666.24	1228.32
318.15	1092.73	/
323.15	1986.08	/

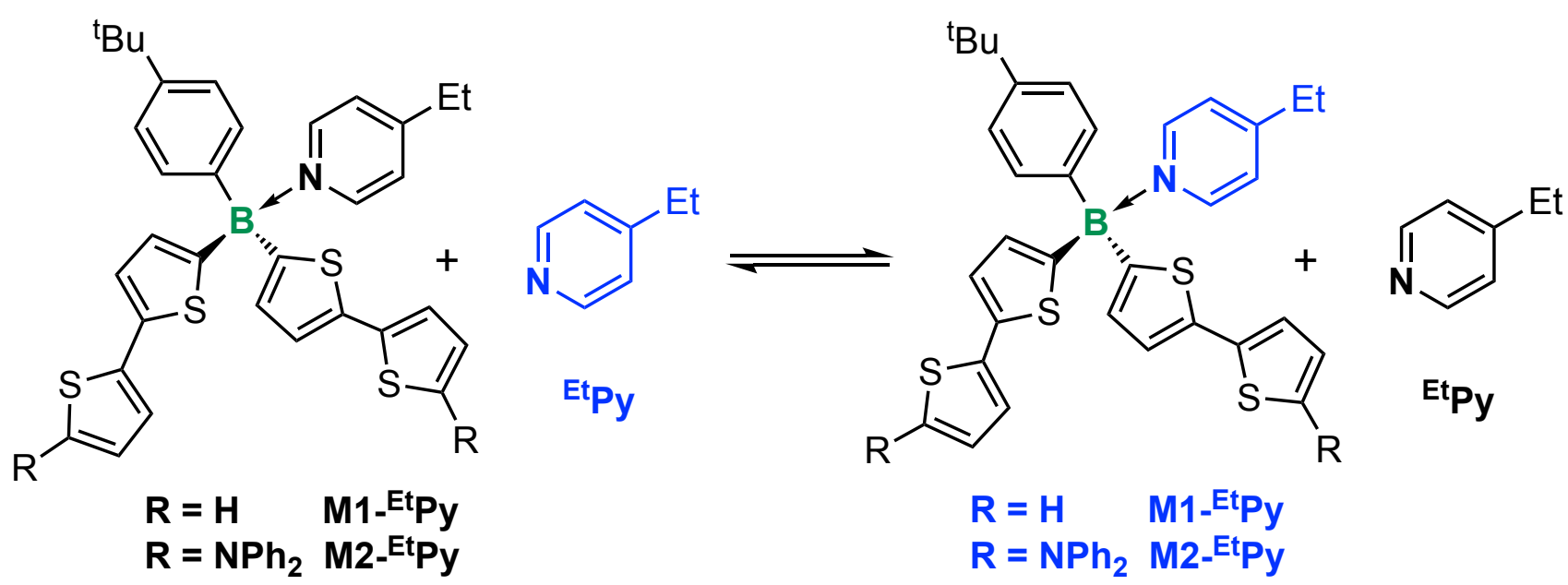

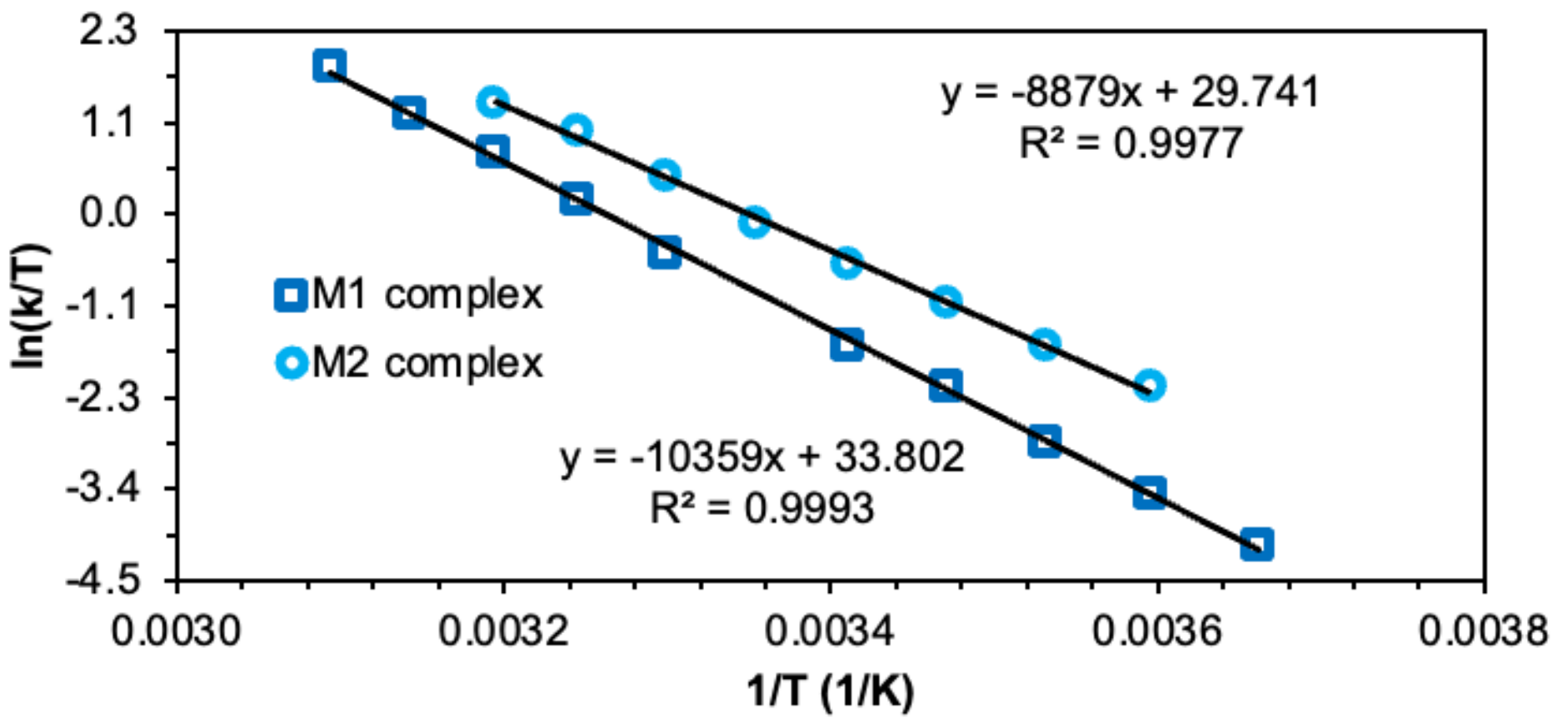

Figure S21. Eyring plot of the rate constants obtained by VT-NMR of the **M-^{Et}Py** + **^{Et}Py** exchange system.

The activation parameters were determined from the Eyring plots according to the following equation,

$$ln\left(\frac{k}{T}\right) = -\frac{\Delta H^{\neq}}{RT} + \frac{\Delta S^{\neq}}{R} + ln\left(\frac{k_B}{h}\right)$$

where R is the ideal gas constant (8.314 J mol^{-1} K^{-1}), k_B is the Boltzmann's constant (1.3806 × 10^{-23} J K^{-1}), T is the temperature (K), and h is the Planck's constant (6.6261 × 10^{-34} J s^{-1}). The activation parameters $\Delta H^{\neq}$and $\Delta S^{\neq}$ were obtained from the slope [$m = -\frac{\Delta H^{\neq}}{R}$] and the axis intercept [$n = \frac{\Delta S^{\neq}}{R} + ln\left(\frac{k_B}{h}\right)$]. Finally, the activation energy at room temperature was obtained from the equation: $\Delta G^{\neq}_{diss,298K} = \Delta H^{\neq} - T\Delta S^{\neq}$.

The dissociation rate constants at 25 °C were calculated according to:

$$k_{diss} = \frac{k_B T}{h} e^{\left(-\Delta G^{\neq}_{diss}/_{RT}\right)}$$

The Eyring's plot gave a linear fit for both complexes.

Table S12. Calculated activation parameters for the model complexes.

	$\Delta H^{\ddagger}$ (kJ/mol)	$\Delta S^{\ddagger}$ (J/mol•K)	$\Delta G^{\ddagger}_{diss,298K}$ (kJ/mol)	$k_{diss,298K}$ (s^{-1})
**M1-^{Et}Py**	86.4	83.5	61.5	103.0
**M2- ^{Et}Py**	73.8	49.7	59.0	286.7

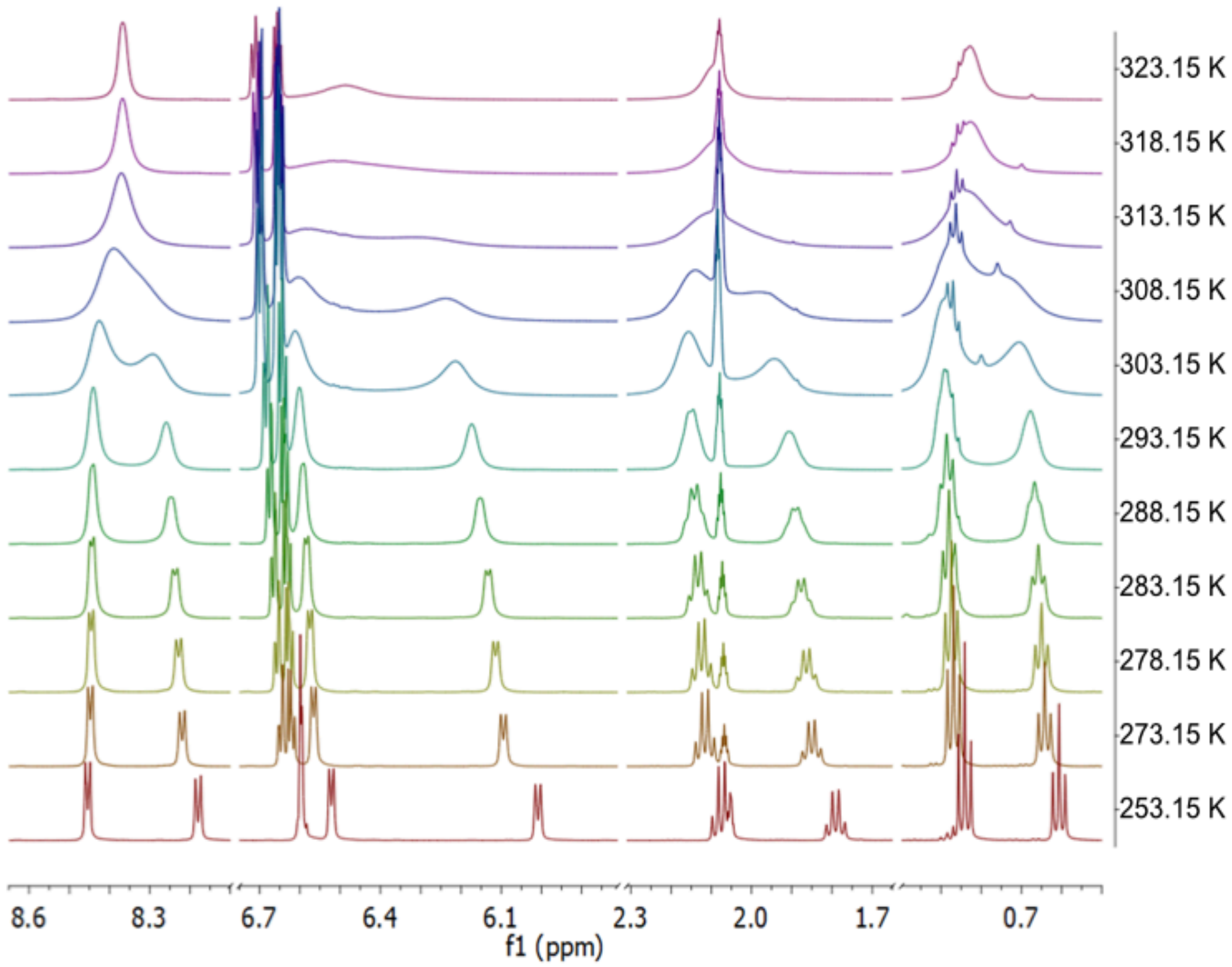

Figure S22. ^{1}H NMR spectra of an equimolar mixture of **M1-^{Et}Py** + **^{Et}Py** in d[8]-toluene at variable temperatures.

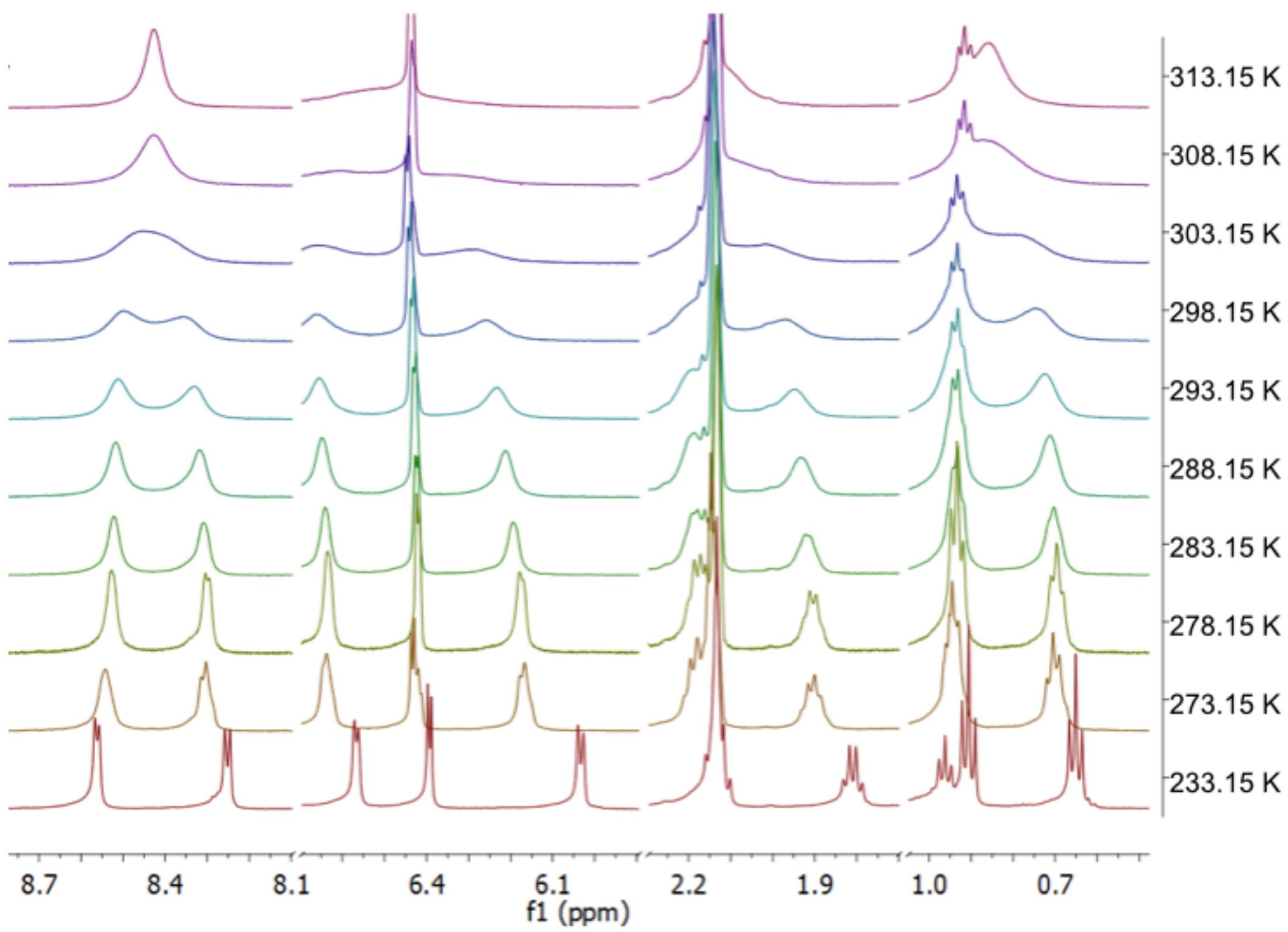

Figure S23. ^{1}H NMR spectra of an equimolar mixture of **M2-^{Et}Py** + **^{Et}Py** in d[8]-toluene at variable temperatures.

6.3 Simulation of B-N Bond Breaking for Model Compounds M1-Py and M2-Py

The breaking of the B-N bonds was simulated by redundant coordinate calculations. The distance between anchor atom [1] and [2] (indicated in light blue) was increased over 15 steps by a total of 1.5 Å for both **M1-Py** and **M2-Py**. Optimizing the structure at each step yielded a stepped energy diagram as a function of distance. Sharp transitions were observed at 1.3 Å, reflecting dramatic geometric changes upon breaking the B-N bond. The results verified that small deformations of the complex structure induced by targeted separation of anchor points can lead to sudden B-N bond cleavage.

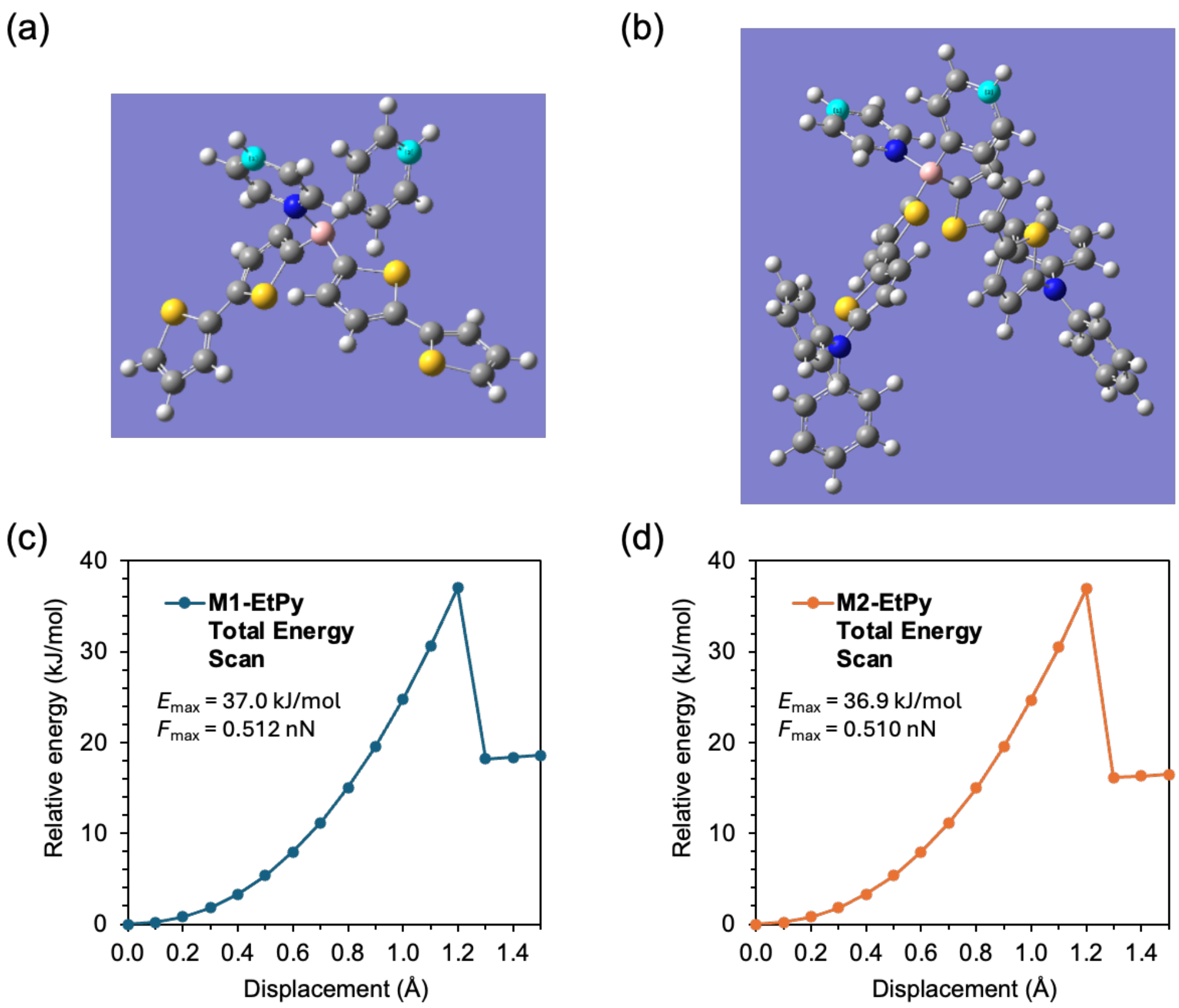

Figure S24. Starting geometries of (a) **M1-Py** and (b) **M2-Py** used to simulate B-N bond breaking, with the anchor atoms indicated in light blue. Plots indicating the total energy as the distance between the anchor points was gradually increased over 15 steps by a total of 1.5 Å for (c) **M1-Py** and (d) **M2-Py**.

7. Polymer Network Formation and Characterization

7.1 Preparation of Polymer Networks

High molecular weight polydimethylsiloxane with pendant pyridine groups (**PDMS-Py**) was chosen as the polymer-supported Lewis base for constructing the Lewis pair-crosslinked polymer networks. **PDMS-Py** was synthesized by thiol-ene functionalization of vinyl-substituted PDMS as previously described.[2]

GPC *vs* PS standards (THF, 1 mL min^{-1}): M_n = 409 kDa, $Đ = M_w/M_n$ = 1.48; when assuming 0.50 mol% functional groups DP_n = 5,454; n/m = 199:1; n = 5427, m = 27.3 based on GPC and ^{1}H NMR analysis.

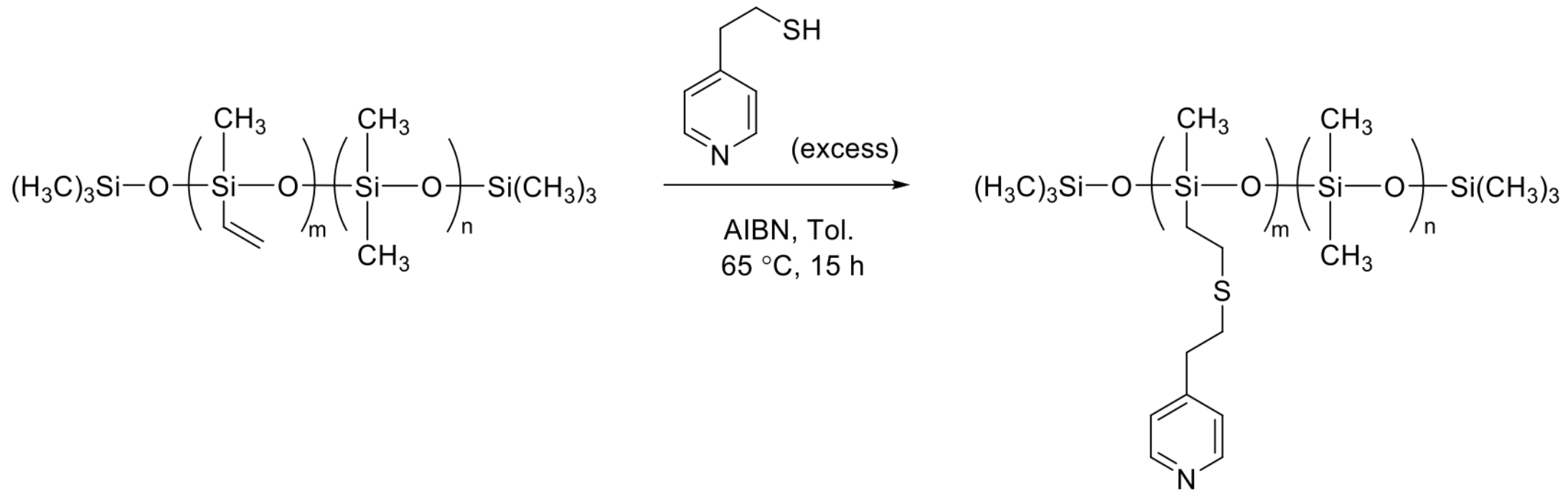

Table S13. Characteristics of Lewis acid- and Lewis base-functionalized polymers employed in the formation of PS/PDMS network materials.

Polymer code	Main chain	Funct. Group (*F*)	M_n [a] (kDa)	$Đ$ [a]	*F* [b] (mol%)	Avg no. of *F* per chain[c]	T_m [d] (°C)	T_g [d] (°C)	T_d [e] (°C)
**PDMS-Py**	PDMS	4-Py	409	1.48	0.50	27.3	−44.4	n.d.	n.d.
**PS1-^{Et}Py** [f]	PS	$B(Th_2)_2$	21.0	1.19	0.11	15.0	–	97.0	142.4 [g]
**PS2-^{Et}Py** [f]	PS	$B(Th_2NPh_2)_2$	23.0	1.25	0.11	13.4	–	101.7	145.3 [g]
**F1**	PDMS-PS	$B(Th_2)_2$	-	-	-	-	-	n.d.	482.2
**F2**	PDMS-PS	$B(Th_2NPh_2)_2$	-	-	-	-	-	n.d.	502.6

[a] M_n determined by GPC in THF against PS standards. [b] Determined from the ^{1}H NMR spectra of the isolated polymers. [c] Calculated from the M_n and F mol%. [d] T_m and T_g determined by DSC during the second heating scan (10 °C min^{-1}). [e] Onset determined by TGA (10 °C min^{-1}); (n.d. = not determined). [f] Measured after the addition of 4-ethylpyridine to samples of **PS1** and **PS2**. [g] Onset of decomposition attributed to loss of 4-ethylpyridine.

To prepare the polymer networks, inside a N_2-filled glovebox, a scintillation vial was charged with a large magnetic stir bar. **PDMS-Py** (105 mg, 0.0070 mmol of pyridyl groups) and a stoichiometric amount of **PS1** (8.6 mg, 0.0067 mmol of boron groups) or **PS2** (11.1 mg, 0.0069 mmol of boron groups) were weighed into the vial. 10 mL of anhydrous toluene were then added, and the mixture was stirred for 4 hours. Once the borylated polymer was fully dispersed within the PDMS gel, the vial was capped and transferred to an oil bath at 70 °C for 24 hours (with very gentle stirring) to allow network equilibration. After equilibrating at 70 °C, a homogenous organogel was observed. Finally, the vial was transferred to a glovebox, where the polymer gel was poured, still warm, into a flat-bottom Teflon mold (folded from Teflon sheets). The solvent was allowed to slowly evaporate undisturbed for 1 week. Free-standing polymer network films of **F1** and **F2** networks (derived from **PS1** and **PS2**, respectively) were peeled off the mold and further dried under high vacuum for at least 24 hours. The polymer

films were cut into disks for rheology tests and into rectangular shapes for tensile tests. Smaller specimens were used for thermogravimetric analysis and swelling tests (*vide infra*).

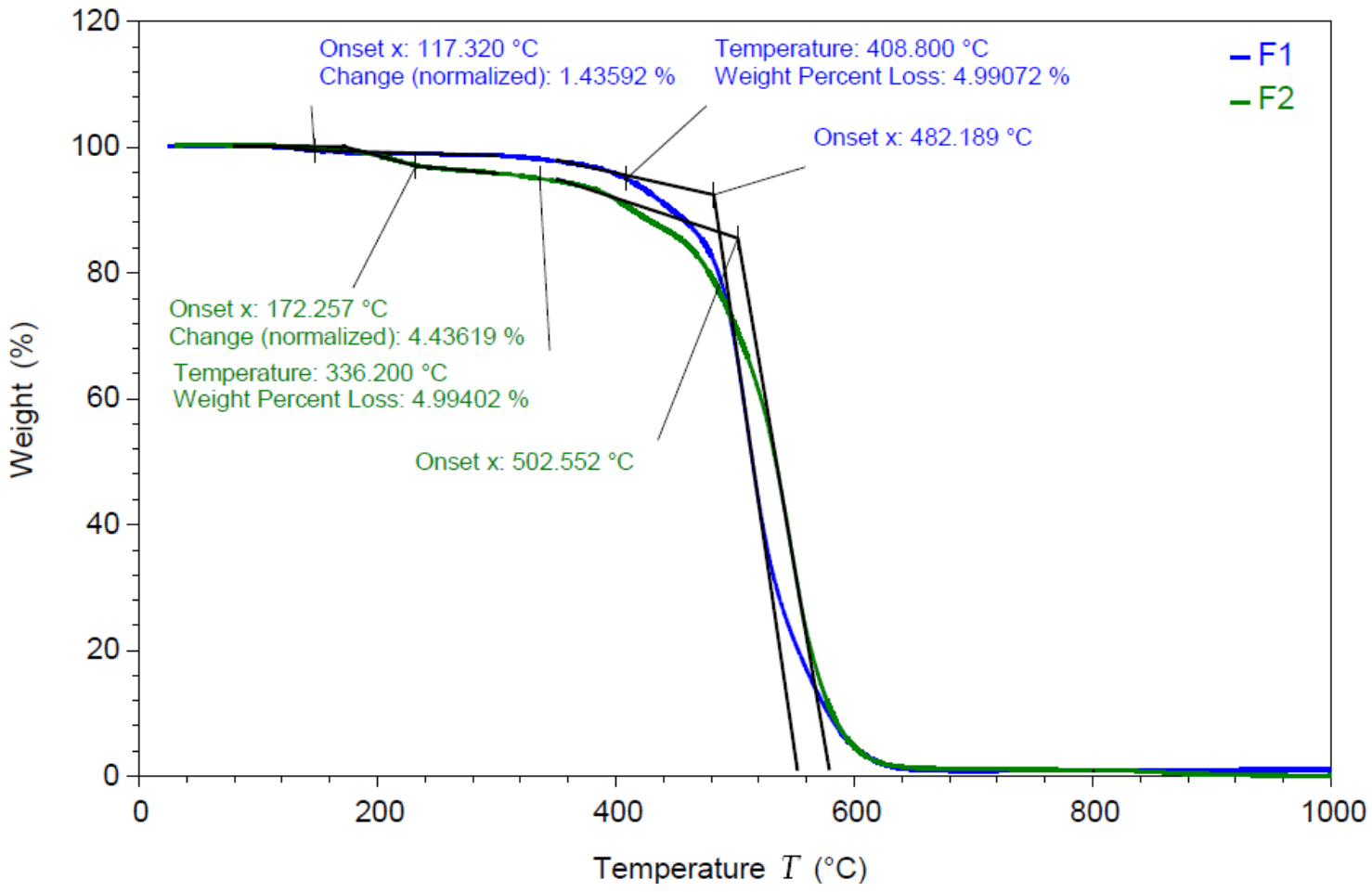

Figure S25. Thermogravimetric analyses (TGA) of polymer networks **F1** and **F2** at a heating rate of 10 °C min^{-1}. Temperatures at 5% weight loss were 409 °C and 336 °C for **F1** and **F2**, respectively.

7.2 Tensile Tests

Stress-strain curves for the polymer networks were recorded with 60% strain increment per minute (Figure S26a). Force ramps were performed at 1 N/min until the specimen reached the maximum length of the apparatus (~28 mm). The calculated stiffness of **F1** was higher than that of **F2**, suggesting more effective crosslinking for **F1**.

Stress-relaxation curves were recorded by holding the specimen at 300% strain over 10 minutes, and the corresponding stress values were recorded (Figure S26b). The data show a slightly larger relative decrease in stress over 10 minutes for **F1** (ca. 32% reduction) than for **F2** (ca. 22% reduction), most likely because the **F1** specimen's initial stress was higher; nevertheless, the **F1** stress remained higher than that of **F2**.

Creep tests were performed by holding the specimen at 0.1 N and recording the strain values over 20 minutes. They further confirmed that network **F2** was softer compared with **F1**, showing larger deformation over the same time scale (Figure S26c).

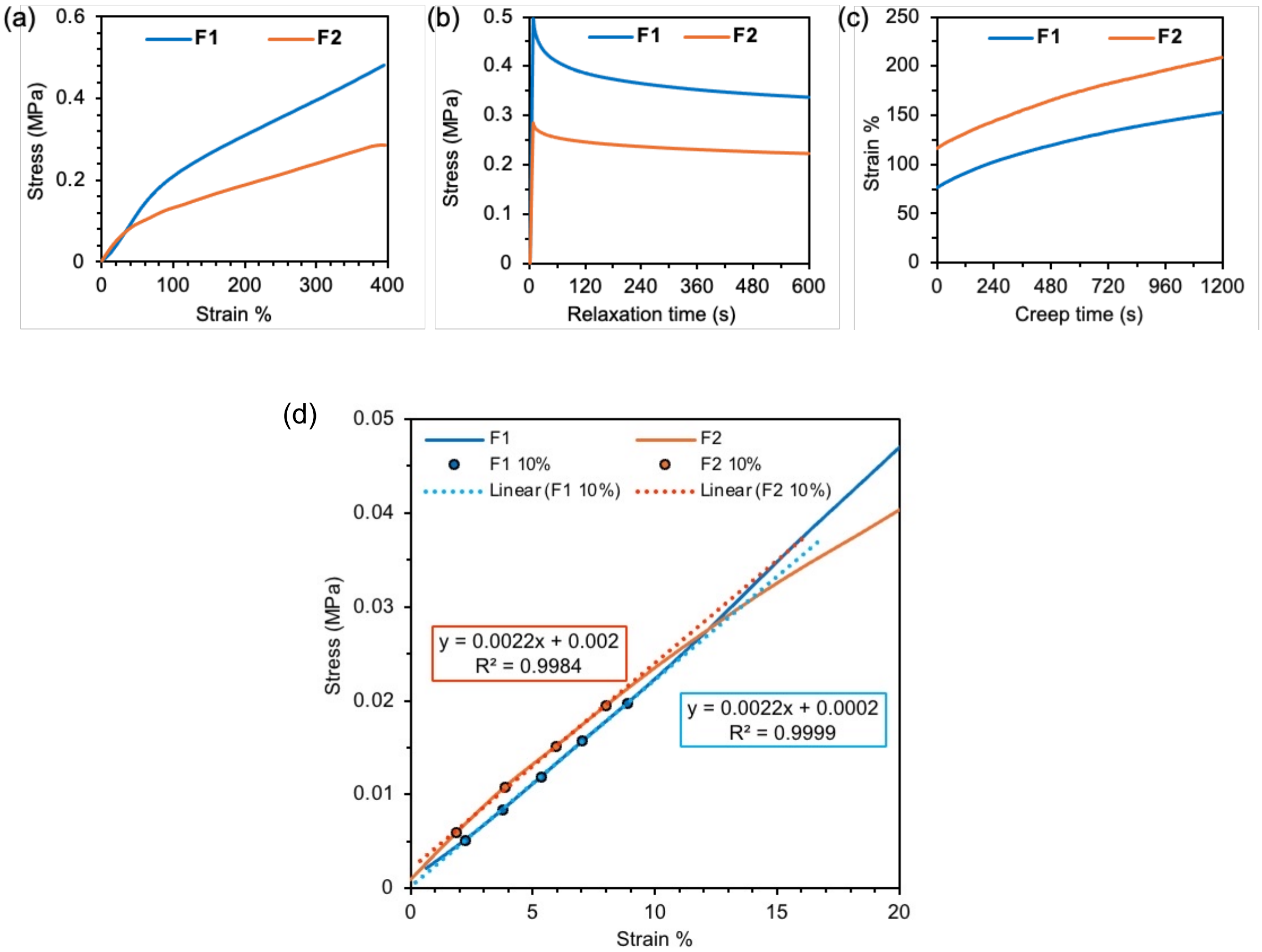

Figure S26. (a) Stress-strain curves, (b) stress relaxation curves, (c) creep tests, and (d) determination of Youngs modulus for elastomers **F1** and **F2**.

7.3 Rheology Measurements

Rheology measurements were performed as described in the general experimental section.

Oscillatory Frequency Sweeps

Oscillatory frequency sweeps confirmed that the polymer specimens obtained were crosslinked, as there was little change in the storage modulus across the frequency range, and there was no crossover of the loss modulus (**G”**) and storage modulus (**G’**) for either polymer specimen (Figure S27). **G’** for **F1** was slightly higher than **G’** for **F2**, suggesting that the crosslink density of **F1** was higher than that of **F2**, which may be related to the difference in exchange kinetics of the B-N Lewis pairs.

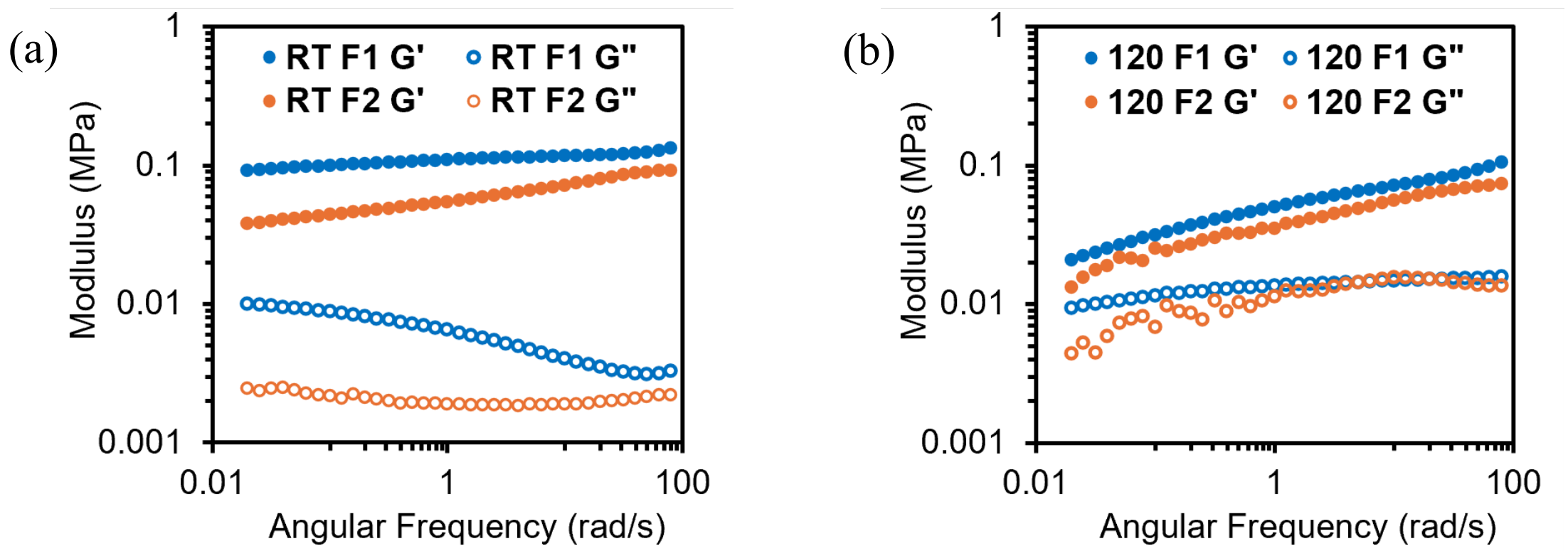

Figure S27. Frequency sweeps at (a) room temperature and (b) 120 ºC showing changes in the storage (**G’**) and loss (**G”**) modulus.

Master Curves

Master curves were generated by TTS (as described in the general experimental section), showing that the **G’** value of **F1** was higher than that of **F2** also across the extended frequency range (Figures S28-S29). For each sample, the storage modulus was higher than the loss modulus, demonstrating that the B-N crosslinks were retained at the reference temperature of 65 ºC. As described in the methods section, samples were tested at elevated temperatures for the purpose of studying the thermostability of the B-N bonds. The results can be compared on the condition that the testing specimens were of the same thickness and contained (theoretically) a similar number of crosslinks.

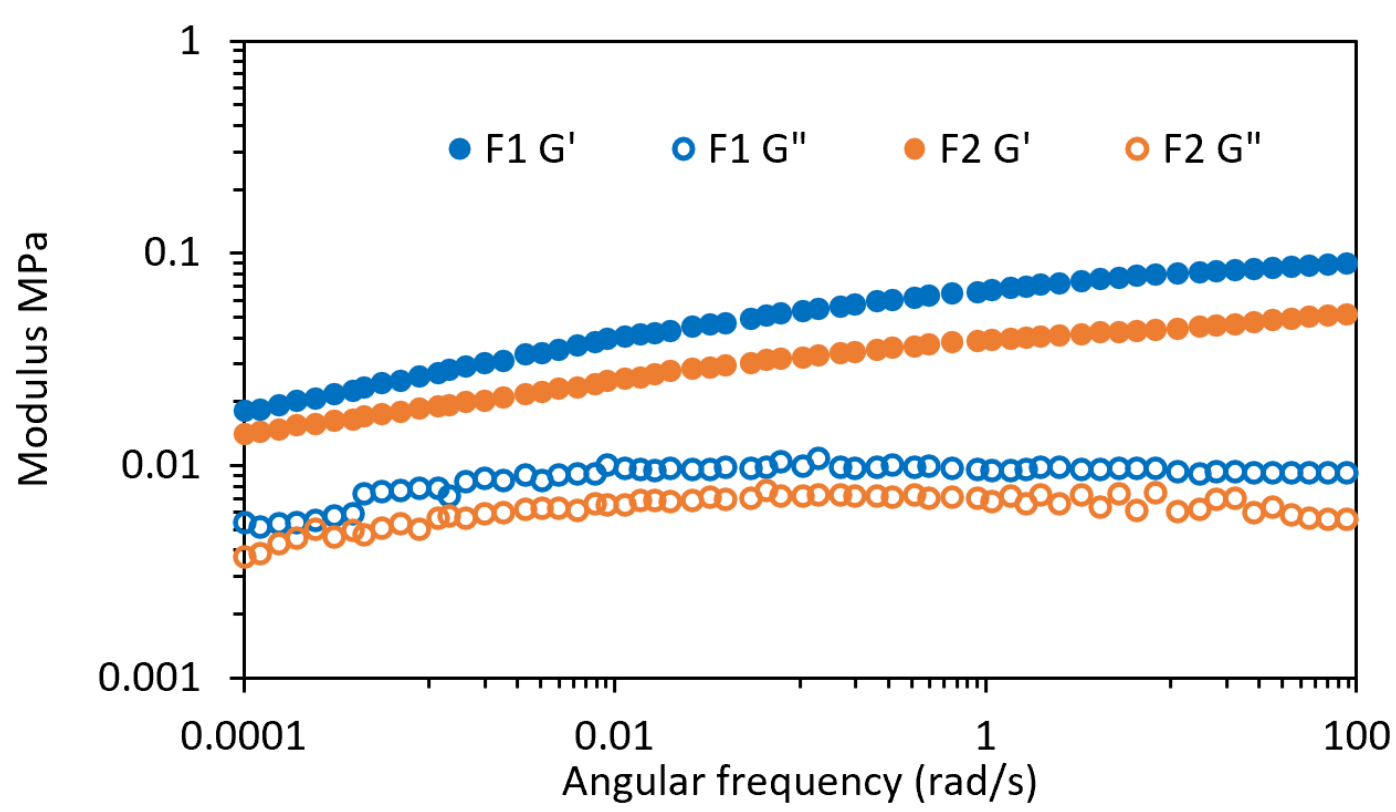

Figure S28. Master curves of **G’** and **G”** for **F1** and **F2** referenced at 65 °C.

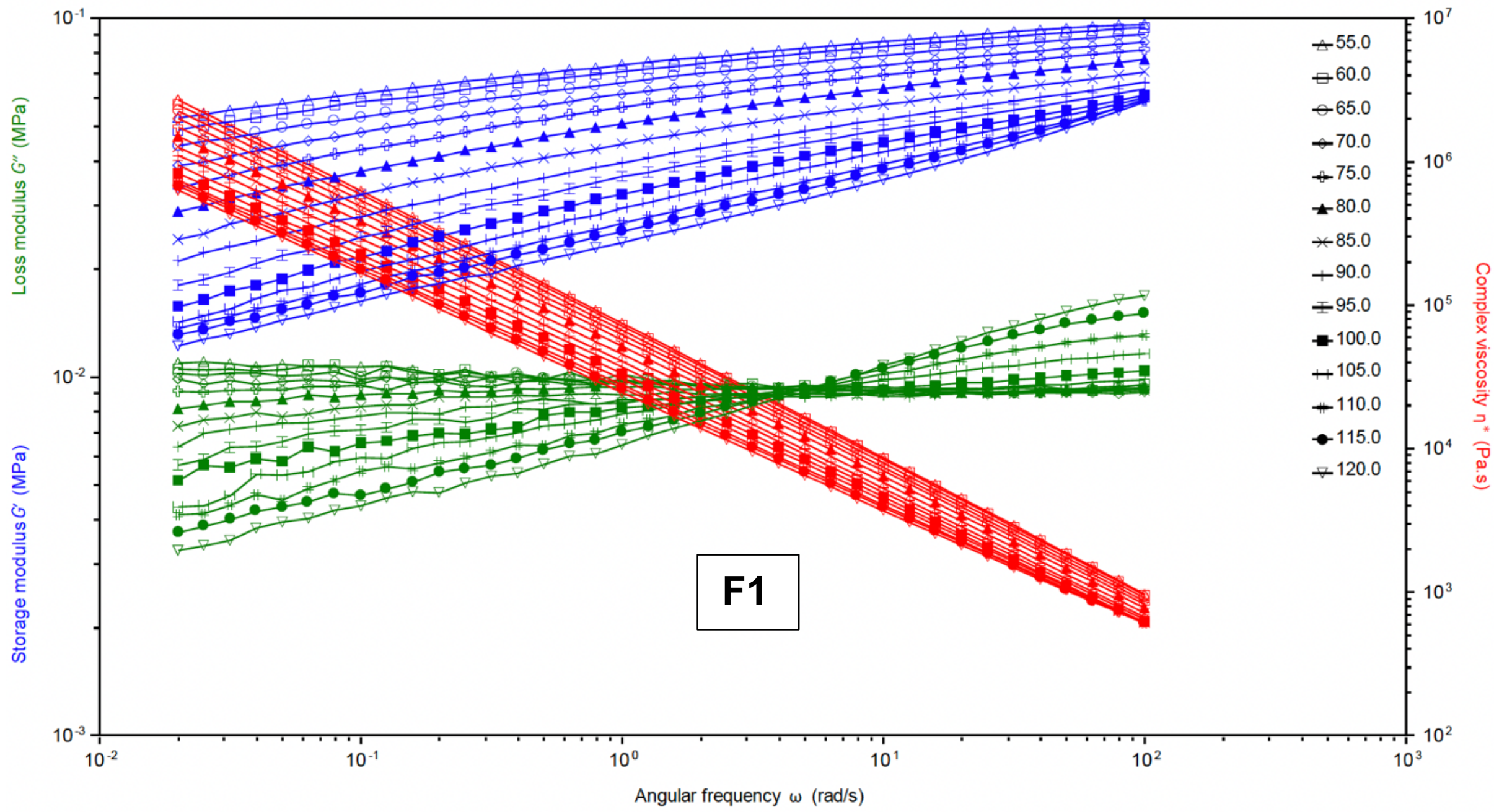

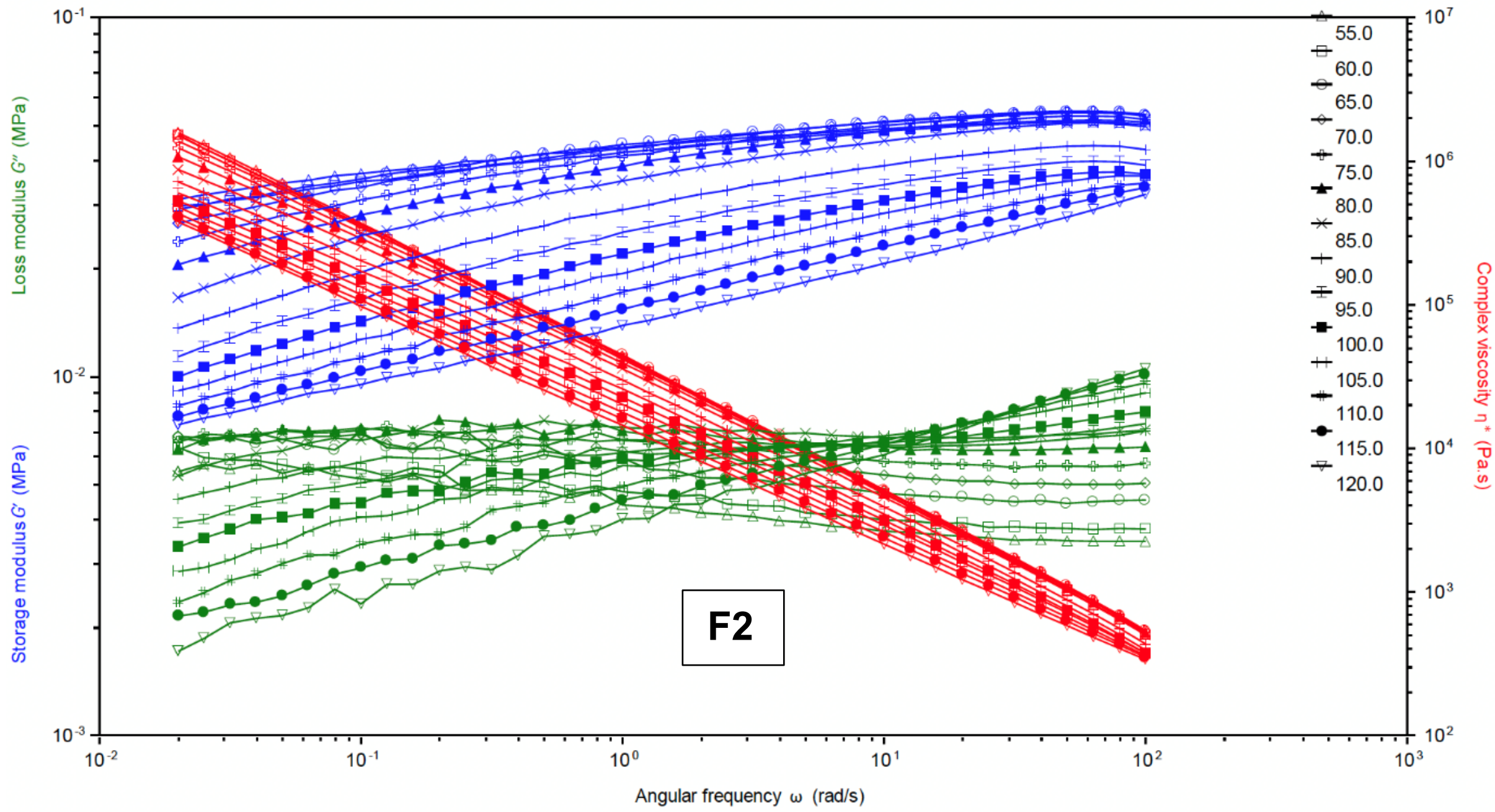

Figure S29. Oscillatory frequency sweeps (1% strain) of **F1** (top) and **F2** (bottom) employed in the creation of TTS master curves.

8. Mechanofluorochromic Properties of Polymer Networks

Videos were recorded during the stretching of **F1** and **F2** specimens (20.0 mm × 8.0 mm × 0.8 mm) under irradiation with a handheld UV lamp at 365 nm. Videos were taken with 30 frames per second. Layers were extracted every 5 frames (0.167 seconds) and analyzed with Adobe Photoshop. Fluorescence changes were analyzed by averaging RGB, HSU, or CMYK values at different positions on the specimens. The sample elongation in each frame was estimated by counting pixels along the force axis, and the corresponding strain was calculated relative to zero force (Tables S14-S16). Three data points were selected on the specimen and the data averaged for a more accurate analysis of the intensity/color changes (Figure S30). Multiple analyses consistently showed changes at Site 1 to be the fastest and most pronounced. On the other hand, Site 3 gave the least pronounced changes in intensity or color. The results qualitatively describe the stress distribution across the specimen under tensile force.

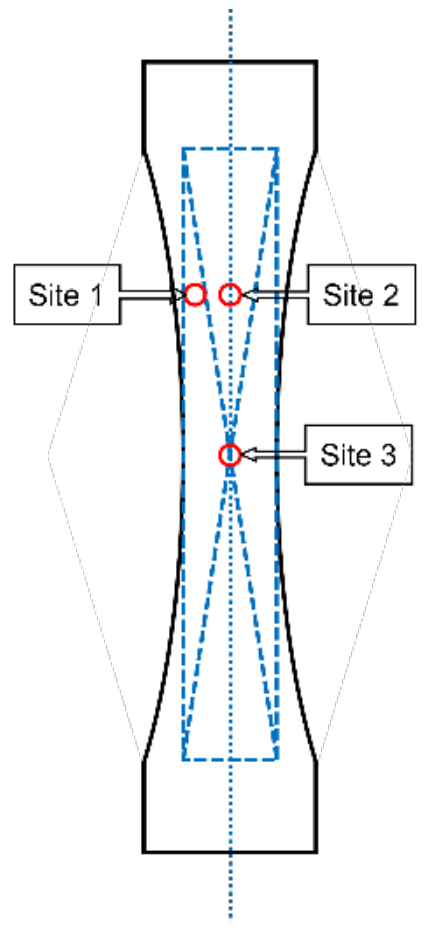

Figure S30. Schematic illustration of sampling sites for specimen. Crosshair was applied in Photoshop with the Frame tool for more accurate pixel measurements.

For **F1**, the blue fluorescence became more intense after stretching the sample to 3 times its original length. Based on the RGB values, the color (blue) did not change significantly, but the intensity increased significantly. To better quantify the intensity change, a Black-White High-contrast-Blue filter was applied to the video frames. The filter converts all RGB values to the HSU scale. Because the samples were transparent, the increase in Hue value was corrected by considering the decrease in sample thickness. The thickness factor was calculated using the equation (1+strain)^-0.45 and an estimated Poisson's ratio of 0.45. The averaged increase in emission intensity relative to strain ratio for **F1** was plotted and fit to an exponential function (see Figure 7 in the manuscript). This can be rationalized by considering that the tensile force should align the chain segments in its direction, slowing energy relaxation. Consequently, more stress is exerted directly on the B-N crosslinks, and upon breaking the B-N bonds, the luminescence of the tricoordinate borane fluorophores is restored. The fluorescence turned off immediately upon release of the stress. This is attributed to the low T_g of the PDMS matrix, which enables rapid chain movement and reformation of B-N Lewis pairs. The intensity changes for all data points were plotted, as well as the averaged value (Figure S31a), all showing exponential trends.

A video of the decrease in **F1** emission intensity during the stress-relaxation stage was recorded and analyzed in a similar manner. An **F1** specimen (20.0 mm x 6.2 mm x 0.8mm) was loaded onto tensile clamps on the DMA Q800 and manually stretched over 15 seconds to maximum strain for the apparatus (Figure S32a). Because the stretched sample had to be manually locked before the video of the sample was recorded for 20 minutes (Figure S32b), a 2-minute gap was

inevitable between the video recording of the stretching (a) and stress-relaxation (b) stages shown in Figure S31. For this reason, the emission intensity at maximum strain during the stretching stage was higher than that at the 0 s time point during the stress-relaxation. The decrease in Hue values at each data site were plotted against time with the averaged Hue value for a comparison of decay trends at each site (Figure S31b).

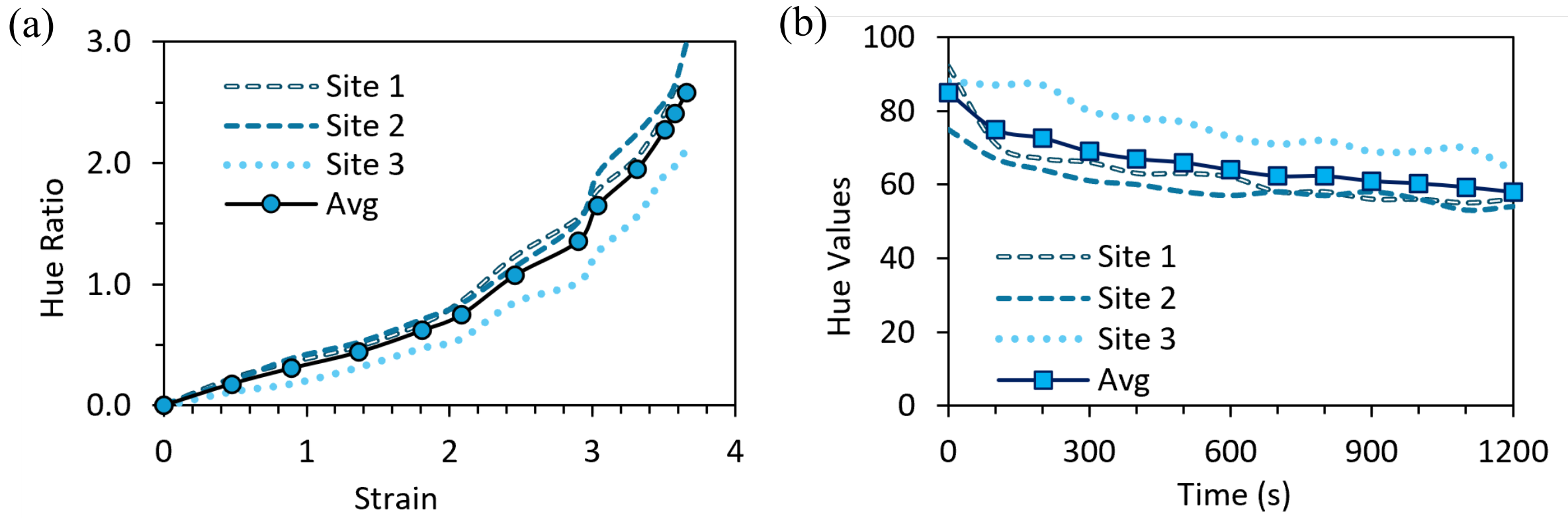

Figure S31. (a) Increase in adjusted Hue ratio for 3 sites and averaged values. (b) Decrease in Hue values for 3 sites and averaged values.

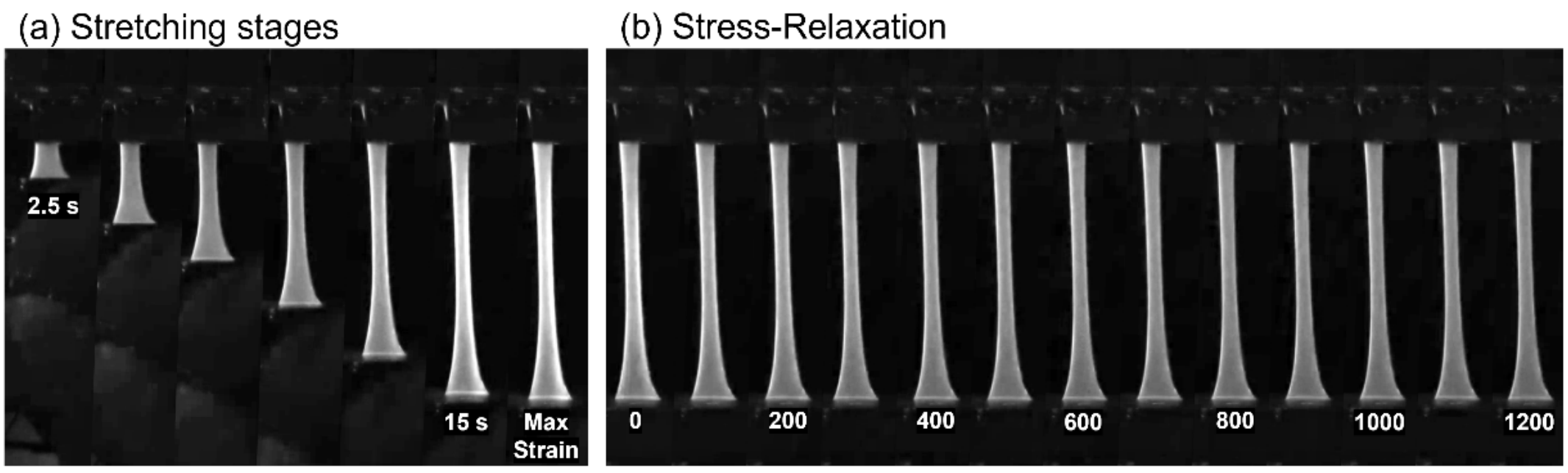

Figure S32. Video frames of the mechanofluorochromic properties of **F1** during stress relaxation.

For **F2**, the emission of the specimen was blue-green in color with no tensile load and gradually turned to yellow after stretching beyond 200% (see Figure 7 in the manuscript). The RGB values at each data point were traced and plotted to visualize the differences in mechanochromic response for different positions on the sample (Figure S33). As discussed before, under the same strain Site 3 showed the least color change. Initially, for low deformation samples, a blue-green color indicates the presence of blue-emissive tetracoordinate borane complexes as well as a certain fraction of yellow-emissive tricoordinate organoborane moieties. The yellow component is likely enhanced by fluorescence resonance energy transfer (FRET) processes throughout the polymer network. The CMYK values were derived from the averaged RGB numbers. By tracking the CMYK values of the sample, it was observed that the Magenta component remained stable, while the yellow component increased and the cyan component decreased. The absolute change in the values against strain at ratios beyond 200% followed approximately a linear fit. This indicates that the segmental chain relaxation dissipates tensile load; hence, only a fraction of the B-N bonds is broken at small deformations. As the chains are extended further, the breaking of B-N bonds becomes inevitable, and the yellow-emissive tricoordinate borane fluorophores are restored. This strain

threshold was more evident for the **F2** networks, possibly because of weaker B-N bonds, which allow the polymer segments greater mobility for energy dissipation.

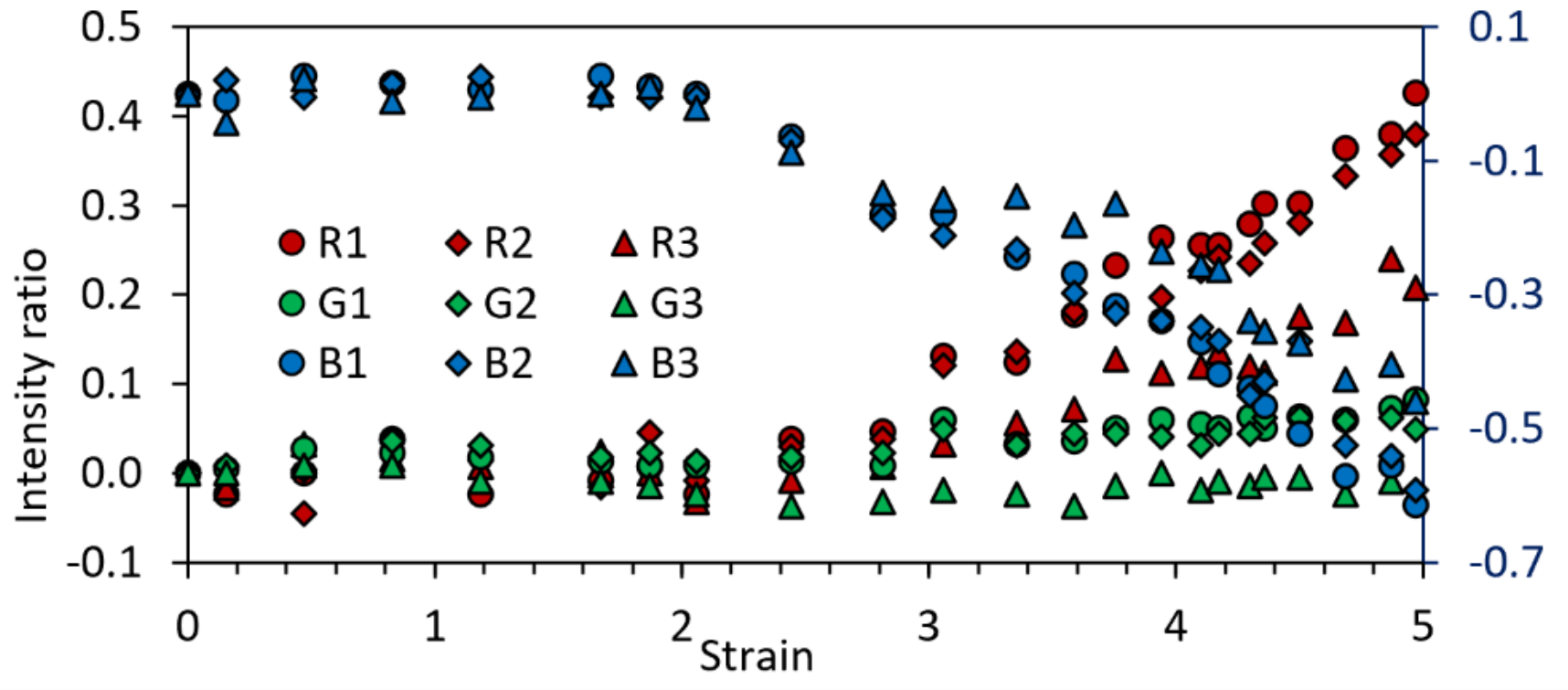

Figure S33. Changes in RGB values at 3 sites. The decrease in blue color was plotted on the secondary axis.

Table S14. Analysis of the emission of **F1** during the stretching of the specimens

			Hue values								Adjusted Hue			ΔH/H		
Data Point	Length	Width	Site 1	Site 2	Site 3	Avg	Thickness factor	Adjusted H	Strain	Avg	Site 1	Site 2	Site 3	Site 1	Site 2	Site 3
0	158	106	116	120	119	118	1.0	118	0.0	0.0	116	120	119	0.0	0.0	0.0
1	233	82	119	120	112	117	0.8	139	0.5	0.2	142	143	133	0.2	0.2	0.1
2	299	79	118	124	106	116	0.8	155	0.9	0.3	157	165	141	0.4	0.4	0.2
3	374	73	117	123	107	116	0.7	170	1.4	0.4	172	181	158	0.5	0.5	0.3
4	444	72	122	128	111	120	0.6	192	1.8	0.6	194	204	177	0.7	0.7	0.5
5	487	66	130	132	112	125	0.6	207	2.1	0.7	216	219	186	0.9	0.8	0.5
6	546	65	148	146	127	140	0.6	245	2.5	1.1	259	255	222	1.2	1.1	0.8
7	616	64	160	162	131	151	0.5	279	2.9	1.4	295	299	242	1.5	1.5	1.0
8	638	62	172	185	145	167	0.5	314	3.0	1.7	322	347	272	1.8	1.9	1.3
9	681	62	183	200	159	181	0.5	349	3.3	1.9	353	386	307	2.0	2.2	1.6
10	712	62	201	212	177	197	0.5	387	3.5	2.3	396	417	348	2.4	2.5	1.9
11	723	62	211	219	180	203	0.5	403	3.6	2.4	418	434	357	2.6	2.6	2.0
12	736	62	211	238	187	212	0.5	424	3.7	2.6	422	476	374	2.6	3.0	2.1

Table S15. Analysis of the emission of **F1** during relaxation of the specimen.

					Decay			
Layers	Time	Site 1	Site 2	Site 3	Avg	ΔH	% Decrease	ΔH normalized
1	0	92	75	88	85	0	0.00	0.00
7	100	71	67	87	75	10	11.76	0.37
13	200	67	64	87	73	12	14.51	0.46
19	300	66	61	80	69	16	18.82	0.59
25	400	63	60	78	67	18	21.18	0.67
31	500	63	58	77	66	19	22.35	0.70
37	600	62	57	73	64	21	24.71	0.78
43	700	58	58	71	62	23	26.67	0.84
49	800	58	57	72	62	23	26.67	0.84
55	900	56	58	69	61	24	28.24	0.89
61	1000	56	56	69	60	25	29.02	0.91
67	1100	55	53	70	59	26	30.20	0.95
73	1200	56	54	64	58	27	31.76	1.00

Table S16. Analysis of the emission for **F2** during the stretching of the specimens

Length	Strain	R1	G1	B1	R2	G2	B2	R3	G3	B3	avg R	avg G	avg B	C	M	Y	K	C %	Y %
70	0.0	129	219	189	132	223	198	125	218	183	129	220	190	41	0	14	14	0.00	0.00
81	0.2	126	220	187	133	225	202	123	218	175	127	221	188	43	0	13	12	0.05	-0.07
103	0.5	129	225	194	126	224	197	129	220	187	128	223	193	43	0	15	12	0.05	0.07
128	0.8	134	224	192	137	231	201	127	220	181	133	225	191	41	0	15	13	0.00	0.07
153	1.2	126	223	190	134	230	203	126	216	182	129	223	192	42	0	14	13	0.02	0.00
187	1.7	128	222	194	130	227	197	128	216	183	129	222	191	42	0	14	13	0.02	0.00
201	1.9	130	221	191	138	228	197	125	215	185	131	221	191	41	0	14	13	0.00	0.00
214	2.1	126	221	189	131	226	197	121	213	179	126	220	188	43	0	15	13	0.05	0.07
241	2.4	134	222	177	136	227	184	124	210	167	131	220	176	40	0	20	9	-0.02	0.43
267	2.8	135	221	155	137	228	161	126	211	156	133	220	157	40	0	29	11	-0.02	1.07
284	3.1	146	232	155	148	234	156	129	214	154	141	227	155	38	0	32	11	-0.07	1.29
305	3.4	145	226	143	150	230	152	132	213	155	142	223	150	36	0	33	10	-0.12	1.36
321	3.6	152	227	138	156	233	139	134	210	147	147	223	141	34	0	37	9	-0.17	1.64
333	3.8	159	230	129	156	233	133	141	215	153	152	226	138	33	0	39	9	-0.20	1.79
346	3.9	163	232	125	158	232	131	139	218	140	153	227	132	33	0	42	10	-0.20	2.00
362	4.2	162	230	110	164	233	125	142	216	135	156	226	123	31	0	46	9	-0.24	2.29
385	4.5	168	233	93	169	237	125	147	217	115	161	229	111	30	0	52	10	-0.27	2.71
398	4.7	176	232	81	176	236	94	146	213	105	166	227	93	27	0	59	9	-0.34	3.21
411	4.9	178	235	84	179	237	91	155	216	109	171	229	95	25	0	59	9	-0.39	3.21
418	5.0	184	237	73	182	234	81	151	213	99	172	228	84	25	0	63	7	-0.39	3.50

9. Reversibility of B-N Lewis Pair-Based Polymer Network Formation

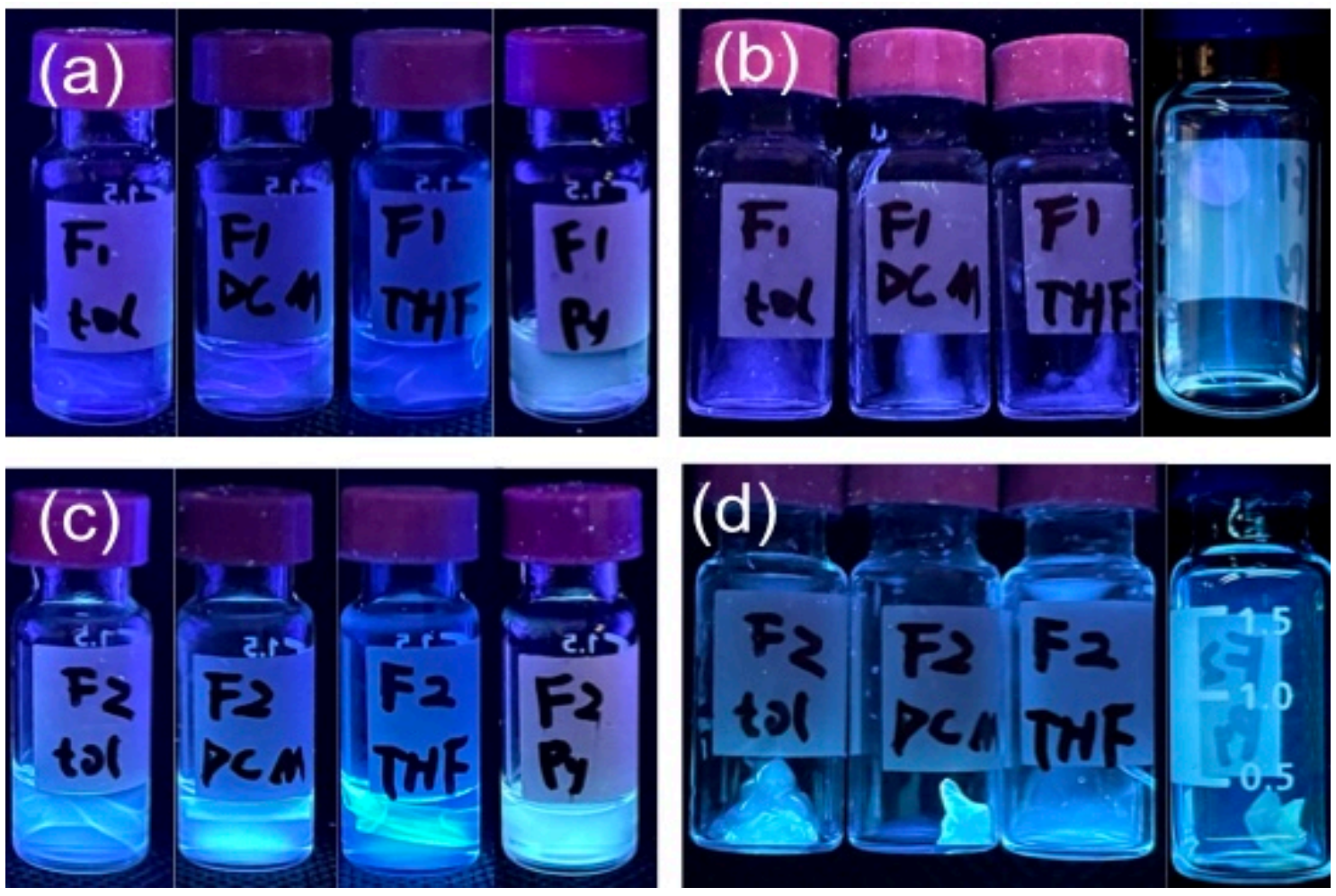

Figure S34. Photographs of network swelling tests in different solvents. (a) **F1** in toluene, DCM, THF, and pyridine after 2 days. (b) **F1** gel residue after 5 days (liquids were decanted for clarity). (c) **F2** in corresponding solvents after 2 days. (d) **F2** gel residue after 5 days (liquids were decanted for clarity).

NMR-scale experiments with BF_3•OEt_2 as a competing Lewis acid were performed on bulk polymer films. Film samples were taken in dry C_6D_6 (10 mg in 0.5 mL) and stored in valved NMR tubes for 1 week to ensure the polymer networks were fully swollen (Figure S35a). Two equivalents of BF_3•OEt_2 per B-N unit were added to each sample. The number of B-N units was estimated assuming that 10 mg of the sample contained 92 wt% PDMS and 8 wt% PS. The color and consistency of the swollen gels changed instantly (Figure S35b,c), and after 5 days, the gels were fully disassembled (Figure S35d).

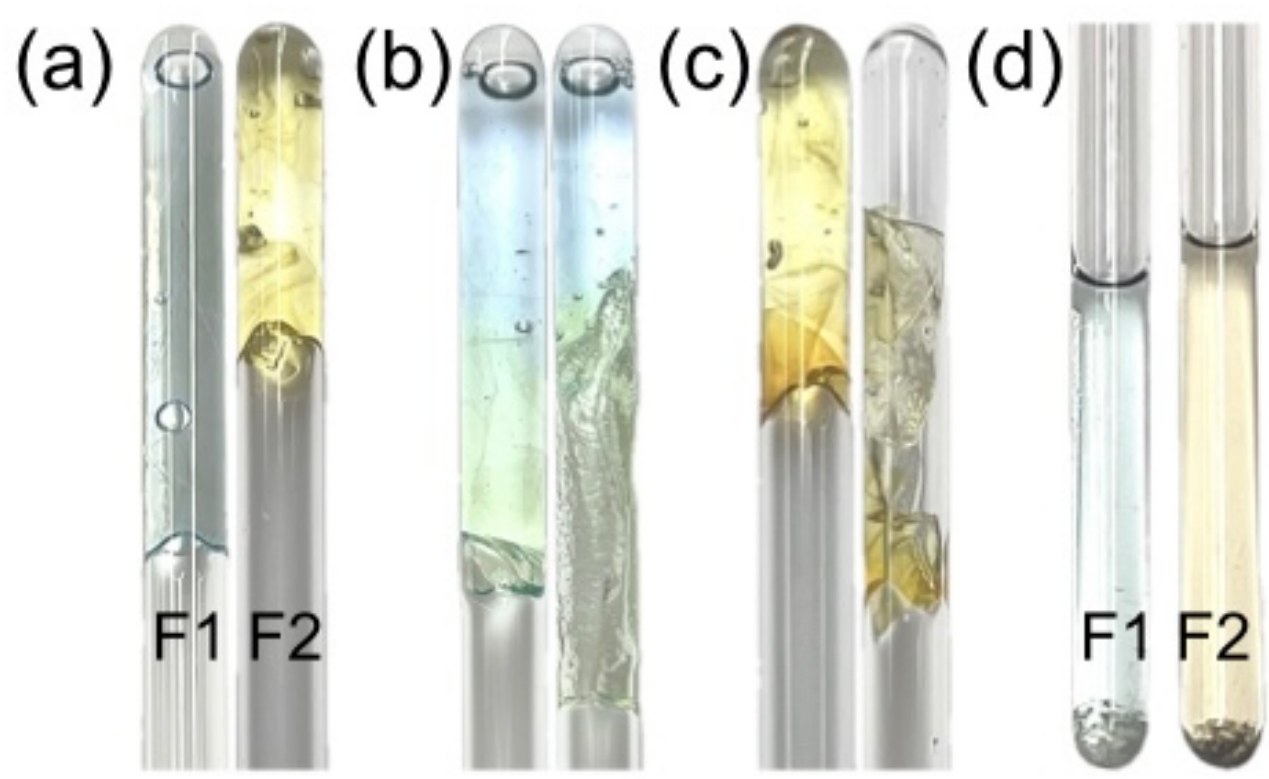

Figure S35. Photographs of (a) pristine swollen gels of **F1** and **F2** specimens in C_6D_6 after 5 days; (b) **F1** after addition of BF_3•OEt_2 for 5 minutes (left) and 20 minutes (right); (c) **F2** after addition of BF_3•OEt_2 for 5 minutes (left) and 20 minutes (right); (d) **F1** and **F2** specimens after 5 days.

The disassembly process was also followed by ^{11}B NMR spectroscopy (Figure S36), which showed that the BF_3•OEt_2 signal at 0.88 ppm gradually decreased, and a broad shoulder at 1.24 ppm appeared. When treating the molecular system **M1-^{Et}Py** with 2 equivalents of BF_3•OEt_2 an ^{11}B NMR signal at a similar shift of 0.4 ppm was observed, confirming the formation of pyridine-complexed BF_3. A new signal at 52.8 ppm was observed, assigned to tricoordinate boron (pristine **M1** at 52.2 ppm). However, the ^{11}B NMR signals of the uncomplexed tricoordinate borane moieties of **PS1** and **PS2** could not be observed because of the very low concentration and extreme broadening of the signals of the polymers.

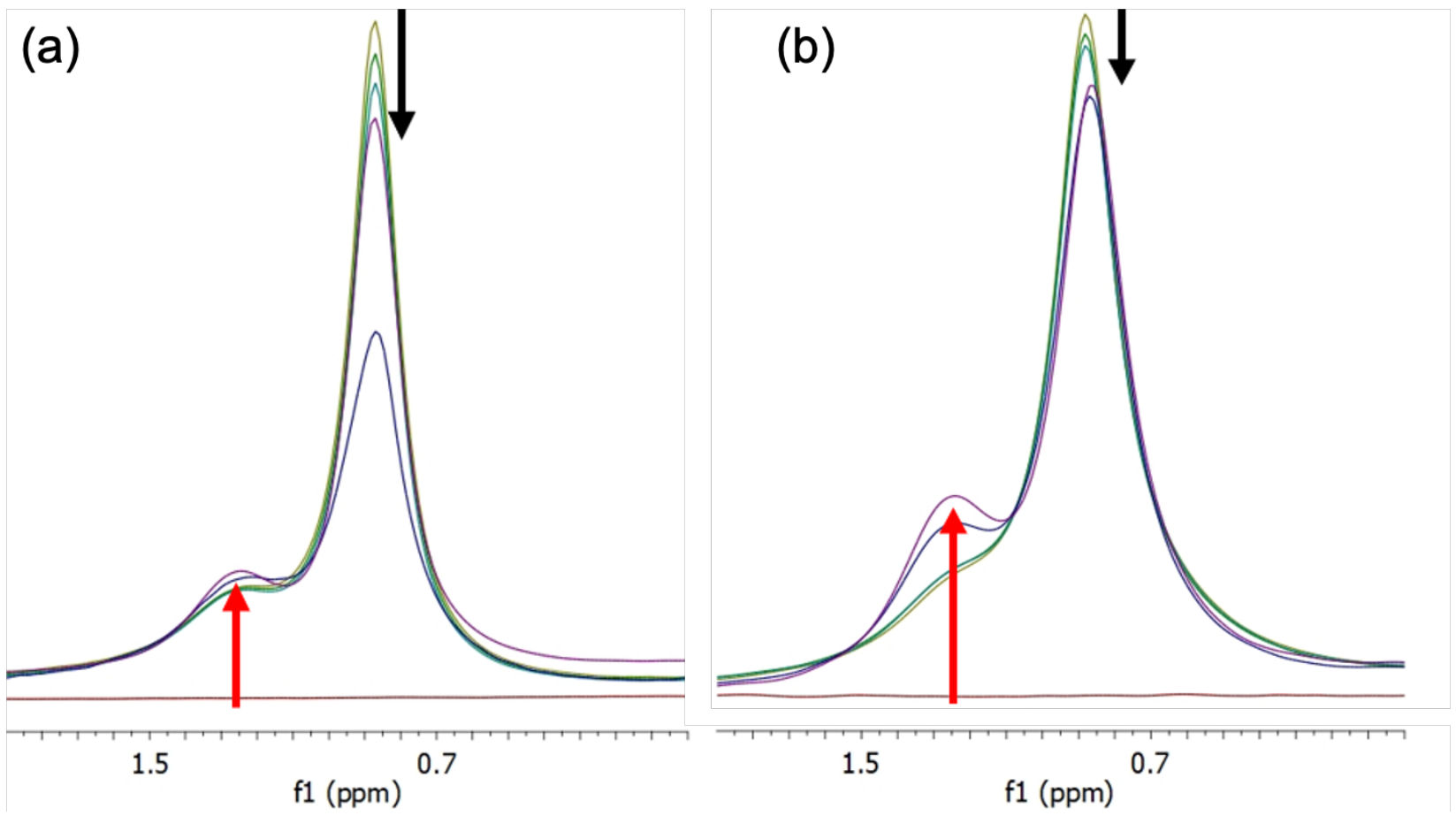

Figure S36. ^{11}B NMR spectra of (a) **F1** and (b) **F2** after treatment with 2 equivalents of BF_3•OEt_2 in C_6D_6, monitored over a period of 20 minutes (yellow trace) to 5 days (purple trace) of reaction time.

10. Spectral Data of Isolated Compounds

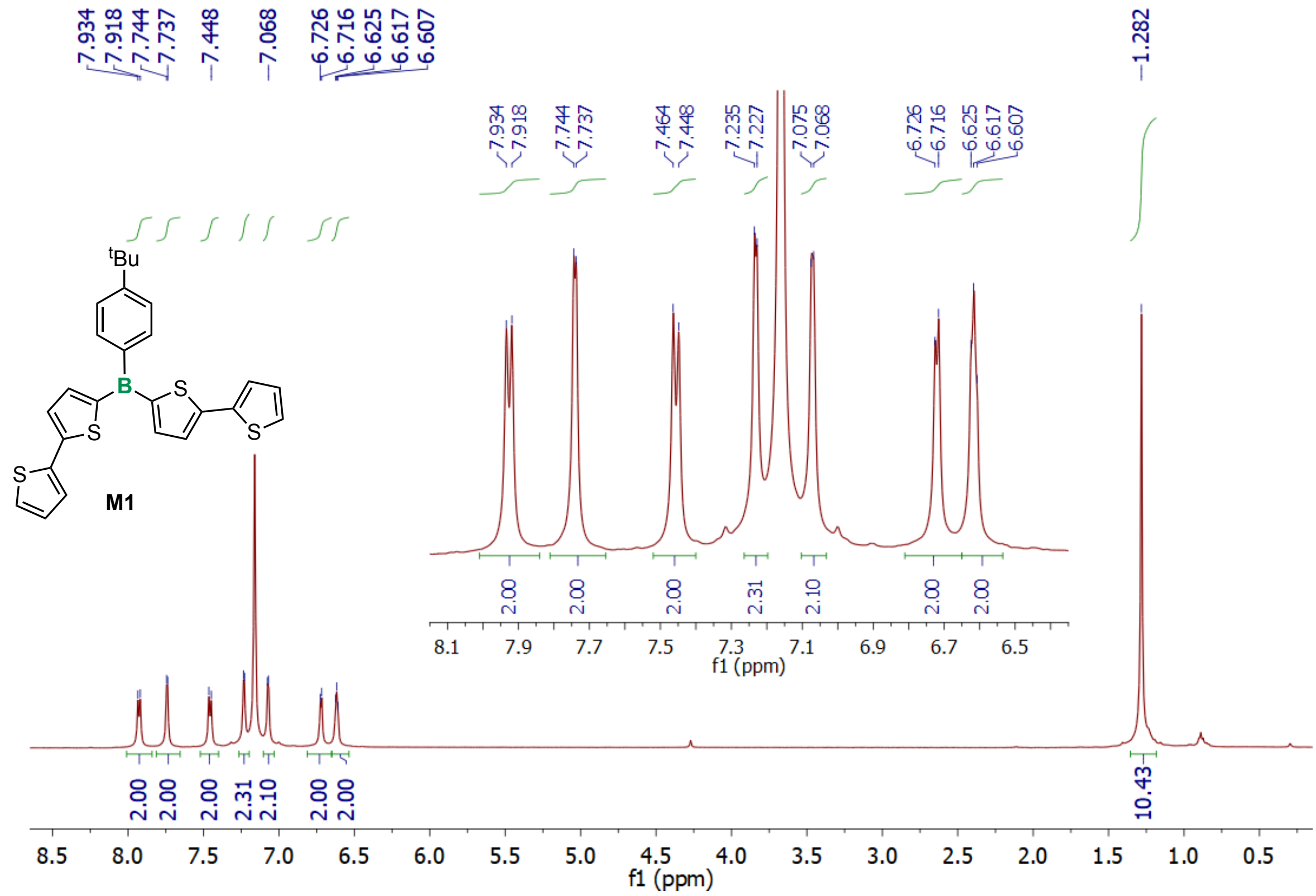

Figure S37. ^{1}H NMR spectrum of **M1** in C_6D_6 at 25 °C.

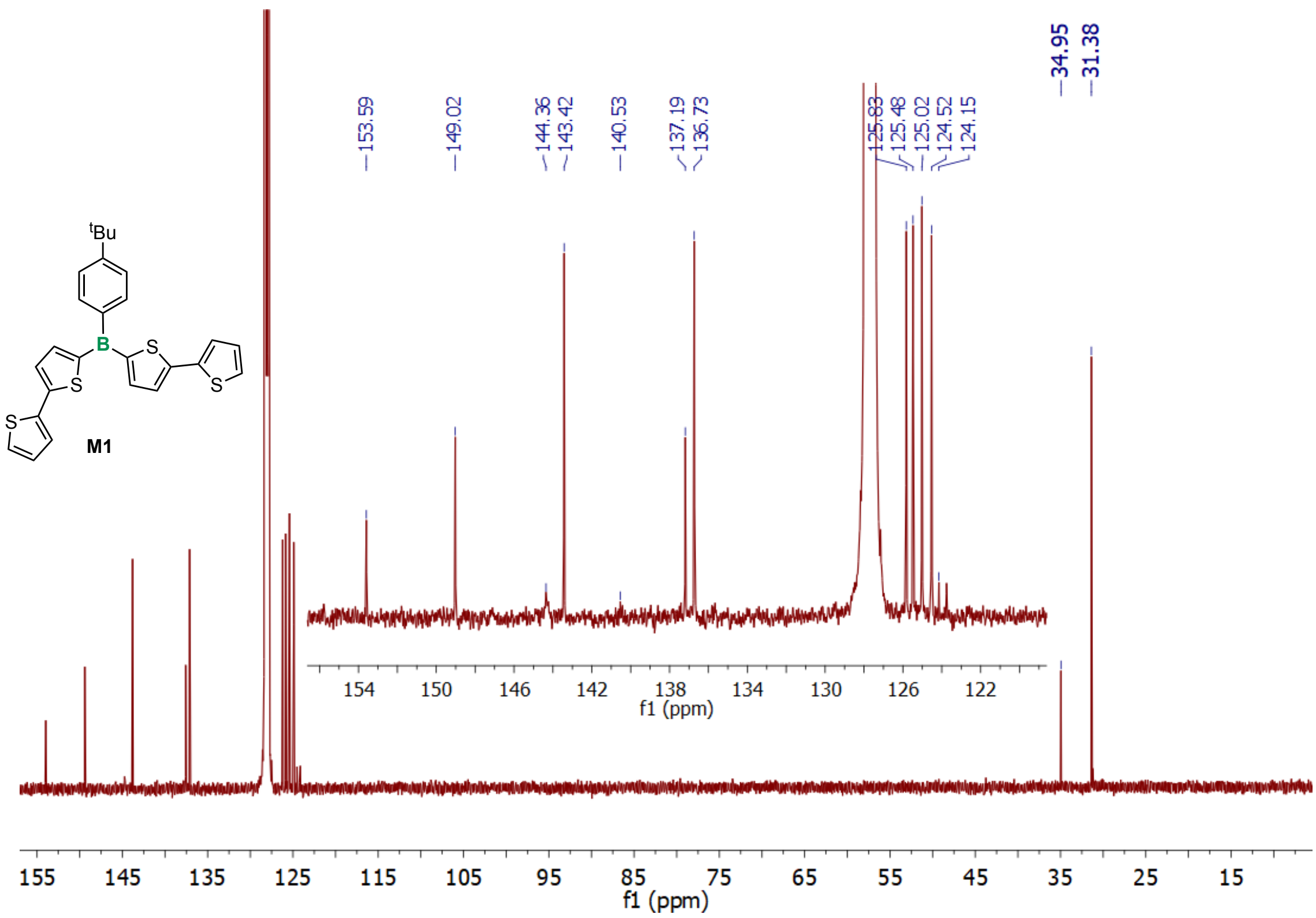

Figure S38. ^{13}C NMR spectrum of **M1** in C_6D_6 at 25 °C.

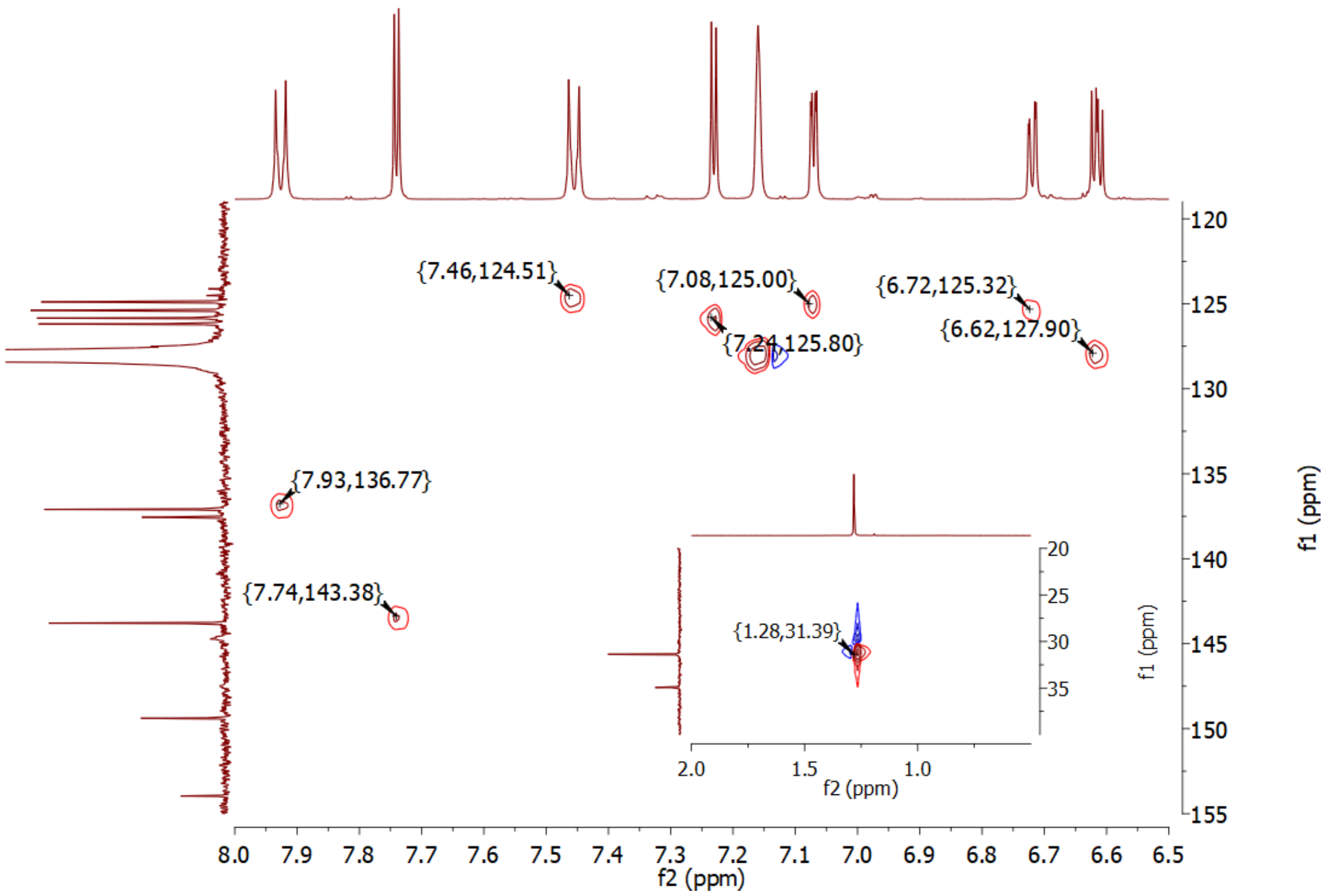

Figure S39. HSQC NMR spectrum of **M1** in C_6D_6 at 25 °C.

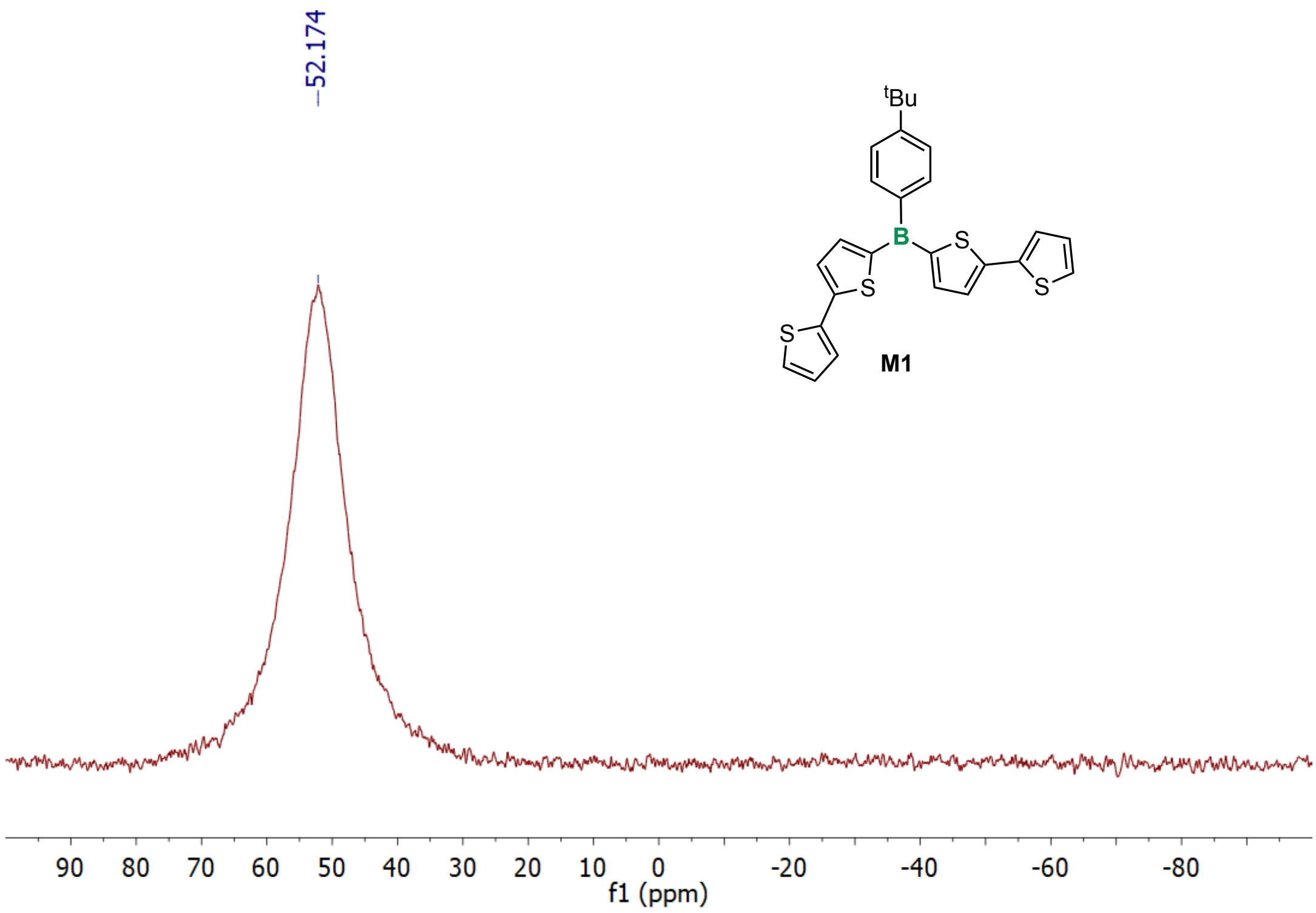

Figure S40. ^{11}B NMR spectrum of **M1** in C_6D_6 at 25 °C.

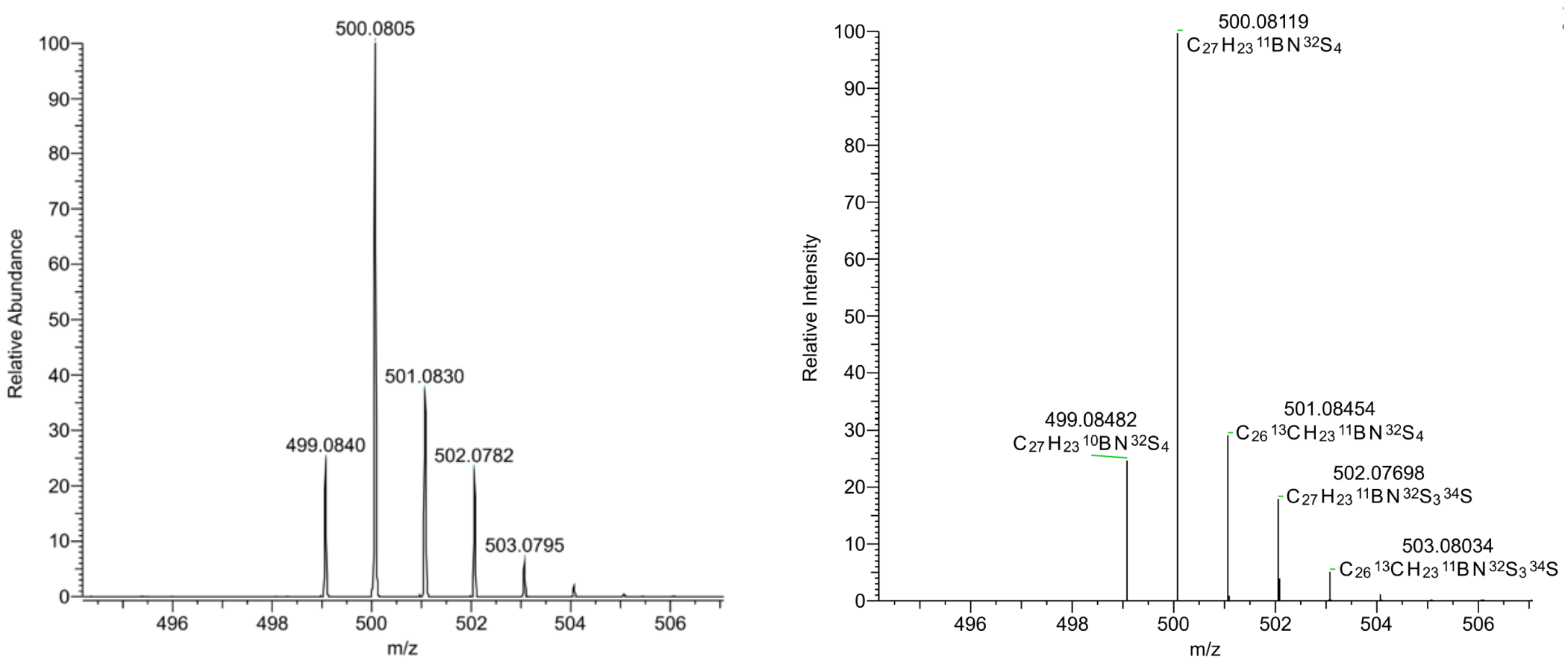

Figure S41. HRMS data for **M1** (CN^- complex, negative mode in toluene/MeCN).

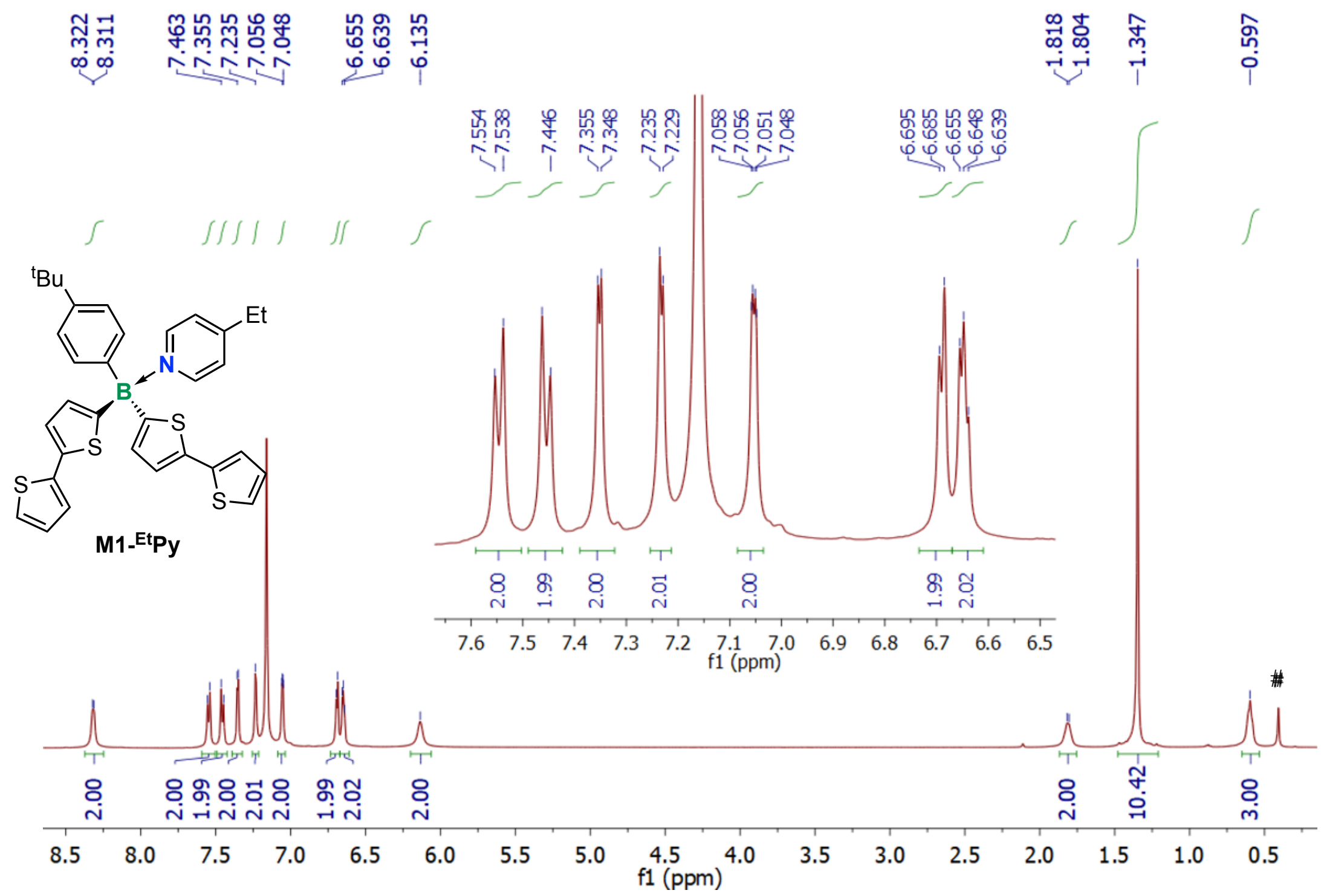

Figure S42. ^{1}H NMR spectrum of **M1-^{Et}Py** in C_6D_6 at 25 °C; # silicone grease.

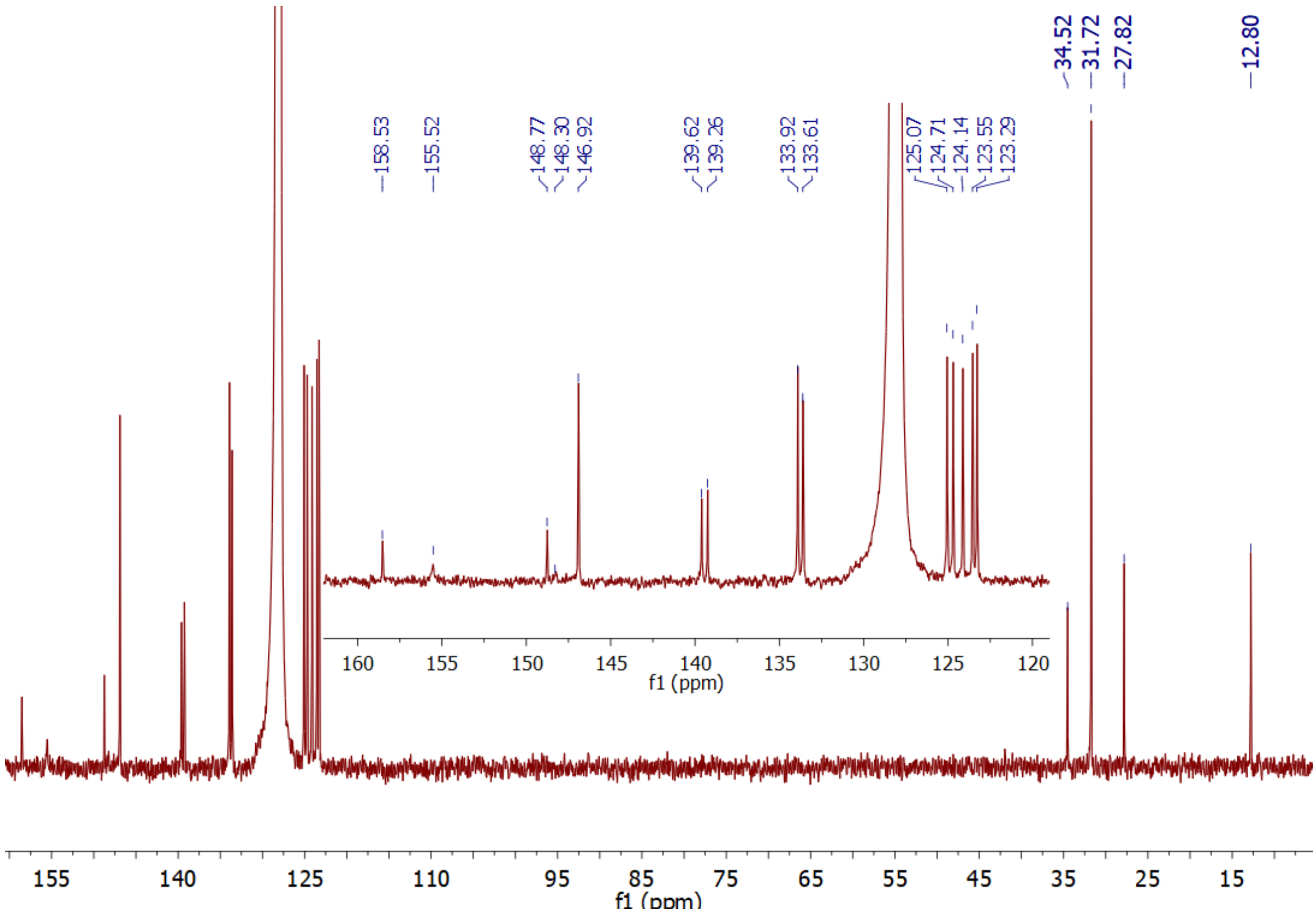

Figure S43. ^{13}C NMR spectrum of **M1-^{Et}Py** in C_6D_6 at 25 °C.

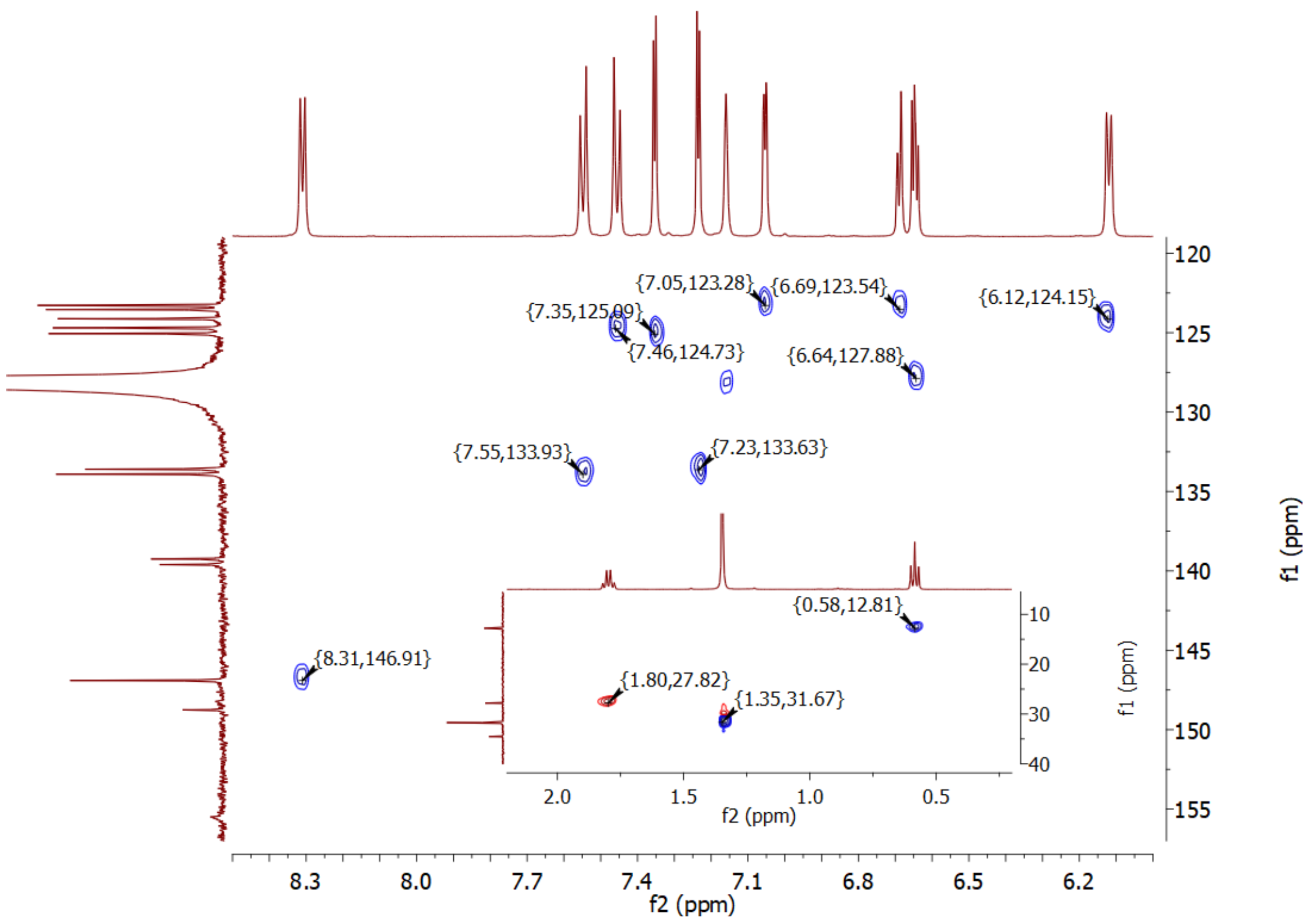

Figure S44. HSQC NMR spectrum of **M1-^{Et}Py** in C_6D_6 at 25 °C.

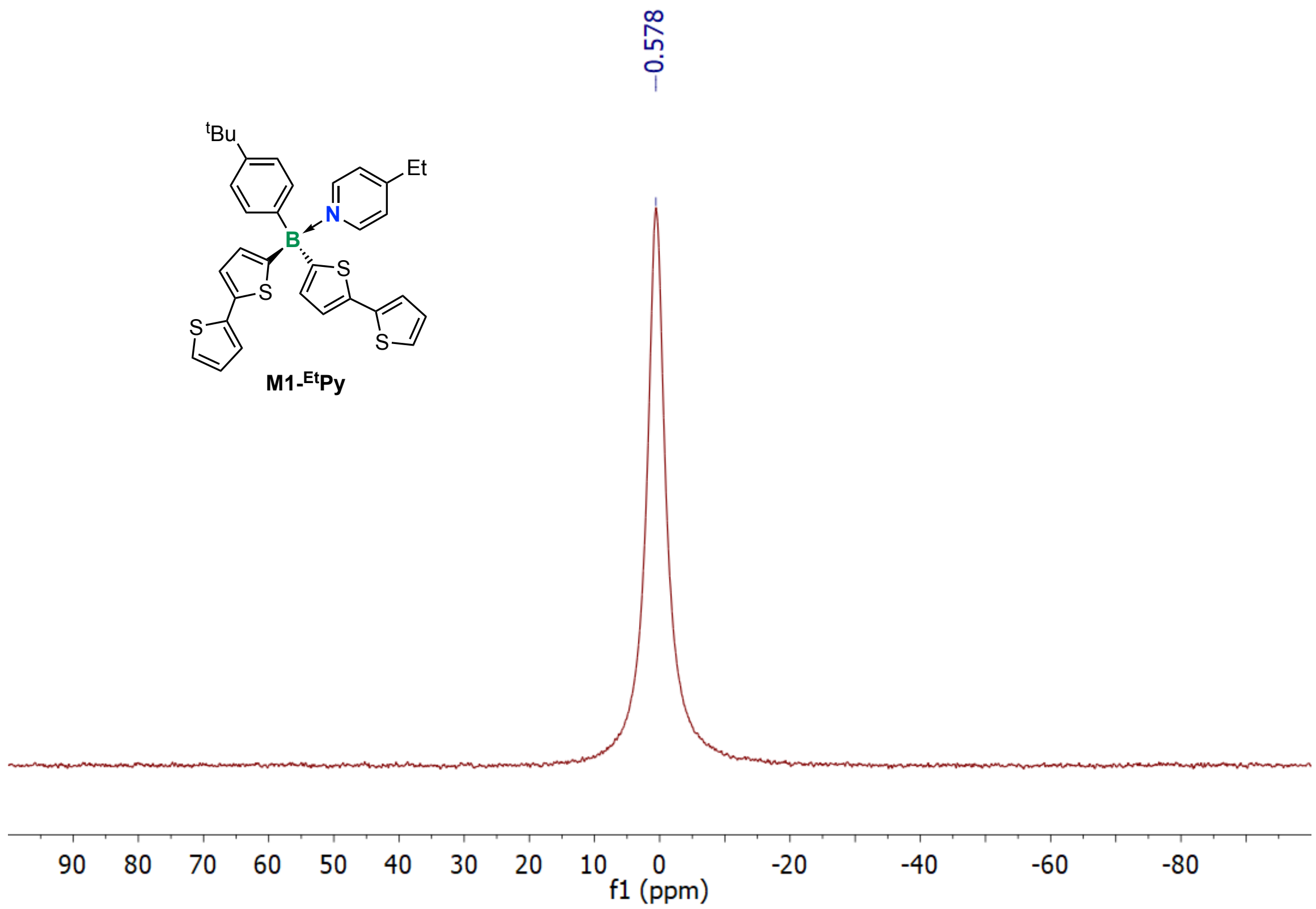

Figure S45. ^{11}B NMR spectrum of **M1-^{Et}Py** in C_6D_6 at 25 °C.

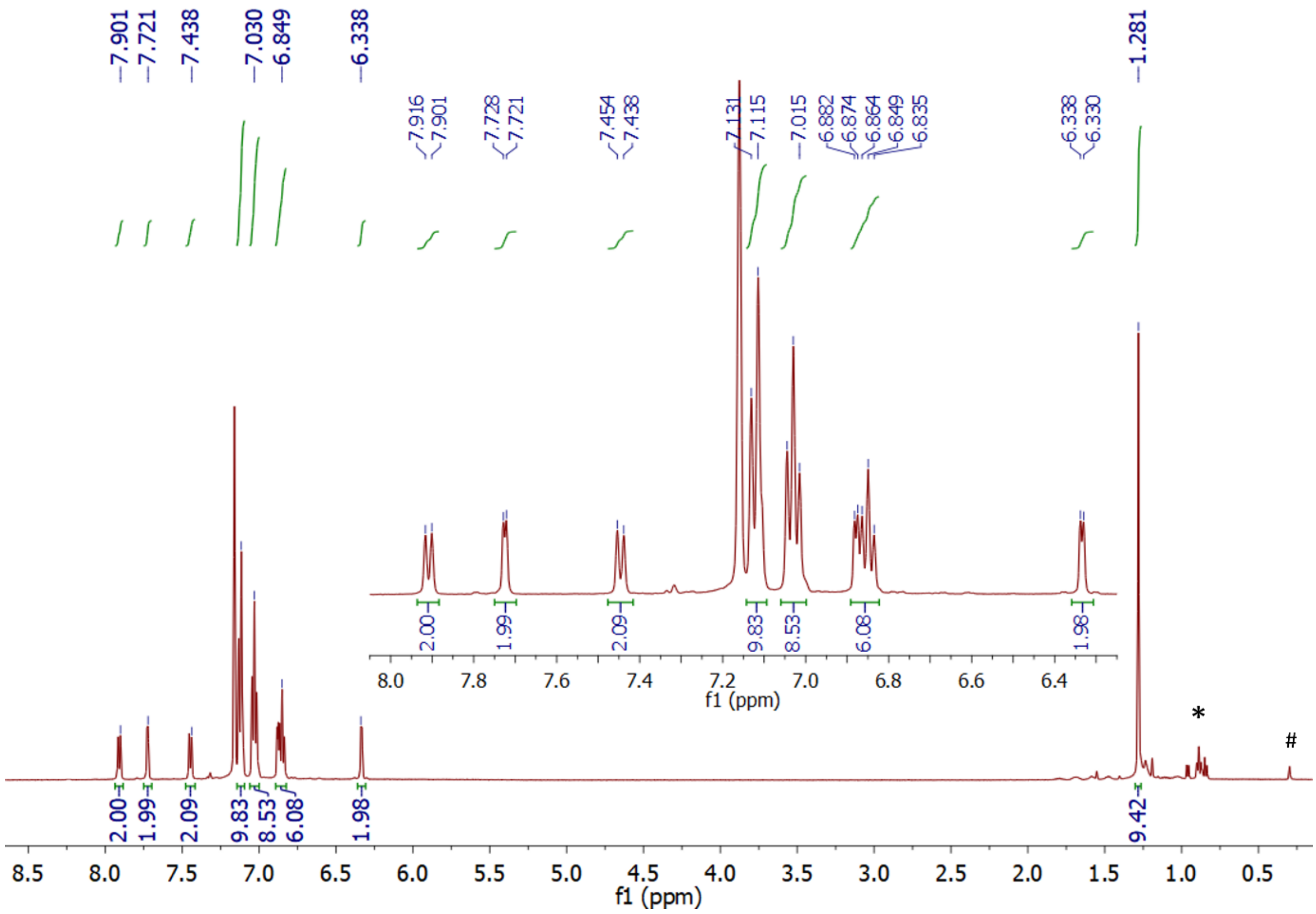

Figure S46. ^{1}H NMR spectrum of **M2** in C_6D_6 at 25 °C; * hexanes, # silicone grease.

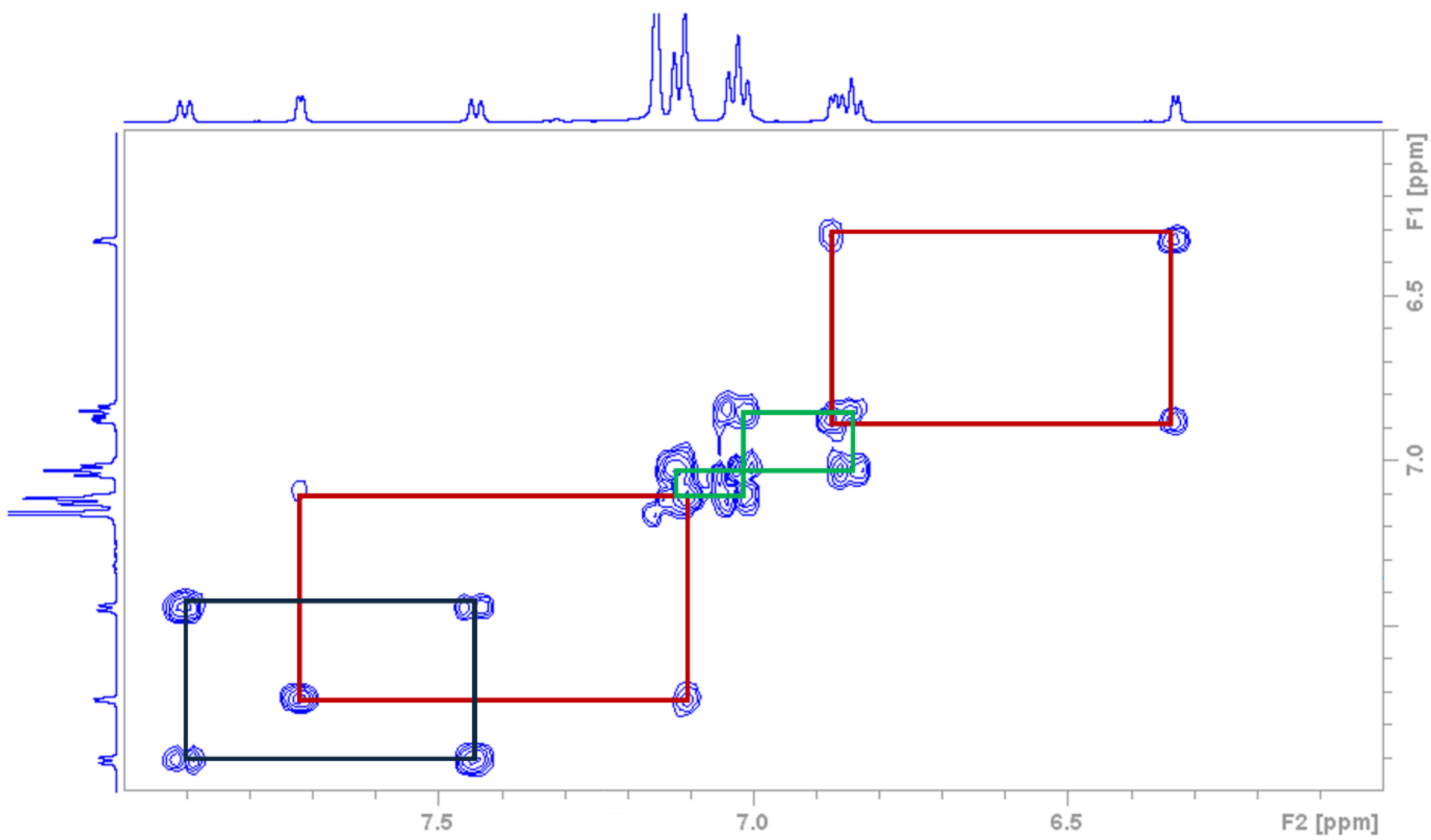

Figure S47. H,H-COSY NMR spectrum of **M2** in C_6D_6 at 25 °C.

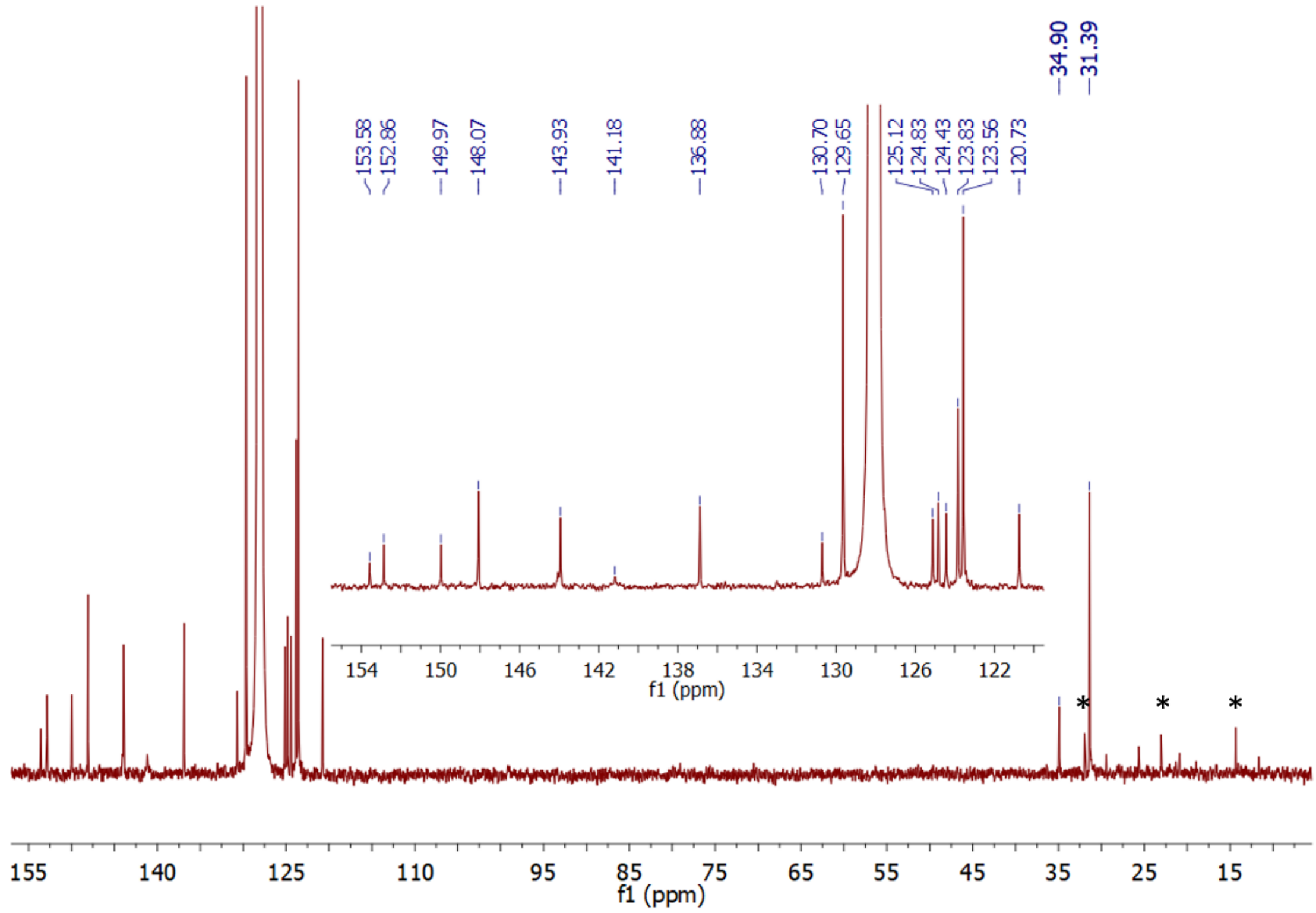

Figure S48. ^{13}C NMR spectrum of **M2** in C_6D_6 at 25 °C; * hexanes.

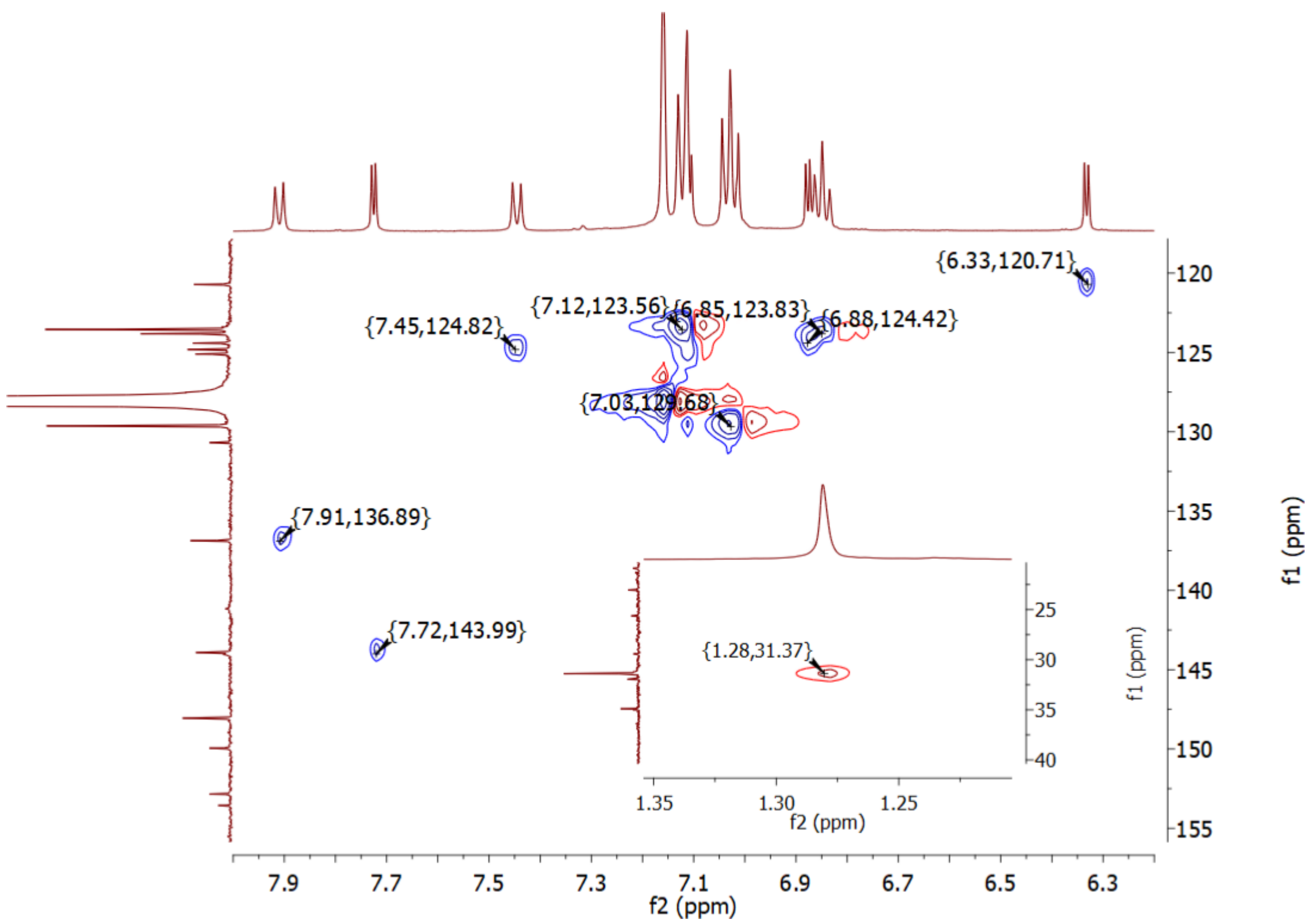

Figure S49. HSQC NMR spectrum of **M2** in C_6D_6 at 25 °C.

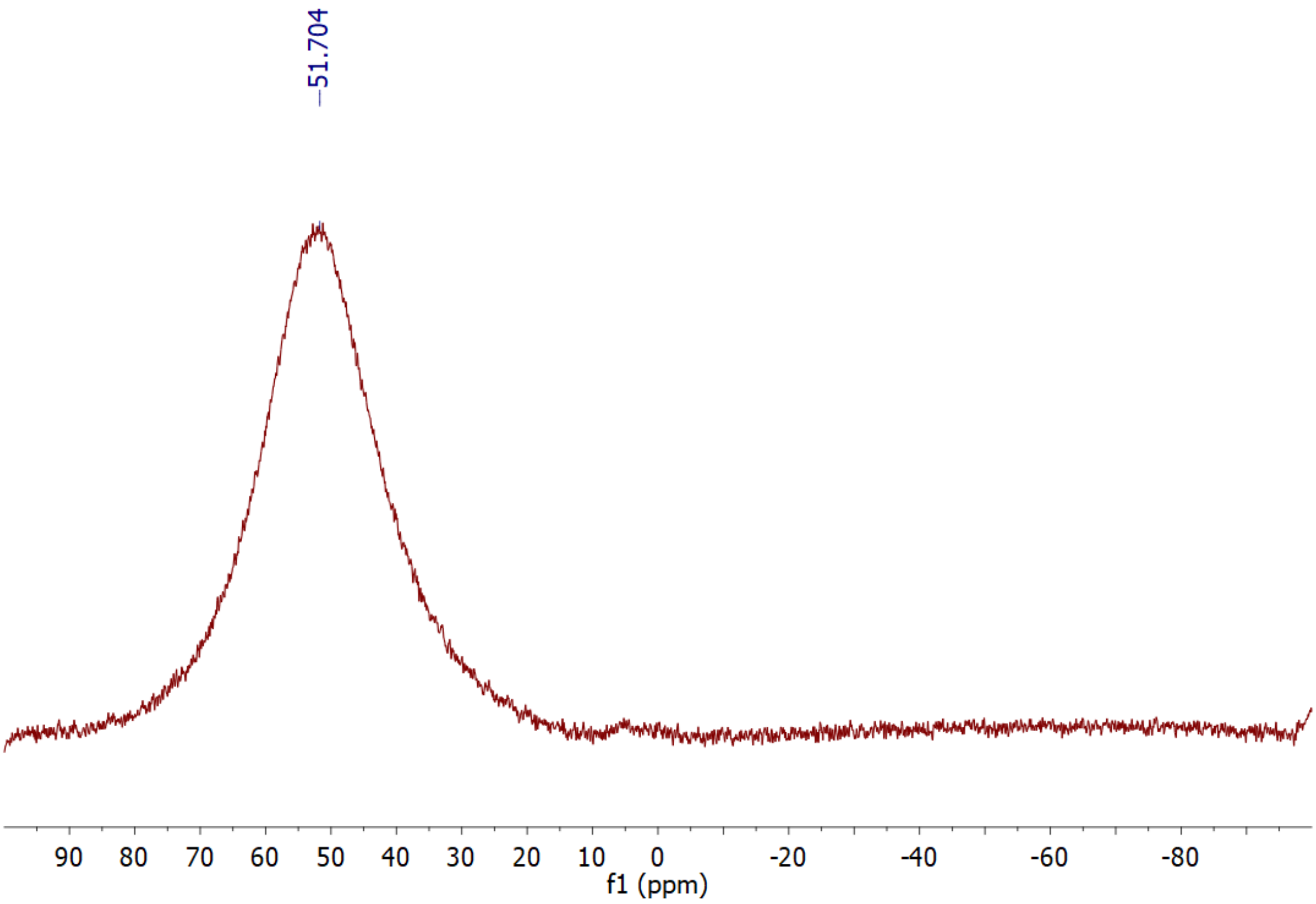

Figure S50. ^{11}B NMR spectrum of **M2** in C_6D_6 at 25 °C.

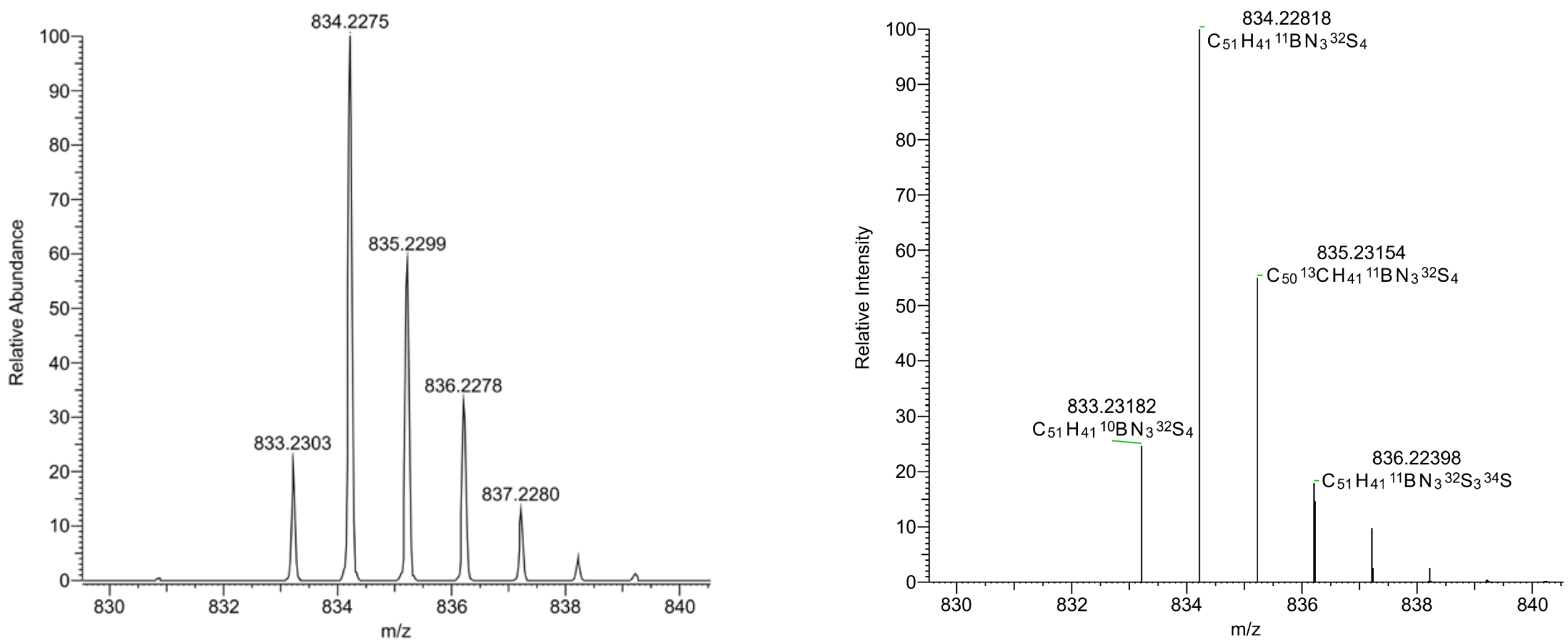

Figure S51. HRMS data for **M2** (CN^- complex, negative mode in toluene/MeCN).

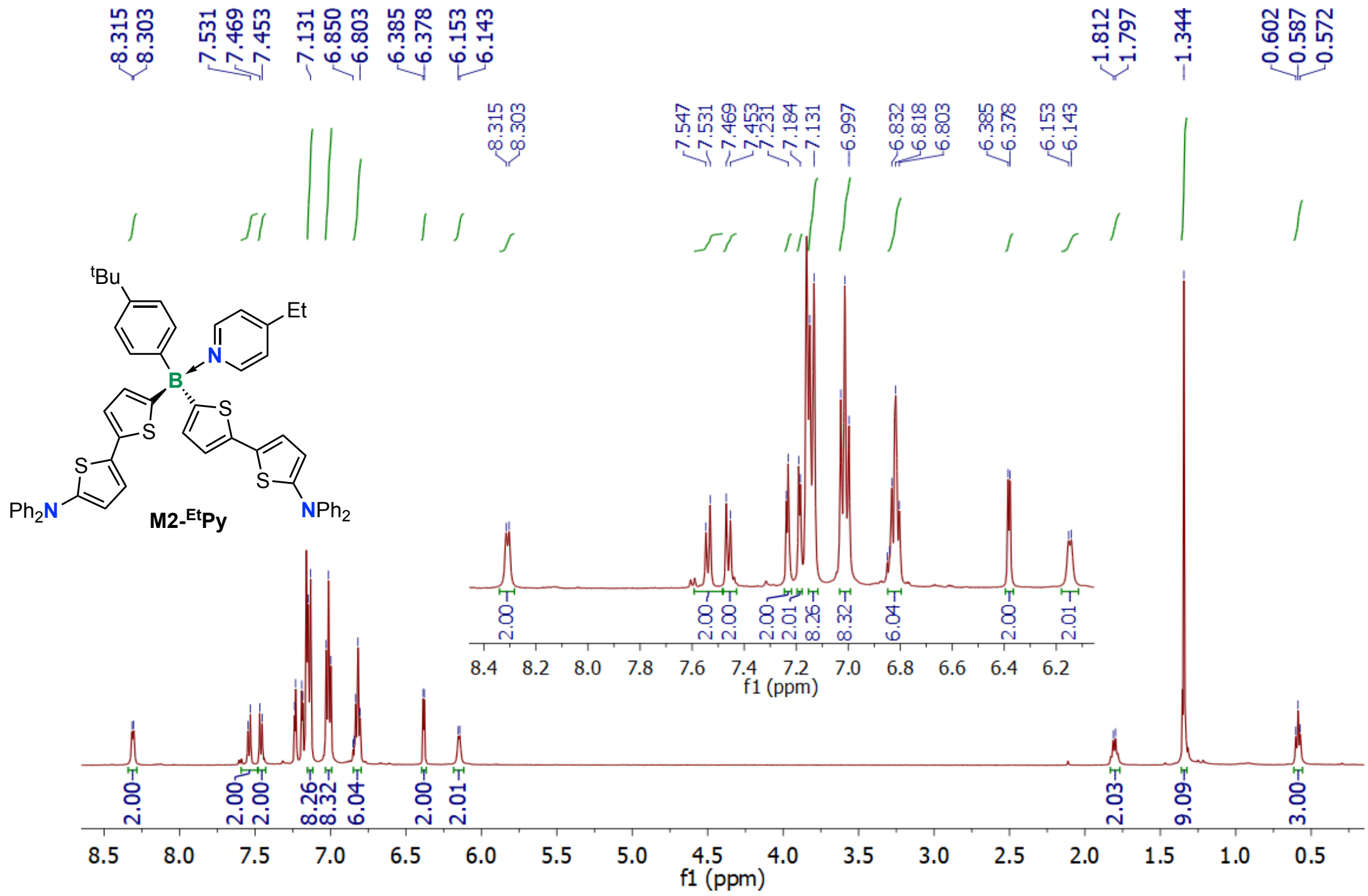

Figure S52. ^{1}H NMR spectrum of **M2-^{Et}Py** in C_6D_6 at 25 °C.

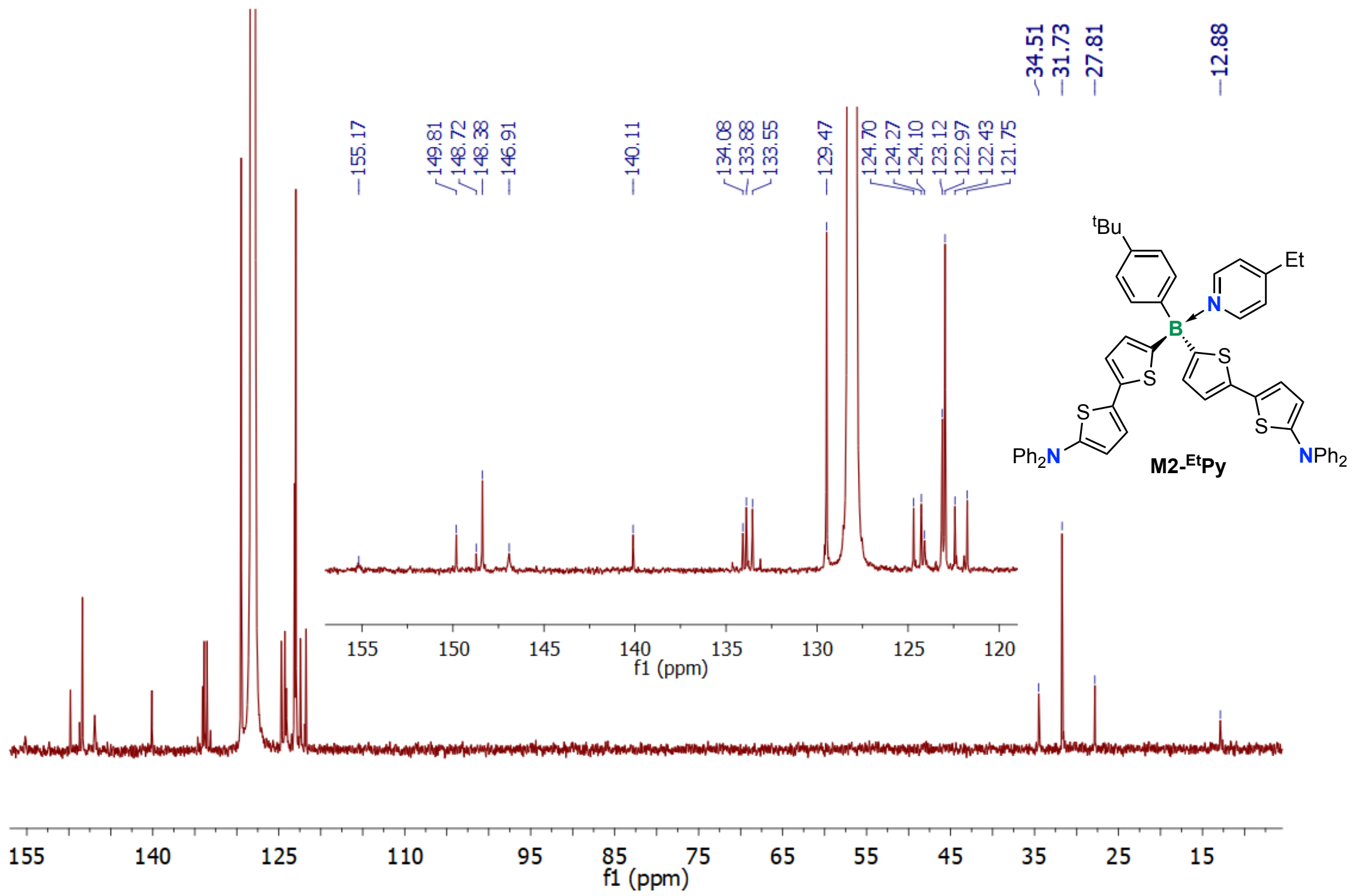

Figure S53. ^{13}C NMR spectrum of **M2-^{Et}Py** in C_6D_6 at 25 °C.

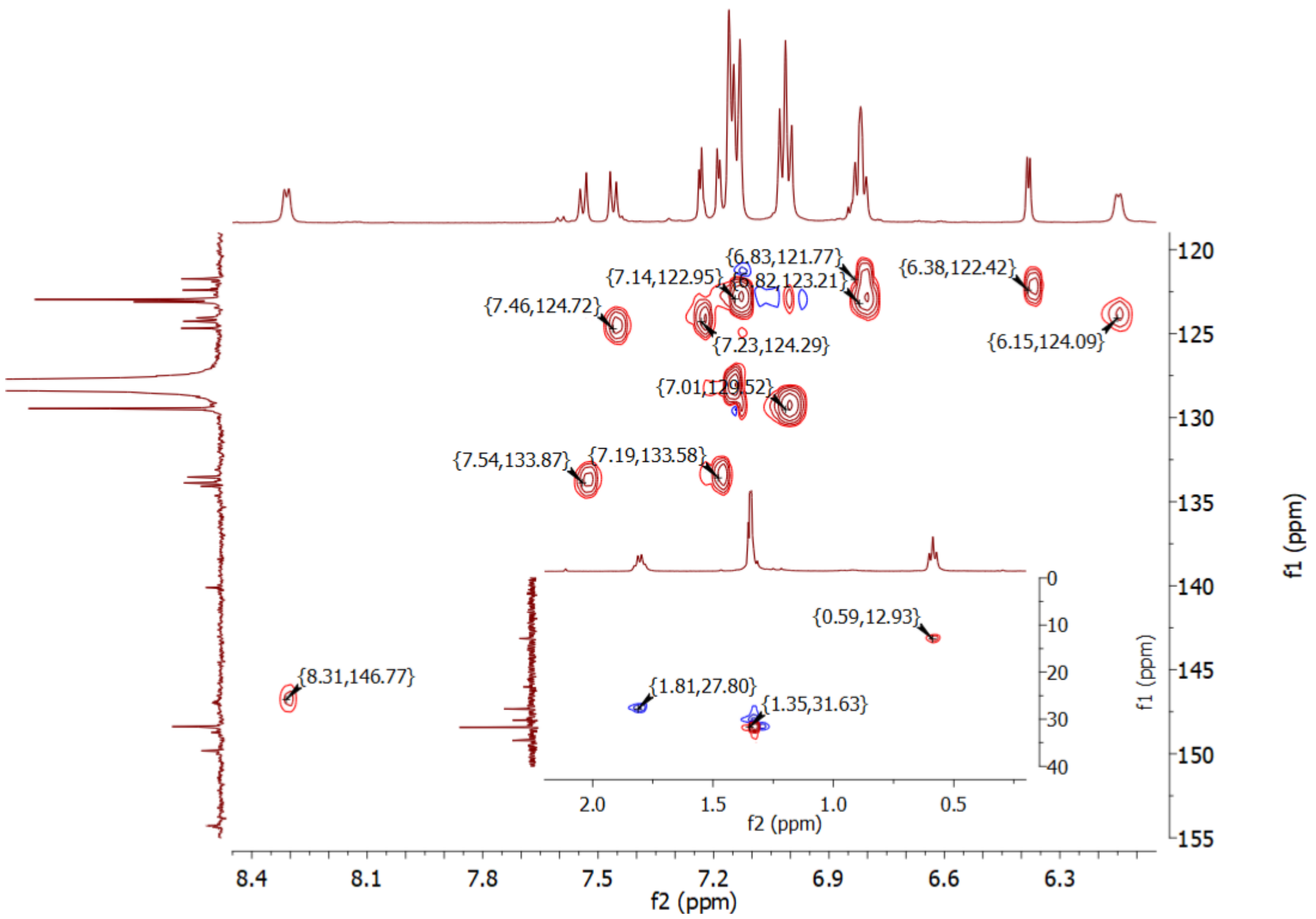

Figure S54. HSQC NMR spectrum of **M2-^{Et}Py** in C_6D_6 at 25 °C.

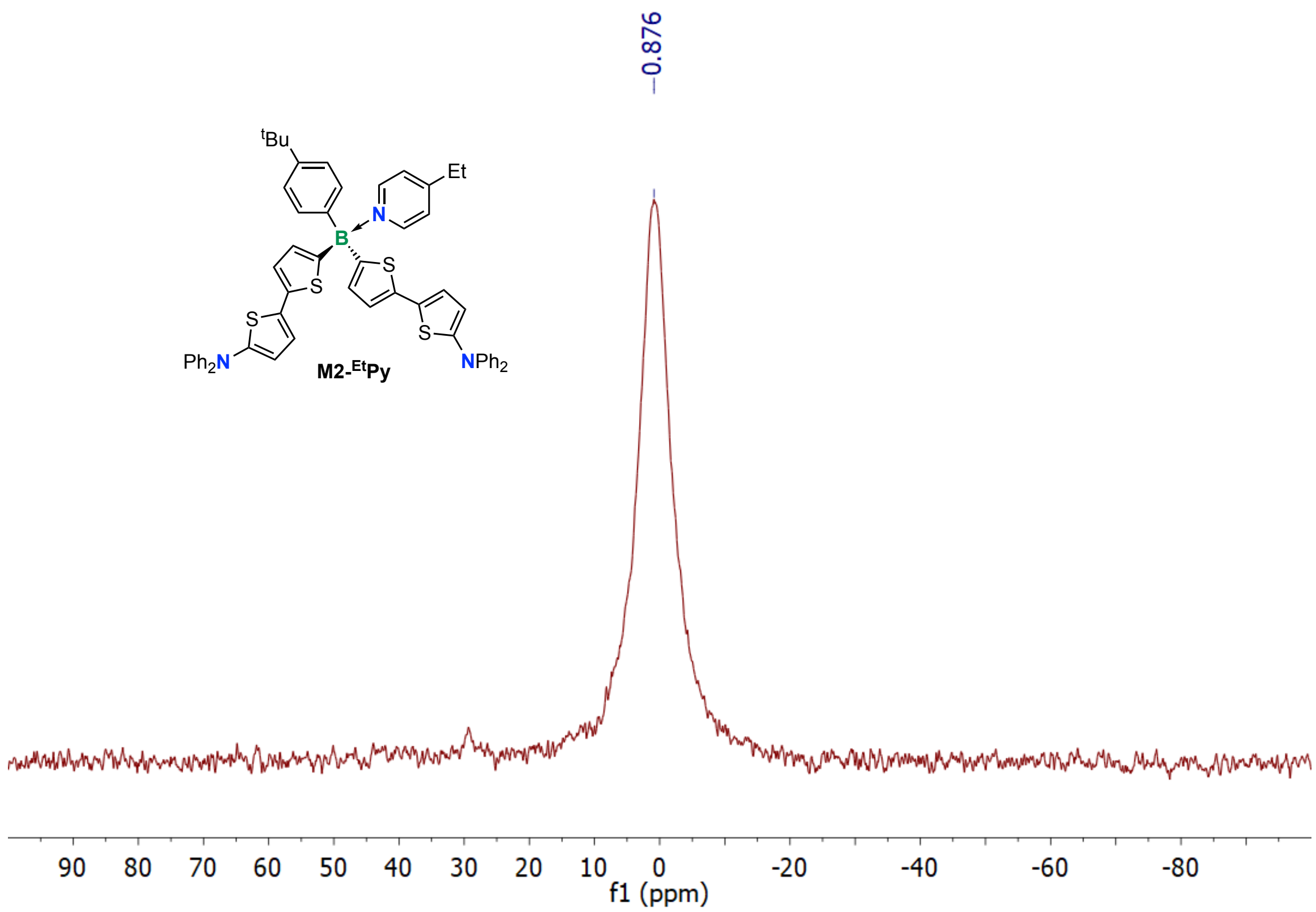

Figure S55. ^{11}B NMR spectrum of **M2-^{Et}Py** in C_6D_6 at 25 °C.

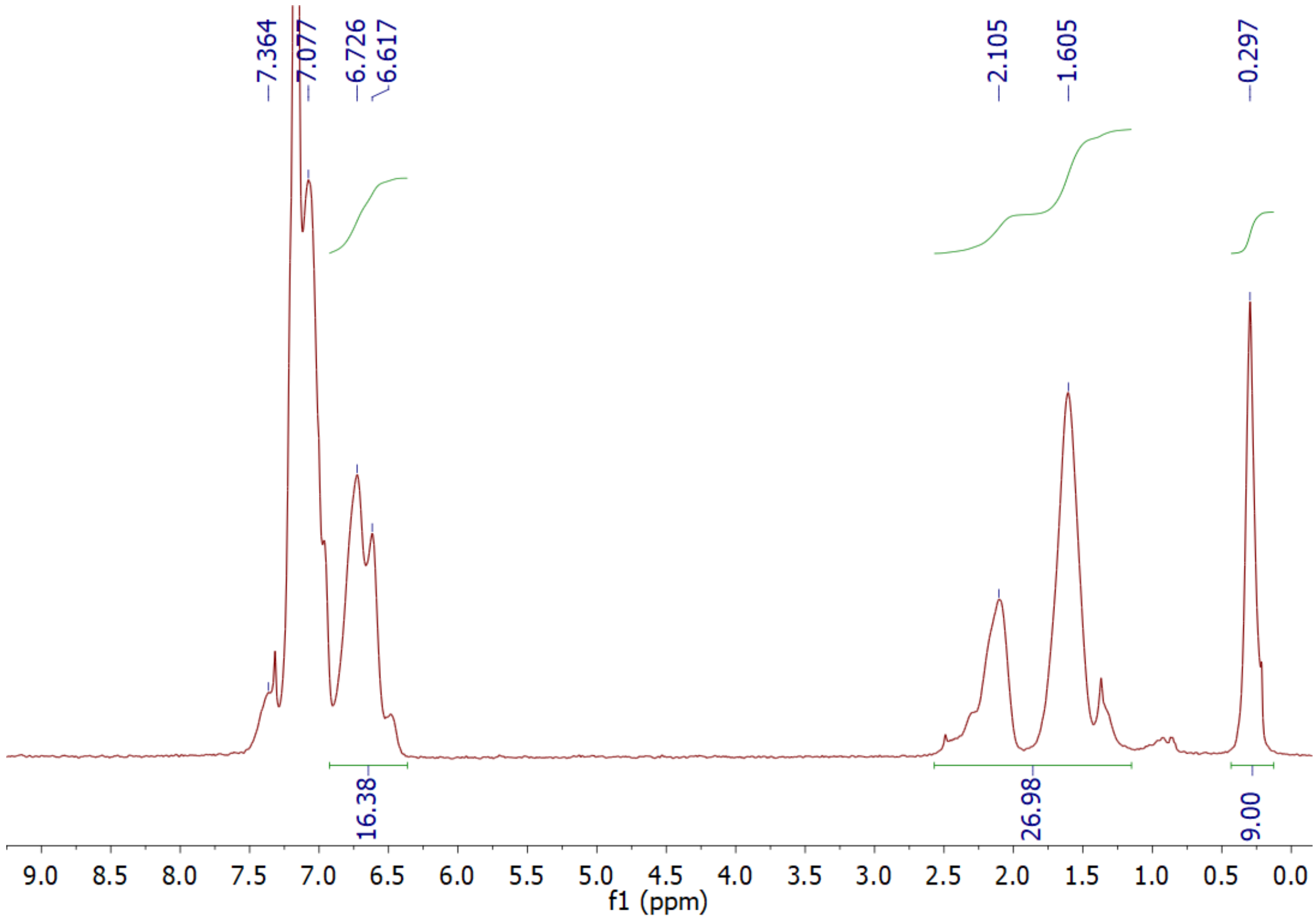

Figure S56. ^{1}H NMR spectrum of P(St-co-StSiMe$_3$) in C_6D_6 at 25 °C.

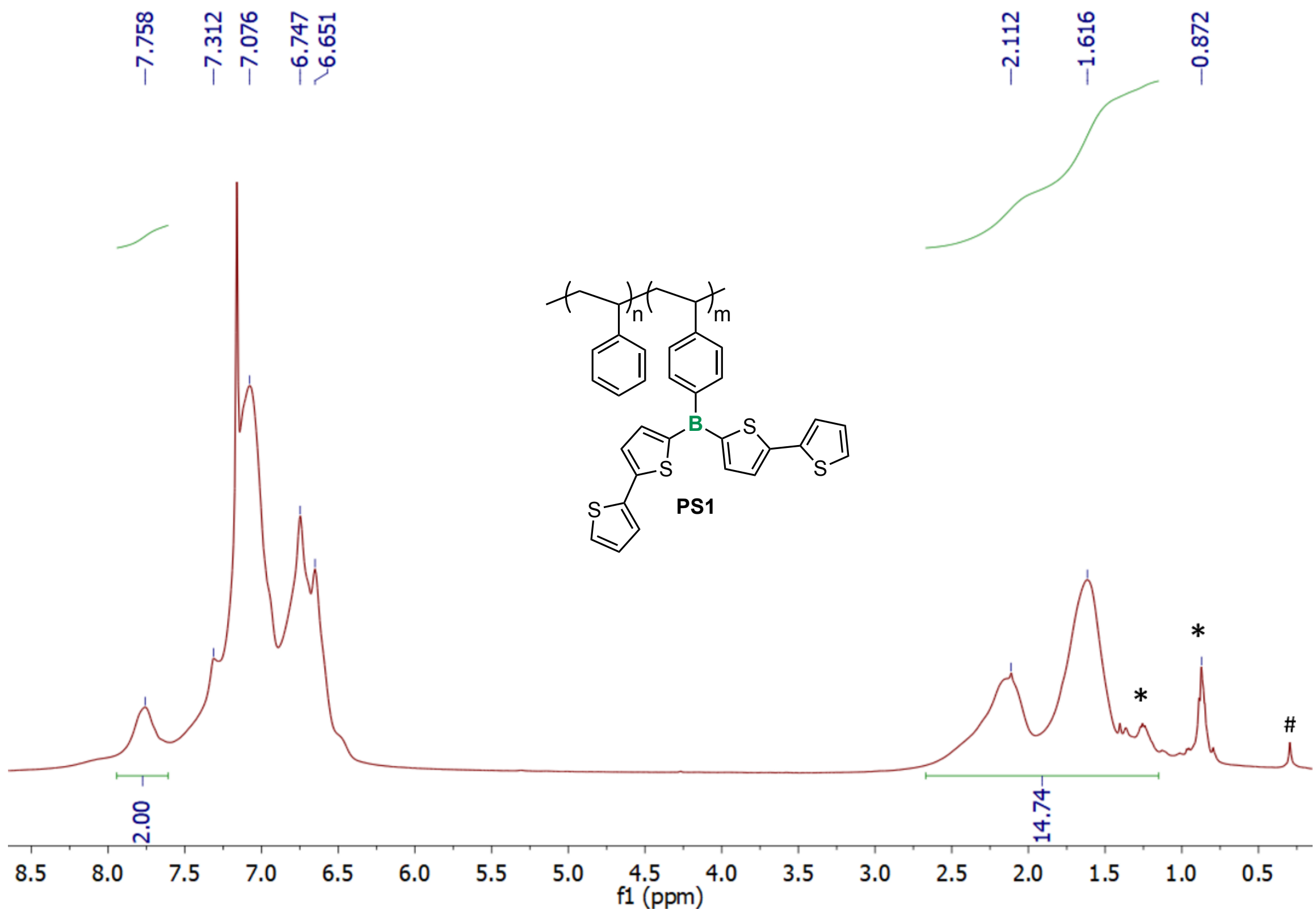

Figure S57. ^{1}H NMR spectrum of **PS1** in C_6D_6 at 25 °C; * residual hexanes, # silicone grease.

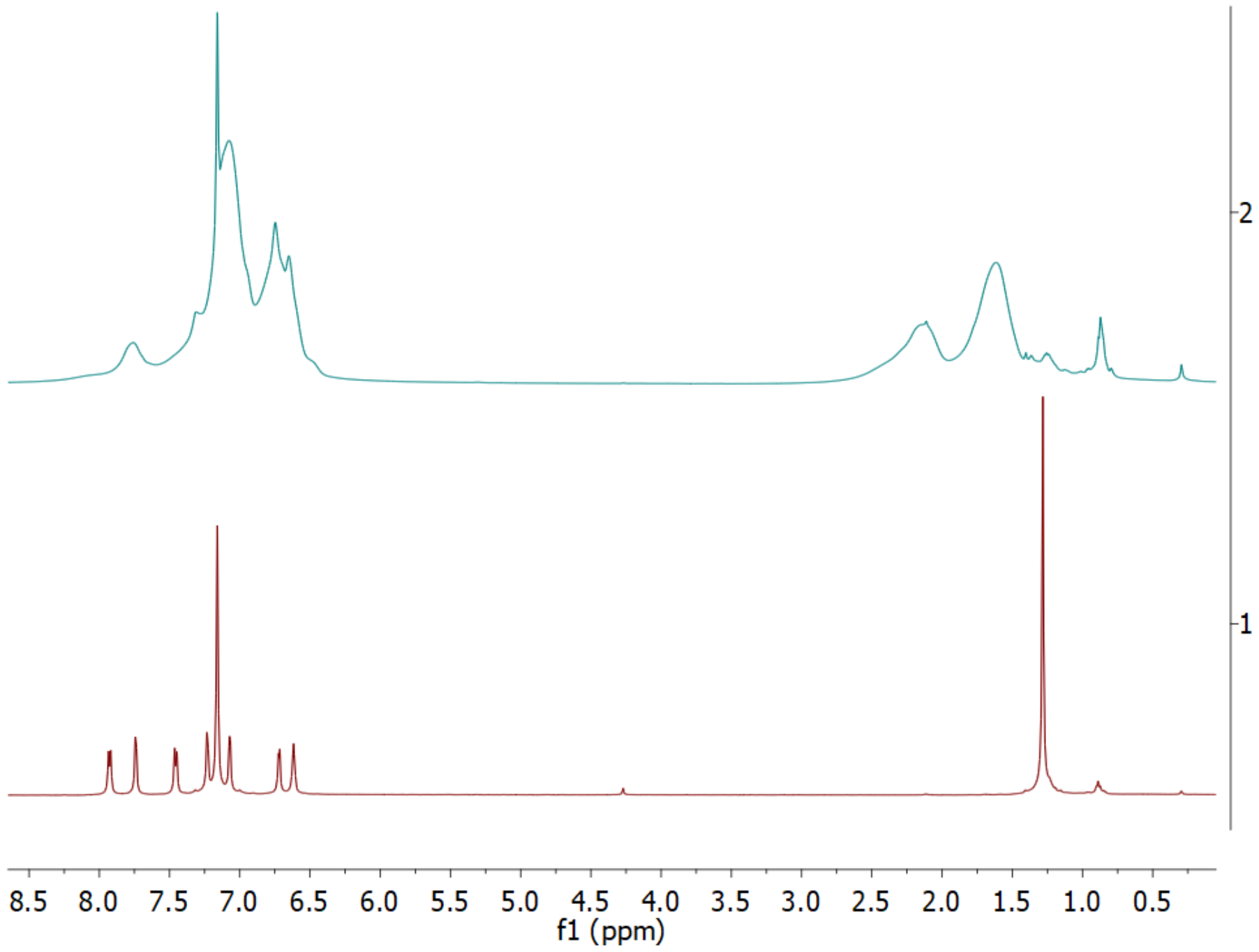

Figure S58. Stacked ^{1}H NMR spectra of **PS1** and **M1** in C_6D_6 at 25 °C. Trace 1 for **M1**, Trace 2 for **PS1**.

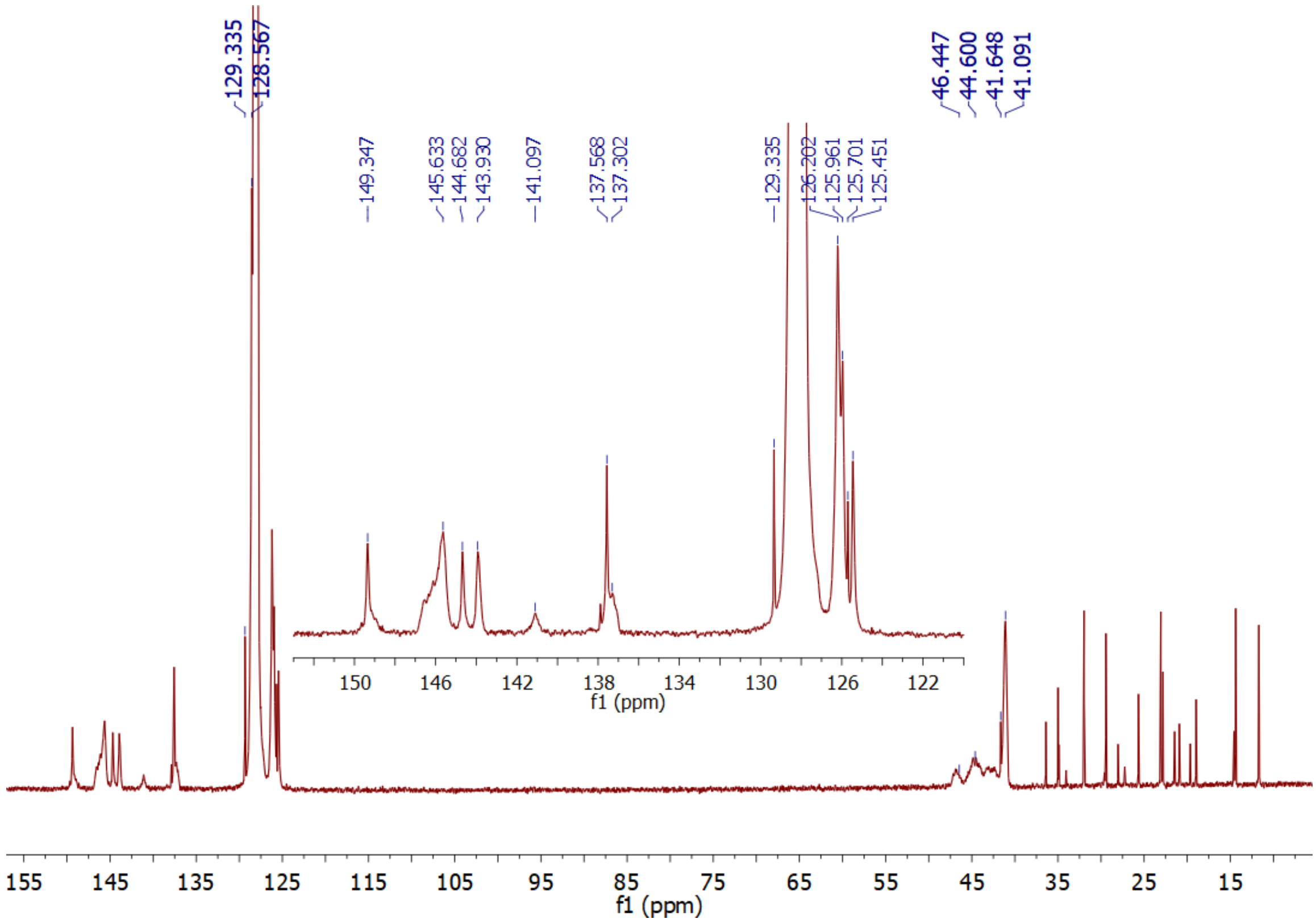

Figure S59. ^{13}C NMR spectrum of **PS1** in C_6D_6 at 25 °C (sharp peaks from 35-10 ppm due to residual hexanes).

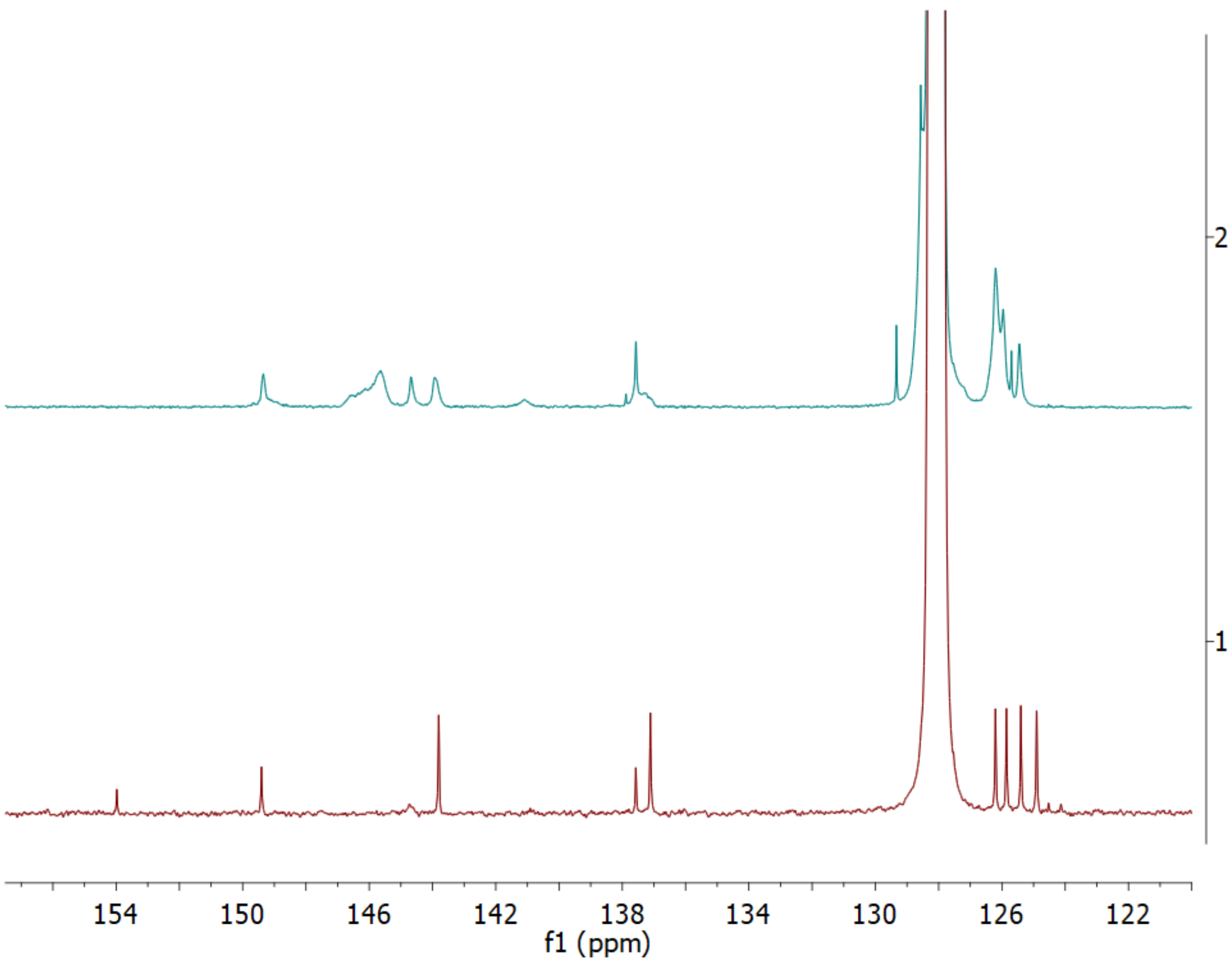

Figure S60. Stacked ^{13}C NMR spectra of **PS1** and **M1** in C_6D_6 at 25 °C. Trace 1 for **M1**, Trace 2 for **PS1**.

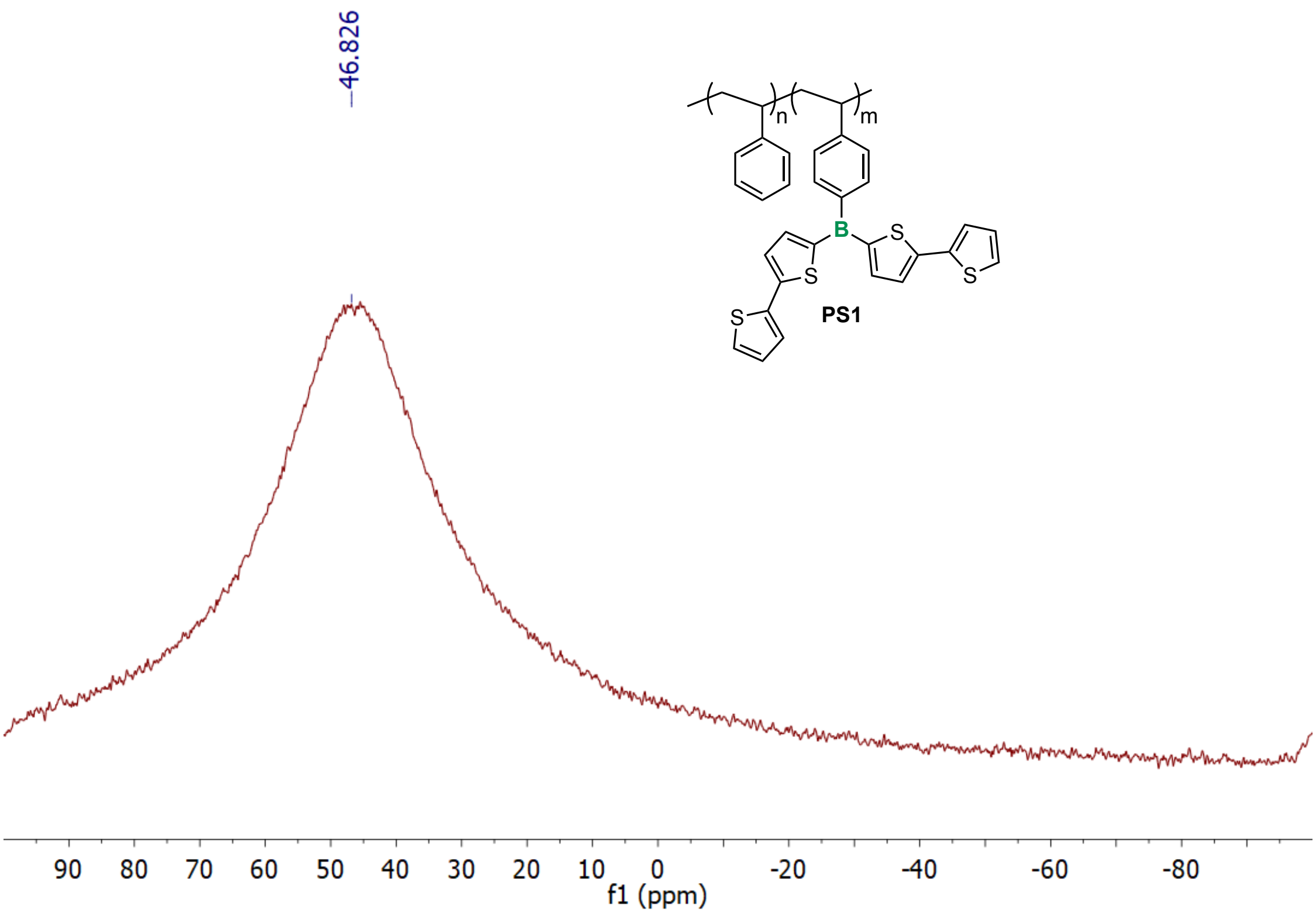

Figure S61. ^{11}B NMR spectrum of **PS1** in C_6D_6 at 25 °C.

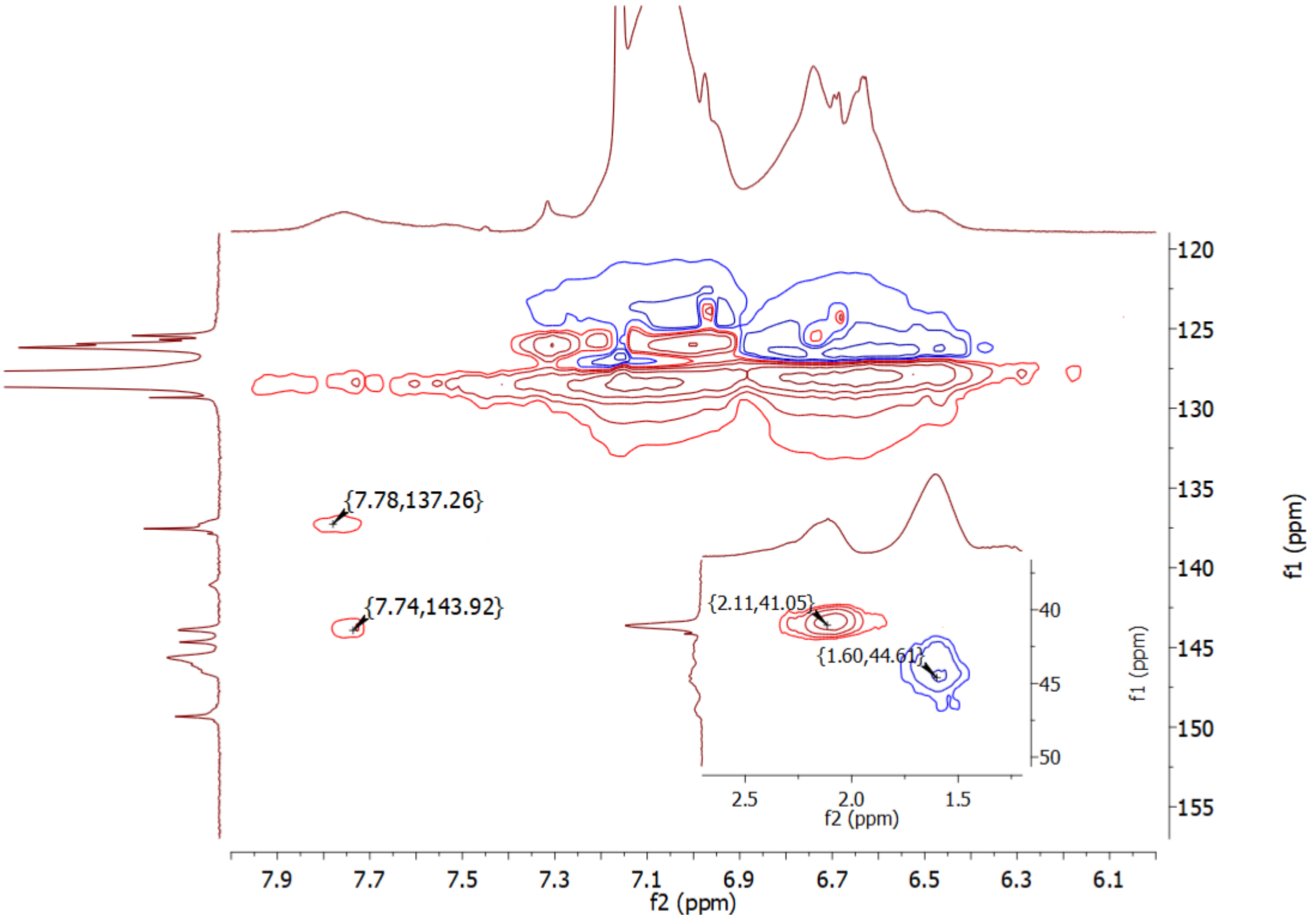

Figure S62. HSQC NMR spectrum of **PS1** in C_6D_6 at 25 °C.

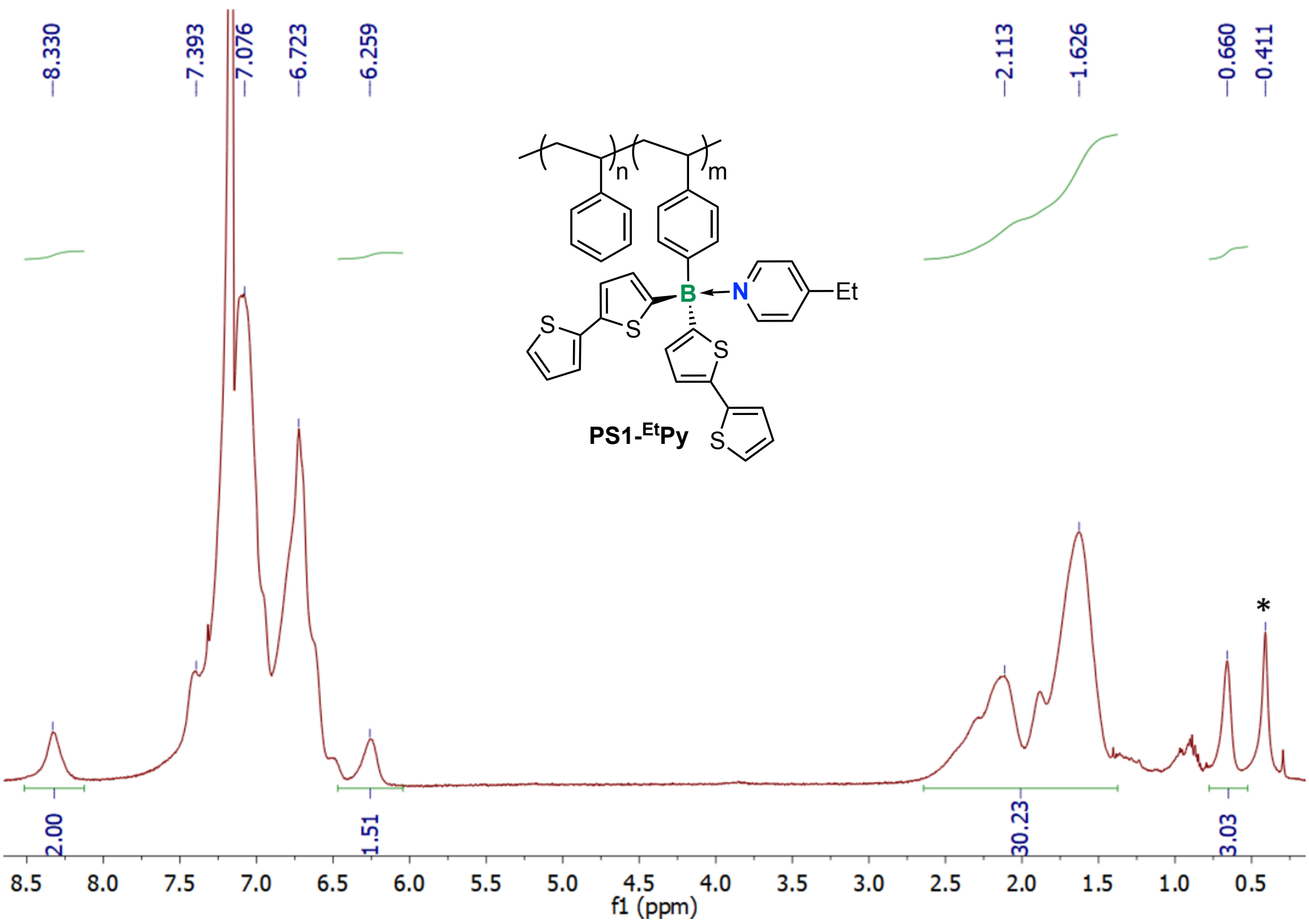

Figure S63. ^{1}H NMR spectrum of **PS1-EtPy** in C_6D_6 at 25 °C; * H_2O in deuterated solvent.

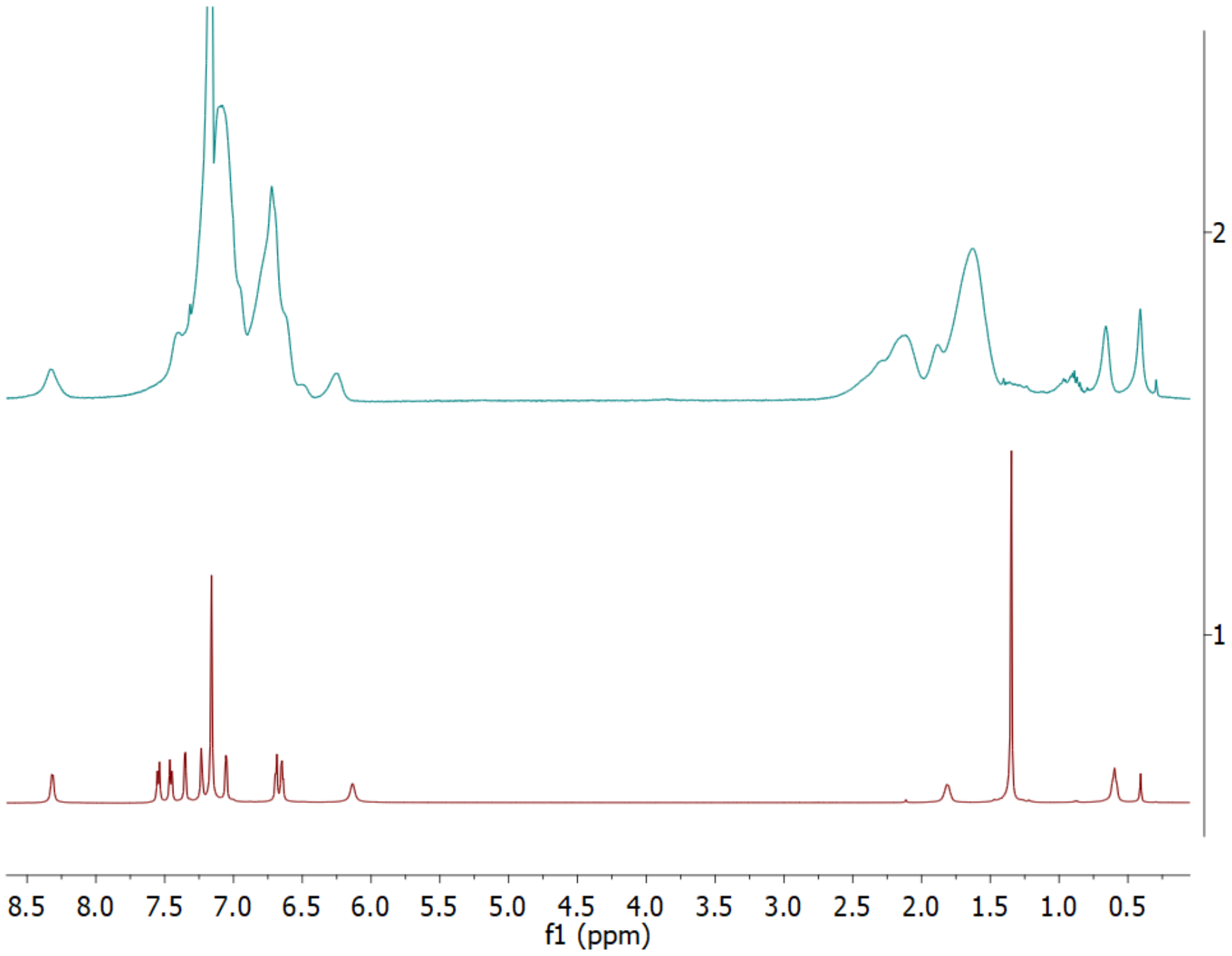

Figure S64. Stacked ^{1}H NMR spectra of **PS1-^{Et}Py** and **M1-^{Et}Py** in C_6D_6 at 25 °C. Trace 1 for **M1-^{Et}Py**, Trace 2 for **PS1-^{Et}Py**.

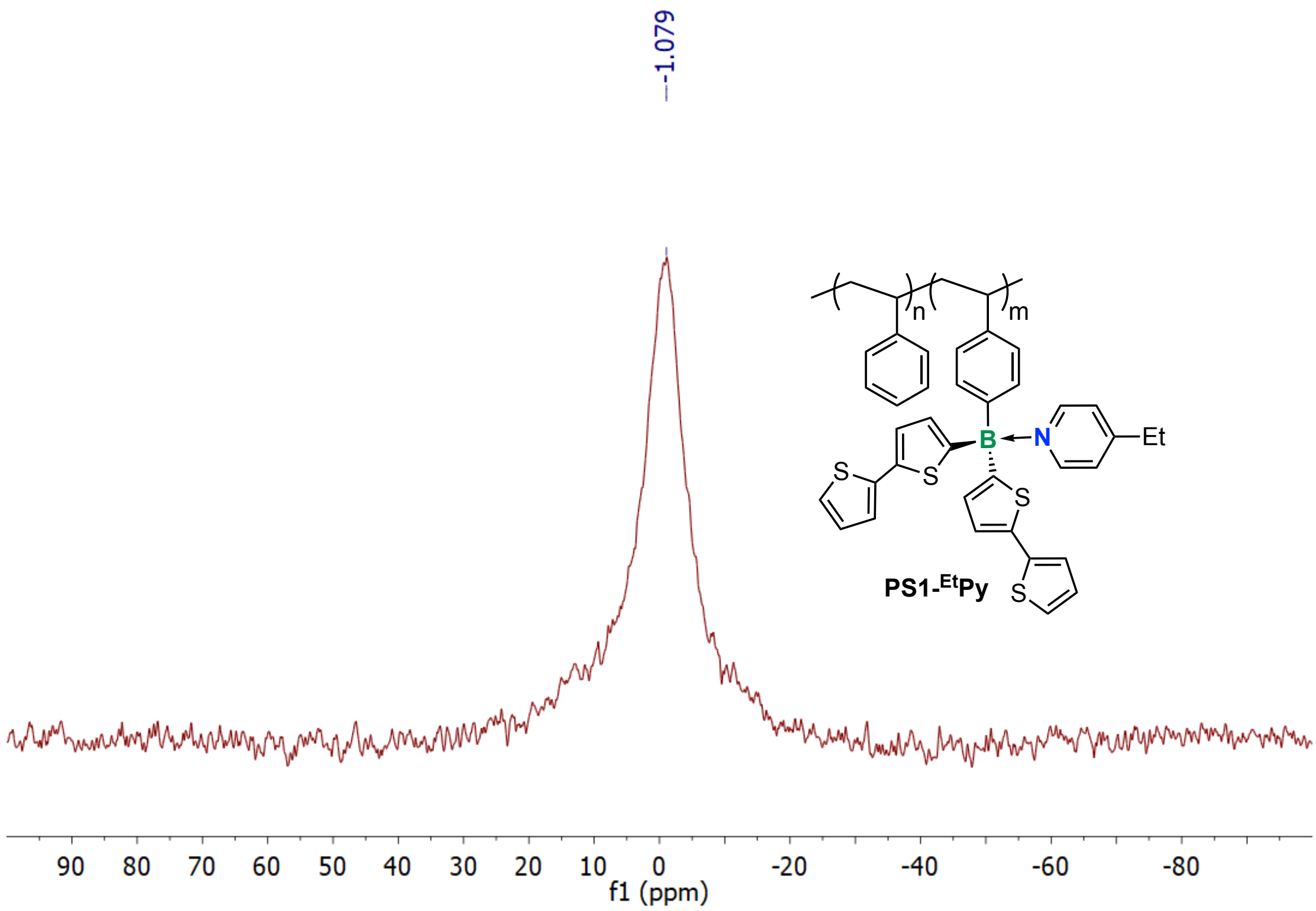

Figure S65. ^{11}B NMR spectrum of **PS1-^{Et}Py** in C_6D_6 at 25 °C.

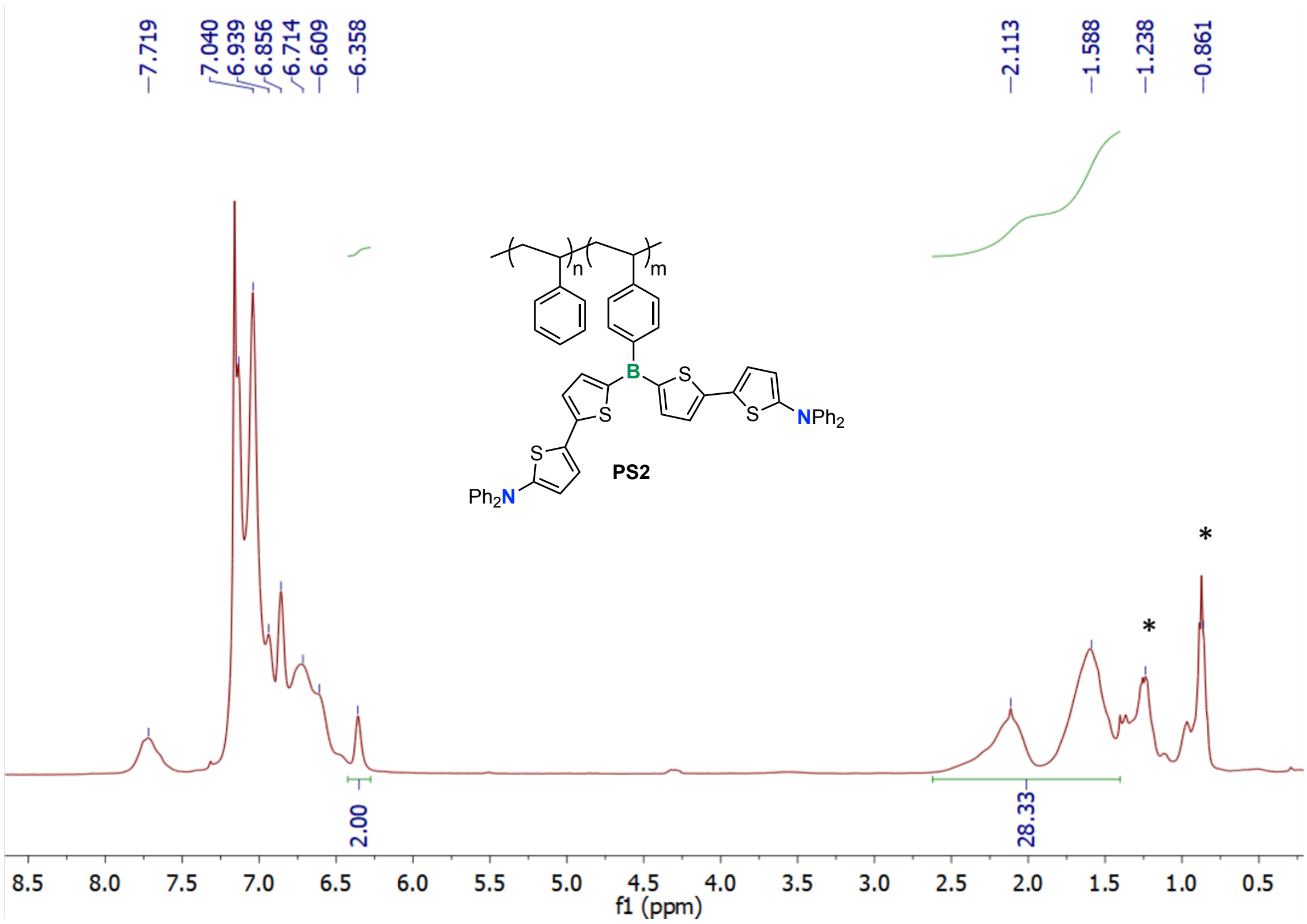

Figure S66. ^{1}H NMR spectrum of **PS2** in C_6D_6 at 25 °C; * residual hexanes.

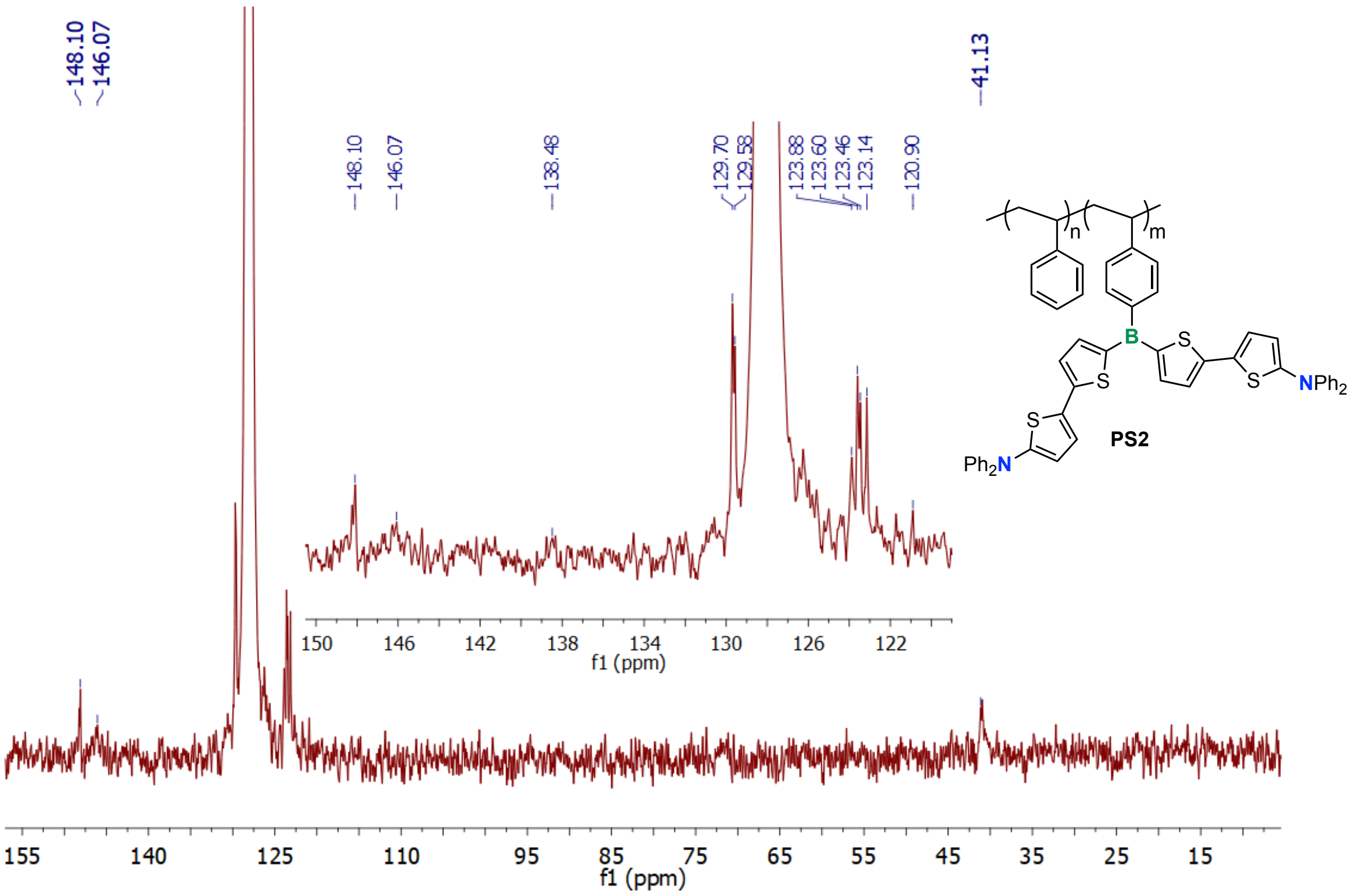

Figure S67. ^{13}C NMR spectrum of **PS2** in C_6D_6 at 25 °C.

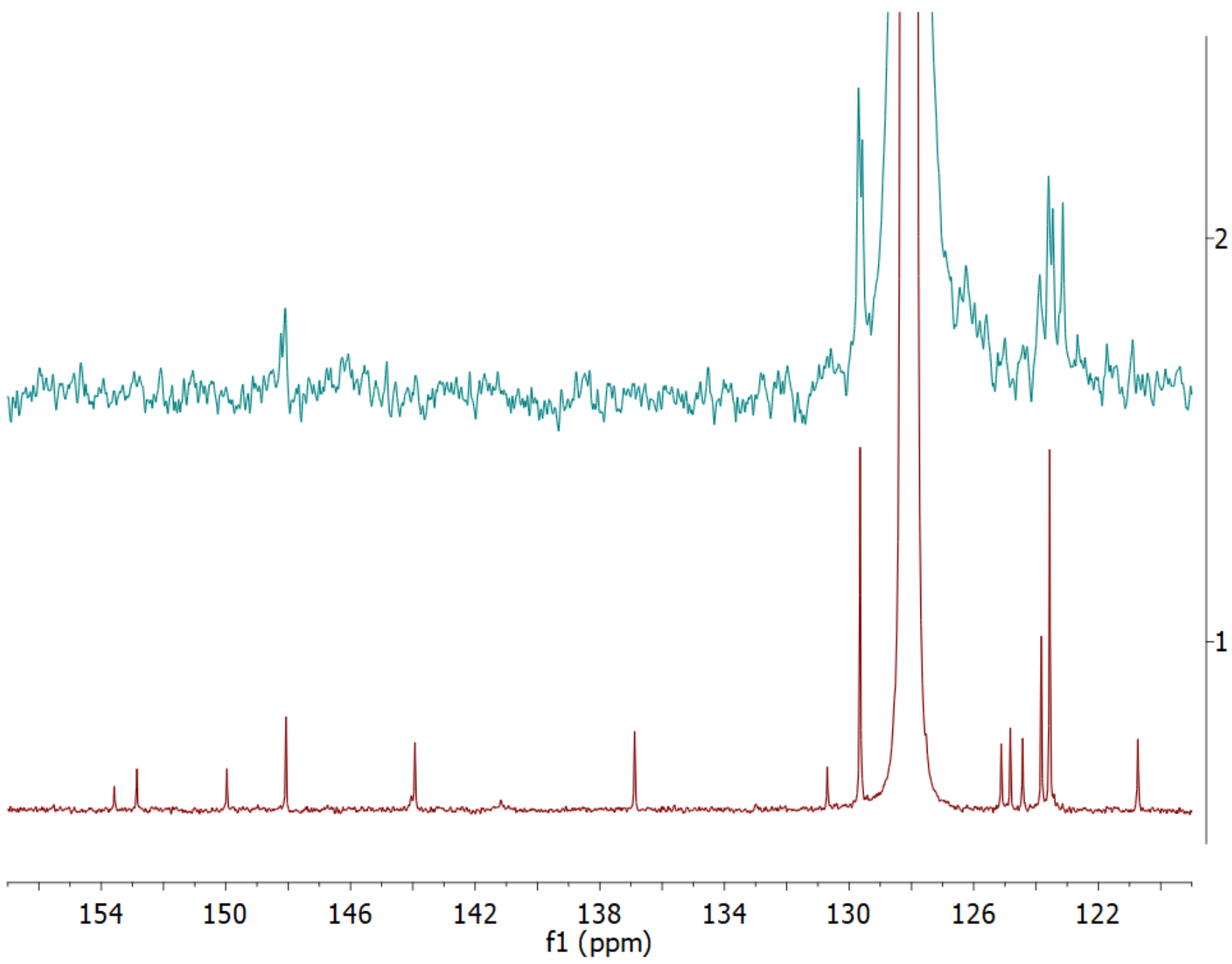

Figure S68. Stacked ^{13}C NMR spectra of **PS2** and **M2** in C_6D_6 at 25 °C. Trace 1 for **M2**, Trace 2 for **PS2**.

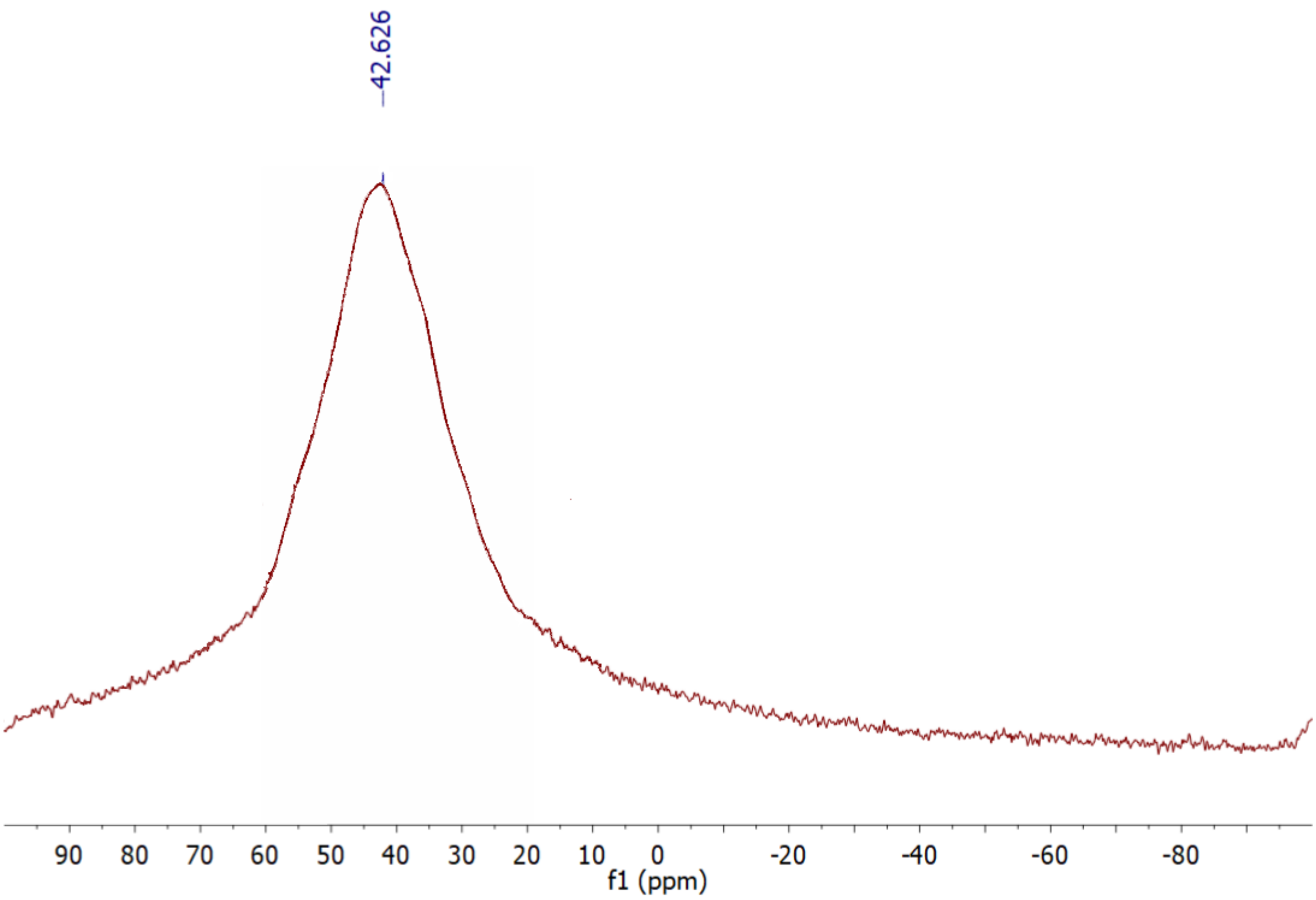

Figure S69. ^{11}B NMR spectrum of **PS2** in C_6D_6 at 25 °C

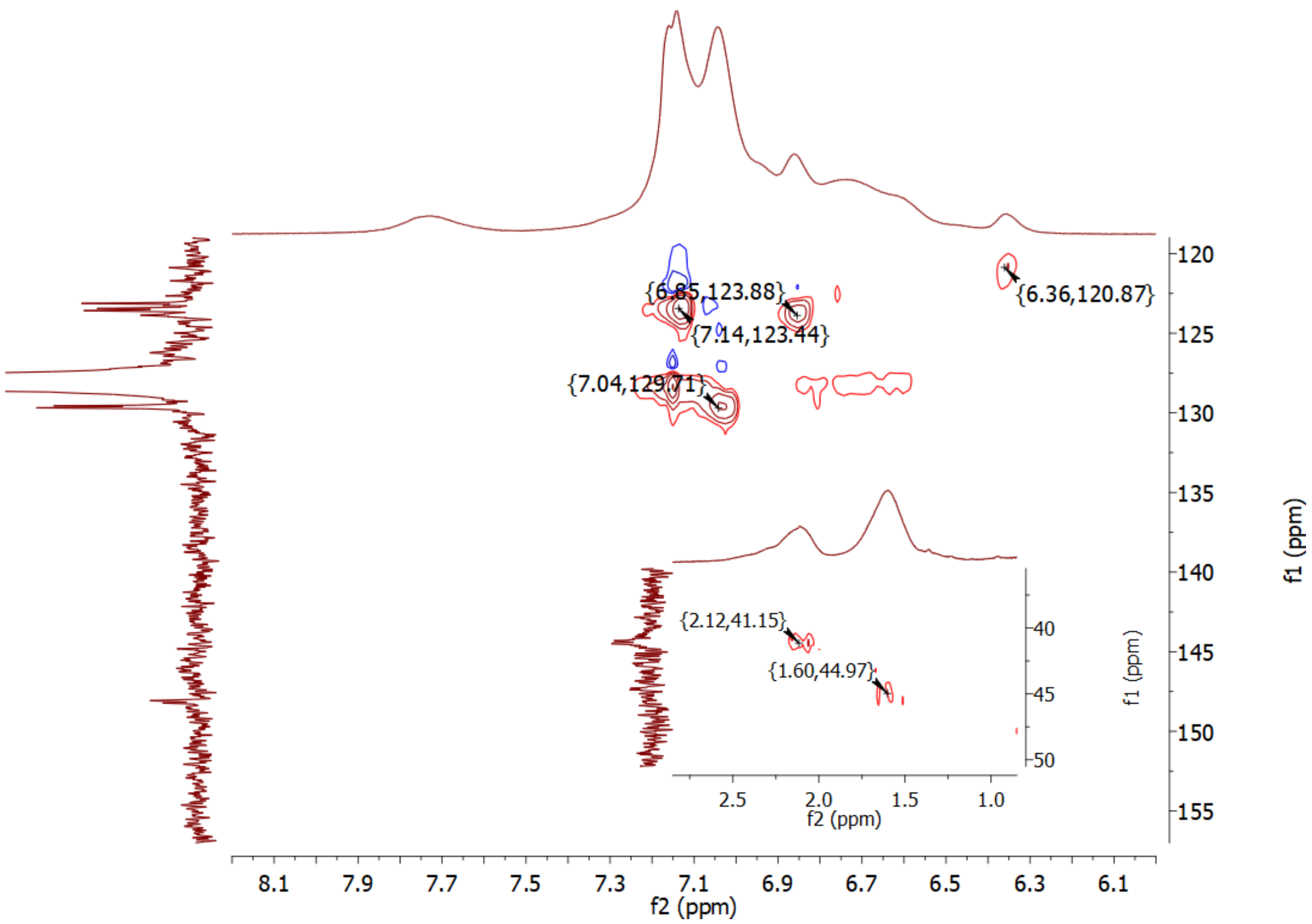

Figure S70. HSQC NMR spectrum of **PS2** in C_6D_6 at 25 °C.

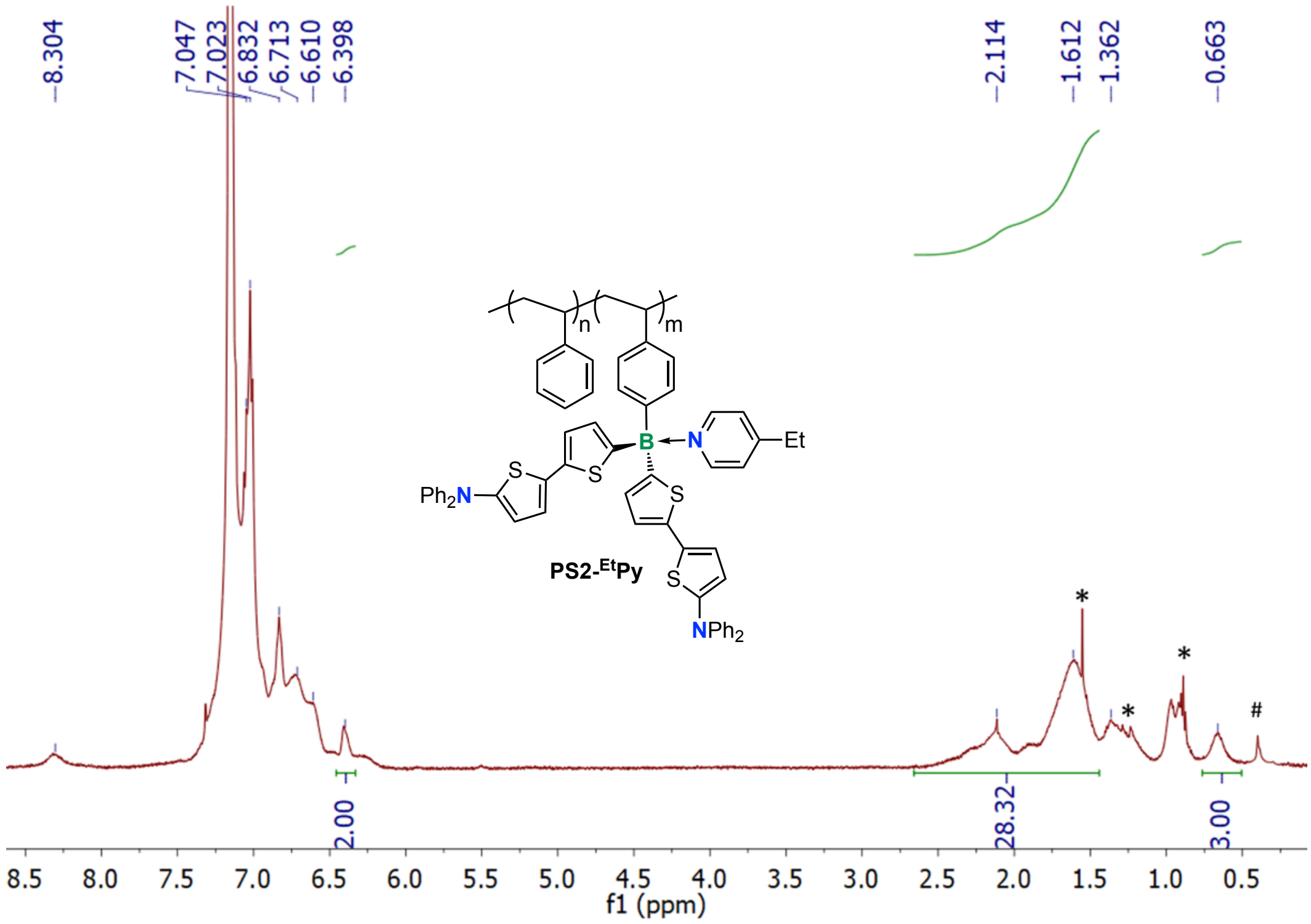

Figure S71. ^{1}H NMR spectrum of **PS2-^{Et}Py** in C_6D_6 at 25 °C. * hexanes & acetone, # H_2O in deuterated solvent.

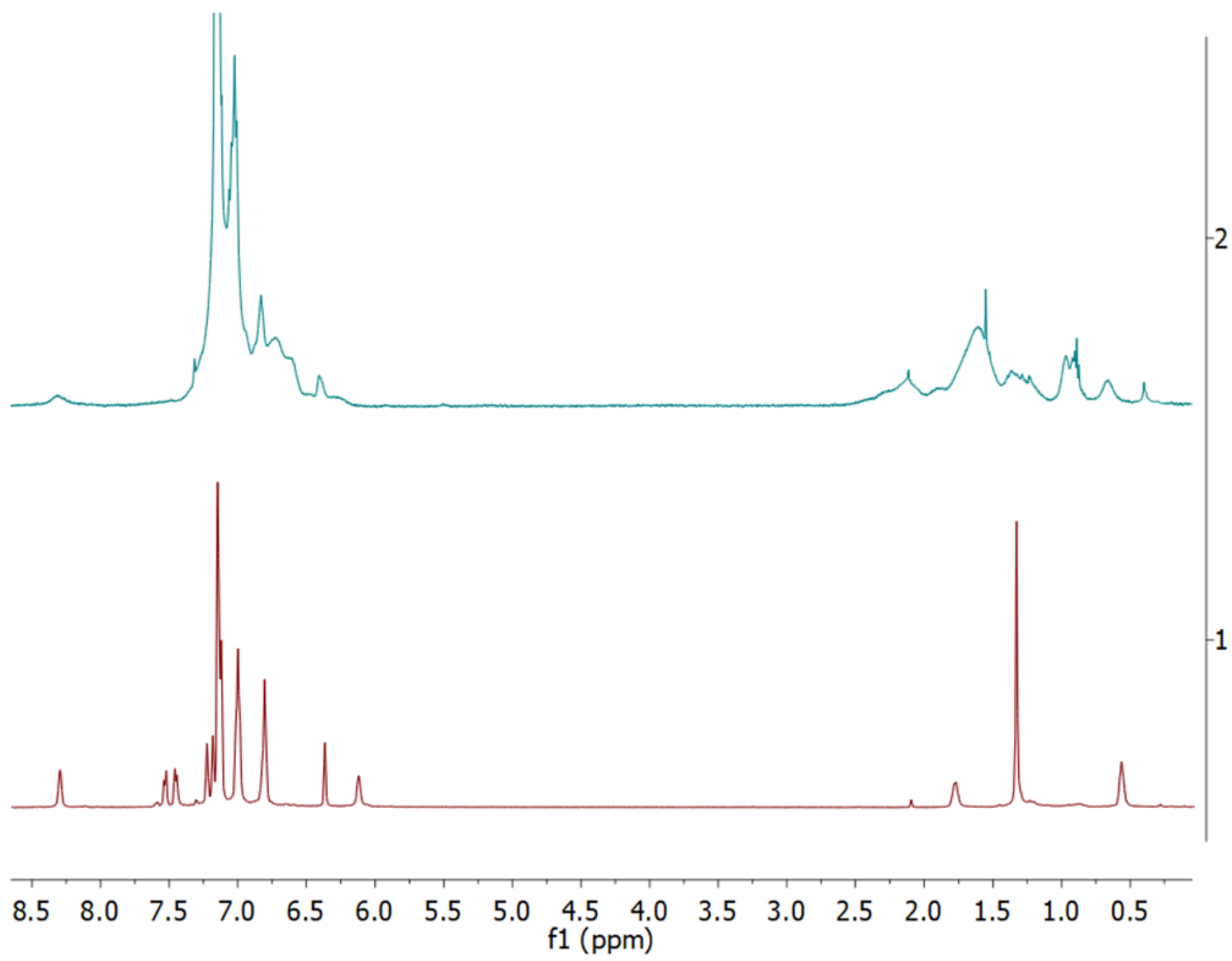

Figure S72. Stacked ^{1}H NMR spectra of **PS2-^{Et}Py** and **M2-^{Et}Py** in C_6D_6 at 25 °C. Trace 1 for **M2-^{Et}Py**, Trace 2 for **PS2-^{Et}Py**.

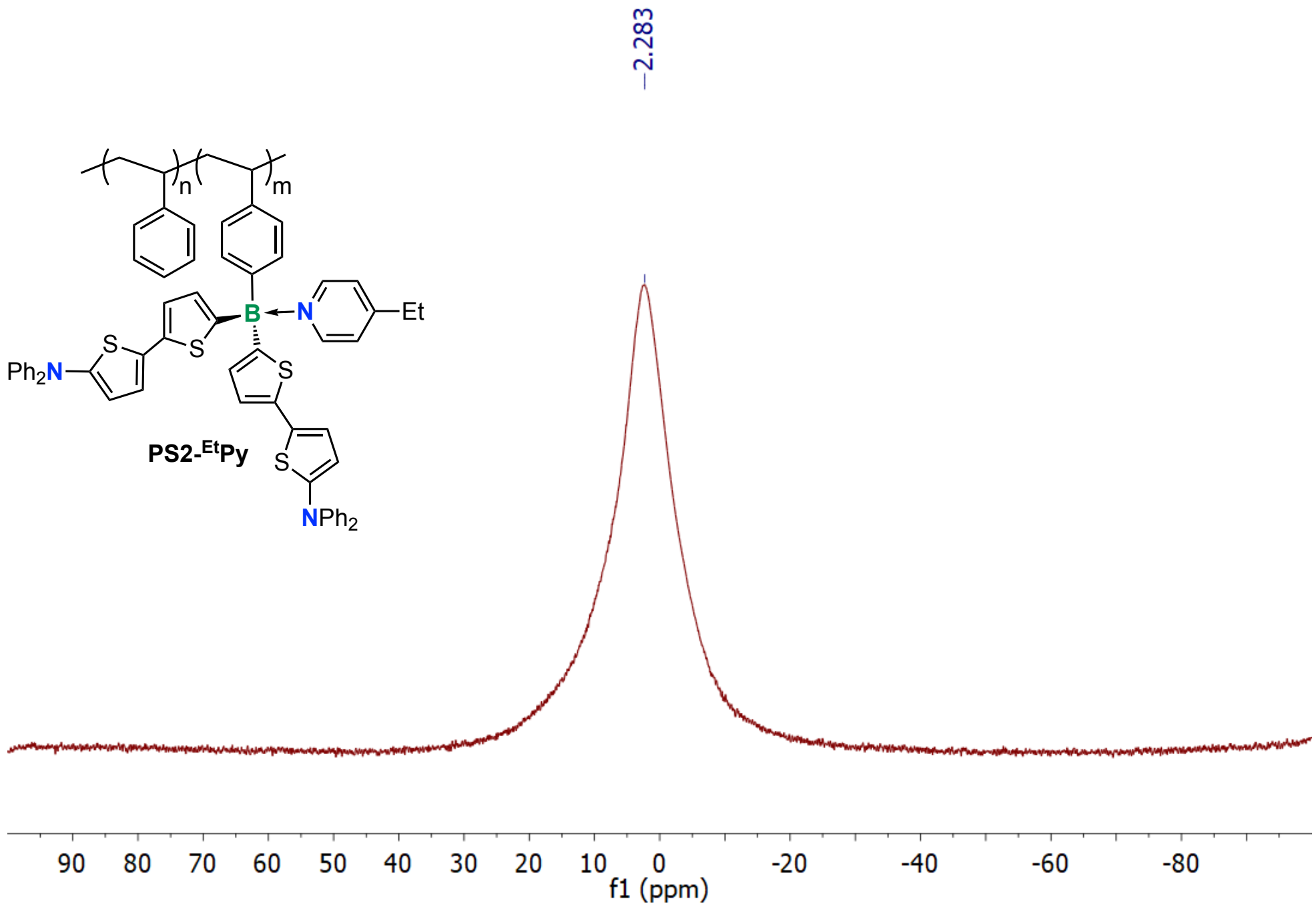

Figure S73. ^{11}B NMR spectrum of **PS2-^{Et}Py** in C_6D_6 at 25 °C.

11. Coordinated for Optimized Structures

Table S17. Coordinates for Optimized Structure of **M1** in the ground state (S_0).
E(RB3LYP) = -2464.98424141 Hartree

```
# opt freq rb3lyp/6-31g(d) geom=connectivity
```

Center Number	Atomic Number	Atomic Type	Coordinates (Angstroms) X	Y	Z
1	6	0	-0.084303	4.989748	-0.970845
2	6	0	-0.393288	3.637807	-0.825030
3	6	0	0.327251	2.804301	0.057238
4	6	0	1.368782	3.403250	0.798091
5	6	0	1.666611	4.760224	0.676134
6	6	0	0.944228	5.556391	-0.214892
7	5	0	-0.019279	1.278690	0.213798
8	6	0	1.102646	0.218823	0.333363
9	6	0	-1.509603	0.861462	0.242187
10	6	0	1.078093	-1.049497	0.899250
11	6	0	2.299641	-1.753121	0.838731
12	6	0	3.307413	-1.044037	0.205244
13	16	0	2.718649	0.517612	-0.300411
14	16	0	-2.082596	-0.754083	-0.161593
15	6	0	-3.751410	-0.338487	0.135119
16	6	0	-3.851845	0.987808	0.522089
17	6	0	-2.605932	1.649090	0.568130
18	6	0	4.672209	-1.454969	-0.052147
19	6	0	-4.811922	-1.308915	-0.040389
20	16	0	5.151337	-3.139688	0.088671
21	6	0	6.788328	-2.766051	-0.342736
22	6	0	6.954314	-1.430336	-0.590116
23	6	0	5.754264	-0.684770	-0.424785
24	6	0	-4.720907	-2.679297	-0.170280
25	6	0	-5.980245	-3.321784	-0.326872
26	6	0	-7.029746	-2.443598	-0.314875
27	16	0	-6.493818	-0.806325	-0.120999
28	1	0	-0.646653	5.602373	-1.671147
29	1	0	-1.198726	3.211282	-1.416774
30	1	0	1.942308	2.797951	1.494711
31	1	0	2.464370	5.195919	1.272458
32	1	0	1.181455	6.612150	-0.319913
33	1	0	0.192436	-1.448225	1.381062
34	1	0	2.447880	-2.741957	1.260399
35	1	0	-4.795808	1.459598	0.774998
36	1	0	-2.495033	2.688098	0.858420
37	1	0	7.527104	-3.554840	-0.388668
38	1	0	7.903011	-0.988870	-0.875133
39	1	0	5.690098	0.389620	-0.560443
40	1	0	-3.775163	-3.209729	-0.138557
41	1	0	-6.099600	-4.394004	-0.437458
42	1	0	-8.085916	-2.657325	-0.409254

Table S18. Coordinates for Optimized Structure of **M2** in the ground state (S_0). E(RB3LYP) = -3499.87885722 Hartree

```
# opt freq rb3lyp/6-31g(d) geom=connectivity
```

Center Number	Atomic Number	Atomic Type	X	Y	Z
1	6	0	-0.300471	6.691871	0.949695
2	6	0	-0.520488	5.326769	0.768131
3	6	0	0.255690	4.564203	-0.130689
4	6	0	1.259114	5.248070	-0.849974
5	6	0	1.468231	6.617947	-0.692063
6	6	0	0.692255	7.343099	0.214382
7	5	0	0.008582	3.022693	-0.328551
8	6	0	-1.451842	2.516981	-0.392336
9	6	0	1.197451	2.040637	-0.451860
10	6	0	1.263766	0.783137	-1.038799
11	6	0	2.527913	0.161134	-0.978106
12	6	0	3.482416	0.922377	-0.319978
13	16	0	2.785398	2.434777	0.204712
14	6	0	4.864980	0.596078	-0.057120
15	16	0	5.476730	-1.031253	-0.336089
16	6	0	7.069649	-0.590532	0.288264
17	6	0	7.099434	0.736418	0.652619
18	6	0	5.864365	1.401006	0.449974
19	16	0	-1.932882	0.856249	-0.049796
20	6	0	-3.621405	1.180562	-0.361373
21	6	0	-3.793666	2.511932	-0.708553
22	6	0	-2.589885	3.247047	-0.710660
23	6	0	-4.623398	0.147642	-0.237288
24	6	0	-4.451972	-1.208278	-0.047324
25	6	0	-5.660859	-1.931152	0.107564
26	6	0	-6.783300	-1.138878	0.023343
27	16	0	-6.338340	0.545679	-0.269893
28	7	0	8.124702	-1.503116	0.282135
29	6	0	9.395013	-1.103297	-0.230180
30	6	0	7.931877	-2.819075	0.796322
31	7	0	-8.119508	-1.537883	0.050455
32	6	0	-9.064918	-0.812624	0.833803
33	6	0	-8.525599	-2.680600	-0.701694
34	6	0	8.482690	-3.927454	0.134811
35	6	0	8.285316	-5.210706	0.640216
36	6	0	7.526579	-5.410379	1.795886
37	6	0	6.971943	-4.308311	2.449338
38	6	0	7.177812	-3.018827	1.962086
39	6	0	9.480522	-0.322463	-1.392617
40	6	0	10.724995	0.071892	-1.879633
41	6	0	11.898702	-0.314391	-1.228421
42	6	0	11.813675	-1.096598	-0.075069
43	6	0	10.573417	-1.483970	0.429500
44	6	0	-9.422420	-3.606983	-0.148444
45	6	0	-9.822122	-4.719803	-0.886560
46	6	0	-9.324715	-4.934937	-2.173264
47	6	0	-8.424988	-4.017123	-2.720074
48	6	0	-8.030517	-2.893295	-1.997004
49	6	0	-10.348825	-0.549121	0.332183
50	6	0	-11.265470	0.164665	1.101202
51	6	0	-10.914112	0.638941	2.367182
52	6	0	-9.634121	0.383576	2.862755
53	6	0	-8.715261	-0.344519	2.108990
54	1	0	-0.904552	7.248770	1.661784
55	1	0	-1.299555	4.834207	1.343782
56	1	0	1.874025	4.698716	-1.557628
57	1	0	2.238887	7.119129	-1.272493
58	1	0	0.860209	8.409008	0.347274
59	1	0	0.411020	0.334749	-1.536159
60	1	0	2.743384	-0.809120	-1.413845

Coordinates (Angstroms)

61	1	0	7.988351	1.212196	1.050425
62	1	0	5.705511	2.446122	0.694367
63	1	0	-4.760045	2.936179	-0.961201
64	1	0	-2.537760	4.300114	-0.964705
65	1	0	-3.472457	-1.672200	0.003080
66	1	0	-5.713175	-3.001062	0.272807
67	1	0	9.060262	-3.777087	-0.771571
68	1	0	8.716344	-6.060366	0.117346
69	1	0	7.369207	-6.413406	2.181942
70	1	0	6.384437	-4.448136	3.352791
71	1	0	6.758919	-2.161689	2.479812
72	1	0	8.571459	-0.027468	-1.906706
73	1	0	10.775424	0.675439	-2.782050
74	1	0	12.866865	-0.008880	-1.614615
75	1	0	12.717633	-1.398297	0.447473
76	1	0	10.511525	-2.080660	1.333847
77	1	0	-9.801185	-3.450600	0.856452
78	1	0	-10.516678	-5.429223	-0.444396
79	1	0	-9.633441	-5.806907	-2.742627
80	1	0	-8.034290	-4.167778	-3.722974
81	1	0	-7.338307	-2.177857	-2.429116
82	1	0	-10.620024	-0.903315	-0.657119
83	1	0	-12.255897	0.362941	0.700184
84	1	0	-11.629697	1.201422	2.959977
85	1	0	-9.349073	0.741177	3.848557
86	1	0	-7.725780	-0.555824	2.502010

Table S19. Coordinates for Optimized Structure of **M1-Py** in the ground state (S_0).
E(RB3LYP) = -2713.27924058 Hartree

```
# opt freq b3lyp/6-31g(d) geom=connectivity
```

Center Number	Atomic Number	Atomic Type	Coordinates (Angstroms) X	Y	Z
1	6	0	1.023829	4.543508	-1.932450
2	6	0	0.661299	3.666921	-0.904887
3	6	0	0.392926	2.307667	-1.140792
4	6	0	0.498698	1.873971	-2.479388
5	6	0	0.864472	2.736872	-3.512184
6	6	0	1.130533	4.081218	-3.242629
7	5	0	-0.023601	1.217352	-0.002398
8	6	0	-1.469098	0.566725	-0.340357
9	6	0	1.127155	0.111258	0.300409
10	6	0	1.192619	-0.836287	1.302606
11	6	0	2.348764	-1.662298	1.278595
12	6	0	3.212127	-1.356967	0.248332
13	16	0	2.573165	-0.016618	-0.682723
14	6	0	-2.478899	1.084374	-1.126347
15	6	0	-3.662758	0.298663	-1.188198
16	6	0	-3.588280	-0.856101	-0.439788
17	16	0	-2.021752	-0.956173	0.340619
18	6	0	4.475380	-1.979494	-0.097912
19	6	0	-4.582045	-1.898849	-0.270274
20	16	0	5.343118	-2.969008	1.068308
21	6	0	6.617579	-3.290642	-0.067007
22	6	0	6.394414	-2.664140	-1.261522
23	6	0	5.179646	-1.920278	-1.281085
24	16	0	-6.276880	-1.594467	-0.625222
25	6	0	-6.716686	-3.219048	-0.197915
26	6	0	-5.629347	-3.943643	0.204928
27	6	0	-4.417400	-3.196258	0.164478
28	7	0	-0.188860	2.068399	1.447363
29	6	0	-1.386811	2.513142	1.872310
30	6	0	-1.525612	3.270562	3.029404
31	6	0	-0.390784	3.588502	3.772443
32	6	0	0.850220	3.137835	3.322705
33	6	0	0.913077	2.380493	2.160973
34	1	0	1.220838	5.588853	-1.704966
35	1	0	0.585734	4.072685	0.101440
36	1	0	0.280583	0.835759	-2.717218
37	1	0	0.937410	2.360009	-4.529733
38	1	0	1.412865	4.758492	-4.044815
39	1	0	0.415525	-0.946507	2.053902
40	1	0	2.527813	-2.469141	1.982805
41	1	0	-2.359794	2.015480	-1.671085
42	1	0	-4.529348	0.560369	-1.787673
43	1	0	7.069090	-2.735110	-2.108052
44	1	0	4.823126	-1.372103	-2.146605
45	1	0	-5.684928	-4.982182	0.513345
46	1	0	-3.447948	-3.606095	0.427035
47	1	0	-2.231440	2.236002	1.253260
48	1	0	-2.513726	3.597376	3.334779
49	1	0	-0.470173	4.175671	4.682604
50	1	0	1.764495	3.362404	3.861286
51	1	0	1.846761	1.994900	1.771913
52	1	0	7.448565	-3.921598	0.218136
53	1	0	-7.747943	-3.536797	-0.272297

Table S20. Coordinates for Optimized Structure of **M2-Py** in the ground state (S_0).
E(RB3LYP) = -3748.17290757 Hartree

```
# opt freq b3lyp/6-31g(d) geom=connectivity
```

Center Number	Atomic Number	Atomic Type	Coordinates (Angstroms) X	Y	Z
1	6	0	0.887459	6.366165	-2.250086
2	6	0	0.584252	5.524571	-1.175159
3	6	0	0.352185	4.148775	-1.344283
4	6	0	0.431431	3.660580	-2.665839
5	6	0	0.738347	4.488352	-3.745513
6	6	0	0.969324	5.850681	-3.542092
7	5	0	0.007060	3.096360	-0.147739
8	6	0	-1.421915	2.379595	-0.414301
9	6	0	1.205088	2.046393	0.169328
10	6	0	-2.473032	2.825350	-1.190351
11	6	0	-3.625979	1.993165	-1.186015
12	6	0	-3.486399	0.874143	-0.392755
13	16	0	-1.895291	0.867163	0.345737
14	6	0	-4.433066	-0.196199	-0.155335
15	7	0	-0.150609	4.005696	1.267677
16	6	0	-1.352257	4.429686	1.703414
17	6	0	-1.488290	5.234035	2.828711
18	6	0	-0.347026	5.622651	3.526975
19	6	0	0.897153	5.193050	3.065629
20	6	0	0.957183	4.386069	1.937607
21	16	0	-6.144904	0.027689	-0.494374
22	6	0	-6.526558	-1.590627	0.111587
23	6	0	-5.384627	-2.234305	0.520557
24	6	0	-4.208781	-1.453430	0.363363
25	6	0	1.335977	1.150822	1.211883
26	6	0	2.521253	0.367714	1.191504
27	6	0	3.340938	0.650694	0.118973
28	16	0	2.622494	1.919394	-0.856025
29	6	0	4.612546	0.056276	-0.238195
30	16	0	5.450610	-1.015691	0.876779
31	6	0	6.731134	-1.324163	-0.303600
32	6	0	6.515606	-0.599822	-1.449470
33	6	0	5.329072	0.180038	-1.409689
34	1	0	1.057756	7.425979	-2.073713
35	1	0	0.526377	5.971404	-0.185167
36	1	0	0.239634	2.606764	-2.852146
37	1	0	0.792607	4.069835	-4.747839
38	1	0	1.205541	6.500666	-4.380922
39	1	0	-2.405559	3.736525	-1.776135
40	1	0	-4.518014	2.196190	-1.770728
41	1	0	-2.201954	4.096847	1.119691
42	1	0	-2.479152	5.541831	3.144773
43	1	0	-0.423982	6.247764	4.411725
44	1	0	1.816185	5.471870	3.569658
45	1	0	1.893501	4.013911	1.541880
46	1	0	-5.395780	-3.244912	0.913439
47	1	0	-3.219038	-1.803748	0.636407
48	1	0	0.587660	1.051074	1.993269
49	1	0	2.754179	-0.390738	1.932710
50	1	0	7.191304	-0.636510	-2.296777
51	1	0	4.986851	0.785153	-2.242513
52	7	0	7.824352	-2.140378	0.011895
53	7	0	-7.828690	-2.099698	0.050845
54	6	0	-8.894790	-1.378348	0.658982
55	6	0	-10.978747	0.081142	1.853274
56	6	0	-8.689209	-0.712204	1.876925
57	6	0	-10.154403	-1.304236	0.042824
58	6	0	-11.186040	-0.586390	0.643994
59	6	0	-9.723564	0.016453	2.461076
60	1	0	-7.717989	-0.771845	2.357802

61	1	0	-10.317575	-1.807256	-0.904667
62	1	0	-12.154809	-0.537715	0.153571
63	1	0	-9.547383	0.526531	3.404575
64	1	0	-11.784491	0.645169	2.314392
65	6	0	-8.064714	-3.328416	-0.632226
66	6	0	-8.511501	-5.763529	-1.963382
67	6	0	-7.378634	-3.625164	-1.819767
68	6	0	-8.973662	-4.264935	-0.115114
69	6	0	-9.197569	-5.466997	-0.784035
70	6	0	-7.599210	-4.836525	-2.472052
71	1	0	-6.673480	-2.905856	-2.223424
72	1	0	-9.498944	-4.047201	0.809182
73	1	0	-9.903735	-6.181686	-0.369244
74	1	0	-7.060008	-5.050059	-3.391287
75	1	0	-8.683758	-6.704581	-2.477833
76	6	0	9.141751	-1.607936	-0.090265
77	6	0	11.736341	-0.547971	-0.318126
78	6	0	9.403778	-0.281598	0.286510
79	6	0	10.191636	-2.396777	-0.587132
80	6	0	11.477302	-1.868660	-0.689792
81	6	0	10.689978	0.240323	0.165682
82	1	0	8.596198	0.334042	0.669085
83	1	0	9.994735	-3.419262	-0.892482
84	1	0	12.277807	-2.493116	-1.078107
85	1	0	10.875105	1.269313	0.463018
86	1	0	12.738475	-0.138240	-0.406348
87	6	0	7.600231	-3.484743	0.424478
88	6	0	7.118430	-6.125244	1.257741
89	6	0	6.572708	-4.239056	-0.162312
90	6	0	8.382809	-4.064658	1.435944
91	6	0	8.145048	-5.377087	1.838475
92	6	0	6.332906	-5.545352	0.259897
93	1	0	5.968117	-3.794826	-0.946612
94	1	0	9.171308	-3.483497	1.902865
95	1	0	8.759078	-5.811134	2.623363
96	1	0	5.532362	-6.115149	-0.204553
97	1	0	6.932293	-7.145668	1.580324

Table S21. Coordinates for Optimized Structure of **M1** in the first singlet excited state (S_1). E(RB3LYP) = -2464.96246385 Hartree; E(TD-HF/TD-DFT) = -2464.89487303 Hartree

```
# opt td rb3lyp/6-31g(d) geom=connectivity
```

Center Number	Atomic Number	Atomic Type	Coordinates (Angstroms) X	Y	Z
1	6	0	-0.027325	5.395603	-0.967919
2	6	0	-0.361956	4.049592	-0.831440
3	6	0	0.347534	3.183870	0.033370
4	6	0	1.436909	3.757234	0.729092
5	6	0	1.778682	5.102242	0.597470
6	6	0	1.044519	5.933752	-0.251095
7	5	0	-0.050495	1.668861	0.180571
8	6	0	1.175399	0.666348	0.468305
9	6	0	-1.420810	1.111087	0.080340
10	6	0	1.724352	0.254710	1.706184
11	6	0	2.798386	-0.615240	1.606723
12	6	0	3.140425	-0.922390	0.268251
13	16	0	2.049298	-0.067921	-0.827424
14	16	0	-1.784552	-0.650056	0.098503
15	6	0	-3.532519	-0.397530	0.018830
16	6	0	-3.795371	0.982327	-0.018670
17	6	0	-2.670290	1.790319	0.015885
18	6	0	4.167376	-1.764180	-0.194697
19	6	0	-4.445334	-1.487100	0.005711
20	16	0	5.263973	-2.599189	0.916349
21	6	0	6.102761	-3.324527	-0.399269
22	6	0	5.586161	-2.959610	-1.631862
23	6	0	4.499554	-2.083551	-1.524455
24	6	0	-4.193033	-2.855576	0.068379
25	6	0	-5.363007	-3.656409	0.034014
26	6	0	-6.521413	-2.931123	-0.056224
27	16	0	-6.194089	-1.217686	-0.102269
28	1	0	-0.600069	6.026300	-1.644320
29	1	0	-1.180458	3.647562	-1.420851
30	1	0	2.026795	3.141500	1.405466
31	1	0	2.618238	5.504349	1.160625
32	1	0	1.307798	6.983066	-0.357539
33	1	0	1.305224	0.595912	2.644628
34	1	0	3.326831	-1.032931	2.457571
35	1	0	-4.808566	1.371903	-0.055535
36	1	0	-2.724427	2.872756	0.036674
37	1	0	6.936497	-3.986512	-0.206152
38	1	0	5.986568	-3.320287	-2.571440
39	1	0	3.953971	-1.683338	-2.370725
40	1	0	-3.188463	-3.257753	0.138775
41	1	0	-5.346267	-4.741097	0.075184
42	1	0	-7.541261	-3.287191	-0.099313

Table S22. Coordinates for Optimized Structure of **M1** in the first singlet excited state (S_1) with RCAM-B3LYP functional.

E(RCAM-B3LYP) = -2464.42907980 Hartree; E(TD-HF/TD-DFT) = -2464.31544514 Hartree

```
# opt td rcam-b3lyp/6-31g(d) geom=connectivity
```

Center Number	Atomic Number	Atomic Type	Coordinates (Angstroms) X	Y	Z
1	6	0	0.098491	4.907014	-0.997744
2	6	0	-0.227478	3.559318	-0.903975
3	6	0	0.350247	2.722287	0.064850
4	6	0	1.273755	3.315056	0.941185
5	6	0	1.598220	4.664333	0.862044
6	6	0	1.012737	5.466320	-0.111310
7	5	0	-0.020322	1.192040	0.163103
8	6	0	1.087722	0.133083	0.231026
9	6	0	-1.501093	0.800772	0.160314
10	6	0	1.037123	-1.236425	0.568347
11	6	0	2.249600	-1.904278	0.495111
12	6	0	3.311441	-1.085014	0.098148
13	16	0	2.744206	0.544381	-0.175786
14	16	0	-2.123580	-0.824734	-0.100266
15	6	0	-3.786083	-0.313361	0.037714
16	6	0	-3.844654	1.068776	0.263526
17	6	0	-2.598982	1.670579	0.326377
18	6	0	4.675674	-1.439119	-0.076613
19	6	0	-4.861028	-1.234021	-0.067080
20	16	0	5.240498	-3.077045	0.192872
21	6	0	6.855968	-2.637993	-0.205939
22	6	0	6.965053	-1.312093	-0.528880
23	6	0	5.734775	-0.627535	-0.459469
24	6	0	-4.808479	-2.604364	-0.286526
25	6	0	-6.079953	-3.211875	-0.327416
26	6	0	-7.103272	-2.321466	-0.143965
27	16	0	-6.529469	-0.714127	0.083876
28	1	0	-0.360904	5.522717	-1.765975
29	1	0	-0.942255	3.139352	-1.606601
30	1	0	1.740271	2.707148	1.711566
31	1	0	2.309604	5.091374	1.563301
32	1	0	1.267105	6.520027	-0.178939
33	1	0	0.122079	-1.713283	0.894824
34	1	0	2.376559	-2.955322	0.730212
35	1	0	-4.781694	1.599807	0.389872
36	1	0	-2.450501	2.727844	0.505959
37	1	0	7.640171	-3.381903	-0.183021
38	1	0	7.901123	-0.844381	-0.809416
39	1	0	5.612807	0.426198	-0.680554
40	1	0	-3.875386	-3.139945	-0.415649
41	1	0	-6.237356	-4.271669	-0.488625
42	1	0	-8.165223	-2.523703	-0.131961

Table S23. Coordinates for Optimized Structure of **M2** in the first singlet excited state (S_1). E(RB3LYP) = -3499.85391163 Hartree; E(TD-HF/TD-DFT) = -3499.80513595 Hartree

opt td rb3lyp/6-31g(d) geom=connectivity

Center Number	Atomic Number	Atomic Type	Coordinates (Angstroms) X	Y	Z
1	6	0	-0.283540	7.451306	0.266856
2	6	0	-0.536400	6.080449	0.253544
3	6	0	0.226234	5.183418	-0.529737
4	6	0	1.281552	5.755039	-1.276784
5	6	0	1.539901	7.124889	-1.271553
6	6	0	0.754714	7.985130	-0.500464
7	5	0	-0.071598	3.637313	-0.542894
8	6	0	-1.415701	3.021203	-0.393915
9	6	0	1.206501	2.681597	-0.748751
10	6	0	1.768218	2.187856	-1.932166
11	6	0	2.895306	1.380264	-1.754319
12	6	0	3.252322	1.219361	-0.407183
13	16	0	2.123798	2.117741	0.607192
14	6	0	4.322021	0.476643	0.134034
15	16	0	5.461753	-0.392397	-0.906983
16	6	0	6.337446	-0.999052	0.496296
17	6	0	5.761604	-0.537518	1.692877
18	6	0	4.655101	0.278019	1.485562
19	16	0	-1.682600	1.248815	-0.257592
20	6	0	-3.442598	1.412623	-0.200272
21	6	0	-3.781029	2.775754	-0.288047
22	6	0	-2.699546	3.634449	-0.392236
23	6	0	-4.295129	0.284488	-0.090586
24	6	0	-3.963215	-1.070616	-0.034322
25	6	0	-5.073402	-1.932009	0.115800
26	6	0	-6.283789	-1.283400	0.172555
27	16	0	-6.055208	0.471990	0.018964
28	7	0	7.448140	-1.773310	0.359655
29	6	0	8.375392	-1.919001	1.451594
30	6	0	7.745772	-2.447963	-0.877559
31	7	0	-7.569722	-1.849251	0.245788
32	6	0	-8.377312	-1.587667	1.385201
33	6	0	-8.038273	-2.638204	-0.839506
34	6	0	8.984452	-2.235274	-1.492703
35	6	0	9.280864	-2.895476	-2.683288
36	6	0	8.348620	-3.762128	-3.259331
37	6	0	7.117269	-3.975044	-2.637350
38	6	0	6.812520	-3.325546	-1.441246
39	6	0	8.903313	-0.787033	2.083371
40	6	0	9.811788	-0.945488	3.129465
41	6	0	10.201529	-2.222915	3.536249
42	6	0	9.679338	-3.347693	2.893128
43	6	0	8.765248	-3.201724	1.851446
44	6	0	-8.845306	-3.767212	-0.614782
45	6	0	-9.279777	-4.547333	-1.684653
46	6	0	-8.909905	-4.232901	-2.993767
47	6	0	-8.097019	-3.119504	-3.217900
48	6	0	-7.666178	-2.325448	-2.157428
49	6	0	-9.771543	-1.445762	1.274940
50	6	0	-10.542042	-1.169754	2.402410
51	6	0	-9.944743	-1.011867	3.654822
52	6	0	-8.558390	-1.136439	3.763763
53	6	0	-7.779197	-1.425545	2.645679
54	1	0	-0.894247	8.105524	0.885459
55	1	0	-1.331037	5.685714	0.879622
56	1	0	1.911139	5.112440	-1.888871
57	1	0	2.355472	7.523851	-1.871330
58	1	0	0.953895	9.053955	-0.491534
59	1	0	1.334971	2.410609	-2.899982
60	1	0	3.439033	0.911129	-2.568865

61	1	0	6.143265	-0.811994	2.667111
62	1	0	4.074141	0.713961	2.289716
63	1	0	-4.813878	3.111759	-0.288741
64	1	0	-2.810416	4.706526	-0.509227
65	1	0	-2.936877	-1.416781	-0.081388
66	1	0	-4.998116	-3.013061	0.177218
67	1	0	9.700951	-1.556925	-1.040546
68	1	0	10.239478	-2.726601	-3.164477
69	1	0	8.583387	-4.272950	-4.188247
70	1	0	6.393860	-4.656463	-3.074586
71	1	0	5.864686	-3.502625	-0.942833
72	1	0	8.610423	0.204079	1.751340
73	1	0	10.222956	-0.066964	3.617591
74	1	0	10.912154	-2.341762	4.348504
75	1	0	9.978085	-4.343505	3.206433
76	1	0	8.352178	-4.071227	1.350273
77	1	0	-9.124878	-4.031146	0.399893
78	1	0	-9.901542	-5.417371	-1.487483
79	1	0	-9.246541	-4.846806	-3.824562
80	1	0	-7.799350	-2.856995	-4.230136
81	1	0	-7.036180	-1.461417	-2.340089
82	1	0	-10.245451	-1.546342	0.304243
83	1	0	-11.618573	-1.061771	2.294341
84	1	0	-10.549300	-0.789261	4.529650
85	1	0	-8.074205	-1.014064	4.729495
86	1	0	-6.702238	-1.522388	2.734631

Table S24. Coordinates for Optimized Structure of **M2** in the first singlet excited state (S_1) with RCAM-B3LYP functional.

E(RCAM-B3LYP) = -3498.73821490 Hartree; E(TD-HF/TD-DFT) = -3498.63942948 Hartree

```
# opt td rcam-b3lyp/6-31g(d) geom=connectivity
```

Center Number	Atomic Number	Atomic Type	Coordinates (Angstroms) X	Y	Z
1	6	0	-0.138442	6.565818	0.900093
2	6	0	-0.373598	5.198696	0.813571
3	6	0	0.269577	4.394459	-0.141119
4	6	0	1.161623	5.040247	-1.012012
5	6	0	1.394104	6.408977	-0.941835
6	6	0	0.746153	7.177902	0.018535
7	5	0	0.001103	2.840476	-0.231560
8	6	0	-1.454912	2.363428	-0.243438
9	6	0	1.177222	1.859515	-0.289072
10	6	0	1.219016	0.499146	-0.627823
11	6	0	2.474632	-0.092769	-0.550745
12	6	0	3.478607	0.788438	-0.145581
13	16	0	2.803947	2.374956	0.125619
14	6	0	4.859621	0.522272	0.037134
15	16	0	5.517112	-1.088412	-0.211830
16	6	0	7.120458	-0.552716	0.254701
17	6	0	7.125059	0.794815	0.559946
18	6	0	5.865106	1.394669	0.434755
19	16	0	-1.985954	0.712980	0.043600
20	6	0	-3.675551	1.119191	-0.141784
21	6	0	-3.807257	2.486889	-0.404589
22	6	0	-2.592990	3.158277	-0.453645
23	6	0	-4.691579	0.141629	-0.031370
24	6	0	-4.562547	-1.220051	0.230748
25	6	0	-5.780893	-1.902937	0.309711
26	6	0	-6.879748	-1.087886	0.104106
27	16	0	-6.388988	0.568211	-0.205220
28	7	0	8.205988	-1.406918	0.207467
29	6	0	9.476152	-0.910890	-0.198548
30	6	0	8.068515	-2.773530	0.576433
31	7	0	-8.205581	-1.469147	0.040455
32	6	0	-9.231331	-0.603851	0.511680
33	6	0	-8.547597	-2.744548	-0.488577
34	6	0	8.667944	-3.770007	-0.197418
35	6	0	8.530137	-5.102687	0.162550
36	6	0	7.785754	-5.457544	1.283626
37	6	0	7.185815	-4.464580	2.050183
38	6	0	7.329975	-3.126943	1.707237
39	6	0	9.583102	-0.054511	-1.296656
40	6	0	10.826811	0.425233	-1.682282
41	6	0	11.974377	0.051156	-0.989958
42	6	0	11.866520	-0.806807	0.099329
43	6	0	10.625867	-1.282693	0.501227
44	6	0	-9.509319	-3.529566	0.151092
45	6	0	-9.849198	-4.770538	-0.369818
46	6	0	-9.229254	-5.247186	-1.520067
47	6	0	-8.267211	-4.465807	-2.152723
48	6	0	-7.928233	-3.218760	-1.647326
49	6	0	-10.402874	-0.439435	-0.231131
50	6	0	-11.400028	0.408390	0.228450
51	6	0	-11.236925	1.109085	1.419870
52	6	0	-10.068109	0.947588	2.155608
53	6	0	-9.069945	0.090550	1.712087
54	1	0	-0.646488	7.155635	1.658112
55	1	0	-1.068866	4.737075	1.509434
56	1	0	1.677758	4.457290	-1.769713
57	1	0	2.083431	6.876923	-1.639191

58	1	0	0.929270	8.246829	0.079882
59	1	0	0.338065	-0.036796	-0.957360
60	1	0	2.667566	-1.133083	-0.789812
61	1	0	8.021806	1.318050	0.866901
62	1	0	5.674160	2.439202	0.650517
63	1	0	-4.769033	2.962747	-0.562702
64	1	0	-2.504092	4.218581	-0.655146
65	1	0	-3.599054	-1.691517	0.383394
66	1	0	-5.874481	-2.962096	0.513866
67	1	0	9.236090	-3.492534	-1.078514
68	1	0	8.998061	-5.870219	-0.445967
69	1	0	7.674749	-6.501629	1.557684
70	1	0	6.609356	-4.729100	2.931066
71	1	0	6.877526	-2.350313	2.314002
72	1	0	8.689701	0.228360	-1.842251
73	1	0	10.899084	1.088024	-2.538960
74	1	0	12.945255	0.425725	-1.297479
75	1	0	12.753834	-1.101025	0.651213
76	1	0	10.540143	-1.942434	1.357632
77	1	0	-9.984113	-3.162505	1.054325
78	1	0	-10.596922	-5.373512	0.135922
79	1	0	-9.493808	-6.220165	-1.921056
80	1	0	-7.782634	-4.823429	-3.055885
81	1	0	-7.186644	-2.604562	-2.145954
82	1	0	-10.522104	-0.975313	-1.166382
83	1	0	-12.306132	0.531748	-0.356398
84	1	0	-12.016547	1.776632	1.772101
85	1	0	-9.933431	1.483216	3.090025
86	1	0	-8.165390	-0.050236	2.293403

Table S25. Coordinates for Optimized Structure of **M1-Py** in the first singlet excited state (S_1).

E(RB3LYP) = -2713.24986038 Hartree; E(TD-HF/TD-DFT) = -2713.21577432 Hartree

opt td rb3lyp/6-31g(d) geom=connectivity

Center Number	Atomic Number	Atomic Type	Coordinates (Angstroms) X	Y	Z
1	6	0	1.073433	4.810440	-1.995090
2	6	0	0.618508	3.934280	-1.007323
3	6	0	0.569314	2.538751	-1.197716
4	6	0	1.019511	2.083165	-2.455789
5	6	0	1.479129	2.946826	-3.453020
6	6	0	1.506138	4.322509	-3.227977
7	5	0	0.031595	1.564852	0.001946
8	6	0	-1.201355	0.577081	-0.469257
9	6	0	1.081261	0.352462	0.390960
10	6	0	1.005681	-0.528340	1.482647
11	6	0	2.004486	-1.502620	1.535987
12	6	0	2.894285	-1.436431	0.459078
13	16	0	2.450556	-0.103738	-0.594910
14	6	0	-1.495418	0.066513	-1.740105
15	6	0	-2.551628	-0.850174	-1.791101
16	6	0	-3.118290	-1.112349	-0.541320
17	16	0	-2.306934	-0.163640	0.684455
18	6	0	4.033322	-2.257953	0.195964
19	6	0	-4.216512	-1.977610	-0.229619
20	16	0	4.548524	-3.504114	1.331997
21	6	0	5.864774	-3.961735	0.308249
22	6	0	5.915574	-3.204314	-0.837415
23	6	0	4.882811	-2.241212	-0.904645
24	16	0	-5.096522	-2.833051	-1.494617
25	6	0	-6.143306	-3.574750	-0.334018
26	6	0	-5.843161	-3.189789	0.949453
27	6	0	-4.754095	-2.287857	1.012060
28	7	0	-0.360752	2.358809	1.297931
29	6	0	-1.563551	3.080354	1.350901
30	6	0	-1.845909	3.983402	2.334635
31	6	0	-0.905823	4.275594	3.360149
32	6	0	0.346066	3.612782	3.258479
33	6	0	0.596061	2.715541	2.259485
34	1	0	1.089975	5.879994	-1.796972
35	1	0	0.293413	4.351249	-0.060966
36	1	0	1.027295	1.019200	-2.678901
37	1	0	1.816301	2.541821	-4.404850
38	1	0	1.860735	5.001960	-3.999331
39	1	0	0.230217	-0.433101	2.232820
40	1	0	2.090324	-2.238904	2.328895
41	1	0	-0.958544	0.391228	-2.621454
42	1	0	-2.908506	-1.308072	-2.708327
43	1	0	6.668469	-3.337229	-1.605934
44	1	0	4.752358	-1.552638	-1.732004
45	1	0	-6.384617	-3.543656	1.819474
46	1	0	-4.365646	-1.874430	1.936153
47	1	0	-2.263570	2.879313	0.552458
48	1	0	-2.809654	4.483767	2.307586
49	1	0	-1.113073	4.994876	4.140880
50	1	0	1.138872	3.814731	3.973157
51	1	0	1.558041	2.233792	2.165472
52	1	0	6.526917	-4.762887	0.608058
53	1	0	-6.919830	-4.251630	-0.664174

Table S26. Coordinates for Optimized Structure of **M1-Py** in the first singlet excited state (S_1) with RCAM-B3LYP functional.

E(RCAM-B3LYP) = -2712.56339671 Hartree; E(TD-HF/TD-DFT) = -2712.48729960 Hartree

```
# opt td rcam-b3lyp/6-31g(d) geom=connectivity
```

Center Number	Atomic Number	Atomic Type	Coordinates (Angstroms) X	Y	Z
1	6	0	0.963756	4.906283	-1.937517
2	6	0	0.533294	3.999303	-0.974637
3	6	0	0.549840	2.613967	-1.189101
4	6	0	1.037313	2.196014	-2.437958
5	6	0	1.473304	3.091112	-3.409738
6	6	0	1.436143	4.457563	-3.164349
7	5	0	0.033117	1.611219	-0.014172
8	6	0	-1.138749	0.574758	-0.491946
9	6	0	1.068982	0.406391	0.360920
10	6	0	1.000146	-0.433625	1.481783
11	6	0	1.988835	-1.414087	1.544699
12	6	0	2.844031	-1.389021	0.451593
13	16	0	2.399336	-0.103609	-0.633043
14	6	0	-1.372386	0.023673	-1.752947
15	6	0	-2.407147	-0.911245	-1.806987
16	6	0	-2.998174	-1.142474	-0.571017
17	16	0	-2.253485	-0.158594	0.640443
18	6	0	3.975728	-2.232818	0.193242
19	6	0	-4.095042	-2.022158	-0.268230
20	16	0	4.520865	-3.405550	1.364286
21	6	0	5.791777	-3.923199	0.329516
22	6	0	5.806499	-3.235457	-0.849622
23	6	0	4.772981	-2.272413	-0.929777
24	16	0	-4.945675	-2.878645	-1.527744
25	6	0	-5.990805	-3.626696	-0.384837
26	6	0	-5.710700	-3.241219	0.893377
27	6	0	-4.630469	-2.327686	0.962292
28	7	0	-0.385396	2.346543	1.276337
29	6	0	-1.621502	2.997390	1.361033
30	6	0	-1.966928	3.781079	2.418135
31	6	0	-1.065931	4.019066	3.482351
32	6	0	0.224111	3.465996	3.335015
33	6	0	0.544512	2.685125	2.265561
34	1	0	0.930349	5.971173	-1.723998
35	1	0	0.174466	4.383157	-0.026871
36	1	0	1.094676	1.137936	-2.676610
37	1	0	1.843605	2.716883	-4.360405
38	1	0	1.773607	5.162699	-3.918438
39	1	0	0.240847	-0.300809	2.240509
40	1	0	2.087079	-2.124566	2.357556
41	1	0	-0.809518	0.330154	-2.623430
42	1	0	-2.728723	-1.404575	-2.717457
43	1	0	6.529997	-3.412156	-1.635733
44	1	0	4.613047	-1.629011	-1.786890
45	1	0	-6.255346	-3.598497	1.758569
46	1	0	-4.252776	-1.908985	1.887618
47	1	0	-2.288673	2.845805	0.525404
48	1	0	-2.952166	4.235839	2.415970
49	1	0	-1.329481	4.639933	4.326615
50	1	0	0.997868	3.666509	4.069177
51	1	0	1.541341	2.295028	2.128590
52	1	0	6.462199	-4.708838	0.648558
53	1	0	-6.757383	-4.310944	-0.720415

Table S27. Coordinates for Optimized Structure of **M2-Py** in the first singlet excited state (S_1).

E(RB3LYP) = -3748.15015729 Hartree; E(TD-HF/TD-DFT) = -3748.12160086

opt td rb3lyp/6-31g(d) geom=connectivity

Center Number	Atomic Number	Atomic Type	Coordinates (Angstroms) X	Y	Z
1	6	0	-0.819863	6.946130	2.041922
2	6	0	-0.500177	6.131120	0.952865
3	6	0	-0.259918	4.751071	1.092594
4	6	0	-0.350545	4.240656	2.405445
5	6	0	-0.674309	5.042411	3.502628
6	6	0	-0.913856	6.405567	3.324715
7	5	0	0.081259	3.795984	-0.193831
8	6	0	1.386104	2.851854	0.139490
9	6	0	-1.155508	2.727319	-0.485487
10	6	0	2.353585	3.018133	1.107265
11	6	0	3.405476	2.062153	1.087317
12	6	0	3.269573	1.117540	0.092102
13	16	0	1.803697	1.433799	-0.816263
14	6	0	4.134308	0.002446	-0.232289
15	7	0	0.327323	4.625529	-1.518121
16	6	0	1.607452	5.070096	-1.867340
17	6	0	1.837573	5.951336	-2.884804
18	6	0	0.768863	6.495210	-3.650596
19	6	0	-0.535554	6.088447	-3.262496
20	6	0	-0.727441	5.206909	-2.236932
21	16	0	5.390665	-0.518179	0.883500
22	6	0	5.963443	-1.745100	-0.253526
23	6	0	5.187617	-1.753411	-1.385251
24	6	0	4.156795	-0.775807	-1.370884
25	6	0	-1.425568	2.008557	-1.661492
26	6	0	-2.479558	1.100467	-1.572460
27	6	0	-3.083511	1.082529	-0.304993
28	16	0	-2.282888	2.254053	0.743352
29	6	0	-4.165275	0.296804	0.140073
30	16	0	-4.971661	-0.858935	-0.934743
31	6	0	-6.055854	-1.364291	0.358434
32	6	0	-5.793149	-0.660033	1.543594
33	6	0	-4.753623	0.256367	1.415661
34	1	0	-0.992869	8.009138	1.886114
35	1	0	-0.429174	6.583724	-0.030717
36	1	0	-0.135900	3.188535	2.582603
37	1	0	-0.726852	4.604303	4.497489
38	1	0	-1.160758	7.038138	4.174421
39	1	0	2.314253	3.834554	1.819748
40	1	0	4.247286	2.081031	1.773180
41	1	0	2.415391	4.660703	-1.279000
42	1	0	2.866547	6.229645	-3.094213
43	1	0	0.936330	7.199059	-4.454635
44	1	0	-1.412397	6.477948	-3.772596
45	1	0	-1.722078	4.920243	-1.926628
46	1	0	5.364813	-2.439404	-2.206458
47	1	0	3.475615	-0.614404	-2.199447
48	1	0	-0.845472	2.167250	-2.561477
49	1	0	-2.806434	0.467866	-2.392637
50	1	0	-6.342470	-0.834498	2.459205
51	1	0	-4.401443	0.879480	2.229352
52	7	0	-7.034162	-2.291354	0.145612
53	7	0	7.021627	-2.604363	0.077159
54	6	0	8.299135	-2.053419	0.379654
55	6	0	10.798586	-0.922602	0.994553
56	6	0	8.761173	-0.926615	-0.317685
57	6	0	9.099786	-2.605650	1.392724
58	6	0	10.341531	-2.046383	1.686632

59	6	0	9.997658	-0.365600	-0.004084
60	1	0	8.144941	-0.496699	-1.100807
61	1	0	8.743761	-3.467860	1.947038
62	1	0	10.948040	-2.485278	2.474755
63	1	0	10.338778	0.508102	-0.553234
64	1	0	11.763977	-0.485361	1.232996
65	6	0	6.791137	-4.008059	0.108944
66	6	0	6.319586	-6.782267	0.140971
67	6	0	5.567259	-4.517567	0.571220
68	6	0	7.774225	-4.903921	-0.342890
69	6	0	7.538099	-6.277080	-0.316871
70	6	0	5.337368	-5.891686	0.579617
71	1	0	4.801904	-3.831740	0.919461
72	1	0	8.718178	-4.519350	-0.714915
73	1	0	8.310518	-6.954473	-0.672220
74	1	0	4.384304	-6.267309	0.943482
75	1	0	6.137696	-7.853186	0.153889
76	6	0	-8.163616	-2.374318	1.032512
77	6	0	-10.374436	-2.564891	2.724029
78	6	0	-8.911234	-1.229361	1.332947
79	6	0	-8.524863	-3.615506	1.568452
80	6	0	-9.631347	-3.705434	2.410939
81	6	0	-10.011298	-1.329619	2.183941
82	1	0	-8.635418	-0.274254	0.897164
83	1	0	-7.940297	-4.497101	1.325621
84	1	0	-9.907759	-4.668825	2.829016
85	1	0	-10.591268	-0.440735	2.413822
86	1	0	-11.234380	-2.639239	3.382800
87	6	0	-6.980017	-3.195146	-0.972539
88	6	0	-6.902447	-4.956810	-3.132554
89	6	0	-5.857241	-4.008331	-1.166722
90	6	0	-8.069280	-3.269734	-1.848218
91	6	0	-8.025982	-4.152831	-2.925030
92	6	0	-5.820949	-4.882025	-2.253204
93	1	0	-5.027192	-3.960620	-0.468982
94	1	0	-8.935941	-2.637474	-1.683501
95	1	0	-8.869123	-4.206333	-3.607260
96	1	0	-4.949496	-5.512288	-2.402922
97	1	0	-6.871893	-5.641448	-3.974810

Table S28. Coordinates for Optimized Structure of **M2-Py** in the first singlet excited state (S_1) with RCAM-B3LYP functional.

E(RCAM-B3LYP) = -3746.88173517 Hartree; E(TD-HF/TD-DFT) = -3746.80484184 Hartree

```
# opt td rcam-b3lyp/6-31g(d) geom=connectivity
```

Center Number	Atomic Number	Atomic Type	Coordinates (Angstroms) X	Y	Z
1	6	0	-0.737263	6.812644	2.245115
2	6	0	-0.432084	5.996237	1.159485
3	6	0	-0.274936	4.611495	1.292498
4	6	0	-0.425318	4.091916	2.588330
5	6	0	-0.735419	4.895207	3.680839
6	6	0	-0.896871	6.265679	3.512314
7	5	0	0.063921	3.650864	0.015369
8	6	0	1.357590	2.711241	0.364367
9	6	0	-1.164205	2.603779	-0.285790
10	6	0	2.318413	2.904857	1.321162
11	6	0	3.355004	1.932385	1.333736
12	6	0	3.198505	0.964107	0.378885
13	16	0	1.758082	1.274856	-0.543854
14	6	0	4.043475	-0.184316	0.105100
15	7	0	0.283009	4.446466	-1.306039
16	6	0	1.541629	4.717092	-1.836260
17	6	0	1.723134	5.445768	-2.968976
18	6	0	0.621504	6.001262	-3.674599
19	6	0	-0.644391	5.796787	-3.096081
20	6	0	-0.795323	5.061118	-1.956414
21	16	0	5.679277	-0.251883	0.704848
22	6	0	5.956645	-1.796049	-0.076963
23	6	0	4.839384	-2.213434	-0.737397
24	6	0	3.753370	-1.303815	-0.627508
25	6	0	-1.406310	1.923588	-1.496688
26	6	0	-2.475265	1.046989	-1.471437
27	6	0	-3.120485	1.011099	-0.225224
28	16	0	-2.333078	2.118412	0.881432
29	6	0	-4.237254	0.249923	0.151202
30	16	0	-5.037788	-0.835470	-0.978077
31	6	0	-6.173489	-1.339897	0.245011
32	6	0	-5.937749	-0.690161	1.458478
33	6	0	-4.865726	0.190614	1.395985
34	1	0	-0.847475	7.883907	2.098156
35	1	0	-0.307137	6.451274	0.183099
36	1	0	-0.266255	3.029235	2.757270
37	1	0	-0.838617	4.451204	4.667663
38	1	0	-1.134101	6.900741	4.361306
39	1	0	2.281811	3.740061	2.010027
40	1	0	4.182103	1.943863	2.035884
41	1	0	2.374501	4.303881	-1.287628
42	1	0	2.738471	5.600655	-3.318508
43	1	0	0.753468	6.583831	-4.575201
44	1	0	-1.532923	6.233447	-3.541872
45	1	0	-1.760590	4.936202	-1.488178
46	1	0	4.799034	-3.152018	-1.277268
47	1	0	2.786449	-1.465557	-1.089378
48	1	0	-0.789780	2.103177	-2.366254
49	1	0	-2.793630	0.448472	-2.317824
50	1	0	-6.528295	-0.872815	2.344848
51	1	0	-4.524153	0.775900	2.241287
52	7	0	-7.162234	-2.228963	-0.035135
53	7	0	7.170108	-2.477567	0.075950
54	6	0	8.370506	-1.860968	-0.361789
55	6	0	10.717400	-0.598051	-1.216941
56	6	0	8.368550	-1.068033	-1.511329
57	6	0	9.557966	-2.012027	0.359710

58	6	0	10.720943	-1.391142	-0.074257
59	6	0	9.533388	-0.437262	-1.927334
60	1	0	7.447930	-0.948229	-2.071935
61	1	0	9.563636	-2.614540	1.261153
62	1	0	11.635210	-1.517922	0.497747
63	1	0	9.513300	0.178547	-2.821321
64	1	0	11.627261	-0.107631	-1.547529
65	6	0	7.169122	-3.765318	0.668949
66	6	0	7.140948	-6.318164	1.822933
67	6	0	6.283358	-4.063710	1.707487
68	6	0	8.036885	-4.760422	0.210071
69	6	0	8.024655	-6.021204	0.791325
70	6	0	6.269348	-5.331854	2.271698
71	1	0	5.605764	-3.297010	2.066393
72	1	0	8.718708	-4.541141	-0.603942
73	1	0	8.705988	-6.781756	0.421643
74	1	0	5.574810	-5.545021	3.078817
75	1	0	7.130653	-7.306660	2.270687
76	6	0	-8.290479	-2.365647	0.839497
77	6	0	-10.501220	-2.662280	2.496243
78	6	0	-9.041302	-1.248646	1.199232
79	6	0	-8.647671	-3.631091	1.297394
80	6	0	-9.755260	-3.775170	2.122383
81	6	0	-10.141186	-1.401116	2.034075
82	1	0	-8.764232	-0.269341	0.823523
83	1	0	-8.058843	-4.493377	1.003591
84	1	0	-10.031482	-4.761785	2.479543
85	1	0	-10.724901	-0.530190	2.313542
86	1	0	-11.363906	-2.778184	3.143823
87	6	0	-7.130880	-3.032124	-1.223620
88	6	0	-7.101419	-4.598077	-3.515809
89	6	0	-6.065233	-3.898652	-1.455523
90	6	0	-8.187408	-2.954414	-2.127598
91	6	0	-8.170017	-3.741332	-3.271060
92	6	0	-6.050926	-4.674209	-2.608338
93	1	0	-5.257180	-3.964798	-0.734615
94	1	0	-9.013987	-2.280696	-1.928621
95	1	0	-8.991672	-3.679052	-3.976841
96	1	0	-5.219943	-5.347697	-2.789858
97	1	0	-7.089463	-5.208698	-4.412518

----

11. References

1. Parab, K.; Venkatasubbaiah, K.; Jäkle, F., Luminescent Triarylborane-Functionalized Polystyrene: Synthesis, Photophysical Characterization, and Anion-Binding Studies. *J. Am. Chem. Soc.* **2006**, *128*, 12879-12885.

2. (a) Vidal, F.; Lin, H.; Morales, C.; Jäkle, F., Polysiloxane/Polystyrene Thermo-Responsive and Self-Healing Polymer Network via Lewis Acid-Lewis Base Pair Formation. *Molecules* **2018**, *23*, 405. (b) Vidal, F.; Gomezcoello, J.; Lalancette, R. A.; Jäkle, F., Lewis Pairs as Highly Tunable Dynamic Cross-Links in Transient Polymer Networks. *J. Am. Chem. Soc.* **2019**, *141*, 15963-15971.

3. (a) Yepremyan, A.; Osamudiamen, A.; Brook, M. A.; Feinle, A., Dynamically tuning transient silicone polymer networks with hydrogen bonding. *Chem. Commun.* **2020**, *56*, 13555-13558. (b) Li, N.; Gu, S.; Wu, Q.; Wu, J., Shear-stiffening supramolecular polymers: fabrication, modification and application. *RSC Appl. Polym.* **2025**, *3*, 299-316.
